# Understanding and Kinetic Modeling of Complex Degradation Pathways in the Solid Dosage Form: The Case of Saxagliptin

**DOI:** 10.3390/pharmaceutics11090452

**Published:** 2019-09-02

**Authors:** Blaž Robnik, Blaž Likozar, Baifan Wang, Tijana Stanić Ljubin, Zdenko Časar

**Affiliations:** 1 Lek Pharmaceuticals d.d., Sandoz Development Center Slovenia, Verovškova ulica 57, SI-1526 Ljubljana, Slovenia; 2 Faculty of Pharmacy, Chair of Medicinal Chemistry, University of Ljubljana, Aškerčeva cesta 7, SI-1000 Ljubljana, Slovenia; 3 National Institute of Chemistry, Hajdrihova 19, SI-1001 Ljubljana, Slovenia

**Keywords:** solid dosage form stability, PEG degradation, drug substance degradation, reactive impurities, kinetic modeling, ab initio calculations, saxagliptin

## Abstract

Drug substance degradation kinetics in solid dosage forms is rarely mechanistically modeled due to several potential micro-environmental and manufacturing related effects that need to be integrated into rate laws. The aim of our work was to construct a model capable of predicting individual degradation product concentrations, taking into account also formulation composition parameters. A comprehensive study was done on active film-coated tablets, manufactured by layering of the drug substance, a primary amine compound saxagliptin, onto inert tablet cores. Formulation variables like polyethylene glycol (PEG) 6000 amount and film-coat polymer composition are incorporated into the model, and are connected to saxagliptin degradation, via formation of reactive impurities. Derived reaction equations are based on mechanisms supported by ab initio calculations of individual reaction activation energies. Alongside temperature, relative humidity, and reactant concentration, the drug substance impurity profile is dependent on micro-environmental pH, altered by formation of acidic PEG degradation products. A consequence of pH lowering, due to formation of formic acid, is lower formation of main saxagliptin degradation product epi-cyclic amidine, a better resistance of formulation to high relative humidity conditions, and satisfactory tablet appearance. Discovered insights enhance the understanding of degradational behavior of similarly composed solid dosage forms on overall drug product quality and may be adopted by pharmaceutical scientists for the design of a stable formulation.

## 1. Introduction

Drug substance degradation kinetics in solid-state pharmaceutical systems is rarely studied and modeled based on actual degradation mechanisms. Multiple potential micro-environmental and manufacturing related effects need to be integrated into rate laws to effectively describe observed behaviors. Published studies that also take into account formulation composition variables are; therefore, limited [1,2,3,4]. Polyethylene glycols (PEGs), as one of the most commonly used excipients in pharmaceutical formulations, particularly as film-coat plasticizers, have been largely investigated over recent decades in relation to their susceptibility to degradation [5,6,7,8]. Not only can changes to their chemical structure affect functional characteristics of pharmaceutical dosage forms and consequently their performance, they can decrease drug’s potency through formation of reactive impurities. Reactive impurities control in excipients is a challenge, since they can originate from synthetic processes, typically with high batch-to-batch variability, or from final dosage form manufacture and storage. In PEGs, prevalent reactive impurities are hydroperoxides and low molecular weight organic compounds such as aldehydes (e.g., formaldehyde (F) and acetaldehyde (A)) and acids (e.g., formic (FA) and glycolic acid (GA)). Formation of the last two is a consequence of radical-initiated oxidation reactions, propagated by peroxides/oxygen, and are reported [5,6,7,8] to be catalyzed by different material properties, as for instance PEG chain length, moisture content, presence of residual metals, and other.

**Scheme 1:**
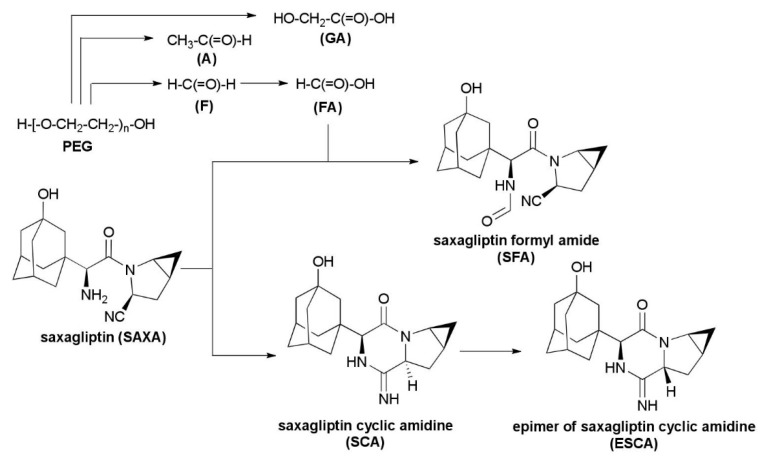
Simplified scheme of polyethylene glycol (PEG) and saxagliptin (SAXA) degradation.

The most detrimental is the effect of these reactive impurities on formulations with low doses of active pharmaceutical ingredients [9,10,11,12]. An interesting example in this context is a dipeptidyl peptidase-IV inhibitor saxagliptin (SAXA), a primary amine compound, commercially available in tablet formulation with concentrations of 2.5 and 5 mg per tablet. SAXA is present in tablet film-coat, together with inactive ingredients: polyvinyl alcohol, polyethylene glycol, titanium dioxide, talc, and iron oxides [13]. It is a very unstable drug substance, whose complex degradation pathways in sold dosage forms have not been previously explained. The low dose of drug product containing SAXA has the highest excipient to drug substance ratio. Therefore, aldehydes and carboxylic acids which are prone to react with amine functionality and are present as impurities in many excipients, PEG being a primary example, present a potential risk for degradation of SAXA. It is reported [14] that SAXA is prone to intra-molecular cyclization, which is accelerated when formulations are subject to commonly used processing activities such as wet granulation, roller compaction, or tableting. Furthermore, most commonly used excipients, when mixed with this compound, can promote the rate of cyclization. Tablet formulation comprising SAXA; therefore, utilizes active film coating technology (i.e., layering of the drug substance onto inert tablet cores) as a solution to the incompatibility issues [15]. Results of our preliminary stress stability testing in solid-state and in solution, together with performed excipient compatibility studies (data not reported here), suggested intra-molecular cyclization to be the major and most important degradation pathway of SAXA. Similar findings are presented by Jones et al., who studied stability of SAXA in different solvents and identified that solvent’s proton affinity, which is representative for its gas-phase basicity, and the solvent’s autoprotolysis equilibrium constant correlate well with the rate of intra-molecular cyclization [16]. Nonetheless, when dispersions with the same concentration of drug substance, but varying concentrations of film forming polymers, were layered onto tablet cores and subjected to stress stability testing, contradicting SAXA impurity profiles were observed. Saxagliptin cyclic amidine impurity (SCA), its epimer, epi-cyclic amidine impurity (ESCA), and saxagliptin formyl amide impurity (SFA) all formed in different ratios, dependent on the minor changes made in film forming polymer composition (simplified reactions are illustrated in Scheme 1). To our knowledge, the relationship between both SCA epimers and SFA has only been briefly mentioned in the patent literature [17,18], but not explained in any previous work.

Elements favoring one or the other reaction pathway are investigated in our study. Moreover, in our work we break down a complex scheme of solid-state degradation reactions on an active tablet surface by building a model, capable of predicting individual degradation product concentrations for desired formulation composition, temperature and relative humidity, over time. In support of proposed mechanisms, ab initio calculations of individual reaction activation energies are studied.

## 2. Materials and Methods

### 2.1. Materials

SAXA in the form of hydrochloride dihydrate was purchased from MSN Pharmachem Pvt. Ltd. (Hyderabad, India) and was used also as an external standard for analytical purposes with determined purity on as is basis of 80.9%. Other raw materials in film-coated tablets are: lactose monohydrate purchased from DMV-Fonterra Excipients B.V. (Groningen, Netherlands), cellulose microcrystalline from FMC BioPolymer (Philadelphia, PA, USA), croscarmellose sodium from FMC BioPolymer (Philadelphia, PA, USA), magnesium stearate from Mallinckrodt Chemical Inc. (Dublin, Ireland), hypromellose (HPMC 603) from Shinetzu Chemical Co. (Tokyo, Japan), polyethylene glycol 6000 (PEG 6000) from Clariant Gendorf KG Plant (Burgkirchen an der Alz, Germany), and hydrochloric acid 0.1 M from Carlo Erba reagents (Milano, Italy). In binary mixtures study in addition to listed chemicals, butylhydroxytoluene purchased from Merck KGaA (Darmstadt, Germany) was used. Standards of SAXA impurities (ESCA, SCA, SFA) for quantification were obtained according to the literature procedures [16,19]. Their structures were confirmed with NMR, MS, and IR. Low molecular weight organic impurities standards for analysis (formic acid (FA), glycolic acid (GA), formaldehyde (F), and acetaldehyde (A)) were obtained from Merck KGaA (Darmstadt, Germany).

Reagents used for analysis: hydrochloric acid (HCl) Titrisol^®^ solution, diammonium hydrogen phosphate, tert-butyl methyl ether (MTBE), *O*-phosphoric acid 85%, pyridine, 1-ethyl-3-(3′-dimethylaminopropyl)carbodiimide HCl (EDC), all from Merck KGaA (Darmstadt, Germany); acetonitrile (ACN) from J.T. Baker (Radnor, PA, US); 2-nitrophenylhydrazin HCl (NPH) from Tokyo Chemical Industry Co., LTD. (Tokyo, Japan). Additionally, purified water used for analysis was purified with Milli-Q gradient system from Merck Millipore (Burlington, MA, US). Certipur^®^ buffer solutions were used for pH calibration of pH meters from Merck KGaA (Darmstadt, Germany). All other reagents were of analytical grade.

Other equipment and materials used for analysis: Centrifuge Eppendorf 5804 R, Eppendorf (Hamburg, Germany), Amicon Ultra-4, Ultracel^®^ 3K ultrafilters (Merck KGaA, Darmstadt, Germany), Handy step^®^ electronic repetitive pipette (Brand GmbH, Wertheim, Germany), Biohit Picus pipettes (Sartorius, Göttingen, Germany), Thermomixer comfort 5355 (Eppendorf, Hamburg, Germany), and Millex-GV Syringe Filter Unit, 0.22 µm, PVDF (Merck KGaA, Darmstadt, Germany).

### 2.2. Film-Coated Tablets

Only standard excipients for preparation of tablets were used. Inert tablet cores were prepared by direct compression technology. Lactose monohydrate, cellulose microcrystalline, croscarmellose sodium, and magnesium stearate were blended and compressed into tablet cores by using a rotary tableting machine.

**Figure 1:**
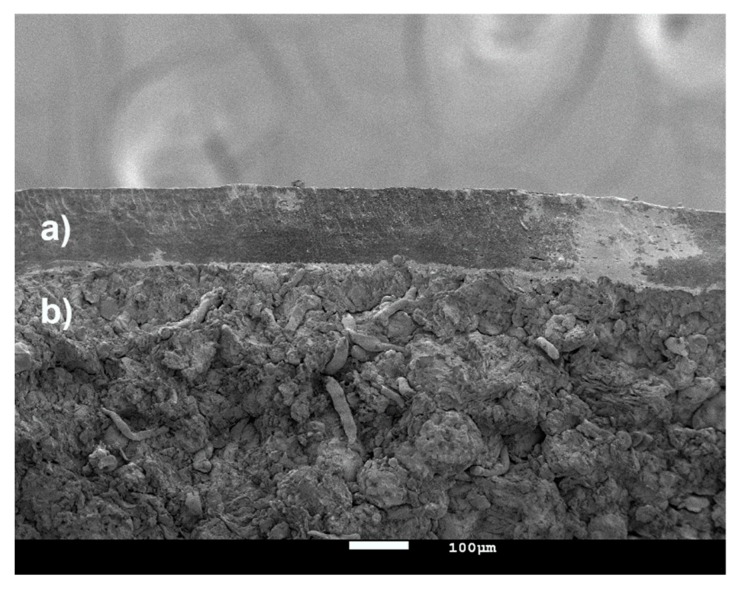
A scanning electron microscope (SEM) image of a film-coated tablet cross-section (magnification 100×); (**a**) active film-coat, (**b**) inert tablet core.

Film-coated tablets were prepared from inert tablet cores by active film coating technology. Four different coating dispersions were prepared by dissolving equivalent amount of SAXA, but different amounts of film-coat plasticizer PEG 6000 and film-forming polymer HPMC 603 in water and adjusting pH to around 2.5 by hydrochloric acid. Coating dispersions were then sprayed onto inert tablet cores, while being rotated in a perforated drum with a heated air stream, resulting in water evaporation and formation of the active film-coat on inert tablet cores surface. The same inert tablet cores are used for all four different formulations (see Table 1 for additional details). Concentration of PEG 6000 was varied in order to provoke different conditions for SAXA degradation. HPMC 603 concentration was varied accordingly, to make up for the difference in tablet mass. An example of a film-coated tablet captured with scanning electron microscope JEOL JSM-70001F is presented in Figure 1.

### 2.3. Stability Study Protocol

Prior to stability study initiation, each of the film-coated tablet formulations was exposed to 10%, 30%, and 50% for 3 days in order to study the effect of relative humidity. Climatic chamber MEMMERT HPP110, with adjustable relative humidity setup was used. Next, samples were subjected to prescribed conditions in tightly sealed glass containers (Table 2 depicts sampling protocol). Different temperature conditions include standard International Conference on Harmonisation (ICH) conditions for accelerated (40 °C) and intermediate (30 °C) stability programs and more stressful conditions of 50 and 60 °C. Climatic chambers (Vötsch VC 0100, from Vötsch Industrietechnik GmbH) and incubators (Binder BF 115, BF 720, and BF 729 from Binder GmbH, Tuttlingen, Germany) were used. At each time-point, from day 7 to day 108, analysis of SAXA-related substances and degradation products (UHPLC), low molecular weight organic impurities (UHPLC), water activity (aw), pH, and coloration check were performed. In binary mixtures study, lyophilisator Scanvac Coolsafe from LaboGene (Lillerød, Denmark) for flushing the samples with nitrogen from Messer GmbH (Krefeld, Germany) was utilized.

### 2.4. Saxagliptin Related Substances and Degradation Products Determination

Saxagliptin related substances and degradation products (SFA, SCA, and ESCA) were determined by liquid chromatography method (see Supporting Information).

### 2.5. Low Molecular Weight Organic Impurities Determination

Low molecular weight organic impurities (GA, A, F, and FA) were determined by liquid chromatography method (see Supporting Information).

### 2.6. Other Measurements (pH, Water Activity, Coloration)

For pH measurement, three film-coated tablets were sonicated in plastic tubes, in 4 mL of water, until disintegration. Suspension was transferred into Amicon Ultra-4, Ultracel^®^ 3K ultrafilters, and centrifuged at 5000 rpm for 1 h. pH of the filtrate was measured with Seven Multi pH meter (Mettler Toledo, Columbus, OH, USA). Water activity (a_w_) [20] measurements were performed on eight uncrushed tablets with Aqualab 4TE (Meter Group, Inc., Pullman, WA, USA). Appearance of tablets was photographed at each time-point and coloration was quantified with ImageJ software (version 1.50i, Wayne Rasband, NIH, USA) using built-in (R+G+B)/3 measurement.

### 2.7. Statistical Evaluation of Data

Statistical evaluation was done in MODDE ^®^ Pro, version 11.0.1.1878, MKS Umetrics AB, DoE software package. The relationship between factors: time (7–180 days), temperature (30–60 °C), relative humidity (10%–50% RH), and tablet composition (PEG to SAXA = 0.8, 1.0, 1.2, and 1.4) (no interactions) and responses: SCA, ESCA, SFA, F, FA, aw, pH, and coloration was established. Logarithmic data transformation was performed for some responses prior to MLR (multiple linear regression) fitting. Two software outputs were utilized, coefficient plot and correlation matrix. For simpler comparison, coefficients were scaled and centered.

### 2.8. Kinetic Model Development

Data obtained at temperatures 30, 40, and 50 °C were used to construct the model. At least four time points for each temperature and relative humidity condition were considered sufficient to build a reliable model, capable of predicting temperature dependent kinetics. Starting model, built for temperature of 40 °C and RH 10%, was subsequently expanded to other conditions. Additional model optimization was done only for 40 °C and 10% RH. A set of ordinary differential equations was solved in Berkeley Madonna^®^, version 8.0.1, mathematical modeling software package. Runge–kutta (fourth order) integration algorithm and a tolerance of 0.00001 was used. Nonlinear regression analysis was performed by Levenberg–Marquardt algorithm.

### 2.9. Computational Methods

All calculations were executed using Gaussian 09, Revision E.01 [21]. The geometries of reactants, intermediates, products, and transition structures were optimized at the B3LYP/6-31G(d,p) level incorporate Tomasi’s polarized continuum model (PCM) corrections [22,23] for water and formic acid. Internal parameters provided by Gaussian 09 were used to model the formic acid solvent medium (*ε* = 58.50) as the bulk solvent. The calculated energies (∆E, 298.15 K, 1.0 atm) result from the sum of the Gibbs free energies as obtained from the frequency analysis at the B3LYP/6-31G(d,p) level and PCM corrections. Energy values are given in kcal/mol and are transformed to kJ/mol for comparison to regressed values. Frequency calculations for all stationary points were carried out to describe them either as minima (*i* = 0) or as first-order transition states (*i* = 1). For all transition structures, visualization of the imaginary frequencies corresponded to the expected normal mode for the elementary step under investigation. Intrinsic reaction coordinate calculations (IRC) were performed from the transition states in forward and reverse directions to confirm the lowest energy reaction pathways that connect the corresponding minima (see Supporting Information).

## 3. Results and Discussion

### 3.1. Film-Coated Tablet Stability Study

**Figure 2:**
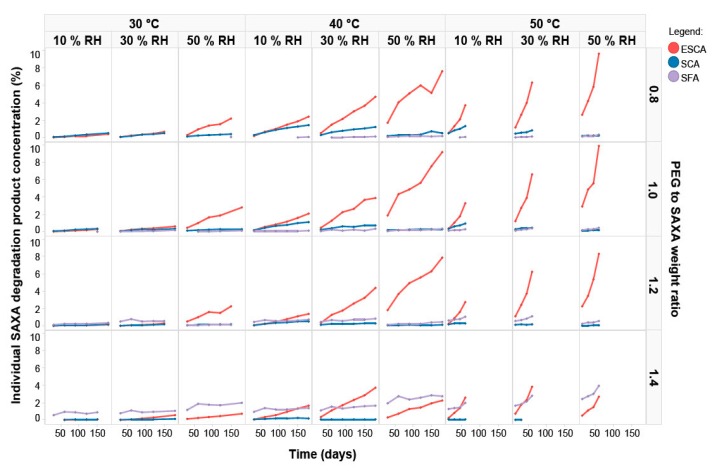
Saxagliptin (SAXA) degradation profile. Concentration of three main SAXA degradation products: cyclic amidine (SCA), epimer (ESCA) and formyl amide (SFA) are graphed on the y-axis (% against SAXA, *w/w*), and time is graphed on the x-axis (days).

Results of SAXA degradation are depicted in Figure 2. It is worth noting that no impurity originating from drug substance or film-coated tablet manufacture is above the quantitation limit of the analytical method and no SAXA degradation occurred prior to stability study initiation. Total SAXA degradation does not exceed 15%, of which, the three main degradation products graphed (SCA, ESCA, and SFA) present the majority. Degradation is temperature and humidity dependent. Raising either, generally results in higher SAXA degradation. As anticipated, tablet composition, namely film-coat plasticizer (PEG 6000) content, affects impurity profile. When PEG to SAXA ratio is high, SFA formation increases, SCA, and ESCA decrease, and vice versa when PEG content is low.

**Figure 3:**
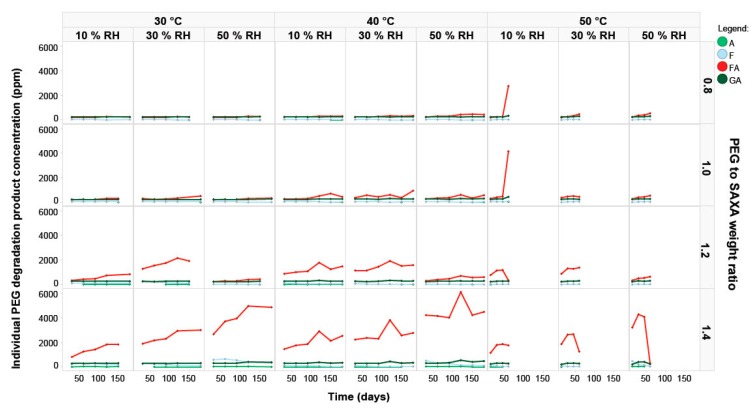
Polyethylene glycol (PEG) degradation profile. Concentration of main PEG degradation products: glycolic acid (GA), acetaldehyde (A), formaldehyde (F) and formic acid (FA) are graphed on y axis (ppm against tablet film-coat; *w/w*), and time is graphed on x axis (days).

Concentration of reactive organic impurities (i.e., acids and aldehydes), in ppm against tablet film-coat (*w/w*), was also measured at every time point (Figure 3). Similar to SAXA degradation products, low molecular weight organic impurities (GA, A, F, FA) levels at the start of stability study were equal to or close to zero. Distinctive amounts of FA are formed in some of the samples. The most obvious is link between FA formation and tablet composition, again, owing to the difference in PEG concentration. Higher PEG to SAXA ratio is responsible for higher FA levels observed. FA generation is also humidity and temperature dependent; however, temperature dependence in particular is not clear, due to the fast observance of concentration plateau (30 and 40 °C) and apparent drop in FA assay (50 °C) at the last time point (60 days). Other low molecular weight organic impurities form at much lower concentrations, as shown already in other unrelated studies [5,24]; therefore, their change over time is not well visible on the presented graphs. Closer evaluation of GA, A, and F profiles shows primarily tablet composition dependent degradation behavior, similar to FA, which is also influenced by changes in relative humidity and temperature. Concentrations of F reach maximum in the first couple of time points and later decrease, assumingly due to conversion to FA.

**Figure 4:**
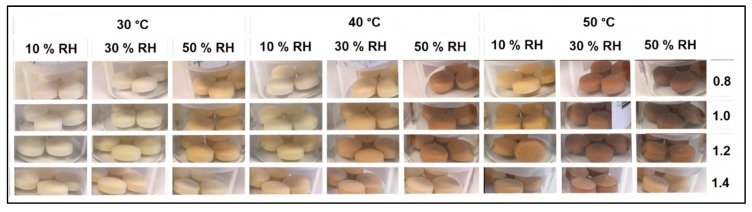
Photographs of individual film-coated tablet (FCT) formulation (polyethylene glycol (PEG) to saxagliptin (SAXA) = 0.8, 1.0, 1.2, and 1.4) exposed to different temperatures (30–50 °C) and relative humidity conditions (10%–50% RH). For sake of brevity, only two months of stability results are presented.

In addition to chromatographic data capture, water activity (a_w_) along with pH measurements were performed and also tablet appearance was examined. A_w_ values depend on exposure to different relative humidity conditions prior to the subjection to elevated temperatures and stay constant over time, whereas formulation pH is changing (Table 3). Composition, temperature and relative humidity all contribute to pH lowering. Again, the biggest difference is observed amongst different compositions, yet relative humidity and temperature also affect the pH, but in lesser extent. Lastly, very diverse tablet coloration, from white/off-white to dark brown, is observed, with no apparent correlation with composition (Figure 4).

We used MODDE^®^, a statistical analysis software, to build MLR models for complementary evaluation of measured parameters on stability. Models obtained have reasonably high R^2^ and Q^2^ (R^2^ predicted) values: R^2^/Q^2^ (SCA) = 0.76 / 0.74, R^2^/Q^2^ (ESCA) = 0.82/0.81, R^2^/Q^2^ (SFA) = 0.86/0.85, R^2^/Q^2^ (F) = 0.57/0.54, R^2^/Q^2^ (FA) = 0.76/0.75, R^2^/Q^2^ (pH) = 0.73/0.71, R^2^/Q^2^ (aw) = 0.96/0.96 and R^2^/Q^2^ (coloration) = 0.73/0.71 (Table S12, Supporting Information).

**Figure 5:**
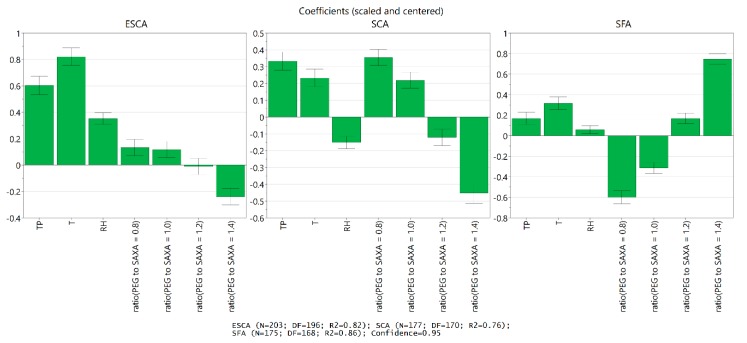
Scaled and centered coefficients for cyclic amidine (SCA), epimer (ESCA) and formyl amide (SFA). TP (time point), T (temperature), RH (relative humidity), and polyethylene glycol (PEG) to saxagliptin (SAXA) weight ratios 0.8, 1.0, 1.2, and 1.4.

Figure 5 presents MODDE^®^ output for responses SCA, ESCA, and SFA, where scaled and centered coefficient bars were used to compare significance of individual factor. For SAXA degradation products, all four coefficients: time, temperature, relative humidity, and tablet composition are significant; however, their order and relative height depends on the degradation product. SCA is most affected by tablet composition and time, ESCA by temperature and time, SFA by tablet composition and temperature. Both modeled PEG degradation products, F and FA, have tablet composition as the most significant factor. Coefficient of composition with PEG to SAXA ratio of 1.4 is strongly positive, composition with PEG to SAXA ratio 1.2 is close to zero, compositions with the lowest two PEG to SAXA ratios are negative. pH is most affected by composition, a_w_ almost exclusively by relative humidity and tablet coloration by temperature and tablet composition (Table S12, Supporting Information).

We used correlation matrix to identify strong linear relationships (*r* > 0.7) also between responses. There is a strong relationship between pH and FA (*r* = − 0.75), which indicates that FA formation is a major factor in pH decrease, as well as between FA and SFA (*r* = − 0.86) and also pH and SFA (*r* = − 0.82), meaning SFA formation is strongly related to lowering of the pH (predominantly through FA formation). Another strong correlation is observed between coloration and ESCA concentration, suggesting that formed impurity is responsible for tablets turning yellow or even brown (Table S13, Supporting Information). Phenomenological and statistical evaluation of stability study results on four different formulations, exhibited, alongside with the expected higher increase of impurities at higher temperatures and relative humidity, a strong influence of PEG to SAXA ratio on impurity profile, pH, and tablet coloration.

### 3.2. Kinetic Model Development

As indicated in the literature [25], while the degradation to GA compound (Equation (1)) is reported for aqueous PEG solution oxidations, reactions, listed as Equations (2)–(4), are observed even in a dry non-aqueous medium [5,26]. In this respect, there was no presented notion that the reactions would need anything else than an oxidant, this being oxygen, present in a comparatively large excess to exposed PEG, to proceed. In addition to some minor impurities, GA, A, F, and consequent FA were detected as predominant PEG degradation products, evolving as indicated in a simplified reaction scheme (*r*_1_–*r*_4_), embodied by Equations (5)–(8) [5,25,26,27,28,29], introducing surplus O_2_ (oxidant). Forward reaction rates are thus simplified. Note, that FA forms exclusively through F oxidation. Hydrolysis of well-known PEG degradation products PEG-formyl esters [5] was not accounted for in order to reduce model complexity. *k*_i_ and *k*_i_’ represent reaction rate constants, intrinsic and apparent, respectively, while *c*_i_ denotes the concentration of species *i*. As only a part of PEG was exposed to air, its initial concentration, *c*_PEG,0_ was defined as total prepared amount, *c*_PEG,TOT,0_, reduced by reactive PEG polymer fraction, *x*_PEG_.

(1) $$H-[-O-CH_{2}-CH_{2}-{]}_{n}-OH\overset{O_{2}}{\to }H-[-O-CH_{2}-CH_{2}-{]}_{n-1}-OH+HO-CH_{2}-C\left(=O\right)-OH.$$

(2) $$H-[-O-CH_{2}-CH_{2}-{]}_{n}-OH\overset{\frac{1}{2}\;O_{2}}{\to }H-[-O-CH_{2}-CH_{2}-{]}_{n-1}-OH+CH_{3}-C\left(=O\right)-H.$$

(3) $$H-[-O-CH_{2}-CH_{2}-{]}_{n}-OH\overset{\frac{1}{2}\;O_{2}}{\to }H-[-O-CH_{2}-CH_{2}-{]}_{n-1}-O-CH_{2}-OH+H-C\left(=O\right)-H.$$

(4) $$H-C\left(=O\right)-H\overset{\frac{1}{2}\;O_{2}}{\to }H-C\left(=O\right)-OH.$$

(5) $$PEG\overset{O_{2}}{\to }GA;r_{1}=k_{1}c_{PEG}c_{O_{2}}\approx k_{1}^{\prime }c_{PEG}.$$

(6) $$PEG\overset{\frac{1}{2}\;O_{2}}{\to }A;r_{2}=k_{2}c_{PEG}c_{O_{2}}\approx k_{2}^{\prime }c_{PEG}.$$

(7) $$PEG\overset{\frac{1}{2}\;O_{2}}{\to }F;r_{3}=k_{3}c_{PEG}c_{O_{2}}\approx k_{3}^{\prime }c_{PEG}.$$

(8) $$F\overset{\frac{1}{2}\;O_{2}}{\to }FA;r_{4}=k_{4}c_{F}c_{O_{2}}\approx k_{4}^{\prime }c_{F}.$$

(9) $$C_{PEG,0}=X_{PEG}C_{PEG,TOT,0}.$$

**Figure 6:**
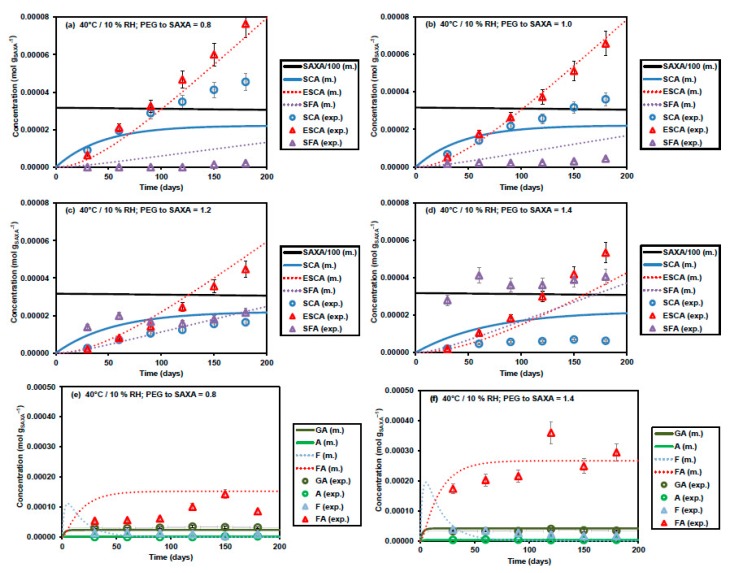
The concentration of saxagliptin (SAXA) degradation products (amidine (SCA), epimer (ESCA) and formyl amide (SFA)) (**a**–**d**) or polyethylene glycol polymer (PEG) (glycolic acid (GA), acetaldehyde (A), formaldehyde (F) and formic acid (FA)) (**e**–**f**) reactions. Lines represent fitted model (m.), markers experimental data points (exp.). Tablet degradation tests were executed at the temperature of 40 °C, the nominal water activity of 10% and the initial polyethylene glycol (PEG) to SAXA weight ratios of 0.8 (**a**,**e**), 1.0 (**b**), 1.2 (**c**) and 1.4 (**d**,**f**). A relative error of 10% is set based on sum of estimated analytical and sample variability.

Regarding SAXA degradation process, reactions (Equations (10)–(12)) were constructed, based on specific available literature [16,30,31], amidine, epimer, and formyl amide formation mechanisms [19,32,33,34,35,36,37] and own experimental measurements. Jones et al. identified solvent’s propensity to participate in proton transfer and ESCA autocatalysis as the two main contributors of SAXA to ESCA conversion [16]; however, to our set of solid-state reactions, this does not directly apply, owing to slower reaction rates and specific micro-environmental conditions, established by tablet excipients. Namely, SAXA is firstly converted to amidine (SCA) (*r*_5_), serially followed by the formation of epimer (ESCA) (*r*_6_), whereas formyl amide (SFA) (*r*_7_) is produced in parallel. While the first two reactions (*r*_5_/*r*_6_) were observed to be catalyzed by OH^−^, formylation (*r*_7_) is promoted by H^+^. Equations (13)–(15) were fitted and are depicted in the Figure 6.

(10) $$SAXA\overset{OH^{-}}{\to }SCA$$

(11) $$SCA\overset{OH^{-}}{\to }ESCA$$

(12) $$SAXA+FA\overset{H^{+}}{\to }SFA$$

(13) $$r_{5}=k_{5}\left(c_{OH^{-}}\right)c_{SAXA}\approx {k_{5}}^{\prime }c_{OH^{-}}c_{SAXA}$$

(14) $$r_{6}=k_{6}\left(c_{OH^{-}}\right)c_{SCA}\approx {k_{6}}^{\prime }c_{OH^{-}}c_{SCA}$$

(15) $$r_{7}=k_{7}\left(c_{H^{+}}\right)c_{SAXA}c_{FA}\approx {k_{7}}^{\prime }c_{H^{+}}c_{SAXA}c_{FA}$$

Even though reactions were firstly tried to be modeled as not dependent on pH/pOH, it was observed that upon solely varying PEG content, taking into account mere FA level change, will not satisfactory describe the distribution of SAXA degradation products. It was initially hypothesized, as with PEG, that only a part of SAXA was available, its initial concentration, *c*_S,0_, being defined as prepared nominal amount, *c*_SAXA,TOT,0_, reduced by ingredient SAXA reactive fraction, *x*_SAXA_, all of this owing to a small reaction extent observed Equation (16). Nonetheless, measurements failed to indicate the presence of a plateau in SAXA decrease, while regressing *x*_SAXA_ resulted in the value of unity, pointing to the fact that SAXA, contrary to PEG, was all reactive.

(16) $$c_{SAXA,0}=x_{SAXA}c_{SAXA,TOT,0}$$

While the model of Equations (1)–(16) was firstly developed, fitted and presented for the nominal water activity of 10% (Figure 6), it was later on translated to a higher air humidity; specifically, corresponding to 30–50%, where the following was observed. pH, related to pOH through water ionization constant (*K*_W_), was a function of humidity (*c*_H2O_) (Equation (17)). Moreover, *x*_PEG_ was also hypothesized to be dependent on the latter (Equation (18)), as water on surface (*k*_H_ being its Henry adsorption constant) would provide a suitable PEG reaction medium [5,25,26,27,38].

While water’s stoichiometric coefficient, *ν*_H2O_, accounts for the number of H_2_O molecules that interact with a single PEG unit, it can be readily integrated with *k*_H_ into a single proportionality constant, *k*_H_’, to establish a correlation (Equation (19)).

(17) $$c_{H^{+}}=\frac{K_{W}}{c_{OH^{-}}}=f\left(c_{H_{2}O}\right)$$

(18) $$x_{PEG}=g\left(c_{H_{2}O}\right)$$

(19) $$x_{PEG}\approx \nu_{H_{2}O}k_{H}\left(T\right)c_{H_{2}O}={k_{H}}^{\prime }\left(T\right)c_{H_{2}O}$$

Moreover, measurements were executed at various degradation temperatures as well. To account for the difference from the initial examined temperature of 40 °C (Figure 6), reaction rate constants were described using the Arrhenius Equation (Equation (20)). In the latter, *R* represents the molar gas constant, *T* temperature, *A*_i_ constants’ pre-exponential factor and *E*_a,i_ their activation energy; *A*_i_/*E*_a,i_ were subject to regression.

(20) $${k_{i}}^{\prime }=A_{i}e^{-\frac{E_{a,i}}{RT}}$$

The concentration profiles of undesired SAXA degradation products (SCA, ESCA and SFA) or PEG degradation reactions products (GA, A, F and FA) is presented in the Figure 6, where tablet degradation tests were executed at the temperature of 40 °C, the nominal water activity of 10% and the initial PEG to SAXA weight ratios of 0.8, 1.0, 1.2, and finally, 1.4. It can be seen that SAXA decreases slowly and the effect of PEG to SAXA ratio is unintelligible. Furthermore, SCA as an intermediate demonstrates the fastest concentration increase, while the maximum is not surpassed within the time of measurements (200 days). PEG to SAXA ratio elevation negatively affects both SCA amount, as well as kinetics, as the reaction Equation (10) is catalyzed by OH^−^, while increasing PEG causes a decrease in pH, partially through the oxidations (Equations (1)–(4)). The latter (in average) resulting in the pH of 5.63, 5.63, 5.53 and 5.43 for the PEG to SAXA of 0.8, 1.0, 1.2 and 1.4. ESCA is produced consecutively from SCA, while the latter being catalyzed by OH^−^ also decreases ESCA concentration, as well as kinetics, mirroring the behavior of SCA. ESCA is at 200 days still increasing constantly, as SAXA is not consumed for ESCA to level, as in typical consecutive reactions. Parallel SFA production reaction is conversely, catalyzed by H^+^, introducing FA as a reactant of formylation as well. An increase in PEG to SAXA ratio thus elevates FA concentration (Figure 6e–f), by proxy also decreasing pH, which, in turn, notably promotes SFA production amount/kinetics, SFA still rising at 200 days, similarly as ESCA. Figure 6e–f also indicates that PEG degradation is concluded comparatively fast, while the reactions of SAXA proceed. Agreement between measurements and the model is not ideal as the number of parameters was intentionally maintained limited (*A*_i_/*E*_a,i_/*x*_PEG_).

**Figure 7:**
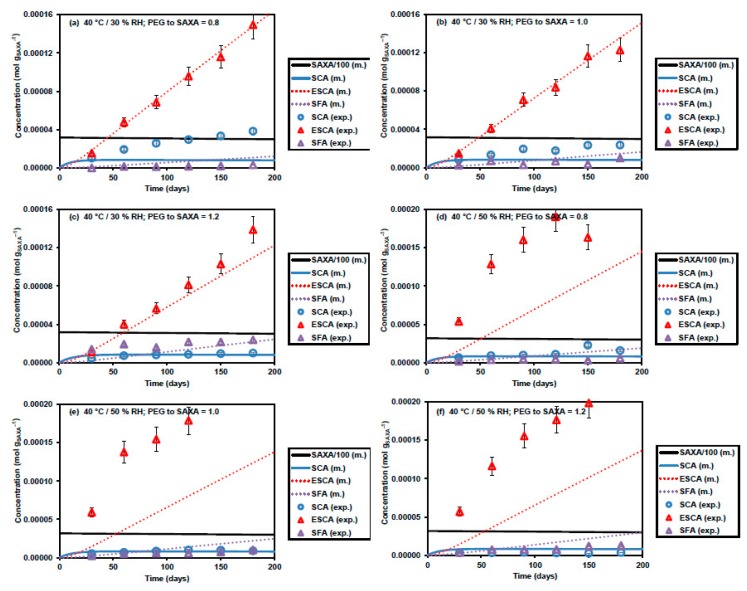
The concentration of saxagliptin (SAXA) degradation products (amidine (SCA), epimer (ESCA), and formyl amide (SFA)) (**a**–**f**) reactions. Lines represent fitted model (m.), markers experimental data points (exp.). Tablet degradation tests were executed at the temperature of 40 °C, the nominal water activity of 30% (**a**–**c**) or 50% (**d**–**f**), and the initial polyethylene glycol (PEG) to SAXA weight ratios of 0.8 (**a**,**d**), 1.0 (**b**,**e**), and 1.2 (**c**,**f**). The data for the measurements at 40 °C, 10%, and 0.8–1.4 PEG to SAXA is presented in the Figure 6, respectively. A relative error of 10% is set based on sum of estimated analytical and sample variability.

The concentration profile of the undesired SAXA degradation reaction products (SCA, ESCA and SFA), where tablet degradation tests were executed at the temperature of 40 °C, the nominal water activity of 30% or 50% and the initial PEG to SAXA weight ratios of 0.8, 1.0, and finally, 1.2, is presented in Figure 7. The most notable effect of humidity is the increase of *x*_PEG_, while SAXA degradation reaction products are influenced only indirectly through the concentration of FA in formylation, as well as via pH, which, alongside increasing *x*_PEG_, almost exclusively decreased, save for the PEG to SAXA of 1.0. Following the previous reasoning (Figure 6), an increase in humidity will yield a decreased SCA, as well as ESCA component amount/kinetics, whereas the effect on SFA is the opposite, SFA rising even more substantially, as can be captured by the model proposed. While the agreement of the model with measurements is excellent up to 30% humidity, the prediction of ESCA is underestimating later on; nonetheless, it has to again be noted that, for example, all ESCA concentration descriptions were regressed through *r*_6_ only. Separate term for humidity within individual rate constant could be considered; however, it would increase the complexity of our calculations dramatically; therefore, it was not further evaluated [39].

**Figure 8:**
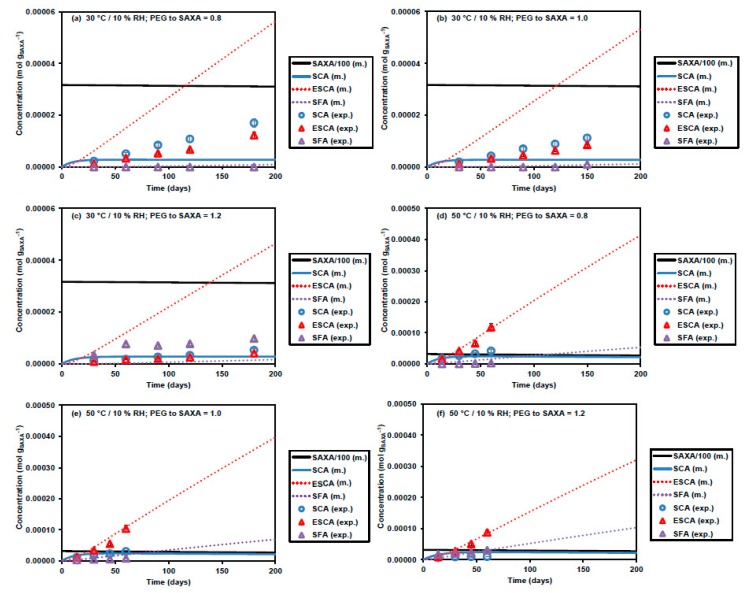
The concentration of saxagliptin (SAXA) degradation products (amidine (SCA), epimer (ESCA) and formyl-amide (SFA)) (**a**–**f**) reactions. Lines represent fitted model (m.), markers experimental data points (exp.). Tablet degradation tests were executed at the temperature of 30 °C (**a**–**c**) or 50 °C (**d**–**f**), the nominal water activity of 10% and the initial polyethylene glycol (PEG) to SAXA weight ratios of 0.8 (**a**,**d**), 1.0 (**b**,**e**), and 1.2 (**c**,**f**). The data for the measurements at 40 °C, 10%, and 0.8–1.4 PEG to SAXA is presented in the Figure 6, respectively. A relative error of 10% is set based on sum of estimated analytical and sample variability.

The effect of temperature may be seen by comparing the measurements at 30, 40, and 50 °C, presented in the Figure 6 and Figure 8; model seems to provide a good description, which is poorer only at 30 °C, where it overestimates ESCA. With the concentrations of SCA, ESCA, and SFA generally increasing with temperature at comparable degradation conditions (time, formulation, etc.), it can be noted that at 30 °C, contrary to 40–50 °C, SCA dominates over ESCA, indicating a slower consecutive reaction. Looking at the rates *r*_5_/*r*_6_ (Equation (13)/(14)), the latter can be attributed to one or more of the following: temperature, pH, or the concentrations of reactants. Table 4 indicates that the formation of epimer is thermally insensitive, thus temperature decrease is resulting in favoring *r*_6_. pH is generally decreasing with temperature, notably when rising from 30 to 40 °C, consequently, levelling off a bit; this is on account of reactions *r*_3_/*r*_4_, forming FA, being thermally-dependent, which was estimated through regression, as it is indicated in the Table 4.

This leaves the concentration of reactants as the most probable explanation for SCA/ESCA proportion; as SCA is more extensively formed at higher degradation temperatures, it in turn increases *r*_6_, yielding more ESCA, while *k*_6_ is larger to begin with. The dependence on the initial PEG to SAXA weight ratios within 30–50 °C is analogous; increasing PEG to SAXA would at all examined temperatures promote pH lowering (from about 5.6 to 5.5 at 30 °C or even lower, 5.4), partially due to the formation of FA through *r*_3_/*r*_4_. Finally, even at 50 °C, the formation of SCA maximum was not observed, the latter being expected at a notably longer time or higher temperature beyond degradation examined.

Considering the formylation reaction, it can be seen that SFA concentrations are increasing with temperature. While *r*_7_ is sensitive to temperature (high *E*_a,7_, Table 4), a bit closer inspection indicates that rather than a direct thermal dependence, pH will be decisive, as the variation from 30 to 40 °C is larger than to 50 °C ensuing. This above rationale may be followed through the dependence on the PEG to SAXA ratio at all examined temperatures. SFA will increase with PEG to SAXA due to two contributing factors, namely, by the indirect catalytic effect of H^+^, as well as FA acting as a reactant directly—as *r*_3_/*r*_4_ have notable activation energies (Table 4), they are increasing from 30 to 50 °C, producing F, FA, as well as H^+^, which promote formylation. A fast initial increase in SFA, which consequently levels off, may be ascribed to the dynamics of FA formation (and PEG degradation), which rises notably within the first 50-day period, whereas it basically remains equilibrated later on, as it is presented in the Figure 6e–f as well.

### 3.3. Additional Model Optimization

In this section, we address two main weaknesses of the proposed model. One is the inability to predict individual degradation product concentration for random desired set of initial conditions (PEG to SAXA ratio, time, temperature and RH), the other is moderate model description of SFA degradation kinetics. Namely, direct predictions of degradation products concentrations are not possible, since the described model includes a_w_ and pH measurements (as averages) in the Equations themselves. Instead, nominal, or measured at time 0, a_w_ values could be used, whereas pH, on the other hand, could be modeled. For this additional model optimization, we decided to test the conformance only for one temperature, 40 °C, and a nominal a_w_ of 10%, due to high complexity of the model. pH values were averaged for every PEG to SAXA ratio and plotted against PEG degradation products concentrations. A linear relationship is observed with FA, also possessing a high R^2^ of 0.995, providing us the possibility to simplify the model and predict pH solely upon FA concentration Equation (21).

(21) $$pH=-570.45\left(C_{FA}\right)+5.6703.$$

With regards to SFA kinetics, two main disagreements were identified; (1) experimental curve exhibits a plateau, whereas model does not; and (2) tablet composition factor is not weighed properly (model overestimates at low PEG to SAXA ratios and underestimates at high PEG to SAXA ratios). The first was addressed with the introduction of reversibility term and a newly established rate constant k_8_ Equation (22).

(22) $$r_{7}=k_{7}\left(c_{H^{+}}\right)c_{SAXA}c_{FA}-k_{8}c_{SFA}\approx {k_{7}}^{\prime }c_{H^{+}}c_{SAXA}c_{FA}-{k_{8}}^{\prime }c_{SFA}.$$

For the second, we envisioned that discrepancy originates already from PEG degradation, since similarly to SFA, FA is also overestimated at low PEG to SAXA ratios and underestimated at high PEG to SAXA ratios. Potential root cause in tablet composition, beside mere PEG to SAXA ratio, that could affect FA concentration, was investigated. Dependency on pH was considered; however, literature review does not support this, at least not in the pH ranges measured in tablets [5]. To our belief, much more relevant is PEG to HPMC weight ratio (Table 1), which has multiple effects on film-coat properties. Plasticizing effect of PEGs, aimed at reducing the film stiffness by weakening intermolecular forces and improving the molecular chain mobility, are well described in literature [40,41,42,43]. Authors have studied the relationship between PEG chain length and concentration on either compatibility or miscibility with film forming polymers, and their effect on film mechanical properties and water vapor transition rates. Very analogous to our study is the work of K. Waterman and his colleagues [11]. They suggest that if PEG is phase-compatible with a higher melting polymer, the molecular mobility of the PEG is reduced. This reduced mobility would lead to lower PEG degradation rates. This was investigated by examining the melt behavior of PEG in the presence of higher melting polymer. At a low *w/w* ratio, that is 10%, 0% of PEG was determined to be out of phase, at 20% ratio, approximately 5% and at 30% ratio, already approximately 22% is out of phase.

The same phenomenon was studied by Laboufie et al., who also chose to quantify the inclusion of PEG molecules into HPMC matrix by differential scanning calorimetry (DSC) measurements of melting enthalpies and compared them to theoretical ones, calculated through Equation (23) [42].

(23) $$∆H_{mix}=X_{COMP\;1}∆H_{COMP\;1}+X_{COMP\;2}∆H_{COMP\;2}.$$

**Figure 9:**
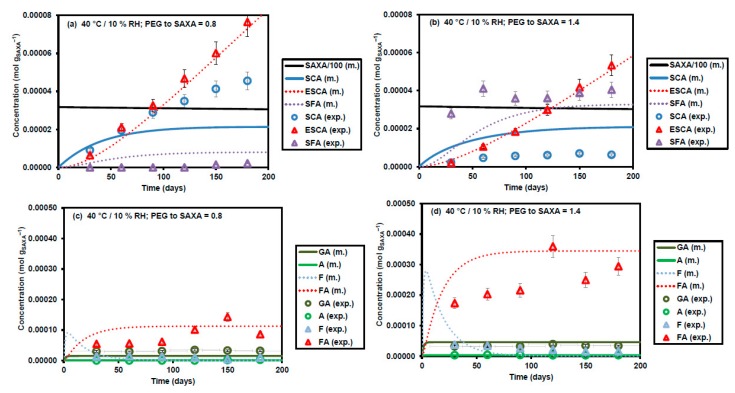
The updated model results for concentration of saxagliptin (SAXA) degradation products (amidine (SCA), epimer (ESCA), and formyl amide (SFA)) or polyethylene glycol polymer (PEG) degradation (glycolic acid (GA), acetaldehyde (A), formaldehyde (F), and formic acid (FA)). Lines represent fitted model (m.), markers experimental data points (exp.). Tablet degradation tests were executed at the temperature of 40 °C, the nominal water activity of 10% and the initial PEG to SAXA weight ratios of 0.8 (**a**,**c**) and 1.4 (**b**,**d**). A relative error of 10% is set based on sum of estimated analytical and sample variability.

Since HPMC does not have a melting event in the low temperature range, change in enthalpy is directly proportional to weight fraction of PEG, which can be implemented in the model, through X_PEG_, reactive PEG content, without increasing model complexity. With all three model modifications in place, we ran the regression again, and the changes applied resulted in an even better model fit (Figure 9).

### 3.4. Ab Initio Calculation of Ea

As aforementioned, it was initially hypothesized that only part of SAXA is reactive, and SFA is formed in preference to SCA and ESCA, due to lower Ea, until FA is consumed to a certain level. To evaluate our hypothesis, computational methods were employed. We expected calculations to show SFA reaction has a much lower Ea compared to SCA or ESCA.

For the formylation of SAXA by FA, three alternative reaction mechanisms were evaluated (Schemes S1–S3, Supporting Information). The transition structures for these different mechanisms in gas phase, water and formic acid were located using density functional theory (DFT) computations at the B3LYP/6-31G(d,p) level (Figure S1, Tables S22–S34, Supporting information). For first two proposed mechanisms, ΔG^‡^_298_ of 39.28 kcal/mol (164.35 kJ/mol) and 37.98 kcal/mol (158.70 kJ/mol) were determined for respective transition structures in gas phase (Table S18, Supporting information). Very good agreement between activation energy (E_a_) determined through kinetic model regression (151.6 kJ/mol) and ab initio calculated Gibbs energies of activation (ΔG^‡^) is observed. On the other hand, third reaction mechanism, where carboxyl group is activated by an acid [19], transition structure with ΔG^‡^_298_ values of −9.47, 18.47, and 18.39 kcal/mol in gas phase, water, and formic acid, were determined respectively (Table S19, Supporting Information). Such mechanism might not be relevant for the presented system since it requires extreme pH; however, it is in support of the assumption that formylation reaction is catalyzed by H^+^. Since intermediate formation is presumed for reaction to proceed, comparison to E_a_ determined through kinetic study is irrelevant.

For the intra-molecular cyclization of SAXA, it has been presented that it is catalyzed by hydroxyl group and self-catalysis (Schemes S4–S6, Supporting Information) [16], We located the transition structures of the cyclization of SAXA catalyzed by hydroxyl group containing molecules, such as water, FA, and PEG (using ethylene glycol as a simplified model) in gas phase and water (Figures S2 and S3, Tables S35–S45, Supporting information). The ΔG^‡^_298_ values for the hydroxyl catalyzed mechanisms are in range of 23 to 29 kcal/mol (96~121 kJ/mol) for gas phase (Table S20, Supporting information). Again, a very good agreement is observed with E_a_ determined through kinetic model regression (88.54 kJ/mol). As in previous studies, the auto-catalytic mechanism for the cyclization of SAXA was also explored. The simplified transition structure displays ΔG^‡^_298_ values of 9.90, 14.17, and 14.29 kcal/mol in gas phase, water, and formic acid, respectively (Table S21, Supporting information). Similarly, mechanism for FA catalyzed cyclization, in which a transition state with double proton transfer was explored resulted in ΔG^‡^_298_ values of 11.92, 17.58, and 17.52 kcal/mol in gas phase, water, and formic acid, respectively (Table S20, Supporting Information). Considering degradation products, SCA, ESCA, and FA, are observed at relatively low concentrations, these mechanisms are believed not to be relevant in our case.

### 3.5. Binary Component Study

**Figure 10:**
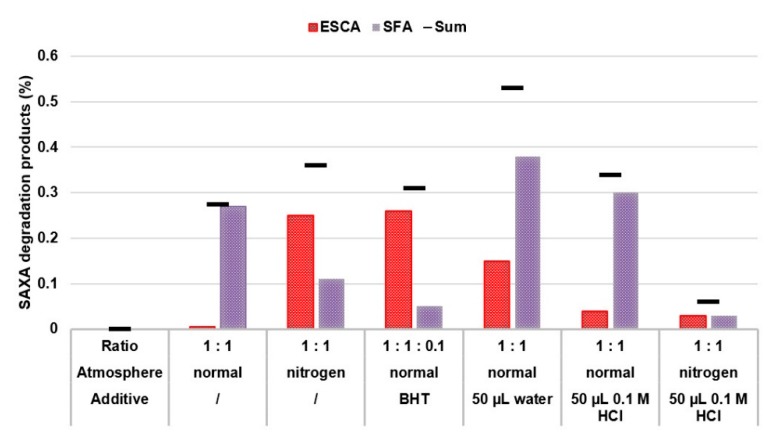
Binary mixtures study. Changes in atmospheric gas composition and additives are graphed. Individual saxagliptin (SAXA) degradation products and their sum are plotted on the y-axis (in % of SAXA). Drug substance to excipient weight ratio for each sample is given.

Kinetic studies, as the one we performed, are long-lasting and require large sets of data. During the formulation development, stability on simplified systems such as binary mixtures of drug substance and excipients are usually also evaluated, because they are easier to (re)produce and provide quick insights. It was our desire as well to emulate film-coated tablet behavior and compatibility of components on the level of binary mixtures, in order to confirm the proposed mechanism described by the model. SAXA and PEG 6000 ratio (1:1), temperature (60 °C), time (7 days), and container type (tightly sealed glass container) were kept constant for this study, namely to evaluate changes to atmospheric gas composition and additives (Figure 10). It was acknowledged that stress temperature had to be above T_m_ of PEG 6000 in order for PEG to degrade significantly. At temperatures of 50 °C and below, no SFA was determined for dry mixtures. Apparently, PEG in crystalline state is not reactive enough to produce significant FA amounts and has to be converted into melt, to best mimic the state in HPMC/PEG polymeric film [44]. SCA, precursor of ESCA, is not observed in any of the mixtures, owing to fast consecutive reaction.

When binary mixture of SAXA and PEG is flushed with nitrogen, ESCA forms predominantly; conversely, in the sample without nitrogen flushing, intra-molecular cyclization is completely inhibited and only SFA forms. As in film-coated tablets, sum of impurities is comparable in both samples, only slightly lower when at normal atmospheric conditions. Butylhydroxytoluene (BHT) addition, an antioxidant capable of radical scavenging, as expected, produces very similar impurity profile as nitrogen flushing. To reduce the amount of ESCA formed, the effect of acid addition (0.1 M HCl) was tested in normal and N_2_ flushed atmospheric conditions; same amount of H_2_O was added to represent a control. With this, we were able to simulate film- coated tablet conditions, since positive effect of acidification on SCA and ESCA formation is observed compared to control. Hence, conditions that provide a stable formulation, meeting also specification requirements for individual impurities, are identified.

## 4. Conclusions

This paper presents the development of a model, describing degradation kinetics of drug substance SAXA in active film-coat of a tablet. It demonstrates for the first time that modeling of complex degradation pathways, taking into account micro-environmental and formulation composition parameters, could be successfully accomplished and provides valuable insights into the origin of impurities formation as well as their interplay. Final developed model highlights the criticality of film-coat composition in the context of PEG to drug substance and PEG to film forming polymer (HPMC) ratio on drug substance degradation behavior.

PEG and SAXA degradation reaction equations were constructed based on the evaluation of existing pathways of PEG and SAXA degradation, as well as goodness of model fit. It was demonstrated that only part of PEG is reactive. Fraction of reactive PEG, which translates directly to its degradation rate, is a function of humidity, through Henry’s adsorption constant and water’s stoichiometric coefficient, and PEG to HPMC weight ratio. SAXA, on the other hand, is all reactive. Direction of SAXA degradation is conditioned by pH of the pharmaceutical formulation composition. A direct linear relationship between FA concentration, PEG’s main degradation product, and pH was identified. Intra-molecular cyclization reaction rate was shown to be dependent on concentration of SAXA and OH^−^, consecutive epimerization to ESCA on concentration of SCA and OH^−^, and parallel formylation to SFA on concentration of SAXA, FA and H^+^. Proposed reaction mechanisms are supported by very good agreement between activation energies (E_a_), determined through kinetic model regression, and ab initio calculated Gibbs energies of activation (ΔG^‡^).

We discovered that the use of higher PEG to SAXA and HPMC ratios in tablet film-coat reduces the levels of the highest forming degradation product ESCA, without affecting total sum of impurities. Moreover, at higher PEG to SAXA and HPMC ratios, SAXA is less susceptible to degradation at humid environment, which also results in satisfactory tablet appearance. These surprising findings are a consequence of pH lowering. Low molecular weight organic acids formed via PEG degradation, generally known to be problematic, especially for low dose drugs, in our case act in advantage of formulation’s stability. SAXA stabilization possibilities discussed in binary mixtures study, further confirm proposed mechanisms that were constructed for the active tablet surface and offer an approach to evaluate, in a short timeframe, whether similar behavior applies also to other molecules with related structural elements.

## Figures and Tables

**Table 1:** Comparison of formulation composition differences of film-coated tablets used in the stability study.

Formulation Composition.	1	2	3	4
Inert tablet core	200.0 mg	200.0 mg	200.0 mg	200.0 mg
SAXA in a form of HCl salt	2.5 mg 0.00317 mol/g_saxa_	2.5 mg 0.00317 mol/g_saxa_	2.5 mg 0.00317 mol/g_saxa_	2.5 mg 0.00317 mol/g_saxa_
HPMC 603	18.0 mg	17.5 mg	17.0 mg	16.5 mg
PEG 6000	2.0 mg 0.0182 mol/g_saxa_	2.5 mg 0.0227 mol/g_saxa_	3.0 mg 0.0272 mol/g_saxa_	3.5 mg 0.0318 mol/g_saxa_
Film-coated tablet (total)	222.5 mg	222.5 mg	222.5 mg	222.5 mg
PEG to SAXA ratio (*w*/*w*)	0.8	1.0	1.2	1.4
PEG to HPMC ratio (*w*/*w*)	0.111	0.143	0.176	0.212

Abbreviations: saxagliptin (SAXA), hypromellose (HPMC), polyethylene glycol (PEG), hydrochloride (HCl).

**Table 2:** Film-coated tablet stability study protocol. Each formulation, polyethylene glycol (PEG) to saxagliptin (SAXA) = 0.8, 1.0, 1.2, and 1.4, was exposed to four different temperature (T) conditions (30–60 °C) and three different nominal relative humidity (RH) conditions (10%, 30%, and 50%) for a prescribed number of days. X represents a sampling point.

T (°C) No. of Days	30 °C	40 °C	50 °C	60 °C
7	-	-	-	X
14	-	-	X	X
30	X	X	X	-
45	-	-	X	-
60	X	X	X	-
90	X	X	-	-
120	X	X	-	-
150	X	X	-	-
180	X	X	-	-

**Table 3:** Averaged results of pH measurements for different compositions at temperatures of 30 to 50 °C and nominal water activities 10% to 50%.

30 °C			40 °C			50 °C			PEG to SAXA Weight Ratio
10% RH	30% RH	50% RH	10% RH	30% RH	50% RH	10% RH	30% RH	50% RH	
5.68	5.70	5.65	5.63	5.63	5.58	5.63	5.62	5.57	0.8
5.66	5.63	5.61	5.63	5.60	5.56	5.62	5.59	5.55	1.0
5.60	5.49	5.61	5.53	5.51	5.56	5.53	5.51	5.55	1.2
5.48	5.40	5.27	5.43	5.40	5.25	5.45	5.37	5.23	1.4

Abbreviations: polyethylene glycol (PEG), saxagliptin (SAXA).

**Table 4:** The reaction (r_i_) rate constants (ki’) at 40 °C, pre-exponential factors (Ai) and activation energies (Ea,i) for tablet degradation tests, executed at the temperatures of 30 to 50 °C, the nominal water activities of 10% to 50%, and the initial polyethylene glycol (PEG) to saxagliptin (SAXA) weight ratios of 0.8 to 1.4.

r_i_	ki’(40 °C)	Ai	Ea,i (kJ mol^−1^)
r_1_	0.803 ± 0.003 d^−1^	0.803 ± 0.009 d^−1^	~0
r_2_	0.0246 ± 0.0002 d^−1^	0.0246 ± 0.0003 d^−1^	~0
r_3_	4.66 ± 0.01 d^−1^	(8.2 ± 0.2) × 103 d^−1^	19.46 ± 0.05
r_4_	0.06672 ± 0.00004 d^−1^	(7.3 ±0.3) × 1013 d^−1^	90.2 ± 0.1
r_5_	0.00025735 ± 0.00000008 L mol^−1^ d^−1^ *	(1.51 ±0.03) × 1011 L mol^−1^ d^−1^ *	88.54 ± 0.04
r_6_	0.09322 ± 0.00006 L mol^−1^ d^−1^ *	0.09322 ± 0.00006 L mol^−1^ d^−1^ *	~0
r_7_	0.1524 ± 0.0001 L g_saxa_ mol^−2^ d^−1^ *	(3.0 ± 0.3) × 1024 L g_saxa_ mol^−2^ d^−1^ *	151.6 ± 0.3

* c_H_^+^/c_OH_^−^ (mol L^−1^) were determined from pH by dissolving the tablets in water as described (15% m/V).

## Supplementary Materials

The following are available online at https://www.mdpi.com/1999-4923/11/9/452/s1, Table S1: Data for concentration of three main SAXA degradation products ESCA, SCA and SFA and their sum at temperature 60 °C, Table S2: Data for concentration of three main SAXA degradation products ESCA, SCA and SFA and their sum at temperature 50 °C, Table S3: Data for concentration of three main SAXA degradation products ESCA, SCA and SFA and their sum at temperature 40 °C, Table S4: Data for concentration of three main SAXA degradation products ESCA, SCA and SFA and their sum at temperature 30 °C, Table S5: Data for concentration of organic impurities A, F, GA and FA at temperature 60 °C, Table S6: Data for concentration of organic impurities A, F, GA and FA at temperature 50 °C, Table S7: Data for concentration of organic impurities A, F, GA and FA at temperature 40 °C, Table S8: Data for concentration of organic impurities A, F, GA and FA at temperature 30 °C, Table S9: Worksheet for statistical evaluation, Table S10: Factors, Table S11: Responses, Table S12: Coefficient list, Table S13: Correlation matrix, Table S14: Imported dataset for organic impurities, Table S15: Imported dataset for saxagliptin degradation products, Table S16: Modelled data for organic impurities, Table S17: Modelled data for saxagliptin degradation products, Table S18: Calculated energy for the species involved in the reaction 1 in gas phase and water, Table S19: Calculated energy for the species involved in the reaction 1 in gas phase, water and formic acid, Table S20: Calculated energy for the species involved in the parallel reaction 2 catalyzed by the hydroxyl group in gas phase, water and formic acid, Table S21: Calculated energy for the species involved in the parallel reaction 2 catalyzed by ESCA in gas phase, water and formic acid, Tables S22–S45: Coordinates for compounds used in DFT calculations, Figure S1: Structure of the transition state parallel reaction 1 in gas phase, Figure S2: Structure of the transition state parallel reaction 2 in gas phase catalyzed by the hydroxyl group, Figure S3: Structure of the transition state parallel reaction 2 in gas phase catalyzed by formic acid and ESCA, Scheme S1: Reaction mechanism 1 for parallel reaction 1, Scheme S2: Reaction mechanism 2 for parallel reaction 1, Scheme S3: Reaction mechanism 3 for parallel reaction 1, Scheme S4: Reaction mechanism 1 for for parallel reaction 2 catalyzed by hydroxyl group, Scheme S5: Reaction mechanism 2 for parallel reaction 2 catalyzed by the ESCA, Scheme S6: Reaction mechanism 3 for parallel reaction 2 catalyzed by formic acid.

## References

[1] Zong Z., Qiu J., Tinmanee R., Kirsch L.E. (2012). Kinetic model for solid-state degradation of gabapentin. J. Pharm. Sci..

[2] Long G.T., Vyazovkin S., Gamble N., Wight C.A. (2002). Hard to swallow dry: Kinetics and mechanism of the anhydrous thermal decomposition of acetylsalicylic acid. J. Pharm. Sci..

[3] Carvalho T.C., La Cruz T.E., Tábora J.E. (2017). A photochemical kinetic model for solid dosage forms. Eur. J. Pharm. Biopharm..

[4] Yoshioka S., Stella V.J. (2000). Stability of Drugs and Dosage Forms.

[5] Hemenway J.N., Carvalho T.C., Rao V.M., Wu Y., Levons J.K., Narang A.S., Paruchuri S.R., Stamato H.J., Varia S.A. (2012). Formation of reactive impurities in aqueous and neat polyethylene glycol 400 and effects of antioxidants and oxidation inducers. J. Pharm. Sci..

[6] Gullapalli R.P., Mazzitelli C.L. (2015). Polyethylene glycols in oral and parenteral formulations--A critical review. Int. J. Pharm..

[7] Zhang K., Pellett J.D., Narang A.S., Wang Y.J., Zhang Y.T. (2017). Reactive impurities in large and small molecule pharmaceutical excipients – A review. Trends Anal. Chem..

[8] Wu Y., Levons J., Narang A.S., Raghavan K., Rao V.M. (2011). Reactive Impurities in Excipients: Profiling, Identification and Mitigation of Drug–Excipient Incompatibility. AAPS Pharm. Sci. Tech..

[9] Wang G., Fiske J.D., Jennings S.P., Tomasella F.P., Palaniswamy V.A., Ray K.L. (2008). Identification and Control of a Degradation Product in Avapro™ Film-Coated Tablet: Low Dose Formulation. Pharm. Dev. Technol..

[10] Narang A.S., Yamniuk A., Zhang L., Comezoglu S.N., Bindra D.S., Varia S.A., Doyle M., Badawy S., Narang A.S., Boddu S.H.S. (2015). Drug Excipient Interactions. Excipient Applications in Formulation Design and Drug Delivery.

[11] Waterman K.C., Arikpo W.B., Fergione M.B., Graul T.W., Johnson B.A., Macdonald B.C., Roy M.C., Timpano R.J. (2008). N-methylation and N-formylation of a secondary amine drug (varenicline) in an osmotic tablet. J. Pharm. Sci..

[12] Baertschi S.W., Dill A.L., Kramer T.T., Scrivens G., Suruzhon M. (2019). Degradation Rate Observations as a Function of Drug Load in Solid-State Drug Products. J. Pharm. Sci..

[13] AstraZeneca Pharmaceuticals LP U.S. Food and Drug Administration Website. https://www.accessdata.fda.gov/drugsatfda_docs/label/2019/022350s023lbl.pdf..

[14] Bing V.L., Desai Divyakant S. (2011). Coated Tablet Formulation and Method. U.S. Patent.

[15] Chen W., Wang J., Desai D., Chang S.-Y., Kiang S., Lyngberg O., Reklaitis G.V., Seymour C., García-Munoz S. (2017). A Strategy for Tablet Active Film Coating Formulation Development Using a Content Uniformity Model and Quality by Design Principles. Comprehensive Quality by Design for Pharmaceutical Product Development and Manufacture.

[16] Jones G.S., Savage S.A., Ivy S., Benitez P.L., Ramirez A. (2011). Kinetic and Mechanistic Insight into the Thermodynamic Degradation of Saxagliptin. J. Org. Chem..

[17] Desai Divyakant S., Narang A., Rao Venkatramana M. (2014). Pharmaceutical Formulations Including an Amine Compound. U.S. Patent.

[18] Rao Venkatramana M., Narang A., Desai Divyakant S. (2013). Drug Formulations Using Water Soluble Antioxidants. U.S. Patent.

[19] Gerack C., McElwee-White L. (2014). Formylation of Amines. Molecules.

[20] Fontana A.J., Cundell A.M. (2009). Water Activity Applications in the Pharmaceutical Industry.

[21] Frisch M.J., Schlegel H.B., Scuseria G.E., Robb M.A., Cheeseman J.R., Scalmani G., Barone V., Mennucci B., Petersson G.A., Nakatsuji H. (2009). Gaussian 09 (Revision, E. 01).

[22] Tomasi J., Persico M. (1994). Molecular Interactions in Solution: An Overview of Methods Based on Continuous Distributions of the Solvent. Chem. Rev..

[23] Tomasi J., Bonaccorsi R., Cammi R., del Valle F.J.O. (1991). Theoretical chemistry in solution. Some results and perspectives of the continuum methods and in particular of the polarizable continuum model. J. Mol. Struct..

[24] Giroto J.A., Teixeira A.C.S.C., Nascimento C.A.O., Guardani R. (2010). Degradation of Poly(ethylene glycol) in Aqueous Solution by Photo-Fenton and H_2_O_2_/UV Processes. Ind. Eng. Chem. Res..

[25] Almkvist G., Persson I. (2008). Fenton-induced degradation of polyethylene glycol and oak holocellulose. A model experiment in comparison to changes observed in conserved waterlogged wood. Holzforschung.

[26] Glastrup J. (1996). Degradation of polyethylene glycol. A study of the reaction mechanism in a model molecule: Tetraethylene glycol. Polym. Degrad. Stab..

[27] Han S., Kim C., Kwon D. (1997). Thermal/oxidative degradation and stabilization of polyethylene glycol. Polymer.

[28] Fujita M., Ueda T., Handa T. (2009). Generation of formaldehyde by pharmaceutical excipients and its absorption by meglumine. Chem. Pharm. Bull..

[29] Fukuyama S., Kihara N., Nakashima K., Morokoshi N., Koda S., Yasuda T. (1994). Mechanism of Optical Isomerization of(S)-N-[1-(2-Fluorophenyl)-3,4,6,7-tetrahydro-4-oxopyrrolo[3,2,1-jk] [l,4]-benzodiazepine-3-y1]-1H-indole-2-carboxamide (FK480) in Soft Capsules Containing Polyethylene Glycol 400 and Glycerol. Pharm. Res..

[30] Jones G.S., Savage S.A., Ivy S., Benitez P.L., Ramirez A. (2013). Correction to Kinetic and Mechanistic Insight into the Thermodynamic Degradation of Saxagliptin. J. Org. Chem..

[31] Ramirez A., Truc V.C., Lawler M., Ye Y.K., Wang J., Wang C., Chen S., Laporte T., Liu N., Kolotuchin S. (2014). The Effect of Additives on the Zinc Carbenoid-Mediated Cyclopropanation of a Dihydropyrrole. J. Org. Chem..

[32] Santos M.S.d., Bernardino A.M.R., Souza M.C.d. (2006). Synthetic approaches to amidines. Química Nova.

[33] Steinreiber J., Faber K., Griengl H. (2008). De-racemization of Enantiomers versus De-epimerization of Diastereomers—Classification of Dynamic Kinetic Asymmetric Transformations (DYKAT). Chem. Eur. J..

[34] Wei D.H., Cui C.X., Qu Z.B., Zhu Y.Y., Tang M.S. (2010). A computational study on the reaction mechanisms of N-formylation of amines under a Lewis acid catalysis. J. Mol. Struct. THEOCHEM.

[35] Jung S.H., Ahn J.H., Park S.K., Choi J.K. (2002). A practical and convenient procedure for the N-formylation of amines using formic acid. Bull. Korean Chem. Soc..

[36] Linda P., Stener A., Cipiciani A., Savelli G. (1983). Hydrolysis of amides. Kinetics and mechanism of the basic hydrolysis of N-acylpyrroles, N-acylindoles and N-acylcarbazoles. J. Heterocycl. Chem..

[37] Hoaglund Hyzer C., Williamson M.L., Jansen P.E., Kopach M., Brian Scherer R., Baertschi S. (2017). Mechanistic Studies of the N-Formylation of Edivoxetine, a Secondary Amine-Containing Drug, in a Solid Oral Dosage Form. J. Pharm. Sci..

[38] Hemenway J.N., Carvalho T.C., Mantri R.V., Wu Y., Levons J.K., Narang A.S., Paruchuri S.R., Stamato H.J., Varia S.A., Narang A.S., Boddu S.H.S. (2015). Reactive Impurities in PEG: A Case Study. Excipient Applications in Formulation Design and Drug Delivery.

[39] Waterman K.C., Carella A.J., Gumkowski M.J., Lukulay P., MacDonald B.C., Roy M.C., Shamblin S.L. (2007). Improved protocol and data analysis for accelerated shelf-life estimation of solid dosage forms. Pharm. Res..

[40] Saringat H.B., Alfadol K.I., Khan G.M. (2005). The influence of different plasticizers on some physical and mechanical properties of hydroxypropyl methylcellulose free films. Pak. J. Pharm. Sci..

[41] Heinämäki J.T., Lehtola V.M., Nikupaavo P., Yliruusi J.K. (1994). The mechanical and moisture permeability properties of aqueous-based hydroxypropyl methylcellulose coating systems plasticized with polyethylene glycol. Int. J. Pharm..

[42] Laboulfie F., Hémati M., Lamure A., Diguet S. (2013). Effect of the plasticizer on permeability, mechanical resistance and thermal behaviour of composite coating films. Powder Technol..

[43] Jarray A., Gerbaud V., Hemati M. (2016). Polymer-plasticizer compatibility during coating formulation: A multi-scale investigation. Prog. Org. Coat..

[44] Wang X., Michoel A., Van den Mooter G. (2004). Study of the phase behavior of polyethylene glycol 6000–itraconazole solid dispersions using DSC. Int. J. Pharm..

