# Supplementary material for: Understanding and Kinetic Modeling of Complex Degradation Pathways in the Solid Dosage Form: The Case of Saxagliptin
Source: Pharmaceutics. 2019 Sep 2;11(9):452. doi: 10.3390/pharmaceutics11090452 (PMC6781548; doi:10.3390/pharmaceutics11090452)
Supplement: Supplementary file 1 [file pharmaceutics-11-00452-s001.pdf]

pharmaceutics

MDPI

# Supplementary Materials: Understanding and Kinetic Modeling of Complex Degradation Pathways in the Solid Dosage Form: The Case of Saxagliptin

**Blaž Robnik, Blaž Likozar, Baifan Wang, Tijana Stanić Ljubin and Zdenko Časar**

## 1. Studied Reaction Schemes and Mechanisms

Two parallel saxagliptin degradation reactions were evaluated: SFA formation (parallel reaction 1) and (E)SCA formation (parallel reaction 2).

### *1.1. SFA Formation (Parallel Reaction 1)*

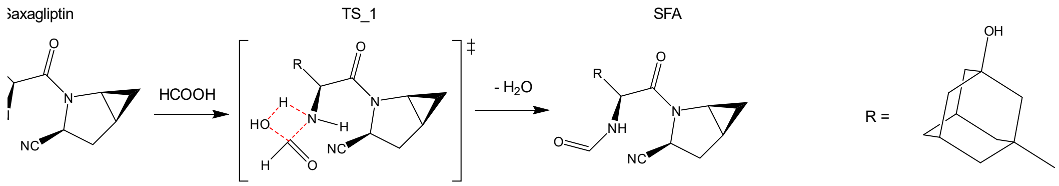


**Scheme S1.** Reaction mechanism 1 for parallel reaction 1.

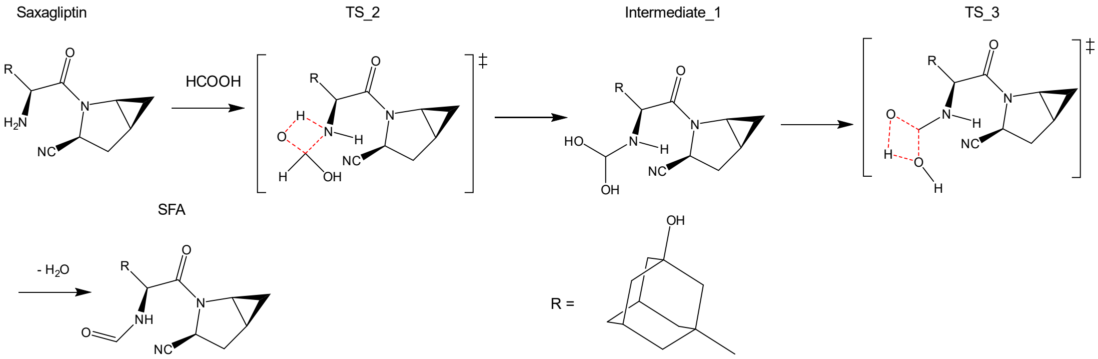


**Scheme S2.** Reaction mechanism 2 for parallel reaction 1.

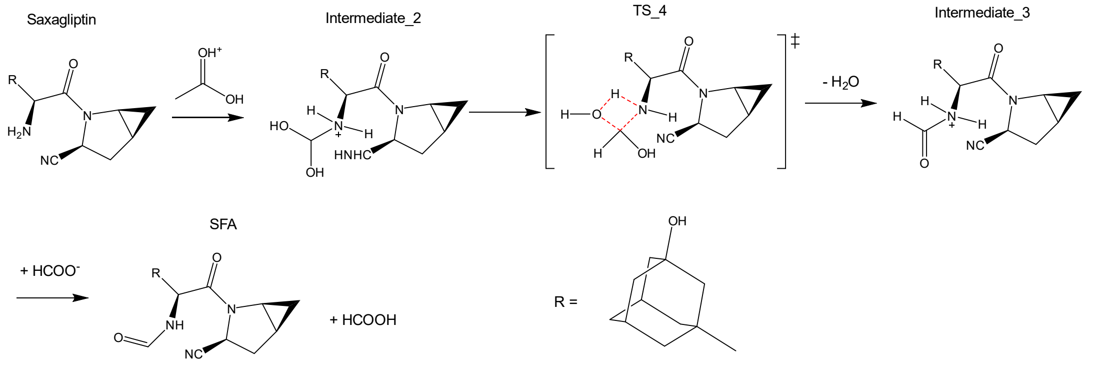


**Scheme S3. Reaction mechanism 3 for** parallel reaction 1 [1]

## *1.2. (E)SCA Formation (Parallel Reaction 2)*

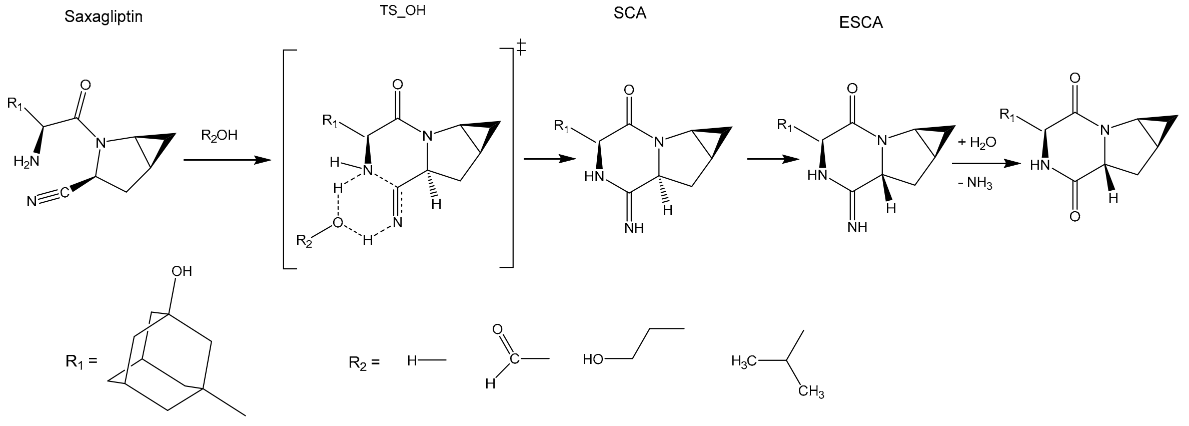


**Scheme S4.** Reaction mechanism 1 for for parallel reaction 2 catalyzed by hydroxyl group [2].

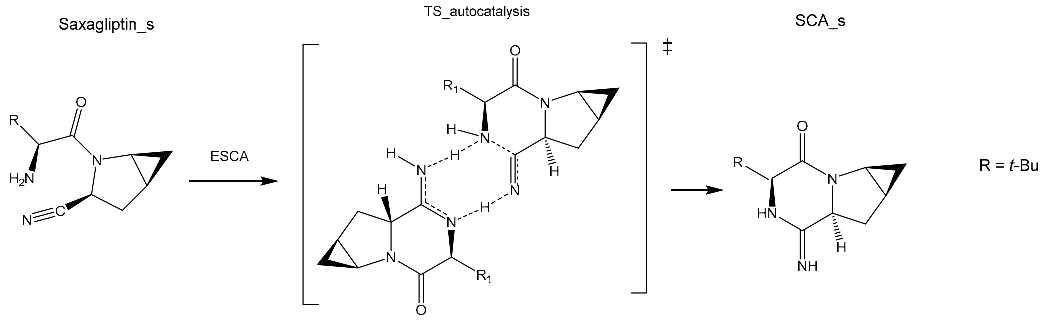


**Scheme S5.** Reaction mechanism 2 for parallel reaction 2 **ca**talyzed by the ESCA [2].

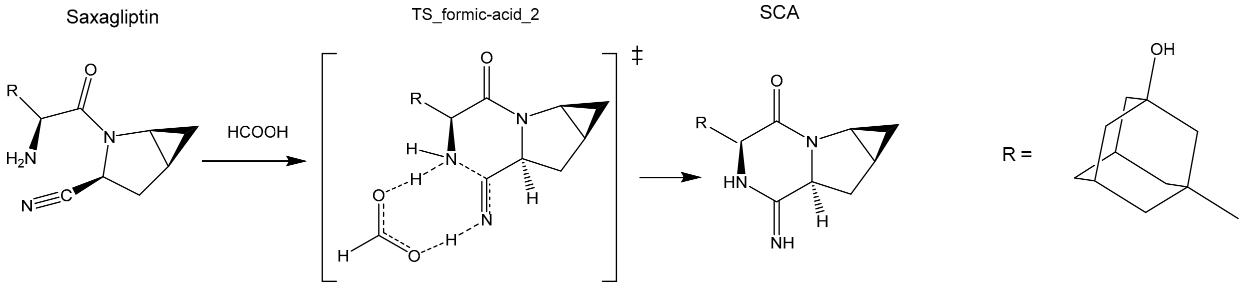


**Scheme S6. Reaction mechanism 3 for parallel reaction 2 c**atalyzed by formic acid.

## 2. Analytical Methods

### *2.1. Saxagliptin (SAXA) Related Substances and Degradation Products Determination*

SAXA was analyzed using the Acquity UPLC (Waters Corp., Milford, MA, USA), a reverse phase column, Luna C18(2)–HST, particle size 2.5 µm, dimensions 100 × 3.0 mm, from Phenomenex Inc. (Torrance, CA, USA) and a suitable UHPLC C18 3.0 mm pre–column at 25 °C. Gradient elution was used to achieve chromatographic separation with mobile phases A (buffer, pH 6) and B (buffer, pH 6 and acetonitrile, 50:50 (*v*/*v*)). The mobile phase in the gradient elution progress was: 0 min 6% B, 2 min 6% B, 11 min 50% B, 16–17.5 min 100% B and 18 min 6% B. The mobile phase flow was 0.7 mL/min. Auto–sampler temperature was set at 5 °C, the injection volume at 12 µL and run time 18 minutes. Data was recorded in photo diode array (PDA) mode and subsequently chromatograms were extracted at a wavelength of 213 nm. Solvent for sample preparation was 1.0 mL of 85% O–phosphoric acid in 1000 mL of additionally purified water. Tablet sample preparation; 2 tablets were transferred into 50 mL Erlenmeyer flask, 40 mL of solvent was added and sonicated until the tablets completely disintegrated. Dispersion was then centrifuged through Amicon Ultra–4, Ultracel® 3K ultrafilters for 15 minutes at 4000 rpm. Compounds of interest elute at 7.0 min (saxagliptin cyclic amidine epimer, ESCA), 7.7 min (saxagliptin cyclic amidine, SCA), 9.4 min (saxaglptin, SAXA) and 11.5 min (saxagliptin formyl amide, SFA). Results were first calculated as % of individual impurity against the declared dose of S (2.5 mg). For the purpose of model development, response factors for individual SAXA degradation products were applied and values were transformed into mol/$g_{saxa}$.

### *2.2. Low Molecular Weight Organic Impurities Determination*

Low molecular weight organic impurites were analyzed using the Acquity UPLC (Waters Corp., Milford, MA, USA), a reverse phase column Acq UPLC HSS C18, particle size 1.8 µm, dimensions 150 × 2.1 mm from Waters Corp. (USA) at 45 °C. Gradient elution was used to achieve chromatographic separation with mobile phases A (HCl solution, pH 2.5) and B (acetonitrile and MTBE, 98:2 (*v*/*v*)). The mobile phase in the gradient elution progress was: 0–1.2 min 5% B, 6 min 19% B, 10 min 50% B, 11.5 min 55% B, 13.5–14.5 min 90% B and 15 min 5% B with equilibration time of 4 minutes. The mobile phase flow was 0.5 mL/min, autosampler temperature at 5 °C and volume of injection 4 µL. Chromatograms were recorded at a wavelength of 400 nm. Derivatization of compounds was performed at the sample preparation phase. Derivatization reagent consisted of 14.4 mg/mL EDC, 2.31 mg/mL 2–NPH and 9 µL/mL of pyridine. Three film–coated tablets were transferred into analysis tube to which 4 mL of additionally purified water were added and sonicated until tablet disintegration. Suspension was transferred into Amicon Ultra–4, Ultracel® 3K ultrafilters and centrifuged at 5000 rpm for 1 hour. Derivatization was performed in 1.5 mL tubes in which 600 µL of sample filtrate, 200 µL of acetonitrile and 400 µL of derivatization reagent were mixed and shaken for 30 minutes at 750 rpm at 60 °C. Compounds of interest elute at 4.5 min (glycolic acid, GA), 5.3 min (formic acid, FA), 11.0 min (formaldehyde, F) and 11.9 min (acetaldehyde, A). Results are reported in ppm calculated against tablet film–coat mass. For the purpose of model development, values were transformed into mol/$g_{saxa}$.

# 3. Tables of Data

## *3.1. Saxagliptin (SAXA) Degradation Profile*

**Table S1.** Data for concentration of three main SAXA degradation products ESCA, SCA and SFA and their sum (% against S, *w/w*) at temperature 60 °C.

| | 60 °C | 60 °C | 60 °C | 60 °C | 60 °C | 60 °C | 60 °C | 60 °C | 60 °C | 60 °C | 60 °C | 60 °C |
|---|---|---|---|---|---|---|---|---|---|---|---|---|
| | **10 % RH** | **10 % RH** | **10 % RH** | **10 % RH** | **30 % RH** | **30 % RH** | **30 % RH** | **30 % RH** | **50 % RH** | **50 % RH** | **50 % RH** | **50 % RH** |
| | **ESCA** | **SCA** | **SFA** | **Sum** | **ESCA** | **SCA** | **SFA** | **Sum** | **ESCA** | **SCA** | **SFA** | **Sum** |
| **PEG/SAXA = 0.8** | | | | | | | | | | | | |
| 7 days | 0.96 | 0.59 | 0.05 | 1.60 | 1.80 | 0.34 | 0.07 | 2.21 | 3.10 | 0.14 | 0.15 | 3.40 |
| 14 days | 2.29 | 0.86 | 0.07 | 3.22 | 3.84 | 0.42 | 0.12 | 4.39 | 5.88 | 0.12 | 0.23 | 6.23 |
| **PEG/SAXA = 1.0** | | | | | | | | | | | | |
| 7 days | 0.78 | 0.41 | 0.23 | 1.42 | 1.87 | 0.24 | 0.27 | 2.38 | 3.49 | 0.10 | 0.22 | 3.80 |
| 14 days | 2.00 | 0.50 | 0.24 | 2.73 | 3.92 | 0.24 | 0.22 | 4.37 | 6.11 | 0.09 | 0.30 | 6.50 |
| **PEG/SAXA = 1.2** | | | | | | | | | | | | |
| 7 days | 0.63 | 0.19 | 0.65 | 1.47 | 1.74 | 0.11 | 0.67 | 2.53 | 2.38 | 0.02 | 0.32 | 2.72 |
| 14 days | 1.73 | 0.17 | 0.72 | 2.62 | 3.46 | 0.11 | 0.71 | 4.28 | 3.33 | 0.00 | 0.68 | 4.02 |
| **PEG/SAXA = 1.4** | | | | | | | | | | | | |
| 7 days | 0.46 | 0.06 | 1.36 | 1.89 | 0.95 | 0.02 | 1.87 | 2.84 | 0.80 | 0.00 | 2.82 | 3.62 |
| 14 days | 1.35 | 0.05 | 1.69 | 3.09 | 1.85 | 0.00 | 2.23 | 4.08 | 2.07 | 0.00 | 3.28 | 5.35 |

**Table S2.** Data for concentration of three main SAXA degradation products ESCA, SCA and SFA and their sum (% against SAXA, *w/w*) at temperature 50 °C.

| | 50 °C | 50 °C | 50 °C | 50 °C | 50 °C | 50 °C | 50 °C | 50 °C | 50 °C | 50 °C | 50 °C | 50 °C |
|---|---|---|---|---|---|---|---|---|---|---|---|---|
| | **10 % RH** | **10 % RH** | **10 % RH** | **10 % RH** | **30 % RH** | **30 % RH** | **30 % RH** | **30 % RH** | **50 % RH** | **50 % RH** | **50 % RH** | **50 % RH** |
| | **ESCA** | **SCA** | **SFA** | **Sum** | **ESCA** | **SCA** | **SFA** | **Sum** | **ESCA** | **SCA** | **SFA** | **Sum** |
| **PEG/SAXA = 0.8** | | | | | | | | | | | | |
| 14 days | 0.50 | 0.53 | 0.00 | 1.03 | 1.15 | 0.44 | 0.03 | 1.61 | 2.66 | 0.18 | 0.12 | 2.96 |
| 30 days | 1.33 | 0.82 | 0.00 | 2.15 | 2.63 | 0.56 | 0.07 | 3.27 | 4.21 | 0.24 | 0.16 | 4.62 |
| 45 days | 2.09 | 1.02 | 0.05 | 3.16 | 3.97 | 0.62 | 0.10 | 4.70 | 5.84 | 0.22 | 0.19 | 6.25 |
| 60 days | 3.73 | 1.31 | 0.08 | 5.12 | 6.35 | 0.83 | 0.14 | 7.31 | 9.64 | 0.27 | 0.36 | 10.28 |
| **PEG/SAXA = 1.0** | | | | | | | | | | | | |
| 14 days | 0.38 | 0.37 | 0.11 | 0.86 | 1.20 | 0.32 | 0.15 | 1.67 | 2.91 | 0.16 | 0.18 | 3.24 |
| 30 days | 1.08 | 0.60 | 0.17 | 1.85 | 2.75 | 0.39 | 0.22 | 3.36 | 4.86 | 0.15 | 0.28 | 5.29 |
| 45 days | 1.74 | 0.73 | 0.21 | 2.68 | 3.86 | 0.43 | 0.28 | 4.57 | 5.59 | 0.20 | 0.27 | 6.06 |
| 60 days | 3.28 | 0.96 | 0.28 | 4.52 | 6.59 | 0.47 | 0.40 | 7.46 | 9.87 | 0.20 | 0.47 | 10.54 |

**Table S2.** *Cont.*

| | 50 °C | 50 °C | 50 °C | 50 °C | 50 °C | 50 °C | 50 °C | 50 °C | 50 °C | 50 °C | 50 °C | 50 °C |
|---|---|---|---|---|---|---|---|---|---|---|---|---|
| | 10 % RH | 10 % RH | 10 % RH | 10 % RH | 30 % RH | 30 % RH | 30 % RH | 30 % RH | 50 % RH | 50 % RH | 50 % RH | 50 % RH |
| | ESCA | SCA | SFA | Sum | ESCA | SCA | SFA | Sum | ESCA | SCA | SFA | Sum |
| **PEG/SAXA = 1.2** | | | | | | | | | | | | |
| 14 days | 0.27 | 0.19 | 0.60 | 1.06 | 1.11 | 0.16 | 0.58 | 1.85 | 2.24 | 0.04 | 0.25 | 2.53 |
| 30 days | 0.87 | 0.27 | 0.71 | 1.86 | 2.42 | 0.16 | 0.69 | 3.27 | 3.46 | 0.03 | 0.38 | 3.87 |
| 45 days | 1.60 | 0.29 | 0.77 | 2.66 | 3.74 | 0.16 | 0.82 | 4.72 | 5.36 | 0.07 | 0.41 | 5.84 |
| 60 days | 2.76 | 0.30 | 1.05 | 4.12 | 6.21 | 0.19 | 1.09 | 7.49 | 8.30 | 0.06 | 0.57 | 8.93 |
| **PEG/SAXA = 1.4** | | | | | | | | | | | | |
| 14 days | 0.24 | 0.10 | 1.29 | 1.63 | 0.72 | 0.04 | 1.64 | 2.40 | 0.49 | 0.00 | 2.42 | 2.91 |
| 30 days | 0.85 | 0.10 | 1.38 | 2.33 | 1.74 | 0.02 | 1.84 | 3.60 | 1.09 | 0.00 | 2.76 | 3.84 |
| 45 days | 1.38 | 0.09 | 1.43 | 2.90 | 2.33 | 0.00 | 2.14 | 4.47 | 1.51 | 0.00 | 2.99 | 4.50 |
| 60 days | 2.57 | 0.09 | 1.99 | 4.64 | 3.82 | 0.00 | 2.79 | 6.62 | 2.72 | 0.00 | 3.94 | 6.66 |

**Table S3.** Data for concentration of three main SAXA degradation products ESCA, SCA and SFA and their sum (% against SAXA, *w*/*w*) at temperature 40 °C.

| | 40 °C | 40 °C | 40 °C | 40 °C | 40 °C | 40 °C | 40 °C | 40 °C | 40 °C | 40 °C | 40 °C | 40 °C |
|---|---|---|---|---|---|---|---|---|---|---|---|---|
| | 10 % RH | 10 % RH | 10 % RH | 10 % RH | 30 % RH | 30 % RH | 30 % RH | 30 % RH | 50 % RH | 50 % RH | 50 % RH | 50 % RH |
| | ESCA | SCA | SFA | Sum | ESCA | SCA | SFA | Sum | ESCA | SCA | SFA | Sum |
| **PEG/SAXA = 0.8** | | | | | | | | | | | | |
| 30 days | 0.20 | 0.29 | 0.00 | 0.49 | 0.49 | 0.32 | 0.00 | 0.81 | 1.70 | 0.20 | 0.05 | 1.95 |
| 60 days | 0.66 | 0.61 | 0.00 | 1.28 | 1.51 | 0.61 | 0.05 | 2.16 | 4.06 | 0.28 | 0.13 | 4.47 |
| 90 days | 1.03 | 0.91 | 0.00 | 1.94 | 2.16 | 0.81 | 0.04 | 3.01 | 5.06 | 0.31 | 0.17 | 5.54 |
| 120 days | 1.47 | 1.10 | 0.00 | 2.57 | 3.02 | 0.93 | 0.06 | 4.01 | 6.00 | 0.34 | 0.17 | 6.51 |
| 150 days | 1.90 | 1.30 | 0.05 | 3.25 | 3.66 | 1.05 | 0.07 | 4.78 | 5.16 | 0.72 | 0.11 | 5.98 |
| 180 days | 2.41 | 1.43 | 0.07 | 3.92 | 4.72 | 1.21 | 0.11 | 6.04 | 7.63 | 0.49 | 0.20 | 8.32 |
| **PEG/SAXA = 1.0** | | | | | | | | | | | | |
| 30 days | 0.16 | 0.21 | 0.06 | 0.44 | 0.47 | 0.26 | 0.09 | 0.81 | 1.87 | 0.17 | 0.08 | 2.12 |
| 60 days | 0.56 | 0.44 | 0.08 | 1.08 | 1.29 | 0.42 | 0.24 | 1.96 | 4.34 | 0.21 | 0.18 | 4.74 |
| 90 days | 0.84 | 0.69 | 0.07 | 1.60 | 2.24 | 0.61 | 0.11 | 2.96 | 4.87 | 0.27 | 0.21 | 5.35 |
| 120 days | 1.18 | 0.81 | 0.08 | 2.07 | 2.65 | 0.56 | 0.23 | 3.44 | 5.64 | 0.31 | 0.21 | 6.16 |
| 150 days | 1.62 | 1.00 | 0.11 | 2.72 | 3.68 | 0.74 | 0.13 | 4.55 | 7.53 | 0.30 | 0.26 | 8.09 |
| 180 days | 2.08 | 1.14 | 0.15 | 3.37 | 3.88 | 0.74 | 0.35 | 4.97 | 9.18 | 0.28 | 0.35 | 9.81 |

**Table S3.** *Cont.*

| | 40 °C | 40 °C | 40 °C | 40 °C | 40 °C | 40 °C | 40 °C | 40 °C | 40 °C | 40 °C | 40 °C | 40 °C |
|---|---|---|---|---|---|---|---|---|---|---|---|---|
| | 10 % RH | 10 % RH | 10 % RH | 10 % RH | 30 % RH | 30 % RH | 30 % RH | 30 % RH | 50 % RH | 50 % RH | 50 % RH | 50 % RH |
| | ESCA | SCA | SFA | Sum | ESCA | SCA | SFA | Sum | ESCA | SCA | SFA | Sum |
| **PEG/SAXA = 1.2** | | | | | | | | | | | | |
| 30 days | 0.07 | 0.08 | 0.48 | 0.63 | 0.36 | 0.14 | 0.48 | 0.98 | 1.80 | 0.10 | 0.12 | 2.02 |
| 60 days | 0.25 | 0.22 | 0.68 | 1.16 | 1.26 | 0.23 | 0.66 | 2.15 | 3.67 | 0.10 | 0.25 | 4.02 |
| 90 days | 0.45 | 0.33 | 0.58 | 1.35 | 1.78 | 0.25 | 0.55 | 2.58 | 4.90 | 0.11 | 0.25 | 5.27 |
| 120 days | 0.77 | 0.39 | 0.55 | 1.71 | 2.56 | 0.27 | 0.74 | 3.57 | 5.57 | 0.09 | 0.26 | 5.92 |
| 150 days | 1.12 | 0.49 | 0.62 | 2.23 | 3.25 | 0.29 | 0.74 | 4.28 | 6.27 | 0.08 | 0.41 | 6.76 |
| 180 days | 1.41 | 0.52 | 0.74 | 2.67 | 4.38 | 0.31 | 0.82 | 5.51 | 7.84 | 0.11 | 0.44 | 8.38 |
| **PEG/SAXA = 1.4** | | | | | | | | | | | | |
| 30 days | 0.06 | 0.07 | 0.96 | 1.09 | 0.34 | 0.06 | 1.10 | 1.51 | 0.30 | 0.00 | 1.95 | 2.25 |
| 60 days | 0.33 | 0.15 | 1.41 | 1.89 | 1.09 | 0.06 | 1.56 | 2.72 | 0.72 | 0.00 | 2.72 | 3.44 |
| 90 days | 0.58 | 0.18 | 1.24 | 1.99 | 1.73 | 0.06 | 1.34 | 3.13 | 1.26 | 0.00 | 2.37 | 3.63 |
| 120 days | 0.94 | 0.19 | 1.24 | 2.37 | 2.30 | 0.06 | 1.50 | 3.85 | 1.43 | 0.00 | 2.58 | 4.01 |
| 150 days | 1.32 | 0.22 | 1.33 | 2.87 | 2.88 | 0.08 | 1.59 | 4.55 | 1.92 | 0.00 | 2.87 | 4.79 |
| 180 days | 1.68 | 0.20 | 1.39 | 3.27 | 3.73 | 0.07 | 1.66 | 5.46 | 2.25 | 0.00 | 2.77 | 5.02 |

**Table S4.** Data for concentration of three main SAXA degradation products ESCA, SCA and SFA and their sum (% against SAXA, *w/w*) at temperature 30 °C (continues on next page).

| | 30 °C | 30 °C | 30 °C | 30 °C | 30 °C | 30 °C | 30 °C | 30 °C | 30 °C | 30 °C | 30 °C | 30 °C |
|---|---|---|---|---|---|---|---|---|---|---|---|---|
| | 10 % RH | 10 % RH | 10 % RH | 10 % RH | 30 % RH | 30 % RH | 30 % RH | 30 % RH | 50 % RH | 50 % RH | 50 % RH | 50 % RH |
| | ESCA | SCA | SFA | Sum | ESCA | SCA | SFA | Sum | ESCA | SCA | SFA | Sum |
| **PEG/SAXA = 0.8** | | | | | | | | | | | | |
| 30 days | 0.04 | 0.07 | 0.00 | 0.11 | 0.07 | 0.10 | 0.00 | 0.17 | 0.31 | 0.13 | 0.00 | 0.44 |
| 60 days | 0.11 | 0.16 | 0.00 | 0.27 | 0.23 | 0.21 | 0.00 | 0.43 | 0.93 | 0.23 | 0.00 | 1.17 |
| 90 days | 0.17 | 0.27 | 0.00 | 0.43 | 0.35 | 0.33 | 0.00 | 0.68 | 1.36 | 0.30 | 0.00 | 1.67 |
| 120 days | 0.21 | 0.34 | 0.00 | 0.55 | 0.48 | 0.39 | 0.00 | 0.86 | 1.57 | 0.35 | 0.00 | 1.92 |
| 150 days* | – | – | – | – | 0.68 | 0.53 | 0.00 | 1.21 | 2.22 | 0.41 | 0.05 | 2.68 |
| 180 days* | 0.39 | 0.54 | 0.00 | 0.93 | – | – | – | – | – | – | – | – |

**Table S4.** *Cont.*

| | 30 °C<br>10 % RH<br>ESCA | 30 °C<br>10 % RH<br>SCA | 30 °C<br>10 % RH<br>SFA | 30 °C<br>10 % RH<br>Sum | 30 °C<br>30 % RH<br>ESCA | 30 °C<br>30 % RH<br>SCA | 30 °C<br>30 % RH<br>SFA | 30 °C<br>30 % RH<br>Sum | 30 °C<br>50 % RH<br>ESCA | 30 °C<br>50 % RH<br>SCA | 30 °C<br>50 % RH<br>SFA | 30 °C<br>50 % RH<br>Sum |
|---|---|---|---|---|---|---|---|---|---|---|---|---|
| **PEG/SAXA = 1.0** | | | | | | | | | | | | |
| 30 days | 0.04 | 0.06 | 0.00 | 0.10 | 0.07 | 0.08 | 0.05 | 0.20 | 0.48 | 0.13 | 0.00 | 0.61 |
| 60 days | 0.10 | 0.14 | 0.00 | 0.24 | 0.23 | 0.18 | 0.00 | 0.41 | 1.02 | 0.20 | 0.05 | 1.27 |
| 120 days | 0.20 | 0.28 | 0.00 | 0.48 | 0.40 | 0.26 | 0.08 | 0.73 | 1.89 | 0.27 | 0.06 | 2.23 |
| 150 days* | 0.27 | 0.35 | 0.04 | 0.66 | – | – | – | – | – | – | – | – |
| 180 days* | – | – | – | – | 0.61 | 0.36 | 0.17 | 1.15 | 2.80 | 0.32 | 0.11 | 3.24 |
| **PEG/SAXA = 1.2** | | | | | | | | | | | | |
| 30 days | 0.03 | 0.04 | 0.12 | 0.18 | 0.04 | 0.03 | 0.49 | 0.56 | 0.51 | 0.09 | 0.06 | 0.65 |
| 60 days | 0.05 | 0.06 | 0.26 | 0.38 | 0.09 | 0.05 | 0.76 | 0.91 | 0.99 | 0.15 | 0.09 | 1.23 |
| 90 days | 0.06 | 0.09 | 0.24 | 0.39 | 0.16 | 0.10 | 0.52 | 0.78 | 1.60 | 0.13 | 0.12 | 1.85 |
| 120 days | 0.08 | 0.11 | 0.27 | 0.45 | 0.20 | 0.11 | 0.55 | 0.86 | 1.49 | 0.18 | 0.11 | 1.79 |
| 150 days* | – | – | – | – | 0.31 | 0.17 | 0.57 | 1.05 | 2.28 | 0.18 | 0.12 | 2.59 |
| 180 days* | 0.13 | 0.17 | 0.34 | 0.63 | – | – | – | – | – | – | – | – |
| **PEG/SAXA = 1.4** | | | | | | | | | | | | |
| 30 days | 0.00 | 0.00 | 0.56 | 0.56 | 0.04 | 0.03 | 0.80 | 0.87 | 0.13 | 0.00 | 1.15 | 1.28 |
| 60 days | 0.03 | 0.03 | 0.93 | 0.99 | 0.10 | 0.06 | 1.10 | 1.26 | 0.24 | 0.00 | 1.86 | 2.11 |
| 90 days | 0.04 | 0.05 | 0.87 | 0.97 | 0.19 | 0.08 | 0.89 | 1.17 | 0.33 | 0.00 | 1.79 | 2.12 |
| 120 days | 0.05 | 0.07 | 0.75 | 0.86 | 0.27 | 0.09 | 0.97 | 1.33 | 0.46 | 0.00 | 1.70 | 2.16 |
| 150 days* | 0.07 | 0.10 | 0.89 | 1.06 | – | – | – | – | – | – | – | – |
| 180 days* | – | – | – | – | 0.56 | 0.12 | 1.08 | 1.76 | 0.72 | 0.00 | 1.99 | 2.71 |

* some results for 150 and 180 day time point are not available due to experimental issues.

## *3.2. PEG Degradation Profile (Low Molecular Weight or Ganic Impurities)*

**Table S5.** Data for concentration of organic impurities A, F, GA and FA (ppm against tablet film−coat, *w*/*w*) at temperature 60 °C.

| | 60 °C | 60 °C | 60 °C | 60 °C | 60 °C | 60 °C | 60 °C | 60 °C | 60 °C | 60 °C | 60 °C | 60 °C |
|---|---|---|---|---|---|---|---|---|---|---|---|---|
| | 10 % RH | 10 % RH | 10 % RH | 10 % RH | 30 % RH | 30 % RH | 30 % RH | 30 % RH | 50 % RH | 50 % RH | 50 % RH | 50 % RH |
| | A | F | GA | FA | A | F | GA | FA | A | F | GA | FA |
| **PEG/SAXA = 0.8** | | | | | | | | | | | | |
| 7 days | 0 | 4 | 234 | 203 | 0 | 2 | 240 | 233 | 0 | 0 | 250 | 292 |
| 14 days | 0 | 3 | 231 | 243 | 0 | 2 | 246 | 289 | 0 | 0 | 272 | 409 |
| **PEG/SAXA = 1.0** | | | | | | | | | | | | |
| 7 days | 0 | 11 | 228 | 392 | 0 | 2 | 225 | 457 | 0 | 0 | 249 | 323 |
| 14 days | 0 | 5 | 224 | 482 | 0 | 3 | 242 | 502 | 0 | 0 | 264 | 440 |
| **PEG/SAXA = 1.2** | | | | | | | | | | | | |
| 7 days | 9 | 19 | 225 | 756 | 0 | 3 | 228 | 924 | 0 | 1 | 258 | 391 |
| 14 days | 0 | 6 | 237 | 977 | 0 | 1 | 254 | 1070 | 0 | 2 | 283 | 803 |
| **PEG/SAXA = 1.4** | | | | | | | | | | | | |
| 7 days | 15 | 69 | 250 | 1251 | 10 | 19 | 221 | 2038 | 31 | 230 | 238 | 3318 |
| 14 days | 7 | 16 | 242 | 1464 | 0 | 6 | 242 | 2202 | 10 | 31 | 276 | 3268 |

**Table S6.** Data for concentration of organic impurities A, F, GA and FA (ppm against tablet film−coat, *w*/*w*) at temperature 50 °C.

| | **50 °C** | **50 °C** | **50 °C** | **50 °C** | **50 °C** | **50 °C** | **50 °C** | **50 °C** | **50 °C** | **50 °C** | **50 °C** | **50 °C** |
|---|---|---|---|---|---|---|---|---|---|---|---|---|
| | **10 % RH** | **10 % RH** | **10 % RH** | **10 % RH** | **30 % RH** | **30 % RH** | **30 % RH** | **30 % RH** | **50 % RH** | **50 % RH** | **50 % RH** | **50 % RH** |
| | **A** | **F** | **GA** | **FA** | **A** | **F** | **GA** | **FA** | **A** | **F** | **GA** | **FA** |
| **PEG/SAXA = 0.8** | | | | | | | | | | | | |
| 14 days | 0 | 8 | 230 | 202 | 0 | 3 | 237 | 220 | 0 | 1 | 236 | 253 |
| 30 days | 0 | 28 | 248 | 249 | 0 | 27 | 253 | 286 | 0 | 34 | 268 | 364 |
| 45 days | 0 | 28 | 250 | 275 | 0 | 30 | 259 | 328 | 0 | 35 | 277 | 417 |
| 60 days | 0 | 45 | 331 | 2780 | 0 | 40 | 299 | 480 | 0 | 38 | 291 | 548 |
| **PEG/SAXA = 1.0** | | | | | | | | | | | | |
| 14 days | 0 | 27 | 236 | 304 | 0 | 5 | 230 | 373 | 0 | 2 | 230 | 269 |
| 30 days | 0 | 36 | 254 | 409 | 0 | 29 | 259 | 488 | 0 | 38 | 269 | 408 |
| 45 days | 0 | 34 | 258 | 457 | 0 | 27 | 262 | 512 | 0 | 34 | 277 | 445 |
| 60 days | 22 | 82 | 441 | 4163 | 0 | 30 | 250 | 433 | 0 | 26 | 257 | 550 |
| **PEG/SAXA = 1.2** | | | | | | | | | | | | |
| 14 days | 9 | 50 | 241 | 763 | 0 | 10 | 229 | 891 | 0 | 2 | 247 | 342 |
| 30 days | 0 | 49 | 268 | 1141 | 0 | 29 | 281 | 1314 | 0 | 40 | 291 | 504 |
| 45 days | 0 | 36 | 268 | 1192 | 0 | 30 | 282 | 1274 | 0 | 46 | 283 | 526 |
| 60 days | 0 | 30 | 260 | 342 | 0 | 28 | 295 | 1399 | 0 | 39 | 306 | 625 |
| **PEG/SAXA = 1.4** | | | | | | | | | | | | |
| 14 days | 13 | 97 | 236 | 1154 | 10 | 52 | 217 | 1863 | 28 | 466 | 213 | 3197 |
| 30 days | 0 | 55 | 296 | 1773 | 0 | 40 | 310 | 2628 | 25 | 209 | 394 | 4285 |
| 45 days | 13 | 48 | 294 | 1851 | 0 | 40 | 301 | 2678 | 22 | 136 | 392 | 4097 |
| 60 days | 0 | 38 | 284 | 1739 | 0 | 35 | 269 | 1258 | 0 | 30 | 252 | 295 |

**Table S7.** Data for concentration of organic impurities A, F, GA and FA (ppm against tablet film−coat, *w*/*w*) at temperature 40 °C.

| | 40 °C | 40 °C | 40 °C | 40 °C | 40 °C | 40 °C | 40 °C | 40 °C | 40 °C | 40 °C | 40 °C | 40 °C |
|---|---|---|---|---|---|---|---|---|---|---|---|---|
| | 10 % RH | 10 % RH | 10 % RH | 10 % RH | 30 % RH | 30 % RH | 30 % RH | 30 % RH | 50 % RH | 50 % RH | 50 % RH | 50 % RH |
| | A | F | GA | FA | A | F | GA | FA | A | F | GA | FA |
| **PEG/SAXA = 0.8** | | | | | | | | | | | | |
| 30 days | 0 | 28 | 238 | 220 | 0 | 26 | 248 | 225 | 0 | 26 | 252 | 247 |
| 60 days | 0 | 34 | 239 | 224 | 0 | 30 | 246 | 226 | 0 | 31 | 255 | 295 |
| 90 days | 0 | 31 | 236 | 227 | 0 | 24 | 256 | 265 | 0 | 28 | 256 | 313 |
| 120 days | 0 | 18 | 245 | 321 | 0 | 14 | 252 | 346 | 0 | 12 | 250 | 427 |
| 150 days | 11 | 19 | 256 | 301 | 8 | 12 | 261 | 300 | 6 | 8 | 280 | 476 |
| 180 days | 10 | 27 | 254 | 312 | 9 | 15 | 259 | 332 | 0 | 15 | 275 | 441 |
| **PEG/SAXA = 1.0** | | | | | | | | | | | | |
| 30 days | 0 | 43 | 249 | 274 | 0 | 38 | 254 | 386 | 0 | 24 | 255 | 259 |
| 60 days | 0 | 46 | 241 | 282 | 0 | 44 | 255 | 564 | 0 | 27 | 256 | 328 |
| 90 days | 0 | 43 | 253 | 309 | 0 | 29 | 244 | 453 | 0 | 25 | 252 | 356 |
| 120 days | 0 | 32 | 285 | 506 | 0 | 15 | 291 | 624 | 0 | 12 | 291 | 606 |
| 150 days | 8 | 15 | 270 | 716 | 12 | 31 | 268 | 363 | 7 | 10 | 273 | 342 |
| 180 days | 11 | 32 | 261 | 431 | 0 | 20 | 272 | 928 | 9 | 18 | 288 | 571 |
| **PEG/SAXA = 1.2** | | | | | | | | | | | | |
| 30 days | 13 | 123 | 264 | 868 | 0 | 63 | 257 | 1143 | 0 | 22 | 248 | 301 |
| 60 days | 18 | 110 | 265 | 1015 | 0 | 44 | 252 | 1153 | 0 | 28 | 266 | 410 |
| 90 days | 16 | 85 | 265 | 1079 | 0 | 40 | 272 | 1456 | 0 | 29 | 275 | 460 |
| 120 days | 12 | 70 | 323 | 1800 | 0 | 18 | 325 | 1924 | 0 | 13 | 299 | 715 |
| 150 days | 13 | 52 | 285 | 1248 | 8 | 17 | 291 | 1529 | 7 | 10 | 292 | 561 |
| 180 days | 15 | 54 | 276 | 1473 | 10 | 24 | 291 | 1577 | 0 | 17 | 289 | 618 |
| **PEG/SAXA = 1.4** | | | | | | | | | | | | |
| 30 days | 16 | 149 | 291 | 1437 | 14 | 107 | 282 | 2216 | 36 | 518 | 313 | 4218 |
| 60 days | 17 | 111 | 295 | 1738 | 14 | 59 | 287 | 2372 | 25 | 309 | 296 | 4132 |
| 90 days | 16 | 87 | 297 | 1848 | 0 | 40 | 287 | 2304 | 30 | 276 | 347 | 4007 |
| 120 days | 17 | 71 | 382 | 2900 | 0 | 25 | 430 | 3796 | 21 | 149 | 553 | 6105 |
| 150 days | 12 | 45 | 294 | 2115 | 10 | 28 | 317 | 2560 | 17 | 129 | 390 | 4204 |
| 180 days | 11 | 44 | 324 | 2523 | 0 | 24 | 325 | 2771 | 14 | 94 | 472 | 4498 |

**Table S8.** Data for concentration of organic impurities A, F, GA and FA (ppm against tablet film−coat, *w/w*) at temperature 30 °C.

| | 30 °C | 30 °C | 30 °C | 30 °C | 30 °C | 30 °C | 30 °C | 30 °C | 30 °C | 30 °C | 30 °C | 30 °C |
|---|---|---|---|---|---|---|---|---|---|---|---|---|
| | 10 % RH | 10 % RH | 10 % RH | 10 % RH | 30 % RH | 30 % RH | 30 % RH | 30 % RH | 50 % RH | 50 % RH | 50 % RH | 50 % RH |
| | A | F | GA | FA | A | F | GA | FA | A | F | GA | FA |
| **PEG/SAXA = 0.8** | | | | | | | | | | | | |
| 30 days | 0 | 27 | 242 | 211 | 0 | 27 | 245 | 215 | 0 | 25 | 241 | 212 |
| 60 days | 0 | 34 | 236 | 205 | 0 | 31 | 237 | 210 | 0 | 26 | 238 | 202 |
| 90 days | 0 | 29 | 239 | 212 | 0 | 28 | 240 | 209 | 0 | 25 | 238 | 215 |
| 120 days | 0 | 15 | 244 | 255 | 0 | 13 | 228 | 285 | 0 | 10 | 234 | 300 |
| 150 days* | − | − | − | − | 9 | 15 | 248 | 231 | 7 | 10 | 263 | 257 |
| 180 days* | 0 | 20 | 237 | 237 | − | − | − | − | − | − | − | − |
| **PEG/SAXA = 1.0** | | | | | | | | | | | | |
| 30 days | 0 | 36 | 241 | 216 | 0 | 57 | 241 | 294 | 0 | 22 | 242 | 210 |
| 60 days | 0 | 47 | 242 | 229 | 0 | 42 | 240 | 245 | 0 | 23 | 244 | 228 |
| 90 days | 0 | 42 | 236 | 227 | 0 | 43 | 241 | 259 | 0 | 21 | 240 | 229 |
| 120 days | 0 | 30 | 221 | 302 | 0 | 24 | 224 | 335 | 0 | 12 | 232 | 316 |
| 150 days* | 9 | 33 | 254 | 291 | − | − | − | − | − | − | − | − |
| 180 days* | − | − | − | − | 10 | 42 | 241 | 515 | 0 | 16 | 257 | 338 |
| **PEG/SAXA = 1.2** | | | | | | | | | | | | |
| 30 days | 0 | 94 | 255 | 336 | 0 | 233 | 259 | 1273 | 0 | 27 | 240 | 232 |
| 60 days | 15 | 152 | 260 | 450 | 0 | 221 | 250 | 1548 | 0 | 32 | 244 | 308 |
| 90 days | 14 | 140 | 254 | 478 | 13 | 167 | 261 | 1764 | 0 | 24 | 246 | 318 |
| 120 days | 15 | 128 | 266 | 732 | 12 | 128 | 281 | 2163 | 0 | 10 | 241 | 410 |
| 150 days* | − | − | − | − | 11 | 123 | 272 | 1921 | 7 | 11 | 259 | 429 |
| 180 days* | 16 | 133 | 273 | 828 | − | − | − | − | − | − | − | − |
| **PEG/SAXA = 1.4** | | | | | | | | | | | | |
| 30 days | 13 | 228 | 284 | 804 | 0 | 267 | 262 | 1885 | 26 | 559 | 293 | 2674 |
| 60 days | 23 | 307 | 287 | 1251 | 17 | 230 | 267 | 2175 | 29 | 631 | 288 | 3725 |
| 90 days | 25 | 255 | 293 | 1424 | 16 | 177 | 270 | 2293 | 29 | 531 | 290 | 3947 |
| 120 days | 15 | 167 | 298 | 1839 | 14 | 136 | 314 | 2929 | 23 | 382 | 406 | 4964 |
| 150 days* | 20 | 204 | 289 | 1835 | − | − | − | − | − | − | − | − |
| 180 days* | − | − | − | − | 10 | 107 | 290 | 2999 | 17 | 339 | 362 | 4847 |

* some results for 150 and 180 day time point are not available due to experimental issues.

## 3.3. MODDE® Datasets and Statistics

**Table S9.** Worksheet for statistical evaluation.

| Exp No | Exp Name | Run Order | Incl/Excl | Time Point | Temperature | Relative humidity | PEG/SAXA ratio | ESCA | SFA | SCA | pH | aw | F | FA | Coloration |
|---|---|---|---|---|---|---|---|---|---|---|---|---|---|---|---|
| 1 | N1 | 1 | Incl | 7 | 60 | 10 | PEG/SAXA = 0.8 | 0.96 | 0.05 | 0.97 | 5.6 | 0.21 | 4.4 | 203.1 | 158 |
| 2 | N2 | 2 | Incl | 7 | 60 | 30 | PEG/SAXA = 0.8 | 1.80 | 0.07 | 0.55 | 5.53 | 0.31 | 1.5 | 232.7 | 129 |
| 3 | N3 | 3 | Incl | 7 | 60 | 50 | PEG/SAXA = 0.8 | 3.10 | 0.15 | 0.23 | 5.51 | 0.42 | 0.3 | 291.9 | 117 |
| 4 | N4 | 4 | Incl | 14 | 50 | 10 | PEG/SAXA = 0.8 | 0.50 | 0 | 0.87 | 5.59 | 0.22 | 7.6 | 202 | 197 |
| 5 | N5 | 5 | Incl | 14 | 50 | 30 | PEG/SAXA = 0.8 | 1.15 | 0.03 | 0.72 | 5.57 | 0.3 | 2.5 | 219.9 | 176 |
| 6 | N6 | 6 | Incl | 14 | 50 | 50 | PEG/SAXA = 0.8 | 2.66 | 0.12 | 0.3 | 5.54 | 0.43 | 0.8 | 252.6 | 132 |
| 7 | N7 | 7 | Incl | 14 | 60 | 10 | PEG/SAXA = 0.8 | 2.29 | 0.07 | 1.4 | 5.54 | 0.22 | 3.5 | 243.3 | 144 |
| 8 | N8 | 8 | Incl | 14 | 60 | 30 | PEG/SAXA = 0.8 | 3.84 | 0.12 | 0.69 | 5.5 | 0.3 | 1.6 | 289.3 | 133 |
| 9 | N9 | 9 | Incl | 14 | 60 | 50 | PEG/SAXA = 0.8 | 5.88 | 0.23 | 0.2 | 5.45 | 0.41 | 0 | 409.3 | 115 |
| 10 | N10 | 10 | Incl | 30 | 30 | 10 | PEG/SAXA = 0.8 | 0.04 | 0 | 0.12 | 5.74 | 0.2 | 27 | 211.3 | 212 |
| 11 | N11 | 11 | Incl | 30 | 30 | 30 | PEG/SAXA = 0.8 | 0.07 | 0 | 0.16 | 5.76 | 0.3 | 26.9 | 215.4 | 199 |
| 12 | N12 | 12 | Incl | 30 | 30 | 50 | PEG/SAXA = 0.8 | 0.31 | 0 | 0.22 | 5.73 | 0.43 | 24.6 | 211.7 | 191 |
| 13 | N13 | 13 | Incl | 30 | 40 | 10 | PEG/SAXA = 0.8 | 0.20 | 0 | 0.47 | 5.59 | 0.2 | 28.2 | 220.3 | 217 |
| 14 | N14 | 14 | Incl | 30 | 40 | 30 | PEG/SAXA = 0.8 | 0.49 | 0 | 0.53 | 5.67 | 0.29 | 26.1 | 224.8 | 195 |
| 15 | N15 | 15 | Incl | 30 | 40 | 50 | PEG/SAXA = 0.8 | 1.70 | 0.05 | 0.33 | 5.62 | 0.41 | 26.1 | 247 | 157 |
| 16 | N16 | 16 | Incl | 30 | 50 | 10 | PEG/SAXA = 0.8 | 1.33 | 0 | 1.35 | 5.67 | 0.21 | 28.1 | 248.6 | 199 |
| 17 | N17 | 17 | Incl | 30 | 50 | 30 | PEG/SAXA = 0.8 | 2.63 | 0.07 | 0.92 | 5.68 | 0.29 | 27.4 | 286.3 | 162 |
| 18 | N18 | 18 | Incl | 30 | 50 | 50 | PEG/SAXA = 0.8 | 4.21 | 0.16 | 0.4 | 5.66 | 0.37 | 34.5 | 364.4 | 125 |
| 19 | N19 | 19 | Incl | 45 | 50 | 10 | PEG/SAXA = 0.8 | 2.09 | 0.05 | 1.67 | 5.67 | 0.19 | 27.7 | 274.6 | 193 |
| 20 | N20 | 20 | Incl | 45 | 50 | 30 | PEG/SAXA = 0.8 | 3.97 | 0.1 | 1.02 | 5.66 | 0.27 | 30 | 328.2 | 155 |
| 21 | N21 | 21 | Incl | 45 | 50 | 50 | PEG/SAXA = 0.8 | 5.84 | 0.19 | 0.36 | 5.63 | 0.37 | 34.5 | 416.8 | 130 |
| 22 | N22 | 22 | Incl | 60 | 30 | 10 | PEG/SAXA = 0.8 | 0.11 | 0 | 0.26 | 5.6 | 0.22 | 33.7 | 205 | 203 |
| 23 | N23 | 23 | Incl | 60 | 30 | 30 | PEG/SAXA = 0.8 | 0.23 | 0 | 0.34 | 5.57 | 0.31 | 30.6 | 210 | 206 |
| 24 | N24 | 24 | Incl | 60 | 30 | 50 | PEG/SAXA = 0.8 | 0.93 | 0 | 0.38 | 5.56 | 0.42 | 25.6 | 202.3 | 175 |
| 25 | N25 | 25 | Incl | 60 | 40 | 10 | PEG/SAXA = 0.8 | 0.66 | 0 | 1.01 | 5.59 | 0.2 | 33.6 | 224 | 200 |
| 26 | N26 | 26 | Incl | 60 | 40 | 30 | PEG/SAXA = 0.8 | 1.51 | 0.05 | 0.99 | 5.58 | 0.28 | 30.3 | 226.2 | 178 |
| 27 | N27 | 27 | Incl | 60 | 40 | 50 | PEG/SAXA = 0.8 | 4.06 | 0.13 | 0.47 | 5.52 | 0.38 | 30.6 | 295.4 | 132 |
| 28 | N28 | 28 | Incl | 60 | 50 | 10 | PEG/SAXA = 0.8 | 3.73 | 0.08 | 2.16 | 5.6 | 0.2 | 45.5 | 2780 | 171 |
| 29 | N29 | 29 | Incl | 60 | 50 | 30 | PEG/SAXA = 0.8 | 6.35 | 0.14 | 1.36 | 5.55 | 0.27 | 39.8 | 479.9 | 147 |
| 30 | N30 | 30 | Incl | 60 | 50 | 50 | PEG/SAXA = 0.8 | 9.64 | 0.36 | 0.44 | 5.46 | 0.39 | 38.3 | 547.6 | 119 |
| 31 | N31 | 31 | Incl | 90 | 30 | 10 | PEG/SAXA = 0.8 | 0.17 | 0 | 0.44 | 5.71 | 0.19 | 29.2 | 211.5 | 212 |

**Table S9.** *Cont.*

| Exp No | Exp Name | Run Order | Incl/Excl | Time Point | Temperature | Relative humidity | PEG/SAXA ratio | ESCA | SFA | SCA | pH | aw | F | FA | Coloration |
|---|---|---|---|---|---|---|---|---|---|---|---|---|---|---|---|
| 32 | N32 | 32 | Incl | 90 | 30 | 30 | PEG/SAXA = 0.8 | 0.35 | 0 | 0.59 | 5.71 | 0.28 | 28.4 | 208.6 | 194 |
| 33 | N33 | 33 | Incl | 90 | 30 | 50 | PEG/SAXA = 0.8 | 1.36 | 0 | 0.34 | 5.62 | 0.38 | 25.2 | 215.5 | 167 |
| 34 | N34 | 34 | Incl | 90 | 40 | 10 | PEG/SAXA = 0.8 | 1.03 | 0 | 0.14 | 5.67 | 0.18 | 30.7 | 227.4 | 199 |
| 35 | N35 | 35 | Incl | 90 | 40 | 30 | PEG/SAXA = 0.8 | 2.16 | 0 | 0.53 | 5.63 | 0.26 | 24.3 | 265.4 | 171 |
| 36 | N36 | 36 | Incl | 90 | 40 | 50 | PEG/SAXA = 0.8 | 5.06 | 0.17 | 0.44 | 5.56 | 0.38 | 27.7 | 312.7 | 138 |
| 37 | N37 | 37 | Incl | 120 | 30 | 10 | PEG/SAXA = 0.8 | 0.21 | 0 | 0.18 | – | 0.17 | 15.4 | 255.4 | – |
| 38 | N38 | 38 | Incl | 120 | 30 | 30 | PEG/SAXA = 0.8 | 0.48 | 0 | 0.85 | – | 0.26 | 13 | 284.5 | – |
| 39 | N39 | 39 | Incl | 120 | 30 | 50 | PEG/SAXA = 0.8 | 1.57 | 0 | 0.42 | – | 0.37 | 9.9 | 300 | – |
| 40 | N40 | 40 | Incl | 120 | 40 | 10 | PEG/SAXA = 0.8 | 1.47 | 0 | 0.12 | – | 0.16 | 18.2 | 320.7 | – |
| 41 | N41 | 41 | Incl | 120 | 40 | 30 | PEG/SAXA = 0.8 | 3.02 | 0.06 | 0.07 | – | 0.24 | 14.1 | 345.9 | – |
| 42 | N42 | 42 | Incl | 120 | 40 | 50 | PEG/SAXA = 0.8 | 6.00 | 0.17 | 0.10 | – | 0.34 | 12 | 426.7 | – |
| 43 | N43 | 43 | Incl | 150 | 30 | 30 | PEG/SAXA = 0.8 | 0.68 | 0 | 0.13 | 5.75 | 0.28 | 14.6 | 231.1 | 201 |
| 44 | N44 | 44 | Incl | 150 | 30 | 50 | PEG/SAXA = 0.8 | 2.22 | 0.05 | 0.29 | 5.7 | 0.38 | 10.4 | 257.4 | 197 |
| 45 | N45 | 45 | Incl | 150 | 40 | 10 | PEG/SAXA = 0.8 | 1.90 | 0.05 | 0.32 | 5.68 | 0.18 | 18.5 | 300.7 | 197 |
| 46 | N46 | 46 | Incl | 150 | 40 | 30 | PEG/SAXA = 0.8 | 3.66 | 0.07 | 0.20 | 5.68 | 0.26 | 11.7 | 300.2 | 163 |
| 47 | N47 | 47 | Incl | 150 | 40 | 50 | PEG/SAXA = 0.8 | 5.16 | 0.11 | 0.82 | 5.62 | 0.32 | 7.6 | 476.1 | 129 |
| 48 | N48 | 48 | Incl | 180 | 30 | 10 | PEG/SAXA = 0.8 | 0.39 | 0 | 0.56 | 5.68 | 0.18 | 20.1 | 237.1 | 202 |
| 49 | N49 | 49 | Incl | 180 | 40 | 10 | PEG/SAXA = 0.8 | 2.41 | 0.07 | 0.24 | 5.63 | 0.18 | 27 | 311.7 | 189 |
| 50 | N50 | 50 | Incl | 180 | 40 | 30 | PEG/SAXA = 0.8 | 4.72 | 0.11 | 1.02 | 5.6 | 0.25 | 15.5 | 332.2 | 164 |
| 51 | N51 | 51 | Incl | 180 | 40 | 50 | PEG/SAXA = 0.8 | 7.63 | 0.2 | 0.62 | 5.57 | 0.33 | 15.4 | 441.3 | 124 |
| 52 | N52 | 52 | Incl | 7 | 60 | 10 | PEG/SAXA = 1.0 | 0.78 | 0.23 | 0.22 | 5.55 | 0.24 | 10.7 | 392.3 | 144 |
| 53 | N53 | 53 | Incl | 7 | 60 | 30 | PEG/SAXA = 1.0 | 1.87 | 0.27 | 0.16 | 5.5 | 0.31 | 1.7 | 456.8 | 136 |
| 54 | N54 | 54 | Incl | 7 | 60 | 50 | PEG/SAXA = 1.0 | 3.49 | 0.22 | 0.21 | 5.48 | 0.43 | 0 | 322.7 | 134 |
| 55 | N55 | 55 | Incl | 14 | 50 | 10 | PEG/SAXA = 1.0 | 0.38 | 0.11 | 0.23 | 5.56 | 0.21 | 26.7 | 303.9 | 184 |
| 56 | N56 | 56 | Incl | 14 | 50 | 30 | PEG/SAXA = 1.0 | 1.20 | 0.15 | 0.62 | 5.54 | 0.3 | 5.4 | 372.6 | 145 |
| 57 | N57 | 57 | Incl | 14 | 50 | 50 | PEG/SAXA = 1.0 | 2.91 | 0.18 | 0.60 | 5.51 | 0.42 | 1.7 | 269.4 | 137 |
| 58 | N58 | 58 | Incl | 14 | 60 | 10 | PEG/SAXA = 1.0 | 2.00 | 0.24 | 0.29 | 5.54 | 0.21 | 4.9 | 482.3 | 150 |
| 59 | N59 | 59 | Incl | 14 | 60 | 30 | PEG/SAXA = 1.0 | 3.92 | 0.22 | 1.32 | 5.5 | 0.31 | 2.5 | 502.2 | 134 |
| 60 | N60 | 60 | Incl | 14 | 60 | 50 | PEG/SAXA = 1.0 | 6.11 | 0.3 | 0.83 | 5.45 | 0.41 | 0.3 | 439.8 | 124 |
| 61 | N61 | 61 | Incl | 30 | 30 | 10 | PEG/SAXA = 1.0 | 0.04 | 0 | 0.27 | 5.72 | 0.19 | 35.5 | 215.6 | 222 |
| 62 | N62 | 62 | Incl | 30 | 30 | 30 | PEG/SAXA = 1.0 | 0.07 | 0.05 | 0.27 | 5.71 | 0.29 | 56.8 | 293.6 | 208 |
| 63 | N63 | 63 | Incl | 30 | 30 | 50 | PEG/SAXA = 1.0 | 0.48 | 0 | 0.33 | 5.69 | 0.41 | 22.1 | 209.5 | 194 |
| 64 | N64 | 64 | Incl | 30 | 40 | 10 | PEG/SAXA = 1.0 | 0.16 | 0.06 | 0.30 | 5.66 | 0.2 | 42.5 | 273.6 | 219 |

**Table S9.** *Cont.*

| Exp No | Exp Name | Run Order | Incl/Excl | Time Point | Temperature | Relative humidity | PEG/SAXA ratio | ESCA | SFA | SCA | pH | aw | F | FA | Coloration |
|---|---|---|---|---|---|---|---|---|---|---|---|---|---|---|---|
| 65 | N65 | 65 | Incl | 30 | 40 | 30 | PEG/SAXA = 1.0 | 0.47 | 0.09 | 0.91 | 5.65 | 0.29 | 37.7 | 385.9 | 199 |
| 66 | N66 | 66 | Incl | 30 | 40 | 50 | PEG/SAXA = 1.0 | 1.87 | 0.08 | 0.81 | 5.64 | 0.41 | 24.1 | 259.5 | 140 |
| 67 | N67 | 67 | Incl | 30 | 50 | 10 | PEG/SAXA = 1.0 | 1.08 | 0.17 | 0.31 | 5.69 | 0.21 | 36.3 | 408.9 | 186 |
| 68 | N68 | 68 | Incl | 30 | 50 | 30 | PEG/SAXA = 1.0 | 2.75 | 0.22 | 0.34 | 5.67 | 0.29 | 28.9 | 487.8 | 156 |
| 69 | N69 | 69 | Incl | 30 | 50 | 50 | PEG/SAXA = 1.0 | 4.86 | 0.28 | 0.38 | 5.64 | 0.39 | 37.7 | 408.1 | 141 |
| 70 | N70 | 70 | Incl | 45 | 50 | 10 | PEG/SAXA = 1.0 | 1.74 | 0.21 | 0.35 | 5.67 | 0.19 | 33.8 | 457 | 170 |
| 71 | N71 | 71 | Incl | 45 | 50 | 30 | PEG/SAXA = 1.0 | 3.86 | 0.28 | 1.10 | 5.66 | 0.27 | 27.1 | 511.5 | 139 |
| 72 | N72 | 72 | Incl | 45 | 50 | 50 | PEG/SAXA = 1.0 | 5.59 | 0.27 | 0.93 | 5.61 | 0.38 | 33.8 | 445 | 120 |
| 73 | N73 | 73 | Incl | 60 | 30 | 10 | PEG/SAXA = 1.0 | 0.10 | 0 | 0.34 | 5.57 | 0.21 | 46.9 | 229.4 | 207 |
| 74 | N74 | 74 | Incl | 60 | 30 | 30 | PEG/SAXA = 1.0 | 0.23 | 0 | 0.52 | 5.57 | 0.3 | 42.1 | 245.1 | 194 |
| 75 | N75 | 75 | Incl | 60 | 30 | 50 | PEG/SAXA = 1.0 | 1.02 | 0.05 | 0.41 | 5.56 | 0.42 | 23.3 | 228.3 | 168 |
| 76 | N76 | 76 | Incl | 60 | 40 | 10 | PEG/SAXA = 1.0 | 0.56 | 0.08 | 1.30 | 5.56 | 0.2 | 46.4 | 281.8 | 192 |
| 77 | N77 | 77 | Incl | 60 | 40 | 30 | PEG/SAXA = 1.0 | 1.29 | 0.24 | 1.05 | 5.53 | 0.29 | 43.6 | 563.5 | 172 |
| 78 | N78 | 78 | Incl | 60 | 40 | 50 | PEG/SAXA = 1.0 | 4.34 | 0.18 | 0.72 | 5.49 | 0.4 | 26.7 | 327.6 | 135 |
| 79 | N79 | 79 | Incl | 60 | 50 | 10 | PEG/SAXA = 1.0 | 3.28 | 0.28 | 0.54 | 5.54 | 0.2 | 81.7 | 4162.7 | 165 |
| 80 | N80 | 80 | Incl | 60 | 50 | 30 | PEG/SAXA = 1.0 | 6.59 | 0.4 | 1.43 | 5.49 | 0.31 | 30.2 | 433.2 | 117 |
| 81 | N81 | 81 | Incl | 60 | 50 | 50 | PEG/SAXA = 1.0 | 9.87 | 0.47 | 1.21 | 5.45 | 0.38 | 25.9 | 549.7 | 124 |
| 82 | N82 | 82 | Incl | 90 | 30 | 10 | PEG/SAXA = 1.0 | 0.15 | 0 | 0.49 | 5.63 | 0.19 | 41.5 | 226.6 | 190 |
| 83 | N83 | 83 | Incl | 90 | 30 | 30 | PEG/SAXA = 1.0 | 0.35 | 0 | 0.41 | 5.6 | 0.27 | 42.8 | 258.7 | 200 |
| 84 | N84 | 84 | Incl | 90 | 30 | 50 | PEG/SAXA = 1.0 | 1.68 | 0.05 | 0.24 | 5.58 | 0.39 | 21.4 | 229.5 | 169 |
| 85 | N85 | 85 | Incl | 90 | 40 | 10 | PEG/SAXA = 1.0 | 0.84 | 0.07 | 0.10 | 5.63 | 0.18 | 42.9 | 309.2 | 183 |
| 86 | N86 | 86 | Incl | 90 | 40 | 30 | PEG/SAXA = 1.0 | 2.24 | 0.11 | 0.37 | 5.6 | 0.26 | 28.8 | 452.8 | 155 |
| 87 | N87 | 87 | Incl | 90 | 40 | 50 | PEG/SAXA = 1.0 | 4.87 | 0.21 | 0.32 | 5.56 | 0.36 | 25.2 | 355.7 | 133 |
| 88 | N88 | 88 | Incl | 120 | 30 | 10 | PEG/SAXA = 1.0 | 0.20 | 0 | 0.16 | – | 0.18 | 29.8 | 302.3 | – |
| 89 | N89 | 89 | Incl | 120 | 30 | 30 | PEG/SAXA = 1.0 | 0.40 | 0.08 | 0.50 | – | 0.27 | 24.1 | 334.9 | – |
| 90 | N90 | 90 | Incl | 120 | 30 | 50 | PEG/SAXA = 1.0 | 1.89 | 0.06 | 0.24 | – | 0.37 | 12.2 | 316.1 | – |
| 91 | N91 | 91 | Incl | 120 | 40 | 10 | PEG/SAXA = 1.0 | 1.18 | 0.08 | 0.09 | – | 0.16 | 31.5 | 506.2 | – |
| 92 | N92 | 92 | Incl | 120 | 40 | 30 | PEG/SAXA = 1.0 | 2.65 | 0.23 | 0.06 | – | 0.24 | 15.4 | 623.6 | – |
| 93 | N93 | 93 | Incl | 120 | 40 | 50 | PEG/SAXA = 1.0 | 5.64 | 0.21 | 0.08 | – | 0.33 | 11.9 | 606.2 | – |
| 94 | N94 | 94 | Incl | 150 | 30 | 10 | PEG/SAXA = 1.0 | 0.27 | 0.04 | 0.13 | 5.72 | 0.18 | 33.2 | 291.1 | 210 |
| 95 | N95 | 95 | Incl | 150 | 40 | 10 | PEG/SAXA = 1.0 | 1.62 | 0.11 | 0.21 | 5.66 | 0.18 | 14.8 | 715.5 | 182 |

**Table S9.** *Cont.*

| Exp No | Exp Name | Run Order | Incl/Excl | Time Point | Temperature | Relative humidity | PEG/SAXA ratio | ESCA | SFA | SCA | pH | aw | F | FA | Coloration |
|---|---|---|---|---|---|---|---|---|---|---|---|---|---|---|---|
| 96 | N96 | 96 | Incl | 150 | 40 | 30 | PEG/SAXA = 1.0 | 3.68 | 0.13 | 0.26 | 5.61 | 0.26 | 31.1 | 363.3 | 160 |
| 97 | N97 | 97 | Incl | 150 | 40 | 50 | PEG/SAXA = 1.0 | 7.53 | 0.26 | 0.16 | 5.56 | 0.36 | 10.2 | 341.6 | 122 |
| 98 | N98 | 98 | Incl | 180 | 30 | 30 | PEG/SAXA = 1.0 | 0.61 | 0.17 | 0.60 | 5.63 | 0.27 | 41.7 | 515.4 | 190 |
| 99 | N99 | 99 | Incl | 180 | 30 | 50 | PEG/SAXA = 1.0 | 2.80 | 0.11 | 0.38 | 5.62 | 0.38 | 15.6 | 338.2 | 147 |
| 100 | N100 | 100 | Incl | 180 | 40 | 10 | PEG/SAXA = 1.0 | 2.08 | 0.15 | 0.15 | 5.63 | 0.18 | 31.8 | 430.6 | 179 |
| 101 | N101 | 101 | Incl | 180 | 40 | 30 | PEG/SAXA = 1.0 | 3.88 | 0.35 | 0.73 | 5.59 | 0.26 | 19.7 | 928.4 | 148 |
| 102 | N102 | 102 | Incl | 180 | 40 | 50 | PEG/SAXA = 1.0 | 9.18 | 0.35 | 0.43 | 5.54 | 0.36 | 17.8 | 571.2 | 122 |
| 103 | N103 | 103 | Incl | 7 | 60 | 10 | PEG/SAXA = 1.2 | 0.63 | 0.65 | 0.20 | 5.44 | 0.21 | 19 | 755.6 | 149 |
| 104 | N104 | 104 | Incl | 7 | 60 | 30 | PEG/SAXA = 1.2 | 1.74 | 0.67 | 0.13 | 5.43 | 0.33 | 2.6 | 923.6 | 127 |
| 105 | N105 | 105 | Incl | 7 | 60 | 50 | PEG/SAXA = 1.2 | 2.38 | 0.32 | 0.18 | 5.47 | 0.44 | 1.4 | 391.2 | 132 |
| 106 | N106 | 106 | Incl | 14 | 50 | 10 | PEG/SAXA = 1.2 | 0.27 | 0.6 | 0.21 | 5.48 | 0.22 | 50.4 | 762.9 | 174 |
| 107 | N107 | 107 | Incl | 14 | 50 | 30 | PEG/SAXA = 1.2 | 1.11 | 0.58 | 0.45 | 5.46 | 0.32 | 9.9 | 891.3 | 150 |
| 108 | N108 | 108 | Incl | 14 | 50 | 50 | PEG/SAXA = 1.2 | 2.24 | 0.25 | 0.42 | 5.53 | 0.45 | 2 | 342 | 152 |
| 109 | N109 | 109 | Incl | 14 | 60 | 10 | PEG/SAXA = 1.2 | 1.73 | 0.72 | 0.21 | 5.46 | 0.21 | 6.2 | 977.4 | 121 |
| 110 | N110 | 110 | Incl | 14 | 60 | 30 | PEG/SAXA = 1.2 | 3.46 | 0.71 | 0.96 | 5.44 | 0.32 | 1.1 | 1070.1 | 124 |
| 111 | N111 | 111 | Incl | 14 | 60 | 50 | PEG/SAXA = 1.2 | 3.33 | 0.68 | 0.47 | 5.46 | 0.44 | 1.9 | 803 | 138 |
| 112 | N112 | 112 | Incl | 30 | 30 | 10 | PEG/SAXA = 1.2 | 0.03 | 0.12 | 0.20 | 5.7 | 0.22 | 93.5 | 335.7 | 211 |
| 113 | N113 | 113 | Incl | 30 | 30 | 30 | PEG/SAXA = 1.2 | 0.04 | 0.49 | 0.23 | 5.59 | 0.31 | 232.7 | 1272.8 | 203 |
| 114 | N114 | 114 | Incl | 30 | 30 | 50 | PEG/SAXA = 1.2 | 0.51 | 0.06 | 0.29 | 5.7 | 0.42 | 27 | 231.9 | 192 |
| 115 | N115 | 115 | Incl | 30 | 40 | 10 | PEG/SAXA = 1.2 | 0.07 | 0.48 | 0.24 | 5.61 | 0.2 | 122.9 | 868.5 | 188 |
| 116 | N116 | 116 | Incl | 30 | 40 | 30 | PEG/SAXA = 1.2 | 0.36 | 0.48 | 0.69 | 5.59 | 0.3 | 63.4 | 1142.7 | 192 |
| 117 | N117 | 117 | Incl | 30 | 40 | 50 | PEG/SAXA = 1.2 | 1.80 | 0.12 | 0.62 | 5.65 | 0.4 | 22.5 | 301.5 | 164 |
| 118 | N118 | 118 | Incl | 30 | 50 | 10 | PEG/SAXA = 1.2 | 0.87 | 0.71 | 0.27 | 5.59 | 0.2 | 48.9 | 1141.3 | 160 |
| 119 | N119 | 119 | Incl | 30 | 50 | 30 | PEG/SAXA = 1.2 | 2.42 | 0.69 | 0.28 | 5.58 | 0.3 | 28.6 | 1314.2 | 146 |
| 120 | N120 | 120 | Incl | 30 | 50 | 50 | PEG/SAXA = 1.2 | 3.46 | 0.38 | 0.26 | 5.61 | 0.42 | 40.4 | 504.1 | 145 |
| 121 | N121 | 121 | Incl | 45 | 50 | 10 | PEG/SAXA = 1.2 | 1.60 | 0.77 | 0.27 | 5.58 | 0.2 | 36.1 | 1192.2 | 155 |
| 122 | N122 | 122 | Incl | 45 | 50 | 30 | PEG/SAXA = 1.2 | 3.74 | 0.82 | 0.81 | 5.58 | 0.29 | 29.7 | 1274.1 | 142 |
| 123 | N123 | 123 | Incl | 45 | 50 | 50 | PEG/SAXA = 1.2 | 5.36 | 0.41 | 0.56 | 5.59 | 0.39 | 46 | 526.1 | 130 |
| 124 | N124 | 124 | Incl | 60 | 30 | 10 | PEG/SAXA = 1.2 | 0.05 | 0.26 | 0.31 | 5.52 | 0.21 | 151.6 | 449.8 | 202 |
| 125 | N125 | 125 | Incl | 60 | 30 | 30 | PEG/SAXA = 1.2 | 0.09 | 0.76 | 0.35 | 5.37 | 0.32 | 220.6 | 1547.9 | 192 |
| 126 | N126 | 126 | Incl | 60 | 30 | 50 | PEG/SAXA = 1.2 | 0.99 | 0.09 | 1.00 | 5.54 | 0.43 | 31.7 | 308.5 | 169 |
| 127 | N127 | 127 | Incl | 60 | 40 | 10 | PEG/SAXA = 1.2 | 0.25 | 0.68 | 0.74 | 5.45 | 0.23 | 110.5 | 1015.1 | 187 |
| 128 | N128 | 128 | Incl | 60 | 40 | 30 | PEG/SAXA = 1.2 | 1.26 | 0.66 | 0.30 | 5.43 | 0.31 | 43.6 | 1153.2 | 160 |

**Table S9.** *Cont.*

| Exp No | Exp Name | Run Order | Incl/Excl | Time Point | Temperature | Relative humidity | PEG/SAXA ratio | ESCA | SFA | SCA | pH | aw | F | FA | Coloration |
|---|---|---|---|---|---|---|---|---|---|---|---|---|---|---|---|
| 129 | N129 | 129 | Incl | 60 | 40 | 50 | PEG/SAXA = 1.2 | 3.67 | 0.25 | 0.37 | 5.48 | 0.41 | 28.3 | 409.9 | 150 |
| 130 | N130 | 130 | Incl | 60 | 50 | 10 | PEG/SAXA = 1.2 | 2.76 | 1.05 | 0.32 | 5.45 | 0.22 | 29.5 | 342 | 165 |
| 131 | N131 | 131 | Incl | 60 | 50 | 30 | PEG/SAXA = 1.2 | 6.21 | 1.09 | 1.14 | 5.41 | 0.3 | 28.2 | 1398.9 | 129 |
| 132 | N132 | 132 | Incl | 60 | 50 | 50 | PEG/SAXA = 1.2 | 8.30 | 0.57 | 0.74 | 5.46 | 0.41 | 38.8 | 625.1 | 139 |
| 133 | N133 | 133 | Incl | 90 | 30 | 10 | PEG/SAXA = 1.2 | 0.06 | 0.24 | 0.28 | 5.56 | 0.19 | 140.2 | 478.3 | 207 |
| 134 | N134 | 134 | Incl | 90 | 30 | 30 | PEG/SAXA = 1.2 | 0.16 | 0.52 | 0.19 | 5.46 | 0.3 | 166.6 | 1764.3 | 190 |
| 135 | N135 | 135 | Incl | 90 | 30 | 50 | PEG/SAXA = 1.2 | 1.60 | 0.12 | 0.11 | 5.57 | 0.39 | 24.1 | 318.4 | 166 |
| 136 | N136 | 136 | Incl | 90 | 40 | 10 | PEG/SAXA = 1.2 | 0.45 | 0.58 | 0.00 | 5.53 | 0.19 | 84.6 | 1078.5 | 168 |
| 137 | N137 | 137 | Incl | 90 | 40 | 30 | PEG/SAXA = 1.2 | 1.78 | 0.55 | 0.20 | 5.5 | 0.27 | 40 | 1455.7 | 150 |
| 138 | N138 | 138 | Incl | 90 | 40 | 50 | PEG/SAXA = 1.2 | 4.90 | 0.25 | 0.15 | 5.56 | 0.37 | 28.6 | 459.8 | 134 |
| 139 | N139 | 139 | Incl | 120 | 30 | 10 | PEG/SAXA = 1.2 | 0.08 | 0.27 | 0.04 | – | 0.18 | 128.3 | 732 | – |
| 140 | N140 | 140 | Incl | 120 | 30 | 30 | PEG/SAXA = 1.2 | 0.20 | 0.55 | 0.18 | – | 0.3 | 127.8 | 2163.5 | – |
| 141 | N141 | 141 | Incl | 120 | 30 | 50 | PEG/SAXA = 1.2 | 1.49 | 0.11 | 0.11 | – | 0.39 | 10 | 409.7 | – |
| 142 | N142 | 142 | Incl | 120 | 40 | 10 | PEG/SAXA = 1.2 | 0.77 | 0.55 | 0.00 | – | 0.16 | 69.6 | 1799.6 | – |
| 143 | N143 | 143 | Incl | 120 | 40 | 30 | PEG/SAXA = 1.2 | 2.56 | 0.74 | 0.04 | – | 0.26 | 18.5 | 1924.4 | – |
| 144 | N144 | 144 | Incl | 120 | 40 | 50 | PEG/SAXA = 1.2 | 5.57 | 0.26 | 0.03 | – | 0.36 | 12.8 | 715.3 | – |
| 145 | N145 | 145 | Incl | 150 | 30 | 30 | PEG/SAXA = 1.2 | 0.31 | 0.57 | 0.09 | 5.55 | 0.29 | 123.5 | 1920.6 | 175 |
| 146 | N146 | 146 | Incl | 150 | 30 | 50 | PEG/SAXA = 1.2 | 2.28 | 0.12 | 0.09 | 5.62 | 0.39 | 10.6 | 428.5 | 147 |
| 147 | N147 | 147 | Incl | 150 | 40 | 10 | PEG/SAXA = 1.2 | 1.12 | 0.62 | 0.15 | 5.56 | 0.19 | 52.1 | 1247.6 | 173 |
| 148 | N148 | 148 | Incl | 150 | 40 | 30 | PEG/SAXA = 1.2 | 3.25 | 0.74 | 0.10 | 5.51 | 0.28 | 17.4 | 1528.6 | 147 |
| 149 | N149 | 149 | Incl | 150 | 40 | 50 | PEG/SAXA = 1.2 | 6.27 | 0.41 | 0.27 | 5.56 | 0.38 | 10.3 | 561.1 | 132 |
| 150 | N150 | 150 | Incl | 180 | 30 | 10 | PEG/SAXA = 1.2 | 0.13 | 0.34 | 0.16 | 5.63 | 0.19 | 133.5 | 828.4 | 183 |
| 151 | N151 | 151 | Incl | 180 | 40 | 10 | PEG/SAXA = 1.2 | 1.41 | 0.74 | 0.00 | 5.52 | 0.19 | 54.4 | 1472.6 | 155 |
| 152 | N152 | 152 | Incl | 180 | 40 | 30 | PEG/SAXA = 1.2 | 4.38 | 0.82 | 0.29 | 5.51 | 0.27 | 23.7 | 1576.9 | 122 |
| 153 | N153 | 153 | Incl | 180 | 40 | 50 | PEG/SAXA = 1.2 | 7.84 | 0.44 | 0.16 | 5.53 | 0.37 | 16.6 | 618.3 | 130 |
| 154 | N154 | 154 | Incl | 7 | 60 | 10 | PEG/SAXA = 1.4 | 0.46 | 1.36 | 0.07 | 5.37 | 0.21 | 69.3 | 1250.6 | 179 |
| 155 | N155 | 155 | Incl | 7 | 60 | 30 | PEG/SAXA = 1.4 | 0.95 | 1.87 | 0.05 | 5.3 | 0.33 | 18.6 | 2038.1 | 153 |
| 156 | N156 | 156 | Incl | 7 | 60 | 50 | PEG/SAXA = 1.4 | 0.80 | 2.82 | 0.05 | 5.16 | 0.45 | 230.2 | 3317.6 | 185 |
| 157 | N157 | 157 | Incl | 14 | 50 | 10 | PEG/SAXA = 1.4 | 0.24 | 1.29 | 0.15 | 5.42 | 0.21 | 97 | 1154.1 | 199 |
| 158 | N158 | 158 | Incl | 14 | 50 | 30 | PEG/SAXA = 1.4 | 0.72 | 1.64 | 0.23 | 5.34 | 0.33 | 52.5 | 1863.2 | 182 |
| 159 | N159 | 159 | Incl | 14 | 50 | 50 | PEG/SAXA = 1.4 | 0.49 | 2.42 | 0.23 | 5.19 | 0.45 | 466 | 3197.2 | 190 |
| 160 | N160 | 160 | Incl | 14 | 60 | 10 | PEG/SAXA = 1.4 | 1.35 | 1.69 | 0.10 | 5.36 | 0.22 | 16.4 | 1464.2 | 156 |
| 161 | N161 | 161 | Incl | 14 | 60 | 30 | PEG/SAXA = 1.4 | 1.85 | 2.23 | 0.30 | 5.31 | 0.33 | 6.1 | 2202.1 | 143 |

**Table S9.** *Cont.*

| Exp No | Exp Name | Run Order | Incl/Excl | Time Point | Temperature | Relative humidity | PEG/SAXA ratio | ESCA | SFA | SCA | pH | aw | F | FA | Coloration |
|---|---|---|---|---|---|---|---|---|---|---|---|---|---|---|---|
| 162 | N162 | 162 | Incl | 14 | 60 | 50 | PEG/SAXA = 1.4 | 2.07 | 3.28 | 0.20 | 5.15 | 0.43 | 30.8 | 3267.9 | 164 |
| 163 | N163 | 163 | Incl | 30 | 30 | 10 | PEG/SAXA = 1.4 | 0.00 | 0.56 | 0.06 | 5.57 | 0.21 | 228.5 | 804.2 | 204 |
| 164 | N164 | 164 | Incl | 30 | 30 | 30 | PEG/SAXA = 1.4 | 0.04 | 0.8 | 0.09 | 5.48 | 0.32 | 267.4 | 1884.6 | 198 |
| 165 | N165 | 165 | Incl | 30 | 30 | 50 | PEG/SAXA = 1.4 | 0.13 | 1.15 | 0.10 | 5.4 | 0.47 | 558.5 | 2674.5 | 211 |
| 166 | N166 | 166 | Incl | 30 | 40 | 10 | PEG/SAXA = 1.4 | 0.06 | 0.96 | 0.13 | 5.51 | 0.2 | 148.7 | 1436.7 | 210 |
| 167 | N167 | 167 | Incl | 30 | 40 | 30 | PEG/SAXA = 1.4 | 0.34 | 1.1 | 0.33 | 5.47 | 0.32 | 106.9 | 2216.3 | 201 |
| 168 | N168 | 168 | Incl | 30 | 40 | 50 | PEG/SAXA = 1.4 | 0.30 | 1.95 | 0.25 | 5.29 | 0.46 | 518.3 | 4217.5 | 209 |
| 169 | N169 | 169 | Incl | 30 | 50 | 10 | PEG/SAXA = 1.4 | 0.85 | 1.38 | 0.12 | 5.53 | 0.2 | 55.1 | 1773.4 | 187 |
| 170 | N170 | 170 | Incl | 30 | 50 | 30 | PEG/SAXA = 1.4 | 1.74 | 1.84 | 0.10 | 5.43 | 0.32 | 39.6 | 2628.4 | 174 |
| 171 | N171 | 171 | Incl | 30 | 50 | 50 | PEG/SAXA = 1.4 | 1.09 | 2.76 | 0.11 | 5.28 | 0.43 | 209 | 4284.7 | 187 |
| 172 | N172 | 172 | Incl | 45 | 50 | 10 | PEG/SAXA = 1.4 | 1.38 | 1.43 | 0.18 | 5.49 | 0.21 | 47.6 | 1851.1 | 172 |
| 173 | N173 | 173 | Incl | 45 | 50 | 30 | PEG/SAXA = 1.4 | 2.33 | 2.14 | 0.39 | 5.43 | 0.31 | 40.1 | 2678.2 | 155 |
| 174 | N174 | 174 | Incl | 45 | 50 | 50 | PEG/SAXA = 1.4 | 1.51 | 2.99 | 0.27 | 5.27 | 0.41 | 135.9 | 4097.2 | 173 |
| 175 | N175 | 175 | Incl | 60 | 30 | 10 | PEG/SAXA = 1.4 | 0.03 | 0.93 | 0.09 | 5.38 | 0.21 | 306.8 | 1250.6 | 202 |
| 176 | N176 | 176 | Incl | 60 | 30 | 30 | PEG/SAXA = 1.4 | 0.10 | 1.1 | 0.16 | 5.31 | 0.32 | 230.3 | 2175.3 | 202 |
| 177 | N177 | 177 | Incl | 60 | 30 | 50 | PEG/SAXA = 1.4 | 0.24 | 1.86 | 0.18 | 5.17 | 0.44 | 631.3 | 3724.6 | 201 |
| 178 | N178 | 178 | Incl | 60 | 40 | 10 | PEG/SAXA = 1.4 | 0.33 | 1.41 | 0.49 | 5.36 | 0.22 | 110.6 | 1737.8 | 194 |
| 179 | N179 | 179 | Incl | 60 | 40 | 30 | PEG/SAXA = 1.4 | 1.09 | 1.56 | 0.29 | 5.29 | 0.32 | 58.9 | 2371.9 | 182 |
| 180 | N180 | 180 | Incl | 60 | 40 | 50 | PEG/SAXA = 1.4 | 0.72 | 2.72 | 0.08 | 5.14 | 0.44 | 309 | 4132 | 188 |
| 181 | N181 | 181 | Incl | 60 | 50 | 10 | PEG/SAXA = 1.4 | 2.57 | 1.99 | 0.16 | 5.34 | 0.2 | 37.8 | 1739.1 | 169 |
| 182 | N182 | 182 | Incl | 60 | 50 | 30 | PEG/SAXA = 1.4 | 3.82 | 2.79 | 0.52 | 5.29 | 0.3 | 35 | 1258.4 | 157 |
| 183 | N183 | 183 | Incl | 60 | 50 | 50 | PEG/SAXA = 1.4 | 2.72 | 3.94 | 0.31 | 5.16 | 0.41 | 29.6 | 295.1 | 182 |
| 184 | N184 | 184 | Incl | 90 | 30 | 10 | PEG/SAXA = 1.4 | 0.04 | 0.87 | 0.11 | 5.49 | 0.19 | 254.9 | 1424.4 | 199 |
| 185 | N185 | 185 | Incl | 90 | 30 | 30 | PEG/SAXA = 1.4 | 0.19 | 0.89 | 0.07 | 5.37 | 0.3 | 177.3 | 2292.5 | 186 |
| 186 | N186 | 186 | Incl | 90 | 30 | 50 | PEG/SAXA = 1.4 | 0.33 | 1.79 | 0.00 | 5.23 | 0.43 | 531.2 | 3946.7 | 208 |
| 187 | N187 | 187 | Incl | 90 | 40 | 10 | PEG/SAXA = 1.4 | 0.58 | 1.24 | 0.00 | 5.42 | 0.18 | 86.8 | 1847.6 | 168 |
| 188 | N188 | 188 | Incl | 90 | 40 | 30 | PEG/SAXA = 1.4 | 1.73 | 1.34 | 0.10 | 5.42 | 0.3 | 40.3 | 2303.5 | 160 |
| 189 | N189 | 189 | Incl | 90 | 40 | 50 | PEG/SAXA = 1.4 | 1.26 | 2.37 | 0.04 | 5.26 | 0.41 | 276.2 | 4006.5 | 173 |
| 190 | N190 | 190 | Incl | 120 | 30 | 10 | PEG/SAXA = 1.4 | 0.05 | 0.75 | 0.00 | − | 0.17 | 166.8 | 1838.7 | − |
| 191 | N191 | 191 | Incl | 120 | 30 | 30 | PEG/SAXA = 1.4 | 0.27 | 0.97 | 0.05 | − | 0.28 | 136.2 | 2928.7 | − |
| 192 | N192 | 192 | Incl | 120 | 30 | 50 | PEG/SAXA = 1.4 | 0.46 | 1.7 | 0.00 | − | 0.38 | 381.9 | 4964.5 | − |
| 193 | N193 | 193 | Incl | 120 | 40 | 10 | PEG/SAXA = 1.4 | 0.94 | 1.24 | 0.00 | − | 0.17 | 70.6 | 2899.6 | − |
| 194 | N194 | 194 | Incl | 120 | 40 | 30 | PEG/SAXA = 1.4 | 2.30 | 1.5 | 0.00 | − | 0.27 | 25.4 | 3796.2 | − |

**Table S9.** *Cont.*

| Exp No | Exp Name | Run Order | Incl/Excl | Time Point | Temperature | Relative humidity | PEG/SAXA ratio | ESCA | SFA | SCA | pH | aw | F | FA | Coloration |
|---|---|---|---|---|---|---|---|---|---|---|---|---|---|---|---|
| 195 | N195 | 195 | Incl | 120 | 40 | 50 | PEG/SAXA = 1.4 | 1.43 | 2.58 | 0.00 | – | 0.34 | 148.6 | 6104.6 | – |
| 196 | N196 | 196 | Incl | 150 | 30 | 10 | PEG/SAXA = 1.4 | 0.07 | 0.89 | 0.00 | 5.49 | 0.2 | 203.7 | 1835.3 | 180 |
| 197 | N197 | 197 | Incl | 150 | 40 | 10 | PEG/SAXA = 1.4 | 1.32 | 1.33 | 0.07 | 5.4 | 0.19 | 44.9 | 2114.6 | 165 |
| 198 | N198 | 198 | Incl | 150 | 40 | 30 | PEG/SAXA = 1.4 | 2.88 | 1.59 | 0.06 | 5.4 | 0.28 | 27.6 | 2560.1 | 159 |
| 199 | N199 | 199 | Incl | 150 | 40 | 50 | PEG/SAXA = 1.4 | 1.92 | 2.87 | 0.00 | 5.26 | 0.39 | 129 | 4203.5 | 184 |
| 200 | N200 | 200 | Incl | 180 | 30 | 30 | PEG/SAXA = 1.4 | 0.56 | 1.08 | 0.10 | 5.42 | 0.29 | 106.5 | 2999 | 166 |
| 201 | N201 | 201 | Incl | 180 | 30 | 50 | PEG/SAXA = 1.4 | 0.72 | 1.99 | 0.00 | 5.27 | 0.43 | 339.1 | 4847 | 184 |
| 202 | N202 | 202 | Incl | 180 | 40 | 10 | PEG/SAXA = 1.4 | 1.68 | 1.39 | 0.00 | 5.45 | 0.19 | 43.9 | 2522.7 | 163 |
| 203 | N203 | 203 | Incl | 180 | 40 | 30 | PEG/SAXA = 1.4 | 3.73 | 1.66 | 0.09 | 5.42 | 0.28 | 24.2 | 2770.7 | 136 |
| 204 | N204 | 204 | Incl | 180 | 40 | 50 | PEG/SAXA = 1.4 | 2.25 | 2.77 | 0.00 | 5.28 | 0.38 | 94.3 | 4497.6 | 164 |

**Table S10.** Factors.

| Name | Abbr. | Units | Type | Settings | Transform | Precision |
|---|---|---|---|---|---|---|
| **Time Point** | TP | d | Quantitative | 7 to 180 | None | 4.32 |
| **Temperature** | T | °C | Quantitative | 30 to 60 | None | 0.75 |
| **Relative humidity** | RH | RH | Quantitative | 10 to 50 | None | 1 |
| **PEG/SAXA ratio** | ratio | | Qualitative | PEG/SAXA = 0.8, PEG/SAXA = 1.0, PEG/SAXA = 1.2, PEG/SAXA = 1.4 | – | – |

**Table S11.** Responses.

| Name | Abbr. | Transform | Type |
|---|---|---|---|
| **ESCA** | ESCA | Log (10Log(Y)) | Regular |
| **SFA** | SFA | Log (10Log(Y)) | Regular |
| **SCA** | CA | Log (10Log(Y)) | Regular |
| **pH** | pH | None | Regular |
| **aw** | aw | None | Regular |
| **F** | F | Log (10Log(Y)) | Regular |
| **FA** | FA | Log (10Log(Y)) | Regular |
| **Coloration** | Color | None | Regular |

**Table S12.** Coefficient list.

| SCA~ | Coeff. SC | Std. Err. | P | Conf. int(±) |
|---|---|---|---|---|
| **Constant** | −0.40 | 0.02 | 0.00 | 0.04 |
| **Time Point** | 0.33 | 0.03 | 0.00 | 0.05 |
| **Temperature** | 0.23 | 0.03 | 0.00 | 0.05 |
| **Relative humidity** | −0.15 | 0.02 | 0.00 | 0.04 |
| | | | | |
| **ratio** DF = | | | | |
| **ratio(PEG/SAXA = 0.8)** | 0.36 | 0.02 | 0.00 | 0.05 |
| **ratio(PEG/SAXA = 1.0)** | 0.22 | 0.02 | 0.00 | 0.05 |
| **ratio(PEG/SAXA = 1.2)** | −0.12 | 0.02 | 0.00 | 0.05 |
| **ratio(PEG/SAXA = 1.4)** | −0.46 | 0.03 | 0.00 | 0.06 |
| | | | | |
| | | | | |
| N = 177 | $Q^2$ = | 0.739 | Cond. no. = | 2.772 |
| DF = 170 | $R^2$ = | 0.761 | RSD = | 0.1894 |
| | $R^2$ adj. = | 0.753 | | |
| | | | Confidence = | 0.95 |
| | | | | |
| **ESCA~** | **Coeff. SC** | **Std. Err.** | **P** | **Conf. int(±)** |
| **Constant** | 0.53 | 0.02 | 0.00 | 0.04 |
| **Time Point** | 0.60 | 0.04 | 0.00 | 0.07 |
| **Temperature** | 0.82 | 0.03 | 0.00 | 0.07 |
| **Relative humidity** | 0.35 | 0.02 | 0.00 | 0.04 |
| | | | | |
| **ratio** DF = | | | | |
| **ratio(PEG/SAXA = 0.8)** | 0.13 | 0.03 | 0.00 | 0.06 |
| **ratio(PEG/SAXA = 1.0)** | 0.12 | 0.03 | 0.00 | 0.06 |
| **ratio(PEG/SAXA = 1.2)** | −0.01 | 0.03 | 0.69 | 0.06 |
| **ratio(PEG/SAXA = 1.4)** | −0.24 | 0.03 | 0.00 | 0.06 |
| | | | | |
| | | | | |
| N = 203 | $Q^2$ = | 0.811 | Cond. no. = | 2.626 |
| DF = 196 | $R^2$ = | 0.824 | RSD = | 0.2587 |
| | $R^2$ adj. = | 0.819 | | |
| | | | Confidence = | 0.95 |

**Table S12.** *Cont.*

| SFA~ | Coeff. SC | Std. Err. | P | Conf. int(±) |
|---|---|---|---|---|
| **Constant** | −0.44 | 0.02 | 0.00 | 0.04 |
| **Time Point** | 0.17 | 0.03 | 0.00 | 0.06 |
| **Temperature** | 0.32 | 0.03 | 0.00 | 0.06 |
| **Relative humidity** | 0.06 | 0.02 | 0.00 | 0.04 |
| | | | | |
| **ratio** | DF = | | | |
| **ratio(PEG/SAXA = 0.8)** | −0.60 | 0.03 | 0.00 | 0.06 |
| **ratio(PEG/SAXA = 1.0)** | −0.31 | 0.03 | 0.00 | 0.05 |
| **ratio(PEG/SAXA = 1.2)** | 0.17 | 0.03 | 0.00 | 0.05 |
| **ratio(PEG/SAXA = 1.4)** | 0.75 | 0.03 | 0.00 | 0.05 |
| | | | | |
| | | | | |
| N = 175 | $Q^2$ = | 0.846 | Cond. no. = | 2.888 |
| DF = 168 | $R^2$ = | 0.858 | RSD = | 0.2024 |
| | R2 adj. = | 0.853 | | |
| | | | Confidence = | 0.95 |
| | | | | |
| **pH** | **Coeff. SC** | **Std. Err.** | **P** | **Conf. int(±)** |
| **Constant** | 5.51 | 0.01 | 0.00 | 0.01 |
| **Time Point** | −0.01 | 0.01 | 0.33 | 0.02 |
| **Temperature** | −0.06 | 0.01 | 0.00 | 0.02 |
| **Relative humidity** | −0.04 | 0.01 | 0.00 | 0.01 |
| | | | | |
| **ratio** | DF = | | | |
| **ratio(PEG/SAXA = 0.8)** | 0.09 | 0.01 | 0.00 | 0.02 |
| **ratio(PEG/SAXA = 1.0)** | 0.07 | 0.01 | 0.00 | 0.02 |
| **ratio(PEG/SAXA = 1.2)** | 0.01 | 0.01 | 0.40 | 0.02 |
| **ratio(PEG/SAXA = 1.4)** | −0.17 | 0.01 | 0.00 | 0.02 |
| | | | | |
| | | | | |
| N = 180 | $Q^2$ = | 0.709 | Cond. no. = | 2.54 |
| DF = 173 | $R^2$ = | 0.731 | RSD = | 0.07068 |
| | $R^2$ adj. = | 0.721 | | |
| | | | Confidence = | 0.95 |

**Table S12.** *Cont.*

| aw | Coeff. SC | Std. Err. | P | Conf. int(±) |
|---|---|---|---|---|
| **Constant** | 0.29 | 0.00 | 0.00 | 0.00 |
| **Time Point** | −0.03 | 0.00 | 0.00 | 0.00 |
| **Temperature** | −0.00 | 0.00 | 0.06 | 0.00 |
| **Relative humidity** | 0.10 | 0.00 | 0.00 | 0.00 |
| | | | | |
| **ratio** | DF = | | | |
| **ratio(PEG/SAXA = 0.8)** | −0.01 | 0.00 | 0.00 | 0.00 |
| **ratio(PEG/SAXA = 1.0)** | −0.01 | 0.00 | 0.00 | 0.00 |
| **ratio(PEG/SAXA = 1.2)** | 0.00 | 0.00 | 0.02 | 0.00 |
| **ratio(PEG/SAXA = 1.4)** | 0.01 | 0.00 | 0.00 | 0.00 |
| | | | | |
| | | | | |
| N = 204 | $Q^2$ = | 0.960 | Cond. no. = | 2.61 |
| DF = 197 | $R^2$ = | 0.963 | RSD = | 0.01693 |
| | $R^2$ adj. = | 0.962 | | |
| | | | Confidence = | 0.95 |
| | | | | |
| **F~** | **Coeff. SC** | **Std. Err.** | **P** | **Conf. int(±)** |
| **Constant** | 1.35 | 0.03 | 0.00 | 0.07 |
| **Time Point** | −0.14 | 0.05 | 0.01 | 0.10 |
| **Temperature** | −0.51 | 0.05 | 0.00 | 0.10 |
| **Relative humidity** | −0.10 | 0.03 | 0.00 | 0.07 |
| | | | | |
| **ratio** | DF = | | | |
| **ratio(PEG/SAXA = 0.8)** | −0.32 | 0.05 | 0.00 | 0.09 |
| **ratio(PEG/SAXA = 1.0)** | −0.19 | 0.05 | 0.00 | 0.09 |
| **ratio(PEG/SAXA = 1.2)** | −0.01 | 0.05 | 0.91 | 0.09 |
| **ratio(PEG/SAXA = 1.4)** | 0.51 | 0.05 | 0.00 | 0.09 |
| | | | | |
| | | | | |
| N = 202 | $Q^2$ = | 0.541 | Cond. no. = | 2.609 |
| DF = 195 | $R^2$ = | 0.573 | RSD = | 0.382 |

**Table S12.** *Cont.*

| | | | | |
|---|---|---|---|---|
| | $R^2$ adj. = | 0.560 | | |
| | | | Confidence = | 0.95 |
| **FA~** | **Coeff. SC** | **Std. Err.** | **P** | **Conf. int(±)** |
| **Constant** | 2.89 | 0.02 | 0.00 | 0.03 |
| **Time Point** | 0.15 | 0.03 | 0.00 | 0.05 |
| **Temperature** | 0.12 | 0.03 | 0.00 | 0.05 |
| **Relative humidity** | 0.01 | 0.02 | 0.69 | 0.03 |
| | | | | |
| **ratio** DF = | | | | |
| **ratio(PEG/SAXA = 0.8)** | –0.37 | 0.02 | 0.00 | 0.05 |
| **ratio(PEG/SAXA = 1.0)** | –0.24 | 0.02 | 0.00 | 0.05 |
| **ratio(PEG/SAXA = 1.2)** | 0.07 | 0.02 | 0.01 | 0.05 |
| **ratio(PEG/SAXA = 1.4)** | 0.54 | 0.02 | 0.00 | 0.05 |
| | | | | |
| | | | | |
| N = 204 | $Q^2$ = | 0.746 | Cond. no. = | 2.61 |
| DF = 197 | $R^2$ = | 0.763 | RSD = | 0.2054 |
| | $R^2$ adj. = | 0.755 | | |
| | | | Confidence = | 0.95 |
| | | | | |
| **Coloration** | **Coeff. SC** | **Std. Err.** | **P** | **Conf. int(±)** |
| **Constant** | 157.38 | 1.34 | 0.00 | 2.65 |
| **Time Point** | –18.40 | 2.03 | 0.00 | 4.01 |
| **Temperature** | –33.54 | 1.92 | 0.00 | 3.79 |
| **Relative humidity** | –14.50 | 1.33 | 0.00 | 2.63 |
| | | | | |
| **ratio** DF = | | | | |
| **ratio(PEG/SAXA = 0.8)** | 1.18 | 1.88 | 0.53 | 3.72 |
| **ratio(PEG/SAXA = 1.0)** | –4.63 | 1.88 | 0.01 | 3.72 |
| **ratio(PEG/SAXA = 1.2)** | –8.75 | 1.88 | 0.00 | 3.72 |
| **ratio(PEG/SAXA = 1.4)** | 12.21 | 1.88 | 0.00 | 3.72 |
| | | | | |
| N = 180 | $Q^2$ = | 0.710 | Cond. no. = | 2.54 |

**Table S12.** *Cont.*

| DF = 173 | $R^2$ = | 0.733 | RSD = | 14.6 |
|---|---|---|---|---|
| | $R^2$ adj. = | 0.724 | | |
| | | | Confidence = | 0.95 |

**Table S13.** Correlation matrix.

| | 1 | 2 | 3 | 4 | 5 | 6 | 7 | 8 | 9 | 10 | 11 | 12 | 13 | 14 |
|---|---|---|---|---|---|---|---|---|---|---|---|---|---|---|
| | **Time Point** | **Temperature** | **Relative humidity** | **ratio(PEG/SAXA = 1.0)** | **ratio(PEG/SAXA = 1.2)** | **ratio(PEG/SAXA = 1.4)** | **ESCA~** | **SFA~** | **SCA~** | **pH** | **aw** | **F~** | **FA~** | **Coloration** |
| **Time Point** | 1.00 | −0.54 | 0.02 | 0.00 | 0.03 | 0.06 | 0.12 | 0.03 | 0.27 | 0.09 | −0.17 | 0.19 | 0.19 | 0.00 |
| **Temperature** | −0.54 | 1.00 | −0.05 | 0.00 | −0.04 | −0.04 | 0.53 | 0.07 | 0.15 | −0.30 | 0.03 | −0.51 | 0.07 | −0.59 |
| **Relative humidity** | 0.02 | −0.05 | 1.00 | 0.00 | −0.03 | −0.20 | 0.53 | −0.27 | −0.16 | 0.03 | 0.96 | −0.35 | −0.24 | −0.55 |
| **ratio(PEG/SAXA = 1.0)** | 0.00 | 0.00 | 0.00 | 1.00 | 0.51 | 0.58 | −0.01 | 0.04 | −0.13 | −0.10 | 0.01 | 0.09 | 0.14 | −0.08 |
| **ratio(PEG/SAXA = 1.2)** | 0.03 | −0.04 | −0.03 | 0.51 | 1.00 | 0.59 | −0.11 | 0.45 | −0.46 | −0.33 | 0.03 | 0.26 | 0.48 | −0.11 |
| **ratio(PEG/SAXA = 1.4)** | 0.06 | −0.04 | −0.20 | 0.58 | 0.59 | 1.00 | −0.21 | 0.73 | −0.58 | −0.61 | −0.15 | 0.42 | 0.72 | 0.06 |
| **ESCA~** | 0.12 | 0.53 | 0.53 | −0.01 | −0.11 | −0.21 | 1.00 | −0.17 | 0.40 | −0.10 | 0.45 | −0.54 | −0.05 | −0.83 |
| **SFA~** | 0.03 | 0.07 | −0.27 | 0.04 | 0.45 | 0.73 | −0.17 | 1.00 | −0.56 | −0.76 | −0.22 | 0.37 | 0.86 | −0.04 |
| **SCA~** | 0.27 | 0.15 | −0.16 | −0.13 | −0.46 | −0.58 | 0.40 | −0.56 | 1.00 | 0.44 | −0.29 | −0.27 | −0.37 | −0.09 |
| **pH** | 0.09 | −0.30 | 0.03 | −0.10 | −0.33 | −0.61 | −0.10 | −0.76 | 0.44 | 1.00 | −0.06 | −0.09 | −0.67 | 0.29 |
| **aw** | −0.17 | 0.03 | 0.96 | 0.01 | 0.03 | −0.15 | 0.45 | −0.22 | −0.29 | −0.06 | 1.00 | −0.37 | −0.24 | −0.51 |
| **F~** | 0.19 | −0.51 | −0.35 | 0.09 | 0.26 | 0.42 | −0.54 | 0.37 | −0.27 | −0.09 | −0.37 | 1.00 | 0.43 | 0.53 |
| **FA~** | 0.19 | 0.07 | −0.24 | 0.14 | 0.48 | 0.72 | −0.05 | 0.86 | −0.37 | −0.67 | −0.24 | 0.43 | 1.00 | −0.10 |
| **Coloration** | 0.00 | −0.59 | −0.55 | −0.08 | −0.11 | 0.06 | −0.83 | −0.04 | −0.09 | 0.29 | −0.51 | 0.53 | −0.10 | 1.00 |

### *3.4. Modelling of Degradation Kinetics*

Equations, experimental values, modelled values and determined rate constants for temperature of 40 °C and RH 10% used for additional model optimization are presented.

### *3.5. Equations*

```
METHOD RK4

STARTTIME=0
STOPTIME=200

DT=0.1

pH08=−570.45*(cFA08)+5.6703
pH10=−570.45*(cFA10)+5.6703
pH12=−570.45*(cFA12)+5.6703
pH14=−570.45*(cFA14)+5.6703

k1c=1
k2c=1
k3c=1
k4c=1
k5=1
k6=1
k7=1
k8=1

xS=1
xPEG=1

xPEGm08=xPEG
xPEGm10=xPEG*0.125/0.1
xPEGm12=xPEG*0.15/0.1
xPEGm14=xPEG*0.175/0.1

cPEGTOT008=0.0181611805
cPEG008=xPEGm08*cPEGTOT008

d/dt(cPEG08)=−k1c*cPEG08−k2c*cPEG08−k3c*cPEG08
```

```
d/dt(cGA08)=k1c*cPEG08
d/dt(cA08)=k2c*cPEG08
d/dt(cF08)=k3c*cPEG08−k4c*cF08
d/dt(cFA08)=k4c*cF08

init cPEG08=cPEG008
init cGA08=0
init cA08=0
init cF08=0
init cFA08=0

cSTOT008=0.0031704765
cS008=xS*cSTOT008

d/dt(cS08)=−k5*cS08−k7*cFA08*cS08+k8*cSF08
d/dt(cSA08)=k5*cS08−k6*cSA08
d/dt(cESA08)=k6*cSA08
d/dt(cSF08)=k7*cFA08*cS08−k8*cSF08

init cS08=cS008
init cSA08=0
init cESA08=0
init cSF08=0

cPEGTOT010=0.0227014756
cPEG010=xPEGm10*cPEGTOT010

d/dt(cPEG10)=−k1c*cPEG10−k2c*cPEG10−k3c*cPEG10
d/dt(cGA10)=k1c*cPEG10
d/dt(cA10)=k2c*cPEG10
d/dt(cF10)=k3c*cPEG10−k4c*cF10
d/dt(cFA10)=k4c*cF10

init cPEG10=cPEG010
```

```
init cGA10=0
init cA10=0
init cF10=0
init cFA10=0
cSTOT010=0.0031704765
cS010=xS*cSTOT010
d/dt(cS10)=−k5*((10^(pH10−14))/(10^(pH08−14)))*cS10−k7*((10^(−pH10))/(10^(−pH08)))*cFA10*cS10+k8*cSF10
d/dt(cSA10)=k5*((10^(pH10−14))/(10^(pH08−14)))*cS10−k6*((10^(pH10−14))/(10^(pH08−14)))*cSA10
d/dt(cESA10)=k6*((10^(pH10−14))/(10^(pH08−14)))*cSA10
d/dt(cSF10)=k7*((10^(−pH10))/(10^(−pH08)))*cFA10*cS10−k8*cSF10
init cS10=cS010
init cSA10=0
init cESA10=0
init cSF10=0
cPEGTOT012=0.0272417707
cPEG012=xPEGm12*cPEGTOT012
d/dt(cPEG12)=−k1c*cPEG12−k2c*cPEG12−k3c*cPEG12
d/dt(cGA12)=k1c*cPEG12
d/dt(cA12)=k2c*cPEG12
d/dt(cF12)=k3c*cPEG12−k4c*cF12
d/dt(cFA12)=k4c*cF12
init cPEG12=cPEG012
init cGA12=0
init cA12=0
init cF12=0
init cFA12=0
cSTOT012=0.0031704765
cS012=xS*cSTOT012
d/dt(cS12)=−k5*((10^(pH12−14))/(10^(pH08−14)))*cS12−k7*((10^(−pH12))/(10^(−pH08)))*cFA12*cS12+k8*cSF12
d/dt(cSA12)=k5*((10^(pH12−14))/(10^(pH08−14)))*cS12−k6*((10^(pH12−14))/(10^(pH08−14)))*cSA12
d/dt(cESA12)=k6*((10^(pH12−14))/(10^(pH08−14)))*cSA12
d/dt(cSF12)=k7*((10^(−pH12))/(10^(−pH08)))*cFA12*cS12−k8*cSF12
init cS12=cS012
init cSA12=0
```

```
init cESA12=0
init cSF12=0
cPEGTOT014=0.0317820658
cPEG014=xPEGm14*cPEGTOT014

d/dt(cPEG14)=−k1c*cPEG14−k2c*cPEG14−k3c*cPEG14
d/dt(cGA14)=k1c*cPEG14
d/dt(cA14)=k2c*cPEG14
d/dt(cF14)=k3c*cPEG14−k4c*cF14
d/dt(cFA14)=k4c*cF14

init cPEG14=cPEG014
init cGA14=0
init cA14=0
init cF14=0
init cFA14=0

cSTOT014=0.0031704765
cS014=xS*cSTOT014

d/dt(cS14)=−k5*((10^(pH14−14))/(10^(pH08−14)))*cS14−k7*((10^(−pH14))/(10^(−pH08)))*cFA14*cS14+k8*cSF14
d/dt(cSA14)=k5*((10^(pH14−14))/(10^(pH08−14)))*cS14−k6*((10^(pH14−14))/(10^(pH08−14)))*cSA14
d/dt(cESA14)=k6*((10^(pH14−14))/(10^(pH08−14)))*cSA14
d/dt(cSF14)=k7*((10^(−pH14))/(10^(−pH08)))*cFA14*cS14−k8*cSF14

init cS14=cS014
init cSA14=0
init cESA14=0
init cSF14=0
```

Experimental data

**Table S14.** Imported dataset for organic impurities (values in mol/$g_{saxa}$).

| | TimePoint (days) | GA | A | F | FA |
|---|---|---|---|---|---|
| **0.8** | 30 | 0.0000288345 | 0.0000000000 | 0.0000086330 | 0.0000440405 |
| | 60 | 0.0000288906 | 0.0000000000 | 0.0000102902 | 0.0000447755 |
| | 90 | 0.0000285908 | 0.0000000000 | 0.0000094031 | 0.0000454649 |
| | 120 | 0.0000296821 | 0.0000000000 | 0.0000055805 | 0.0000641012 |
| | 150 | 0.0000309696 | 0.0000022343 | 0.0000056773 | 0.0000601126 |
| | 180 | 0.0000307233 | 0.0000021840 | 0.0000082824 | 0.0000623029 |
| **1** | 30 | 0.0000300946 | 0.0000000000 | 0.0000130329 | 0.0000546879 |
| | 60 | 0.0000291543 | 0.0000000000 | 0.0000142177 | 0.0000563260 |
| | 90 | 0.0000305892 | 0.0000000000 | 0.0000131391 | 0.0000618047 |
| | 120 | 0.0000345007 | 0.0000000000 | 0.0000096577 | 0.0001011784 |
| | 150 | 0.0000326029 | 0.0000016783 | 0.0000045257 | 0.0001430299 |
| | 180 | 0.0000315294 | 0.0000023071 | 0.0000097536 | 0.0000860655 |
| **1.2** | 30 | 0.0000319194 | 0.0000028043 | 0.0000376531 | 0.0001735970 |
| | 60 | 0.0000320094 | 0.0000037533 | 0.0000338475 | 0.0002029180 |
| | 90 | 0.0000321056 | 0.0000034046 | 0.0000259072 | 0.0002155883 |
| | 120 | 0.0000391023 | 0.0000025601 | 0.0000213116 | 0.0003597215 |
| | 150 | 0.0000344614 | 0.0000027292 | 0.0000159765 | 0.0002493824 |
| | 180 | 0.0000333889 | 0.0000030509 | 0.0000166734 | 0.0002943538 |
| **1.4** | 30 | 0.0000352133 | 0.0000033385 | 0.0000455659 | 0.0002871912 |
| | 60 | 0.0000356637 | 0.0000034776 | 0.0000338841 | 0.0003473679 |
| | 90 | 0.0000358764 | 0.0000033827 | 0.0000265859 | 0.0003693140 |
| | 120 | 0.0000462426 | 0.0000035750 | 0.0000216307 | 0.0005796135 |
| | 150 | 0.0000355406 | 0.0000025717 | 0.0000137465 | 0.0004226970 |
| | 180 | 0.0000392171 | 0.0000022911 | 0.0000134461 | 0.0005042563 |

**Table S15.** Imported dataset for saxagliptin degradation products (values in mol/$g_{saxa}$).

| PEG/SAXA ratio | TimePoint (days) | SCA | ESCA | SFA |
|---|---|---|---|---|
| **0.8** | 30 | 0.0000090363 | 0.0000063778 | 0.0000000000 |
| | 60 | 0.0000194300 | 0.0000210633 | 0.0000000000 |
| | 90 | 0.0000288421 | 0.0000326124 | 0.0000000000 |
| | 120 | 0.0000348269 | 0.0000467544 | 0.0000000000 |
| | 150 | 0.0000412375 | 0.0000601218 | 0.0000014596 |
| | 180 | 0.0000454932 | 0.0000765514 | 0.0000021721 |
| **1** | 30 | 0.0000067507 | 0.0000052179 | 0.0000017727 |
| | 60 | 0.0000140392 | 0.0000176348 | 0.0000022965 |
| | 90 | 0.0000218035 | 0.0000265181 | 0.0000021741 |
| | 120 | 0.0000257447 | 0.0000373596 | 0.0000023429 |
| | 150 | 0.0000316957 | 0.0000512749 | 0.0000030700 |
| | 180 | 0.0000360807 | 0.0000658352 | 0.0000044738 |
| **1.2** | 30 | 0.0000026306 | 0.0000022160 | 0.0000139546 |
| | 60 | 0.0000070698 | 0.0000080635 | 0.0000198998 |
| | 90 | 0.0000104400 | 0.0000142243 | 0.0000167838 |
| | 120 | 0.0000123231 | 0.0000245514 | 0.0000159019 |
| | 150 | 0.0000154855 | 0.0000356448 | 0.0000180762 |
| | 180 | 0.0000163778 | 0.0000447367 | 0.0000216494 |
| **1.4** | 30 | 0.0000020861 | 0.0000020375 | 0.0000279559 |
| | 60 | 0.0000046133 | 0.0000105469 | 0.0000411142 |
| | 90 | 0.0000056227 | 0.0000183767 | 0.0000359713 |
| | 120 | 0.0000060124 | 0.0000299248 | 0.0000360929 |
| | 150 | 0.0000069010 | 0.0000417796 | 0.0000388216 |
| | 180 | 0.0000061933 | 0.0000533430 | 0.0000404983 |

## 4. Modelled Data

**Table S16.** Modelled data for organic impurities (values in mol/$g_{saxa}$).

| TIME (days) | cFA08:1 | cFA10:1 | cFA12:1 | cFA14:1 | cGA08:1 | cA08:1 | cF08:1 | cGA10:1 | cA10:1 | cF10:1 | cGA12:1 | cA12:1 | cF12:1 | cGA14:1 | cA14:1 | cF14:1 |
|---|---|---|---|---|---|---|---|---|---|---|---|---|---|---|---|---|
| 0 | 0 | 0 | 0 | 0 | 0 | 0 | 0 | 0 | 0 | 0 | 0 | 0 | 0 | 0 | 0 | 0 |
| 1 | 1.81E−06 | 2.83E−06 | 4.08E−06 | 5.55E−06 | 7.84E−06 | 5.29E−07 | 5.74E−05 | 1.23E−05 | 8.26E−07 | 8.97E−05 | 1.76E−05 | 1.19E−06 | 1.29E−04 | 2.40E−05 | 1.62E−06 | 1.76E−04 |
| 2 | 5.77E−06 | 9.02E−06 | 1.30E−05 | 1.77E−05 | 1.16E−05 | 7.79E−07 | 8.15E−05 | 1.81E−05 | 1.22E−06 | 1.27E−04 | 2.60E−05 | 1.75E−06 | 1.83E−04 | 3.54E−05 | 2.39E−06 | 2.50E−04 |
| 3 | 1.06E−05 | 1.65E−05 | 2.38E−05 | 3.24E−05 | 1.33E−05 | 8.97E−07 | 9.00E−05 | 2.08E−05 | 1.40E−06 | 1.41E−04 | 3.00E−05 | 2.02E−06 | 2.02E−04 | 4.08E−05 | 2.75E−06 | 2.76E−04 |
| 4 | 1.56E−05 | 2.44E−05 | 3.52E−05 | 4.79E−05 | 1.42E−05 | 9.54E−07 | 9.12E−05 | 2.21E−05 | 1.49E−06 | 1.43E−04 | 3.18E−05 | 2.15E−06 | 2.05E−04 | 4.33E−05 | 2.92E−06 | 2.79E−04 |
| 5 | 2.07E−05 | 3.23E−05 | 4.65E−05 | 6.32E−05 | 1.45E−05 | 9.80E−07 | 8.92E−05 | 2.27E−05 | 1.53E−06 | 1.39E−04 | 3.27E−05 | 2.21E−06 | 2.01E−04 | 4.45E−05 | 3.00E−06 | 2.73E−04 |
| 6 | 2.55E−05 | 3.99E−05 | 5.74E−05 | 7.81E−05 | 1.47E−05 | 9.93E−07 | 8.57E−05 | 2.30E−05 | 1.55E−06 | 1.34E−04 | 3.31E−05 | 2.23E−06 | 1.93E−04 | 4.51E−05 | 3.04E−06 | 2.63E−04 |
| 7 | 3.02E−05 | 4.71E−05 | 6.79E−05 | 9.24E−05 | 1.48E−05 | 9.99E−07 | 8.18E−05 | 2.32E−05 | 1.56E−06 | 1.28E−04 | 3.33E−05 | 2.25E−06 | 1.84E−04 | 4.54E−05 | 3.06E−06 | 2.50E−04 |
| 8 | 3.46E−05 | 5.40E−05 | 7.78E−05 | 1.06E−04 | 1.49E−05 | 1.00E−06 | 7.76E−05 | 2.32E−05 | 1.56E−06 | 1.21E−04 | 3.34E−05 | 2.25E−06 | 1.75E−04 | 4.55E−05 | 3.07E−06 | 2.38E−04 |
| 9 | 3.88E−05 | 6.06E−05 | 8.73E−05 | 1.19E−04 | 1.49E−05 | 1.00E−06 | 7.36E−05 | 2.33E−05 | 1.57E−06 | 1.15E−04 | 3.35E−05 | 2.26E−06 | 1.66E−04 | 4.56E−05 | 3.07E−06 | 2.25E−04 |
| 10 | 4.28E−05 | 6.68E−05 | 9.62E−05 | 1.31E−04 | 1.49E−05 | 1.00E−06 | 6.97E−05 | 2.33E−05 | 1.57E−06 | 1.09E−04 | 3.35E−05 | 2.26E−06 | 1.57E−04 | 4.56E−05 | 3.07E−06 | 2.13E−04 |
| 11 | 4.65E−05 | 7.27E−05 | 1.05E−04 | 1.42E−04 | 1.49E−05 | 1.00E−06 | 6.60E−05 | 2.33E−05 | 1.57E−06 | 1.03E−04 | 3.35E−05 | 2.26E−06 | 1.48E−04 | 4.56E−05 | 3.07E−06 | 2.02E−04 |
| 12 | 5.01E−05 | 7.83E−05 | 1.13E−04 | 1.53E−04 | 1.49E−05 | 1.00E−06 | 6.24E−05 | 2.33E−05 | 1.57E−06 | 9.75E−05 | 3.35E−05 | 2.26E−06 | 1.40E−04 | 4.56E−05 | 3.07E−06 | 1.91E−04 |
| 13 | 5.35E−05 | 8.35E−05 | 1.20E−04 | 1.64E−04 | 1.49E−05 | 1.00E−06 | 5.90E−05 | 2.33E−05 | 1.57E−06 | 9.23E−05 | 3.35E−05 | 2.26E−06 | 1.33E−04 | 4.56E−05 | 3.07E−06 | 1.81E−04 |
| 14 | 5.66E−05 | 8.85E−05 | 1.27E−04 | 1.73E−04 | 1.49E−05 | 1.00E−06 | 5.59E−05 | 2.33E−05 | 1.57E−06 | 8.73E−05 | 3.35E−05 | 2.26E−06 | 1.26E−04 | 4.56E−05 | 3.07E−06 | 1.71E−04 |
| 15 | 5.97E−05 | 9.32E−05 | 1.34E−04 | 1.83E−04 | 1.49E−05 | 1.00E−06 | 5.28E−05 | 2.33E−05 | 1.57E−06 | 8.26E−05 | 3.35E−05 | 2.26E−06 | 1.19E−04 | 4.56E−05 | 3.07E−06 | 1.62E−04 |
| 16 | 6.25E−05 | 9.77E−05 | 1.41E−04 | 1.91E−04 | 1.49E−05 | 1.00E−06 | 5.00E−05 | 2.33E−05 | 1.57E−06 | 7.81E−05 | 3.35E−05 | 2.26E−06 | 1.12E−04 | 4.56E−05 | 3.07E−06 | 1.53E−04 |
| 17 | 6.52E−05 | 1.02E−04 | 1.47E−04 | 2.00E−04 | 1.49E−05 | 1.00E−06 | 4.73E−05 | 2.33E−05 | 1.57E−06 | 7.39E−05 | 3.35E−05 | 2.26E−06 | 1.06E−04 | 4.56E−05 | 3.07E−06 | 1.45E−04 |
| 18 | 6.78E−05 | 1.06E−04 | 1.52E−04 | 2.08E−04 | 1.49E−05 | 1.00E−06 | 4.47E−05 | 2.33E−05 | 1.57E−06 | 6.99E−05 | 3.35E−05 | 2.26E−06 | 1.01E−04 | 4.56E−05 | 3.07E−06 | 1.37E−04 |
| 19 | 7.02E−05 | 1.10E−04 | 1.58E−04 | 2.15E−04 | 1.49E−05 | 1.00E−06 | 4.23E−05 | 2.33E−05 | 1.57E−06 | 6.61E−05 | 3.35E−05 | 2.26E−06 | 9.52E−05 | 4.56E−05 | 3.07E−06 | 1.30E−04 |
| 20 | 7.25E−05 | 1.13E−04 | 1.63E−04 | 2.22E−04 | 1.49E−05 | 1.00E−06 | 4.00E−05 | 2.33E−05 | 1.57E−06 | 6.26E−05 | 3.35E−05 | 2.26E−06 | 9.01E−05 | 4.56E−05 | 3.07E−06 | 1.23E−04 |
| 21 | 7.46E−05 | 1.17E−04 | 1.68E−04 | 2.29E−04 | 1.49E−05 | 1.00E−06 | 3.79E−05 | 2.33E−05 | 1.57E−06 | 5.92E−05 | 3.35E−05 | 2.26E−06 | 8.52E−05 | 4.56E−05 | 3.07E−06 | 1.16E−04 |

**Table S16.** *Cont.*

| TIME (days) | cFA08:1 | cFA10:1 | cFA12:1 | cFA14:1 | cGA08:1 | cA08:1 | cF08:1 | cGA10:1 | cA10:1 | cF10:1 | cGA12:1 | cA12:1 | cF12:1 | cGA14:1 | cA14:1 | cF14:1 |
|---|---|---|---|---|---|---|---|---|---|---|---|---|---|---|---|---|
| 22 | 7.67E−05 | 1.20E−04 | 1.73E−04 | 2.35E−04 | 1.49E−05 | 1.00E−06 | 3.58E−05 | 2.33E−05 | 1.57E−06 | 5.60E−05 | 3.35E−05 | 2.26E−06 | 8.06E−05 | 4.56E−05 | 3.07E−06 | 1.10E−04 |
| 23 | 7.86E−05 | 1.23E−04 | 1.77E−04 | 2.41E−04 | 1.49E−05 | 1.00E−06 | 3.39E−05 | 2.33E−05 | 1.57E−06 | 5.30E−05 | 3.35E−05 | 2.26E−06 | 7.63E−05 | 4.56E−05 | 3.07E−06 | 1.04E−04 |
| 24 | 8.04E−05 | 1.26E−04 | 1.81E−04 | 2.46E−04 | 1.49E−05 | 1.00E−06 | 3.21E−05 | 2.33E−05 | 1.57E−06 | 5.01E−05 | 3.35E−05 | 2.26E−06 | 7.21E−05 | 4.56E−05 | 3.07E−06 | 9.82E−05 |
| 25 | 8.22E−05 | 1.28E−04 | 1.85E−04 | 2.52E−04 | 1.49E−05 | 1.00E−06 | 3.03E−05 | 2.33E−05 | 1.57E−06 | 4.74E−05 | 3.35E−05 | 2.26E−06 | 6.82E−05 | 4.56E−05 | 3.07E−06 | 9.29E−05 |
| 26 | 8.38E−05 | 1.31E−04 | 1.89E−04 | 2.57E−04 | 1.49E−05 | 1.00E−06 | 2.87E−05 | 2.33E−05 | 1.57E−06 | 4.48E−05 | 3.35E−05 | 2.26E−06 | 6.46E−05 | 4.56E−05 | 3.07E−06 | 8.79E−05 |
| 27 | 8.54E−05 | 1.33E−04 | 1.92E−04 | 2.61E−04 | 1.49E−05 | 1.00E−06 | 2.71E−05 | 2.33E−05 | 1.57E−06 | 4.24E−05 | 3.35E−05 | 2.26E−06 | 6.11E−05 | 4.56E−05 | 3.07E−06 | 8.31E−05 |
| 28 | 8.68E−05 | 1.36E−04 | 1.95E−04 | 2.66E−04 | 1.49E−05 | 1.00E−06 | 2.57E−05 | 2.33E−05 | 1.57E−06 | 4.01E−05 | 3.35E−05 | 2.26E−06 | 5.78E−05 | 4.56E−05 | 3.07E−06 | 7.86E−05 |
| 29 | 8.82E−05 | 1.38E−04 | 1.98E−04 | 2.70E−04 | 1.49E−05 | 1.00E−06 | 2.43E−05 | 2.33E−05 | 1.57E−06 | 3.80E−05 | 3.35E−05 | 2.26E−06 | 5.47E−05 | 4.56E−05 | 3.07E−06 | 7.44E−05 |
| 30 | 8.95E−05 | 1.40E−04 | 2.01E−04 | 2.74E−04 | 1.49E−05 | 1.00E−06 | 2.30E−05 | 2.33E−05 | 1.57E−06 | 3.59E−05 | 3.35E−05 | 2.26E−06 | 5.17E−05 | 4.56E−05 | 3.07E−06 | 7.04E−05 |
| 31 | 9.08E−05 | 1.42E−04 | 2.04E−04 | 2.78E−04 | 1.49E−05 | 1.00E−06 | 2.17E−05 | 2.33E−05 | 1.57E−06 | 3.40E−05 | 3.35E−05 | 2.26E−06 | 4.89E−05 | 4.56E−05 | 3.07E−06 | 6.66E−05 |
| 32 | 9.19E−05 | 1.44E−04 | 2.07E−04 | 2.82E−04 | 1.49E−05 | 1.00E−06 | 2.06E−05 | 2.33E−05 | 1.57E−06 | 3.21E−05 | 3.35E−05 | 2.26E−06 | 4.63E−05 | 4.56E−05 | 3.07E−06 | 6.30E−05 |
| 33 | 9.31E−05 | 1.45E−04 | 2.09E−04 | 2.85E−04 | 1.49E−05 | 1.00E−06 | 1.95E−05 | 2.33E−05 | 1.57E−06 | 3.04E−05 | 3.35E−05 | 2.26E−06 | 4.38E−05 | 4.56E−05 | 3.07E−06 | 5.96E−05 |
| 34 | 9.41E−05 | 1.47E−04 | 2.12E−04 | 2.88E−04 | 1.49E−05 | 1.00E−06 | 1.84E−05 | 2.33E−05 | 1.57E−06 | 2.88E−05 | 3.35E−05 | 2.26E−06 | 4.14E−05 | 4.56E−05 | 3.07E−06 | 5.64E−05 |
| 35 | 9.51E−05 | 1.49E−04 | 2.14E−04 | 2.91E−04 | 1.49E−05 | 1.00E−06 | 1.74E−05 | 2.33E−05 | 1.57E−06 | 2.72E−05 | 3.35E−05 | 2.26E−06 | 3.92E−05 | 4.56E−05 | 3.07E−06 | 5.33E−05 |
| 36 | 9.60E−05 | 1.50E−04 | 2.16E−04 | 2.94E−04 | 1.49E−05 | 1.00E−06 | 1.65E−05 | 2.33E−05 | 1.57E−06 | 2.57E−05 | 3.35E−05 | 2.26E−06 | 3.71E−05 | 4.56E−05 | 3.07E−06 | 5.04E−05 |
| 37 | 9.69E−05 | 1.51E−04 | 2.18E−04 | 2.97E−04 | 1.49E−05 | 1.00E−06 | 1.56E−05 | 2.33E−05 | 1.57E−06 | 2.43E−05 | 3.35E−05 | 2.26E−06 | 3.51E−05 | 4.56E−05 | 3.07E−06 | 4.77E−05 |
| 38 | 9.78E−05 | 1.53E−04 | 2.20E−04 | 2.99E−04 | 1.49E−05 | 1.00E−06 | 1.47E−05 | 2.33E−05 | 1.57E−06 | 2.30E−05 | 3.35E−05 | 2.26E−06 | 3.32E−05 | 4.56E−05 | 3.07E−06 | 4.51E−05 |
| 39 | 9.86E−05 | 1.54E−04 | 2.22E−04 | 3.02E−04 | 1.49E−05 | 1.00E−06 | 1.39E−05 | 2.33E−05 | 1.57E−06 | 2.18E−05 | 3.35E−05 | 2.26E−06 | 3.14E−05 | 4.56E−05 | 3.07E−06 | 4.27E−05 |
| 40 | 9.93E−05 | 1.55E−04 | 2.23E−04 | 3.04E−04 | 1.49E−05 | 1.00E−06 | 1.32E−05 | 2.33E−05 | 1.57E−06 | 2.06E−05 | 3.35E−05 | 2.26E−06 | 2.97E−05 | 4.56E−05 | 3.07E−06 | 4.04E−05 |
| 41 | 1.00E−04 | 1.56E−04 | 2.25E−04 | 3.06E−04 | 1.49E−05 | 1.00E−06 | 1.25E−05 | 2.33E−05 | 1.57E−06 | 1.95E−05 | 3.35E−05 | 2.26E−06 | 2.81E−05 | 4.56E−05 | 3.07E−06 | 3.82E−05 |
| 42 | 1.01E−04 | 1.57E−04 | 2.27E−04 | 3.08E−04 | 1.49E−05 | 1.00E−06 | 1.18E−05 | 2.33E−05 | 1.57E−06 | 1.84E−05 | 3.35E−05 | 2.26E−06 | 2.66E−05 | 4.56E−05 | 3.07E−06 | 3.61E−05 |
| 43 | 1.01E−04 | 1.58E−04 | 2.28E−04 | 3.10E−04 | 1.49E−05 | 1.00E−06 | 1.12E−05 | 2.33E−05 | 1.57E−06 | 1.74E−05 | 3.35E−05 | 2.26E−06 | 2.51E−05 | 4.56E−05 | 3.07E−06 | 3.42E−05 |
| 44 | 1.02E−04 | 1.59E−04 | 2.29E−04 | 3.12E−04 | 1.49E−05 | 1.00E−06 | 1.06E−05 | 2.33E−05 | 1.57E−06 | 1.65E−05 | 3.35E−05 | 2.26E−06 | 2.38E−05 | 4.56E−05 | 3.07E−06 | 3.23E−05 |

**Table S16.** *Cont.*

| TIME (days) | cFA08:1 | cFA10:1 | cFA12:1 | cFA14:1 | cGA08:1 | cA08:1 | cF08:1 | cGA10:1 | cA10:1 | cF10:1 | cGA12:1 | cA12:1 | cF12:1 | cGA14:1 | cA14:1 | cF14:1 |
|---|---|---|---|---|---|---|---|---|---|---|---|---|---|---|---|---|
| 45 | 1.03E−04 | 1.60E−04 | 2.31E−04 | 3.14E−04 | 1.49E−05 | 1.00E−06 | 9.99E−06 | 2.33E−05 | 1.57E−06 | 1.56E−05 | 3.35E−05 | 2.26E−06 | 2.25E−05 | 4.56E−05 | 3.07E−06 | 3.06E−05 |
| 46 | 1.03E−04 | 1.61E−04 | 2.32E−04 | 3.16E−04 | 1.49E−05 | 1.00E−06 | 9.45E−06 | 2.33E−05 | 1.57E−06 | 1.48E−05 | 3.35E−05 | 2.26E−06 | 2.13E−05 | 4.56E−05 | 3.07E−06 | 2.89E−05 |
| 47 | 1.04E−04 | 1.62E−04 | 2.33E−04 | 3.17E−04 | 1.49E−05 | 1.00E−06 | 8.94E−06 | 2.33E−05 | 1.57E−06 | 1.40E−05 | 3.35E−05 | 2.26E−06 | 2.01E−05 | 4.56E−05 | 3.07E−06 | 2.74E−05 |
| 48 | 1.04E−04 | 1.63E−04 | 2.34E−04 | 3.19E−04 | 1.49E−05 | 1.00E−06 | 8.46E−06 | 2.33E−05 | 1.57E−06 | 1.32E−05 | 3.35E−05 | 2.26E−06 | 1.90E−05 | 4.56E−05 | 3.07E−06 | 2.59E−05 |
| 49 | 1.05E−04 | 1.63E−04 | 2.35E−04 | 3.20E−04 | 1.49E−05 | 1.00E−06 | 8.00E−06 | 2.33E−05 | 1.57E−06 | 1.25E−05 | 3.35E−05 | 2.26E−06 | 1.80E−05 | 4.56E−05 | 3.07E−06 | 2.45E−05 |
| 50 | 1.05E−04 | 1.64E−04 | 2.36E−04 | 3.21E−04 | 1.49E−05 | 1.00E−06 | 7.57E−06 | 2.33E−05 | 1.57E−06 | 1.18E−05 | 3.35E−05 | 2.26E−06 | 1.70E−05 | 4.56E−05 | 3.07E−06 | 2.32E−05 |
| 51 | 1.05E−04 | 1.65E−04 | 2.37E−04 | 3.23E−04 | 1.49E−05 | 1.00E−06 | 7.16E−06 | 2.33E−05 | 1.57E−06 | 1.12E−05 | 3.35E−05 | 2.26E−06 | 1.61E−05 | 4.56E−05 | 3.07E−06 | 2.19E−05 |
| 52 | 1.06E−04 | 1.65E−04 | 2.38E−04 | 3.24E−04 | 1.49E−05 | 1.00E−06 | 6.77E−06 | 2.33E−05 | 1.57E−06 | 1.06E−05 | 3.35E−05 | 2.26E−06 | 1.52E−05 | 4.56E−05 | 3.07E−06 | 2.07E−05 |
| 53 | 1.06E−04 | 1.66E−04 | 2.39E−04 | 3.25E−04 | 1.49E−05 | 1.00E−06 | 6.41E−06 | 2.33E−05 | 1.57E−06 | 1.00E−05 | 3.35E−05 | 2.26E−06 | 1.44E−05 | 4.56E−05 | 3.07E−06 | 1.96E−05 |
| 54 | 1.06E−04 | 1.66E−04 | 2.40E−04 | 3.26E−04 | 1.49E−05 | 1.00E−06 | 6.06E−06 | 2.33E−05 | 1.57E−06 | 9.47E−06 | 3.35E−05 | 2.26E−06 | 1.36E−05 | 4.56E−05 | 3.07E−06 | 1.86E−05 |
| 55 | 1.07E−04 | 1.67E−04 | 2.40E−04 | 3.27E−04 | 1.49E−05 | 1.00E−06 | 5.73E−06 | 2.33E−05 | 1.57E−06 | 8.96E−06 | 3.35E−05 | 2.26E−06 | 1.29E−05 | 4.56E−05 | 3.07E−06 | 1.76E−05 |
| 56 | 1.07E−04 | 1.67E−04 | 2.41E−04 | 3.28E−04 | 1.49E−05 | 1.00E−06 | 5.43E−06 | 2.33E−05 | 1.57E−06 | 8.48E−06 | 3.35E−05 | 2.26E−06 | 1.22E−05 | 4.56E−05 | 3.07E−06 | 1.66E−05 |
| 57 | 1.07E−04 | 1.68E−04 | 2.42E−04 | 3.29E−04 | 1.49E−05 | 1.00E−06 | 5.13E−06 | 2.33E−05 | 1.57E−06 | 8.02E−06 | 3.35E−05 | 2.26E−06 | 1.15E−05 | 4.56E−05 | 3.07E−06 | 1.57E−05 |
| 58 | 1.08E−04 | 1.68E−04 | 2.42E−04 | 3.30E−04 | 1.49E−05 | 1.00E−06 | 4.86E−06 | 2.33E−05 | 1.57E−06 | 7.59E−06 | 3.35E−05 | 2.26E−06 | 1.09E−05 | 4.56E−05 | 3.07E−06 | 1.49E−05 |
| 59 | 1.08E−04 | 1.69E−04 | 2.43E−04 | 3.31E−04 | 1.49E−05 | 1.00E−06 | 4.59E−06 | 2.33E−05 | 1.57E−06 | 7.18E−06 | 3.35E−05 | 2.26E−06 | 1.03E−05 | 4.56E−05 | 3.07E−06 | 1.41E−05 |
| 60 | 1.08E−04 | 1.69E−04 | 2.43E−04 | 3.31E−04 | 1.49E−05 | 1.00E−06 | 4.34E−06 | 2.33E−05 | 1.57E−06 | 6.79E−06 | 3.35E−05 | 2.26E−06 | 9.78E−06 | 4.56E−05 | 3.07E−06 | 1.33E−05 |
| 61 | 1.08E−04 | 1.69E−04 | 2.44E−04 | 3.32E−04 | 1.49E−05 | 1.00E−06 | 4.11E−06 | 2.33E−05 | 1.57E−06 | 6.42E−06 | 3.35E−05 | 2.26E−06 | 9.25E−06 | 4.56E−05 | 3.07E−06 | 1.26E−05 |
| 62 | 1.09E−04 | 1.70E−04 | 2.44E−04 | 3.33E−04 | 1.49E−05 | 1.00E−06 | 3.89E−06 | 2.33E−05 | 1.57E−06 | 6.08E−06 | 3.35E−05 | 2.26E−06 | 8.75E−06 | 4.56E−05 | 3.07E−06 | 1.19E−05 |
| 63 | 1.09E−04 | 1.70E−04 | 2.45E−04 | 3.33E−04 | 1.49E−05 | 1.00E−06 | 3.68E−06 | 2.33E−05 | 1.57E−06 | 5.75E−06 | 3.35E−05 | 2.26E−06 | 8.28E−06 | 4.56E−05 | 3.07E−06 | 1.13E−05 |
| 64 | 1.09E−04 | 1.70E−04 | 2.45E−04 | 3.34E−04 | 1.49E−05 | 1.00E−06 | 3.48E−06 | 2.33E−05 | 1.57E−06 | 5.44E−06 | 3.35E−05 | 2.26E−06 | 7.83E−06 | 4.56E−05 | 3.07E−06 | 1.07E−05 |
| 65 | 1.09E−04 | 1.71E−04 | 2.46E−04 | 3.34E−04 | 1.49E−05 | 1.00E−06 | 3.29E−06 | 2.33E−05 | 1.57E−06 | 5.14E−06 | 3.35E−05 | 2.26E−06 | 7.41E−06 | 4.56E−05 | 3.07E−06 | 1.01E−05 |
| 66 | 1.09E−04 | 1.71E−04 | 2.46E−04 | 3.35E−04 | 1.49E−05 | 1.00E−06 | 3.11E−06 | 2.33E−05 | 1.57E−06 | 4.87E−06 | 3.35E−05 | 2.26E−06 | 7.01E−06 | 4.56E−05 | 3.07E−06 | 9.54E−06 |
| 67 | 1.10E−04 | 1.71E−04 | 2.47E−04 | 3.36E−04 | 1.49E−05 | 1.00E−06 | 2.95E−06 | 2.33E−05 | 1.57E−06 | 4.60E−06 | 3.35E−05 | 2.26E−06 | 6.63E−06 | 4.56E−05 | 3.07E−06 | 9.02E−06 |

**Table S16.** *Cont.*

| TIME (days) | cFA08:1 | cFA10:1 | cFA12:1 | cFA14:1 | cGA08:1 | cA08:1 | cF08:1 | cGA10:1 | cA10:1 | cF10:1 | cGA12:1 | cA12:1 | cF12:1 | cGA14:1 | cA14:1 | cF14:1 |
|---|---|---|---|---|---|---|---|---|---|---|---|---|---|---|---|---|
| 68 | 1.10E−04 | 1.71E−04 | 2.47E−04 | 3.36E−04 | 1.49E−05 | 1.00E−06 | 2.79E−06 | 2.33E−05 | 1.57E−06 | 4.35E−06 | 3.35E−05 | 2.26E−06 | 6.27E−06 | 4.56E−05 | 3.07E−06 | 8.53E−06 |
| 69 | 1.10E−04 | 1.72E−04 | 2.47E−04 | 3.37E−04 | 1.49E−05 | 1.00E−06 | 2.64E−06 | 2.33E−05 | 1.57E−06 | 4.12E−06 | 3.35E−05 | 2.26E−06 | 5.93E−06 | 4.56E−05 | 3.07E−06 | 8.07E−06 |
| 70 | 1.10E−04 | 1.72E−04 | 2.48E−04 | 3.37E−04 | 1.49E−05 | 1.00E−06 | 2.49E−06 | 2.33E−05 | 1.57E−06 | 3.90E−06 | 3.35E−05 | 2.26E−06 | 5.61E−06 | 4.56E−05 | 3.07E−06 | 7.64E−06 |
| 71 | 1.10E−04 | 1.72E−04 | 2.48E−04 | 3.37E−04 | 1.49E−05 | 1.00E−06 | 2.36E−06 | 2.33E−05 | 1.57E−06 | 3.69E−06 | 3.35E−05 | 2.26E−06 | 5.31E−06 | 4.56E−05 | 3.07E−06 | 7.22E−06 |
| 72 | 1.10E−04 | 1.72E−04 | 2.48E−04 | 3.38E−04 | 1.49E−05 | 1.00E−06 | 2.23E−06 | 2.33E−05 | 1.57E−06 | 3.49E−06 | 3.35E−05 | 2.26E−06 | 5.02E−06 | 4.56E−05 | 3.07E−06 | 6.83E−06 |
| 73 | 1.10E−04 | 1.73E−04 | 2.48E−04 | 3.38E−04 | 1.49E−05 | 1.00E−06 | 2.11E−06 | 2.33E−05 | 1.57E−06 | 3.30E−06 | 3.35E−05 | 2.26E−06 | 4.75E−06 | 4.56E−05 | 3.07E−06 | 6.47E−06 |
| 74 | 1.11E−04 | 1.73E−04 | 2.49E−04 | 3.38E−04 | 1.49E−05 | 1.00E−06 | 2.00E−06 | 2.33E−05 | 1.57E−06 | 3.12E−06 | 3.35E−05 | 2.26E−06 | 4.49E−06 | 4.56E−05 | 3.07E−06 | 6.12E−06 |
| 75 | 1.11E−04 | 1.73E−04 | 2.49E−04 | 3.39E−04 | 1.49E−05 | 1.00E−06 | 1.89E−06 | 2.33E−05 | 1.57E−06 | 2.95E−06 | 3.35E−05 | 2.26E−06 | 4.25E−06 | 4.56E−05 | 3.07E−06 | 5.79E−06 |
| 76 | 1.11E−04 | 1.73E−04 | 2.49E−04 | 3.39E−04 | 1.49E−05 | 1.00E−06 | 1.79E−06 | 2.33E−05 | 1.57E−06 | 2.79E−06 | 3.35E−05 | 2.26E−06 | 4.02E−06 | 4.56E−05 | 3.07E−06 | 5.47E−06 |
| 77 | 1.11E−04 | 1.73E−04 | 2.49E−04 | 3.39E−04 | 1.49E−05 | 1.00E−06 | 1.69E−06 | 2.33E−05 | 1.57E−06 | 2.64E−06 | 3.35E−05 | 2.26E−06 | 3.80E−06 | 4.56E−05 | 3.07E−06 | 5.18E−06 |
| 78 | 1.11E−04 | 1.73E−04 | 2.50E−04 | 3.40E−04 | 1.49E−05 | 1.00E−06 | 1.60E−06 | 2.33E−05 | 1.57E−06 | 2.50E−06 | 3.35E−05 | 2.26E−06 | 3.60E−06 | 4.56E−05 | 3.07E−06 | 4.90E−06 |
| 79 | 1.11E−04 | 1.73E−04 | 2.50E−04 | 3.40E−04 | 1.49E−05 | 1.00E−06 | 1.51E−06 | 2.33E−05 | 1.57E−06 | 2.36E−06 | 3.35E−05 | 2.26E−06 | 3.40E−06 | 4.56E−05 | 3.07E−06 | 4.63E−06 |
| 80 | 1.11E−04 | 1.74E−04 | 2.50E−04 | 3.40E−04 | 1.49E−05 | 1.00E−06 | 1.43E−06 | 2.33E−05 | 1.57E−06 | 2.24E−06 | 3.35E−05 | 2.26E−06 | 3.22E−06 | 4.56E−05 | 3.07E−06 | 4.38E−06 |
| 81 | 1.11E−04 | 1.74E−04 | 2.50E−04 | 3.40E−04 | 1.49E−05 | 1.00E−06 | 1.35E−06 | 2.33E−05 | 1.57E−06 | 2.12E−06 | 3.35E−05 | 2.26E−06 | 3.05E−06 | 4.56E−05 | 3.07E−06 | 4.15E−06 |
| 82 | 1.11E−04 | 1.74E−04 | 2.50E−04 | 3.41E−04 | 1.49E−05 | 1.00E−06 | 1.28E−06 | 2.33E−05 | 1.57E−06 | 2.00E−06 | 3.35E−05 | 2.26E−06 | 2.88E−06 | 4.56E−05 | 3.07E−06 | 3.92E−06 |
| 83 | 1.11E−04 | 1.74E−04 | 2.50E−04 | 3.41E−04 | 1.49E−05 | 1.00E−06 | 1.21E−06 | 2.33E−05 | 1.57E−06 | 1.89E−06 | 3.35E−05 | 2.26E−06 | 2.73E−06 | 4.56E−05 | 3.07E−06 | 3.71E−06 |
| 84 | 1.11E−04 | 1.74E−04 | 2.51E−04 | 3.41E−04 | 1.49E−05 | 1.00E−06 | 1.15E−06 | 2.33E−05 | 1.57E−06 | 1.79E−06 | 3.35E−05 | 2.26E−06 | 2.58E−06 | 4.56E−05 | 3.07E−06 | 3.51E−06 |
| 85 | 1.11E−04 | 1.74E−04 | 2.51E−04 | 3.41E−04 | 1.49E−05 | 1.00E−06 | 1.08E−06 | 2.33E−05 | 1.57E−06 | 1.69E−06 | 3.35E−05 | 2.26E−06 | 2.44E−06 | 4.56E−05 | 3.07E−06 | 3.32E−06 |
| 86 | 1.11E−04 | 1.74E−04 | 2.51E−04 | 3.41E−04 | 1.49E−05 | 1.00E−06 | 1.03E−06 | 2.33E−05 | 1.57E−06 | 1.60E−06 | 3.35E−05 | 2.26E−06 | 2.31E−06 | 4.56E−05 | 3.07E−06 | 3.14E−06 |
| 87 | 1.12E−04 | 1.74E−04 | 2.51E−04 | 3.42E−04 | 1.49E−05 | 1.00E−06 | 9.70E−07 | 2.33E−05 | 1.57E−06 | 1.52E−06 | 3.35E−05 | 2.26E−06 | 2.18E−06 | 4.56E−05 | 3.07E−06 | 2.97E−06 |
| 88 | 1.12E−04 | 1.74E−04 | 2.51E−04 | 3.42E−04 | 1.49E−05 | 1.00E−06 | 9.18E−07 | 2.33E−05 | 1.57E−06 | 1.43E−06 | 3.35E−05 | 2.26E−06 | 2.07E−06 | 4.56E−05 | 3.07E−06 | 2.81E−06 |
| 89 | 1.12E−04 | 1.74E−04 | 2.51E−04 | 3.42E−04 | 1.49E−05 | 1.00E−06 | 8.68E−07 | 2.33E−05 | 1.57E−06 | 1.36E−06 | 3.35E−05 | 2.26E−06 | 1.95E−06 | 4.56E−05 | 3.07E−06 | 2.66E−06 |
| 90 | 1.12E−04 | 1.75E−04 | 2.51E−04 | 3.42E−04 | 1.49E−05 | 1.00E−06 | 8.21E−07 | 2.33E−05 | 1.57E−06 | 1.28E−06 | 3.35E−05 | 2.26E−06 | 1.85E−06 | 4.56E−05 | 3.07E−06 | 2.52E−06 |

**Table S16.** *Cont.*

| TIME (days) | cFA08:1 | cFA10:1 | cFA12:1 | cFA14:1 | cGA08:1 | cA08:1 | cF08:1 | cGA10:1 | cA10:1 | cF10:1 | cGA12:1 | cA12:1 | cF12:1 | cGA14:1 | cA14:1 | cF14:1 |
|---|---|---|---|---|---|---|---|---|---|---|---|---|---|---|---|---|
| 91 | 1.12E−04 | 1.75E−04 | 2.51E−04 | 3.42E−04 | 1.49E−05 | 1.00E−06 | 7.77E−07 | 2.33E−05 | 1.57E−06 | 1.21E−06 | 3.35E−05 | 2.26E−06 | 1.75E−06 | 4.56E−05 | 3.07E−06 | 2.38E−06 |
| 92 | 1.12E−04 | 1.75E−04 | 2.52E−04 | 3.42E−04 | 1.49E−05 | 1.00E−06 | 7.35E−07 | 2.33E−05 | 1.57E−06 | 1.15E−06 | 3.35E−05 | 2.26E−06 | 1.65E−06 | 4.56E−05 | 3.07E−06 | 2.25E−06 |
| 93 | 1.12E−04 | 1.75E−04 | 2.52E−04 | 3.42E−04 | 1.49E−05 | 1.00E−06 | 6.95E−07 | 2.33E−05 | 1.57E−06 | 1.09E−06 | 3.35E−05 | 2.26E−06 | 1.56E−06 | 4.56E−05 | 3.07E−06 | 2.13E−06 |
| 94 | 1.12E−04 | 1.75E−04 | 2.52E−04 | 3.43E−04 | 1.49E−05 | 1.00E−06 | 6.58E−07 | 2.33E−05 | 1.57E−06 | 1.03E−06 | 3.35E−05 | 2.26E−06 | 1.48E−06 | 4.56E−05 | 3.07E−06 | 2.01E−06 |
| 95 | 1.12E−04 | 1.75E−04 | 2.52E−04 | 3.43E−04 | 1.49E−05 | 1.00E−06 | 6.22E−07 | 2.33E−05 | 1.57E−06 | 9.72E−07 | 3.35E−05 | 2.26E−06 | 1.40E−06 | 4.56E−05 | 3.07E−06 | 1.91E−06 |
| 96 | 1.12E−04 | 1.75E−04 | 2.52E−04 | 3.43E−04 | 1.49E−05 | 1.00E−06 | 5.89E−07 | 2.33E−05 | 1.57E−06 | 9.20E−07 | 3.35E−05 | 2.26E−06 | 1.32E−06 | 4.56E−05 | 3.07E−06 | 1.80E−06 |
| 97 | 1.12E−04 | 1.75E−04 | 2.52E−04 | 3.43E−04 | 1.49E−05 | 1.00E−06 | 5.57E−07 | 2.33E−05 | 1.57E−06 | 8.70E−07 | 3.35E−05 | 2.26E−06 | 1.25E−06 | 4.56E−05 | 3.07E−06 | 1.71E−06 |
| 98 | 1.12E−04 | 1.75E−04 | 2.52E−04 | 3.43E−04 | 1.49E−05 | 1.00E−06 | 5.27E−07 | 2.33E−05 | 1.57E−06 | 8.23E−07 | 3.35E−05 | 2.26E−06 | 1.19E−06 | 4.56E−05 | 3.07E−06 | 1.61E−06 |
| 99 | 1.12E−04 | 1.75E−04 | 2.52E−04 | 3.43E−04 | 1.49E−05 | 1.00E−06 | 4.98E−07 | 2.33E−05 | 1.57E−06 | 7.79E−07 | 3.35E−05 | 2.26E−06 | 1.12E−06 | 4.56E−05 | 3.07E−06 | 1.53E−06 |
| 100 | 1.12E−04 | 1.75E−04 | 2.52E−04 | 3.43E−04 | 1.49E−05 | 1.00E−06 | 4.71E−07 | 2.33E−05 | 1.57E−06 | 7.37E−07 | 3.35E−05 | 2.26E−06 | 1.06E−06 | 4.56E−05 | 3.07E−06 | 1.44E−06 |
| 101 | 1.12E−04 | 1.75E−04 | 2.52E−04 | 3.43E−04 | 1.49E−05 | 1.00E−06 | 4.46E−07 | 2.33E−05 | 1.57E−06 | 6.97E−07 | 3.35E−05 | 2.26E−06 | 1.00E−06 | 4.56E−05 | 3.07E−06 | 1.37E−06 |
| 102 | 1.12E−04 | 1.75E−04 | 2.52E−04 | 3.43E−04 | 1.49E−05 | 1.00E−06 | 4.22E−07 | 2.33E−05 | 1.57E−06 | 6.59E−07 | 3.35E−05 | 2.26E−06 | 9.49E−07 | 4.56E−05 | 3.07E−06 | 1.29E−06 |
| 103 | 1.12E−04 | 1.75E−04 | 2.52E−04 | 3.43E−04 | 1.49E−05 | 1.00E−06 | 3.99E−07 | 2.33E−05 | 1.57E−06 | 6.24E−07 | 3.35E−05 | 2.26E−06 | 8.98E−07 | 4.56E−05 | 3.07E−06 | 1.22E−06 |
| 104 | 1.12E−04 | 1.75E−04 | 2.52E−04 | 3.43E−04 | 1.49E−05 | 1.00E−06 | 3.78E−07 | 2.33E−05 | 1.57E−06 | 5.90E−07 | 3.35E−05 | 2.26E−06 | 8.50E−07 | 4.56E−05 | 3.07E−06 | 1.16E−06 |
| 105 | 1.12E−04 | 1.75E−04 | 2.52E−04 | 3.43E−04 | 1.49E−05 | 1.00E−06 | 3.57E−07 | 2.33E−05 | 1.57E−06 | 5.58E−07 | 3.35E−05 | 2.26E−06 | 8.04E−07 | 4.56E−05 | 3.07E−06 | 1.09E−06 |
| 106 | 1.12E−04 | 1.75E−04 | 2.52E−04 | 3.44E−04 | 1.49E−05 | 1.00E−06 | 3.38E−07 | 2.33E−05 | 1.57E−06 | 5.28E−07 | 3.35E−05 | 2.26E−06 | 7.60E−07 | 4.56E−05 | 3.07E−06 | 1.03E−06 |
| 107 | 1.12E−04 | 1.75E−04 | 2.52E−04 | 3.44E−04 | 1.49E−05 | 1.00E−06 | 3.20E−07 | 2.33E−05 | 1.57E−06 | 4.99E−07 | 3.35E−05 | 2.26E−06 | 7.19E−07 | 4.56E−05 | 3.07E−06 | 9.79E−07 |
| 108 | 1.12E−04 | 1.75E−04 | 2.52E−04 | 3.44E−04 | 1.49E−05 | 1.00E−06 | 3.02E−07 | 2.33E−05 | 1.57E−06 | 4.72E−07 | 3.35E−05 | 2.26E−06 | 6.80E−07 | 4.56E−05 | 3.07E−06 | 9.26E−07 |
| 109 | 1.12E−04 | 1.75E−04 | 2.53E−04 | 3.44E−04 | 1.49E−05 | 1.00E−06 | 2.86E−07 | 2.33E−05 | 1.57E−06 | 4.47E−07 | 3.35E−05 | 2.26E−06 | 6.44E−07 | 4.56E−05 | 3.07E−06 | 8.76E−07 |
| 110 | 1.12E−04 | 1.75E−04 | 2.53E−04 | 3.44E−04 | 1.49E−05 | 1.00E−06 | 2.71E−07 | 2.33E−05 | 1.57E−06 | 4.23E−07 | 3.35E−05 | 2.26E−06 | 6.09E−07 | 4.56E−05 | 3.07E−06 | 8.29E−07 |
| 111 | 1.12E−04 | 1.75E−04 | 2.53E−04 | 3.44E−04 | 1.49E−05 | 1.00E−06 | 2.56E−07 | 2.33E−05 | 1.57E−06 | 4.00E−07 | 3.35E−05 | 2.26E−06 | 5.76E−07 | 4.56E−05 | 3.07E−06 | 7.84E−07 |
| 112 | 1.12E−04 | 1.75E−04 | 2.53E−04 | 3.44E−04 | 1.49E−05 | 1.00E−06 | 2.42E−07 | 2.33E−05 | 1.57E−06 | 3.78E−07 | 3.35E−05 | 2.26E−06 | 5.45E−07 | 4.56E−05 | 3.07E−06 | 7.42E−07 |
| 113 | 1.12E−04 | 1.75E−04 | 2.53E−04 | 3.44E−04 | 1.49E−05 | 1.00E−06 | 2.29E−07 | 2.33E−05 | 1.57E−06 | 3.58E−07 | 3.35E−05 | 2.26E−06 | 5.15E−07 | 4.56E−05 | 3.07E−06 | 7.02E−07 |

**Table S16.** *Cont.*

| TIME (days) | cFA08:1 | cFA10:1 | cFA12:1 | cFA14:1 | cGA08:1 | cA08:1 | cF08:1 | cGA10:1 | cA10:1 | cF10:1 | cGA12:1 | cA12:1 | cF12:1 | cGA14:1 | cA14:1 | cF14:1 |
|---|---|---|---|---|---|---|---|---|---|---|---|---|---|---|---|---|
| 114 | 1.12E−04 | 1.75E−04 | 2.53E−04 | 3.44E−04 | 1.49E−05 | 1.00E−06 | 2.17E−07 | 2.33E−05 | 1.57E−06 | 3.39E−07 | 3.35E−05 | 2.26E−06 | 4.88E−07 | 4.56E−05 | 3.07E−06 | 6.64E−07 |
| 115 | 1.12E−04 | 1.75E−04 | 2.53E−04 | 3.44E−04 | 1.49E−05 | 1.00E−06 | 2.05E−07 | 2.33E−05 | 1.57E−06 | 3.20E−07 | 3.35E−05 | 2.26E−06 | 4.61E−07 | 4.56E−05 | 3.07E−06 | 6.28E−07 |
| 116 | 1.12E−04 | 1.75E−04 | 2.53E−04 | 3.44E−04 | 1.49E−05 | 1.00E−06 | 1.94E−07 | 2.33E−05 | 1.57E−06 | 3.03E−07 | 3.35E−05 | 2.26E−06 | 4.36E−07 | 4.56E−05 | 3.07E−06 | 5.94E−07 |
| 117 | 1.12E−04 | 1.76E−04 | 2.53E−04 | 3.44E−04 | 1.49E−05 | 1.00E−06 | 1.83E−07 | 2.33E−05 | 1.57E−06 | 2.87E−07 | 3.35E−05 | 2.26E−06 | 4.13E−07 | 4.56E−05 | 3.07E−06 | 5.62E−07 |
| 118 | 1.12E−04 | 1.76E−04 | 2.53E−04 | 3.44E−04 | 1.49E−05 | 1.00E−06 | 1.74E−07 | 2.33E−05 | 1.57E−06 | 2.71E−07 | 3.35E−05 | 2.26E−06 | 3.91E−07 | 4.56E−05 | 3.07E−06 | 5.32E−07 |
| 119 | 1.12E−04 | 1.76E−04 | 2.53E−04 | 3.44E−04 | 1.49E−05 | 1.00E−06 | 1.64E−07 | 2.33E−05 | 1.57E−06 | 2.57E−07 | 3.35E−05 | 2.26E−06 | 3.69E−07 | 4.56E−05 | 3.07E−06 | 5.03E−07 |
| 120 | 1.12E−04 | 1.76E−04 | 2.53E−04 | 3.44E−04 | 1.49E−05 | 1.00E−06 | 1.55E−07 | 2.33E−05 | 1.57E−06 | 2.43E−07 | 3.35E−05 | 2.26E−06 | 3.49E−07 | 4.56E−05 | 3.07E−06 | 4.76E−07 |
| 121 | 1.12E−04 | 1.76E−04 | 2.53E−04 | 3.44E−04 | 1.49E−05 | 1.00E−06 | 1.47E−07 | 2.33E−05 | 1.57E−06 | 2.30E−07 | 3.35E−05 | 2.26E−06 | 3.31E−07 | 4.56E−05 | 3.07E−06 | 4.50E−07 |
| 122 | 1.12E−04 | 1.76E−04 | 2.53E−04 | 3.44E−04 | 1.49E−05 | 1.00E−06 | 1.39E−07 | 2.33E−05 | 1.57E−06 | 2.17E−07 | 3.35E−05 | 2.26E−06 | 3.13E−07 | 4.56E−05 | 3.07E−06 | 4.26E−07 |
| 123 | 1.12E−04 | 1.76E−04 | 2.53E−04 | 3.44E−04 | 1.49E−05 | 1.00E−06 | 1.31E−07 | 2.33E−05 | 1.57E−06 | 2.05E−07 | 3.35E−05 | 2.26E−06 | 2.96E−07 | 4.56E−05 | 3.07E−06 | 4.03E−07 |
| 124 | 1.12E−04 | 1.76E−04 | 2.53E−04 | 3.44E−04 | 1.49E−05 | 1.00E−06 | 1.24E−07 | 2.33E−05 | 1.57E−06 | 1.94E−07 | 3.35E−05 | 2.26E−06 | 2.80E−07 | 4.56E−05 | 3.07E−06 | 3.81E−07 |
| 125 | 1.12E−04 | 1.76E−04 | 2.53E−04 | 3.44E−04 | 1.49E−05 | 1.00E−06 | 1.18E−07 | 2.33E−05 | 1.57E−06 | 1.84E−07 | 3.35E−05 | 2.26E−06 | 2.65E−07 | 4.56E−05 | 3.07E−06 | 3.60E−07 |
| 126 | 1.12E−04 | 1.76E−04 | 2.53E−04 | 3.44E−04 | 1.49E−05 | 1.00E−06 | 1.11E−07 | 2.33E−05 | 1.57E−06 | 1.74E−07 | 3.35E−05 | 2.26E−06 | 2.50E−07 | 4.56E−05 | 3.07E−06 | 3.41E−07 |
| 127 | 1.12E−04 | 1.76E−04 | 2.53E−04 | 3.44E−04 | 1.49E−05 | 1.00E−06 | 1.05E−07 | 2.33E−05 | 1.57E−06 | 1.65E−07 | 3.35E−05 | 2.26E−06 | 2.37E−07 | 4.56E−05 | 3.07E−06 | 3.22E−07 |
| 128 | 1.12E−04 | 1.76E−04 | 2.53E−04 | 3.44E−04 | 1.49E−05 | 1.00E−06 | 9.96E−08 | 2.33E−05 | 1.57E−06 | 1.56E−07 | 3.35E−05 | 2.26E−06 | 2.24E−07 | 4.56E−05 | 3.07E−06 | 3.05E−07 |
| 129 | 1.12E−04 | 1.76E−04 | 2.53E−04 | 3.44E−04 | 1.49E−05 | 1.00E−06 | 9.42E−08 | 2.33E−05 | 1.57E−06 | 1.47E−07 | 3.35E−05 | 2.26E−06 | 2.12E−07 | 4.56E−05 | 3.07E−06 | 2.89E−07 |
| 130 | 1.12E−04 | 1.76E−04 | 2.53E−04 | 3.44E−04 | 1.49E−05 | 1.00E−06 | 8.91E−08 | 2.33E−05 | 1.57E−06 | 1.39E−07 | 3.35E−05 | 2.26E−06 | 2.01E−07 | 4.56E−05 | 3.07E−06 | 2.73E−07 |
| 131 | 1.12E−04 | 1.76E−04 | 2.53E−04 | 3.44E−04 | 1.49E−05 | 1.00E−06 | 8.43E−08 | 2.33E−05 | 1.57E−06 | 1.32E−07 | 3.35E−05 | 2.26E−06 | 1.90E−07 | 4.56E−05 | 3.07E−06 | 2.58E−07 |
| 132 | 1.12E−04 | 1.76E−04 | 2.53E−04 | 3.44E−04 | 1.49E−05 | 1.00E−06 | 7.98E−08 | 2.33E−05 | 1.57E−06 | 1.25E−07 | 3.35E−05 | 2.26E−06 | 1.79E−07 | 4.56E−05 | 3.07E−06 | 2.44E−07 |
| 133 | 1.12E−04 | 1.76E−04 | 2.53E−04 | 3.44E−04 | 1.49E−05 | 1.00E−06 | 7.55E−08 | 2.33E−05 | 1.57E−06 | 1.18E−07 | 3.35E−05 | 2.26E−06 | 1.70E−07 | 4.56E−05 | 3.07E−06 | 2.31E−07 |
| 134 | 1.12E−04 | 1.76E−04 | 2.53E−04 | 3.44E−04 | 1.49E−05 | 1.00E−06 | 7.14E−08 | 2.33E−05 | 1.57E−06 | 1.12E−07 | 3.35E−05 | 2.26E−06 | 1.61E−07 | 4.56E−05 | 3.07E−06 | 2.19E−07 |
| 135 | 1.12E−04 | 1.76E−04 | 2.53E−04 | 3.44E−04 | 1.49E−05 | 1.00E−06 | 6.75E−08 | 2.33E−05 | 1.57E−06 | 1.06E−07 | 3.35E−05 | 2.26E−06 | 1.52E−07 | 4.56E−05 | 3.07E−06 | 2.07E−07 |
| 136 | 1.12E−04 | 1.76E−04 | 2.53E−04 | 3.44E−04 | 1.49E−05 | 1.00E−06 | 6.39E−08 | 2.33E−05 | 1.57E−06 | 9.98E−08 | 3.35E−05 | 2.26E−06 | 1.44E−07 | 4.56E−05 | 3.07E−06 | 1.96E−07 |

**Table S16.** *Cont.*

| TIME (days) | cFA08:1 | cFA10:1 | cFA12:1 | cFA14:1 | cGA08:1 | cA08:1 | cF08:1 | cGA10:1 | cA10:1 | cF10:1 | cGA12:1 | cA12:1 | cF12:1 | cGA14:1 | cA14:1 | cF14:1 |
|---|---|---|---|---|---|---|---|---|---|---|---|---|---|---|---|---|
| 137 | 1.12E−04 | 1.76E−04 | 2.53E−04 | 3.44E−04 | 1.49E−05 | 1.00E−06 | 6.04E−08 | 2.33E−05 | 1.57E−06 | 9.44E−08 | 3.35E−05 | 2.26E−06 | 1.36E−07 | 4.56E−05 | 3.07E−06 | 1.85E−07 |
| 138 | 1.12E−04 | 1.76E−04 | 2.53E−04 | 3.44E−04 | 1.49E−05 | 1.00E−06 | 5.72E−08 | 2.33E−05 | 1.57E−06 | 8.93E−08 | 3.35E−05 | 2.26E−06 | 1.29E−07 | 4.56E−05 | 3.07E−06 | 1.75E−07 |
| 139 | 1.12E−04 | 1.76E−04 | 2.53E−04 | 3.44E−04 | 1.49E−05 | 1.00E−06 | 5.41E−08 | 2.33E−05 | 1.57E−06 | 8.45E−08 | 3.35E−05 | 2.26E−06 | 1.22E−07 | 4.56E−05 | 3.07E−06 | 1.66E−07 |
| 140 | 1.12E−04 | 1.76E−04 | 2.53E−04 | 3.44E−04 | 1.49E−05 | 1.00E−06 | 5.12E−08 | 2.33E−05 | 1.57E−06 | 7.99E−08 | 3.35E−05 | 2.26E−06 | 1.15E−07 | 4.56E−05 | 3.07E−06 | 1.57E−07 |
| 141 | 1.12E−04 | 1.76E−04 | 2.53E−04 | 3.44E−04 | 1.49E−05 | 1.00E−06 | 4.84E−08 | 2.33E−05 | 1.57E−06 | 7.56E−08 | 3.35E−05 | 2.26E−06 | 1.09E−07 | 4.56E−05 | 3.07E−06 | 1.48E−07 |
| 142 | 1.12E−04 | 1.76E−04 | 2.53E−04 | 3.44E−04 | 1.49E−05 | 1.00E−06 | 4.58E−08 | 2.33E−05 | 1.57E−06 | 7.15E−08 | 3.35E−05 | 2.26E−06 | 1.03E−07 | 4.56E−05 | 3.07E−06 | 1.40E−07 |
| 143 | 1.12E−04 | 1.76E−04 | 2.53E−04 | 3.44E−04 | 1.49E−05 | 1.00E−06 | 4.33E−08 | 2.33E−05 | 1.57E−06 | 6.77E−08 | 3.35E−05 | 2.26E−06 | 9.75E−08 | 4.56E−05 | 3.07E−06 | 1.33E−07 |
| 144 | 1.12E−04 | 1.76E−04 | 2.53E−04 | 3.44E−04 | 1.49E−05 | 1.00E−06 | 4.10E−08 | 2.33E−05 | 1.57E−06 | 6.40E−08 | 3.35E−05 | 2.26E−06 | 9.22E−08 | 4.56E−05 | 3.07E−06 | 1.25E−07 |
| 145 | 1.12E−04 | 1.76E−04 | 2.53E−04 | 3.44E−04 | 1.49E−05 | 1.00E−06 | 3.88E−08 | 2.33E−05 | 1.57E−06 | 6.06E−08 | 3.35E−05 | 2.26E−06 | 8.72E−08 | 4.56E−05 | 3.07E−06 | 1.19E−07 |
| 146 | 1.12E−04 | 1.76E−04 | 2.53E−04 | 3.44E−04 | 1.49E−05 | 1.00E−06 | 3.67E−08 | 2.33E−05 | 1.57E−06 | 5.73E−08 | 3.35E−05 | 2.26E−06 | 8.25E−08 | 4.56E−05 | 3.07E−06 | 1.12E−07 |
| 147 | 1.12E−04 | 1.76E−04 | 2.53E−04 | 3.44E−04 | 1.49E−05 | 1.00E−06 | 3.47E−08 | 2.33E−05 | 1.57E−06 | 5.42E−08 | 3.35E−05 | 2.26E−06 | 7.80E−08 | 4.56E−05 | 3.07E−06 | 1.06E−07 |
| 148 | 1.12E−04 | 1.76E−04 | 2.53E−04 | 3.44E−04 | 1.49E−05 | 1.00E−06 | 3.28E−08 | 2.33E−05 | 1.57E−06 | 5.13E−08 | 3.35E−05 | 2.26E−06 | 7.38E−08 | 4.56E−05 | 3.07E−06 | 1.00E−07 |
| 149 | 1.12E−04 | 1.76E−04 | 2.53E−04 | 3.44E−04 | 1.49E−05 | 1.00E−06 | 3.10E−08 | 2.33E−05 | 1.57E−06 | 4.85E−08 | 3.35E−05 | 2.26E−06 | 6.98E−08 | 4.56E−05 | 3.07E−06 | 9.51E−08 |
| 150 | 1.12E−04 | 1.76E−04 | 2.53E−04 | 3.44E−04 | 1.49E−05 | 1.00E−06 | 2.94E−08 | 2.33E−05 | 1.57E−06 | 4.59E−08 | 3.35E−05 | 2.26E−06 | 6.61E−08 | 4.56E−05 | 3.07E−06 | 8.99E−08 |
| 151 | 1.12E−04 | 1.76E−04 | 2.53E−04 | 3.44E−04 | 1.49E−05 | 1.00E−06 | 2.78E−08 | 2.33E−05 | 1.57E−06 | 4.34E−08 | 3.35E−05 | 2.26E−06 | 6.25E−08 | 4.56E−05 | 3.07E−06 | 8.51E−08 |
| 152 | 1.12E−04 | 1.76E−04 | 2.53E−04 | 3.44E−04 | 1.49E−05 | 1.00E−06 | 2.63E−08 | 2.33E−05 | 1.57E−06 | 4.11E−08 | 3.35E−05 | 2.26E−06 | 5.91E−08 | 4.56E−05 | 3.07E−06 | 8.05E−08 |
| 153 | 1.12E−04 | 1.76E−04 | 2.53E−04 | 3.44E−04 | 1.49E−05 | 1.00E−06 | 2.49E−08 | 2.33E−05 | 1.57E−06 | 3.88E−08 | 3.35E−05 | 2.26E−06 | 5.59E−08 | 4.56E−05 | 3.07E−06 | 7.61E−08 |
| 154 | 1.12E−04 | 1.76E−04 | 2.53E−04 | 3.45E−04 | 1.49E−05 | 1.00E−06 | 2.35E−08 | 2.33E−05 | 1.57E−06 | 3.67E−08 | 3.35E−05 | 2.26E−06 | 5.29E−08 | 4.56E−05 | 3.07E−06 | 7.20E−08 |
| 155 | 1.12E−04 | 1.76E−04 | 2.53E−04 | 3.45E−04 | 1.49E−05 | 1.00E−06 | 2.22E−08 | 2.33E−05 | 1.57E−06 | 3.48E−08 | 3.35E−05 | 2.26E−06 | 5.01E−08 | 4.56E−05 | 3.07E−06 | 6.81E−08 |
| 156 | 1.12E−04 | 1.76E−04 | 2.53E−04 | 3.45E−04 | 1.49E−05 | 1.00E−06 | 2.10E−08 | 2.33E−05 | 1.57E−06 | 3.29E−08 | 3.35E−05 | 2.26E−06 | 4.74E−08 | 4.56E−05 | 3.07E−06 | 6.45E−08 |
| 157 | 1.12E−04 | 1.76E−04 | 2.53E−04 | 3.45E−04 | 1.49E−05 | 1.00E−06 | 1.99E−08 | 2.33E−05 | 1.57E−06 | 3.11E−08 | 3.35E−05 | 2.26E−06 | 4.48E−08 | 4.56E−05 | 3.07E−06 | 6.10E−08 |
| 158 | 1.12E−04 | 1.76E−04 | 2.53E−04 | 3.45E−04 | 1.49E−05 | 1.00E−06 | 1.88E−08 | 2.33E−05 | 1.57E−06 | 2.94E−08 | 3.35E−05 | 2.26E−06 | 4.24E−08 | 4.56E−05 | 3.07E−06 | 5.77E−08 |
| 159 | 1.12E−04 | 1.76E−04 | 2.53E−04 | 3.45E−04 | 1.49E−05 | 1.00E−06 | 1.78E−08 | 2.33E−05 | 1.57E−06 | 2.78E−08 | 3.35E−05 | 2.26E−06 | 4.01E−08 | 4.56E−05 | 3.07E−06 | 5.46E−08 |

**Table S16.** *Cont.*

| TIME (days) | cFA08:1 | cFA10:1 | cFA12:1 | cFA14:1 | cGA08:1 | cA08:1 | cF08:1 | cGA10:1 | cA10:1 | cF10:1 | cGA12:1 | cA12:1 | cF12:1 | cGA14:1 | cA14:1 | cF14:1 |
|---|---|---|---|---|---|---|---|---|---|---|---|---|---|---|---|---|
| 160 | 1.12E−04 | 1.76E−04 | 2.53E−04 | 3.45E−04 | 1.49E−05 | 1.00E−06 | 1.69E−08 | 2.33E−05 | 1.57E−06 | 2.63E−08 | 3.35E−05 | 2.26E−06 | 3.79E−08 | 4.56E−05 | 3.07E−06 | 5.16E−08 |
| 161 | 1.12E−04 | 1.76E−04 | 2.53E−04 | 3.45E−04 | 1.49E−05 | 1.00E−06 | 1.59E−08 | 2.33E−05 | 1.57E−06 | 2.49E−08 | 3.35E−05 | 2.26E−06 | 3.59E−08 | 4.56E−05 | 3.07E−06 | 4.88E−08 |
| 162 | 1.12E−04 | 1.76E−04 | 2.53E−04 | 3.45E−04 | 1.49E−05 | 1.00E−06 | 1.51E−08 | 2.33E−05 | 1.57E−06 | 2.36E−08 | 3.35E−05 | 2.26E−06 | 3.39E−08 | 4.56E−05 | 3.07E−06 | 4.62E−08 |
| 163 | 1.12E−04 | 1.76E−04 | 2.53E−04 | 3.45E−04 | 1.49E−05 | 1.00E−06 | 1.43E−08 | 2.33E−05 | 1.57E−06 | 2.23E−08 | 3.35E−05 | 2.26E−06 | 3.21E−08 | 4.56E−05 | 3.07E−06 | 4.37E−08 |
| 164 | 1.13E−04 | 1.76E−04 | 2.53E−04 | 3.45E−04 | 1.49E−05 | 1.00E−06 | 1.35E−08 | 2.33E−05 | 1.57E−06 | 2.11E−08 | 3.35E−05 | 2.26E−06 | 3.04E−08 | 4.56E−05 | 3.07E−06 | 4.13E−08 |
| 165 | 1.13E−04 | 1.76E−04 | 2.53E−04 | 3.45E−04 | 1.49E−05 | 1.00E−06 | 1.28E−08 | 2.33E−05 | 1.57E−06 | 2.00E−08 | 3.35E−05 | 2.26E−06 | 2.87E−08 | 4.56E−05 | 3.07E−06 | 3.91E−08 |
| 166 | 1.13E−04 | 1.76E−04 | 2.53E−04 | 3.45E−04 | 1.49E−05 | 1.00E−06 | 1.21E−08 | 2.33E−05 | 1.57E−06 | 1.89E−08 | 3.35E−05 | 2.26E−06 | 2.72E−08 | 4.56E−05 | 3.07E−06 | 3.70E−08 |
| 167 | 1.13E−04 | 1.76E−04 | 2.53E−04 | 3.45E−04 | 1.49E−05 | 1.00E−06 | 1.14E−08 | 2.33E−05 | 1.57E−06 | 1.79E−08 | 3.35E−05 | 2.26E−06 | 2.57E−08 | 4.56E−05 | 3.07E−06 | 3.50E−08 |
| 168 | 1.13E−04 | 1.76E−04 | 2.53E−04 | 3.45E−04 | 1.49E−05 | 1.00E−06 | 1.08E−08 | 2.33E−05 | 1.57E−06 | 1.69E−08 | 3.35E−05 | 2.26E−06 | 2.43E−08 | 4.56E−05 | 3.07E−06 | 3.31E−08 |
| 169 | 1.13E−04 | 1.76E−04 | 2.53E−04 | 3.45E−04 | 1.49E−05 | 1.00E−06 | 1.02E−08 | 2.33E−05 | 1.57E−06 | 1.60E−08 | 3.35E−05 | 2.26E−06 | 2.30E−08 | 4.56E−05 | 3.07E−06 | 3.13E−08 |
| 170 | 1.13E−04 | 1.76E−04 | 2.53E−04 | 3.45E−04 | 1.49E−05 | 1.00E−06 | 9.67E−09 | 2.33E−05 | 1.57E−06 | 1.51E−08 | 3.35E−05 | 2.26E−06 | 2.18E−08 | 4.56E−05 | 3.07E−06 | 2.96E−08 |
| 171 | 1.13E−04 | 1.76E−04 | 2.53E−04 | 3.45E−04 | 1.49E−05 | 1.00E−06 | 9.15E−09 | 2.33E−05 | 1.57E−06 | 1.43E−08 | 3.35E−05 | 2.26E−06 | 2.06E−08 | 4.56E−05 | 3.07E−06 | 2.80E−08 |
| 172 | 1.13E−04 | 1.76E−04 | 2.53E−04 | 3.45E−04 | 1.49E−05 | 1.00E−06 | 8.66E−09 | 2.33E−05 | 1.57E−06 | 1.35E−08 | 3.35E−05 | 2.26E−06 | 1.95E−08 | 4.56E−05 | 3.07E−06 | 2.65E−08 |
| 173 | 1.13E−04 | 1.76E−04 | 2.53E−04 | 3.45E−04 | 1.49E−05 | 1.00E−06 | 8.19E−09 | 2.33E−05 | 1.57E−06 | 1.28E−08 | 3.35E−05 | 2.26E−06 | 1.84E−08 | 4.56E−05 | 3.07E−06 | 2.51E−08 |
| 174 | 1.13E−04 | 1.76E−04 | 2.53E−04 | 3.45E−04 | 1.49E−05 | 1.00E−06 | 7.75E−09 | 2.33E−05 | 1.57E−06 | 1.21E−08 | 3.35E−05 | 2.26E−06 | 1.74E−08 | 4.56E−05 | 3.07E−06 | 2.37E−08 |
| 175 | 1.13E−04 | 1.76E−04 | 2.53E−04 | 3.45E−04 | 1.49E−05 | 1.00E−06 | 7.33E−09 | 2.33E−05 | 1.57E−06 | 1.15E−08 | 3.35E−05 | 2.26E−06 | 1.65E−08 | 4.56E−05 | 3.07E−06 | 2.24E−08 |
| 176 | 1.13E−04 | 1.76E−04 | 2.53E−04 | 3.45E−04 | 1.49E−05 | 1.00E−06 | 6.93E−09 | 2.33E−05 | 1.57E−06 | 1.08E−08 | 3.35E−05 | 2.26E−06 | 1.56E−08 | 4.56E−05 | 3.07E−06 | 2.12E−08 |
| 177 | 1.13E−04 | 1.76E−04 | 2.53E−04 | 3.45E−04 | 1.49E−05 | 1.00E−06 | 6.56E−09 | 2.33E−05 | 1.57E−06 | 1.02E−08 | 3.35E−05 | 2.26E−06 | 1.48E−08 | 4.56E−05 | 3.07E−06 | 2.01E−08 |
| 178 | 1.13E−04 | 1.76E−04 | 2.53E−04 | 3.45E−04 | 1.49E−05 | 1.00E−06 | 6.20E−09 | 2.33E−05 | 1.57E−06 | 9.69E−09 | 3.35E−05 | 2.26E−06 | 1.40E−08 | 4.56E−05 | 3.07E−06 | 1.90E−08 |
| 179 | 1.13E−04 | 1.76E−04 | 2.53E−04 | 3.45E−04 | 1.49E−05 | 1.00E−06 | 5.87E−09 | 2.33E−05 | 1.57E−06 | 9.17E−09 | 3.35E−05 | 2.26E−06 | 1.32E−08 | 4.56E−05 | 3.07E−06 | 1.80E−08 |
| 180 | 1.13E−04 | 1.76E−04 | 2.53E−04 | 3.45E−04 | 1.49E−05 | 1.00E−06 | 5.55E−09 | 2.33E−05 | 1.57E−06 | 8.68E−09 | 3.35E−05 | 2.26E−06 | 1.25E−08 | 4.56E−05 | 3.07E−06 | 1.70E−08 |
| 181 | 1.13E−04 | 1.76E−04 | 2.53E−04 | 3.45E−04 | 1.49E−05 | 1.00E−06 | 5.25E−09 | 2.33E−05 | 1.57E−06 | 8.21E−09 | 3.35E−05 | 2.26E−06 | 1.18E−08 | 4.56E−05 | 3.07E−06 | 1.61E−08 |
| 182 | 1.13E−04 | 1.76E−04 | 2.53E−04 | 3.45E−04 | 1.49E−05 | 1.00E−06 | 4.97E−09 | 2.33E−05 | 1.57E−06 | 7.76E−09 | 3.35E−05 | 2.26E−06 | 1.12E−08 | 4.56E−05 | 3.07E−06 | 1.52E−08 |

**Table S16.** *Cont.*

| TIME (days) | cFA08:1 | cFA10:1 | cFA12:1 | cFA14:1 | cGA08:1 | cA08:1 | cF08:1 | cGA10:1 | cA10:1 | cF10:1 | cGA12:1 | cA12:1 | cF12:1 | cGA14:1 | cA14:1 | cF14:1 |
|---|---|---|---|---|---|---|---|---|---|---|---|---|---|---|---|---|
| 183 | 1.13E−04 | 1.76E−04 | 2.53E−04 | 3.45E−04 | 1.49E−05 | 1.00E−06 | 4.70E−09 | 2.33E−05 | 1.57E−06 | 7.34E−09 | 3.35E−05 | 2.26E−06 | 1.06E−08 | 4.56E−05 | 3.07E−06 | 1.44E−08 |
| 184 | 1.13E−04 | 1.76E−04 | 2.53E−04 | 3.45E−04 | 1.49E−05 | 1.00E−06 | 4.45E−09 | 2.33E−05 | 1.57E−06 | 6.95E−09 | 3.35E−05 | 2.26E−06 | 1.00E−08 | 4.56E−05 | 3.07E−06 | 1.36E−08 |
| 185 | 1.13E−04 | 1.76E−04 | 2.53E−04 | 3.45E−04 | 1.49E−05 | 1.00E−06 | 4.21E−09 | 2.33E−05 | 1.57E−06 | 6.57E−09 | 3.35E−05 | 2.26E−06 | 9.46E−09 | 4.56E−05 | 3.07E−06 | 1.29E−08 |
| 186 | 1.13E−04 | 1.76E−04 | 2.53E−04 | 3.45E−04 | 1.49E−05 | 1.00E−06 | 3.98E−09 | 2.33E−05 | 1.57E−06 | 6.22E−09 | 3.35E−05 | 2.26E−06 | 8.95E−09 | 4.56E−05 | 3.07E−06 | 1.22E−08 |
| 187 | 1.13E−04 | 1.76E−04 | 2.53E−04 | 3.45E−04 | 1.49E−05 | 1.00E−06 | 3.76E−09 | 2.33E−05 | 1.57E−06 | 5.88E−09 | 3.35E−05 | 2.26E−06 | 8.47E−09 | 4.56E−05 | 3.07E−06 | 1.15E−08 |
| 188 | 1.13E−04 | 1.76E−04 | 2.53E−04 | 3.45E−04 | 1.49E−05 | 1.00E−06 | 3.56E−09 | 2.33E−05 | 1.57E−06 | 5.56E−09 | 3.35E−05 | 2.26E−06 | 8.01E−09 | 4.56E−05 | 3.07E−06 | 1.09E−08 |
| 189 | 1.13E−04 | 1.76E−04 | 2.53E−04 | 3.45E−04 | 1.49E−05 | 1.00E−06 | 3.37E−09 | 2.33E−05 | 1.57E−06 | 5.26E−09 | 3.35E−05 | 2.26E−06 | 7.58E−09 | 4.56E−05 | 3.07E−06 | 1.03E−08 |
| 190 | 1.13E−04 | 1.76E−04 | 2.53E−04 | 3.45E−04 | 1.49E−05 | 1.00E−06 | 3.19E−09 | 2.33E−05 | 1.57E−06 | 4.98E−09 | 3.35E−05 | 2.26E−06 | 7.17E−09 | 4.56E−05 | 3.07E−06 | 9.76E−09 |
| 191 | 1.13E−04 | 1.76E−04 | 2.53E−04 | 3.45E−04 | 1.49E−05 | 1.00E−06 | 3.01E−09 | 2.33E−05 | 1.57E−06 | 4.71E−09 | 3.35E−05 | 2.26E−06 | 6.78E−09 | 4.56E−05 | 3.07E−06 | 9.23E−09 |
| 192 | 1.13E−04 | 1.76E−04 | 2.53E−04 | 3.45E−04 | 1.49E−05 | 1.00E−06 | 2.85E−09 | 2.33E−05 | 1.57E−06 | 4.46E−09 | 3.35E−05 | 2.26E−06 | 6.42E−09 | 4.56E−05 | 3.07E−06 | 8.73E−09 |
| 193 | 1.13E−04 | 1.76E−04 | 2.53E−04 | 3.45E−04 | 1.49E−05 | 1.00E−06 | 2.70E−09 | 2.33E−05 | 1.57E−06 | 4.22E−09 | 3.35E−05 | 2.26E−06 | 6.07E−09 | 4.56E−05 | 3.07E−06 | 8.26E−09 |
| 194 | 1.13E−04 | 1.76E−04 | 2.53E−04 | 3.45E−04 | 1.49E−05 | 1.00E−06 | 2.55E−09 | 2.33E−05 | 1.57E−06 | 3.99E−09 | 3.35E−05 | 2.26E−06 | 5.74E−09 | 4.56E−05 | 3.07E−06 | 7.82E−09 |
| 195 | 1.13E−04 | 1.76E−04 | 2.53E−04 | 3.45E−04 | 1.49E−05 | 1.00E−06 | 2.41E−09 | 2.33E−05 | 1.57E−06 | 3.77E−09 | 3.35E−05 | 2.26E−06 | 5.43E−09 | 4.56E−05 | 3.07E−06 | 7.39E−09 |
| 196 | 1.13E−04 | 1.76E−04 | 2.53E−04 | 3.45E−04 | 1.49E−05 | 1.00E−06 | 2.28E−09 | 2.33E−05 | 1.57E−06 | 3.57E−09 | 3.35E−05 | 2.26E−06 | 5.14E−09 | 4.56E−05 | 3.07E−06 | 6.99E−09 |
| 197 | 1.13E−04 | 1.76E−04 | 2.53E−04 | 3.45E−04 | 1.49E−05 | 1.00E−06 | 2.16E−09 | 2.33E−05 | 1.57E−06 | 3.38E−09 | 3.35E−05 | 2.26E−06 | 4.86E−09 | 4.56E−05 | 3.07E−06 | 6.62E−09 |
| 198 | 1.13E−04 | 1.76E−04 | 2.53E−04 | 3.45E−04 | 1.49E−05 | 1.00E−06 | 2.04E−09 | 2.33E−05 | 1.57E−06 | 3.19E−09 | 3.35E−05 | 2.26E−06 | 4.60E−09 | 4.56E−05 | 3.07E−06 | 6.26E−09 |
| 199 | 1.13E−04 | 1.76E−04 | 2.53E−04 | 3.45E−04 | 1.49E−05 | 1.00E−06 | 1.93E−09 | 2.33E−05 | 1.57E−06 | 3.02E−09 | 3.35E−05 | 2.26E−06 | 4.35E−09 | 4.56E−05 | 3.07E−06 | 5.92E−09 |
| 200 | 1.13E−04 | 1.76E−04 | 2.53E−04 | 3.45E−04 | 1.49E−05 | 1.00E−06 | 1.83E−09 | 2.33E−05 | 1.57E−06 | 2.86E−09 | 3.35E−05 | 2.26E−06 | 4.12E−09 | 4.56E−05 | 3.07E−06 | 5.60E−09 |

**Table S17.** Modelled data for saxagliptin degradation products (values in mol/$g_{saxa}$).

| TIME (days) | cSAXA08:1 | cSCA08:1 | cESCA08:1 | cSFA08:1 | cSAXA10:1 | cSCA10:1 | cESCA10:1 | cSFA10:1 | cSAXA12:1 | cSCA12:1 | cESCA12:1 | cSFA12:1 | cSAXA14:1 | cSCA14:1 | cESCA14:1 | cSFA14:1 |
|---|---|---|---|---|---|---|---|---|---|---|---|---|---|---|---|---|
| 0 | 0.00317 | 0 | 0 | 0 | 0.00317 | 0 | 0 | 0 | 0.00317 | 0 | 0 | 0 | 0.00317 | 0 | 0 | 0 |
| 1 | 0.00317 | 5.26E−07 | 6.37E−09 | 1.20E−09 | 0.00317 | 5.26E−07 | 6.37E−09 | 1.87E−09 | 0.00317 | 5.26E−07 | 6.36E−09 | 2.70E−09 | 0.00317 | 5.25E−07 | 6.35E−09 | 3.67E−09 |
| 2 | 0.003169 | 1.04E−06 | 2.53E−08 | 7.99E−09 | 0.003169 | 1.04E−06 | 2.52E−08 | 1.25E−08 | 0.003169 | 1.04E−06 | 2.51E−08 | 1.81E−08 | 0.003169 | 1.03E−06 | 2.50E−08 | 2.47E−08 |
| 3 | 0.003169 | 1.54E−06 | 5.64E−08 | 2.29E−08 | 0.003169 | 1.54E−06 | 5.61E−08 | 3.59E−08 | 0.003169 | 1.53E−06 | 5.57E−08 | 5.20E−08 | 0.003169 | 1.52E−06 | 5.52E−08 | 7.13E−08 |
| 4 | 0.003168 | 2.03E−06 | 9.95E−08 | 4.65E−08 | 0.003168 | 2.02E−06 | 9.86E−08 | 7.32E−08 | 0.003168 | 2.01E−06 | 9.75E−08 | 1.06E−07 | 0.003168 | 2.00E−06 | 9.62E−08 | 1.46E−07 |
| 5 | 0.003168 | 2.51E−06 | 1.54E−07 | 7.89E−08 | 0.003168 | 2.49E−06 | 1.52E−07 | 1.25E−07 | 0.003168 | 2.47E−06 | 1.50E−07 | 1.82E−07 | 0.003168 | 2.45E−06 | 1.47E−07 | 2.51E−07 |
| 6 | 0.003167 | 2.97E−06 | 2.20E−07 | 1.20E−07 | 0.003167 | 2.95E−06 | 2.17E−07 | 1.89E−07 | 0.003167 | 2.92E−06 | 2.13E−07 | 2.77E−07 | 0.003167 | 2.89E−06 | 2.08E−07 | 3.84E−07 |
| 7 | 0.003167 | 3.43E−06 | 2.98E−07 | 1.68E−07 | 0.003167 | 3.40E−06 | 2.92E−07 | 2.67E−07 | 0.003166 | 3.36E−06 | 2.85E−07 | 3.91E−07 | 0.003166 | 3.32E−06 | 2.77E−07 | 5.44E−07 |
| 8 | 0.003166 | 3.87E−06 | 3.86E−07 | 2.24E−07 | 0.003166 | 3.83E−06 | 3.77E−07 | 3.56E−07 | 0.003166 | 3.78E−06 | 3.67E−07 | 5.23E−07 | 0.003166 | 3.72E−06 | 3.55E−07 | 7.31E−07 |
| 9 | 0.003165 | 4.30E−06 | 4.84E−07 | 2.86E−07 | 0.003165 | 4.25E−06 | 4.72E−07 | 4.56E−07 | 0.003165 | 4.19E−06 | 4.57E−07 | 6.72E−07 | 0.003165 | 4.12E−06 | 4.41E−07 | 9.41E−07 |
| 10 | 0.003165 | 4.73E−06 | 5.93E−07 | 3.54E−07 | 0.003165 | 4.66E−06 | 5.76E−07 | 5.65E−07 | 0.003164 | 4.59E−06 | 5.56E−07 | 8.36E−07 | 0.003164 | 4.50E−06 | 5.34E−07 | 1.18E−06 |
| 11 | 0.003164 | 5.14E−06 | 7.12E−07 | 4.28E−07 | 0.003164 | 5.07E−06 | 6.90E−07 | 6.84E−07 | 0.003164 | 4.98E−06 | 6.64E−07 | 1.02E−06 | 0.003164 | 4.87E−06 | 6.34E−07 | 1.43E−06 |
| 12 | 0.003164 | 5.54E−06 | 8.41E−07 | 5.06E−07 | 0.003163 | 5.46E−06 | 8.12E−07 | 8.12E−07 | 0.003163 | 5.35E−06 | 7.79E−07 | 1.21E−06 | 0.003163 | 5.23E−06 | 7.41E−07 | 1.71E−06 |
| 13 | 0.003163 | 5.94E−06 | 9.80E−07 | 5.89E−07 | 0.003163 | 5.84E−06 | 9.43E−07 | 9.46E−07 | 0.003162 | 5.72E−06 | 9.01E−07 | 1.41E−06 | 0.003162 | 5.58E−06 | 8.54E−07 | 2.00E−06 |
| 14 | 0.003162 | 6.32E−06 | 1.13E−06 | 6.76E−07 | 0.003162 | 6.21E−06 | 1.08E−06 | 1.09E−06 | 0.003162 | 6.07E−06 | 1.03E−06 | 1.63E−06 | 0.003161 | 5.92E−06 | 9.74E−07 | 2.31E−06 |
| 15 | 0.003162 | 6.69E−06 | 1.28E−06 | 7.66E−07 | 0.003161 | 6.57E−06 | 1.23E−06 | 1.24E−06 | 0.003161 | 6.42E−06 | 1.17E−06 | 1.85E−06 | 0.00316 | 6.25E−06 | 1.10E−06 | 2.64E−06 |
| 16 | 0.003161 | 7.06E−06 | 1.45E−06 | 8.59E−07 | 0.003161 | 6.92E−06 | 1.39E−06 | 1.39E−06 | 0.00316 | 6.76E−06 | 1.31E−06 | 2.08E−06 | 0.00316 | 6.57E−06 | 1.23E−06 | 2.98E−06 |
| 17 | 0.00316 | 7.41E−06 | 1.62E−06 | 9.56E−07 | 0.00316 | 7.26E−06 | 1.55E−06 | 1.55E−06 | 0.00316 | 7.08E−06 | 1.46E−06 | 2.33E−06 | 0.003159 | 6.88E−06 | 1.37E−06 | 3.33E−06 |
| 18 | 0.00316 | 7.76E−06 | 1.81E−06 | 1.05E−06 | 0.003159 | 7.60E−06 | 1.72E−06 | 1.71E−06 | 0.003159 | 7.40E−06 | 1.62E−06 | 2.57E−06 | 0.003158 | 7.18E−06 | 1.51E−06 | 3.70E−06 |
| 19 | 0.003159 | 8.10E−06 | 2.00E−06 | 1.15E−06 | 0.003159 | 7.92E−06 | 1.90E−06 | 1.88E−06 | 0.003158 | 7.71E−06 | 1.78E−06 | 2.83E−06 | 0.003157 | 7.47E−06 | 1.65E−06 | 4.07E−06 |
| 20 | 0.003159 | 8.43E−06 | 2.20E−06 | 1.26E−06 | 0.003158 | 8.24E−06 | 2.08E−06 | 2.04E−06 | 0.003157 | 8.02E−06 | 1.95E−06 | 3.09E−06 | 0.003156 | 7.76E−06 | 1.81E−06 | 4.46E−06 |
| 21 | 0.003158 | 8.76E−06 | 2.41E−06 | 1.36E−06 | 0.003157 | 8.55E−06 | 2.27E−06 | 2.22E−06 | 0.003157 | 8.31E−06 | 2.12E−06 | 3.36E−06 | 0.003155 | 8.04E−06 | 1.96E−06 | 4.85E−06 |

**Table S17.** *Cont.*

| TIME (days) | cSAXA08:1 | cSCA08:1 | cESCA08:1 | cSFA08:1 | cSAXA10:1 | cSCA10:1 | cESCA10:1 | cSFA10:1 | cSAXA12:1 | cSCA12:1 | cESCA12:1 | cSFA12:1 | cSAXA14:1 | cSCA14:1 | cESCA14:1 | cSFA14:1 |
|---|---|---|---|---|---|---|---|---|---|---|---|---|---|---|---|---|
| 22 | 0.003157 | 9.07E−06 | 2.62E−06 | 1.47E−06 | 0.003157 | 8.86E−06 | 2.47E−06 | 2.39E−06 | 0.003156 | 8.60E−06 | 2.31E−06 | 3.63E−06 | 0.003155 | 8.31E−06 | 2.12E−06 | 5.26E−06 |
| 23 | 0.003157 | 9.38E−06 | 2.84E−06 | 1.57E−06 | 0.003156 | 9.15E−06 | 2.68E−06 | 2.57E−06 | 0.003155 | 8.88E−06 | 2.49E−06 | 3.90E−06 | 0.003154 | 8.57E−06 | 2.29E−06 | 5.66E−06 |
| 24 | 0.003156 | 9.68E−06 | 3.07E−06 | 1.68E−06 | 0.003155 | 9.44E−06 | 2.89E−06 | 2.75E−06 | 0.003154 | 9.15E−06 | 2.68E−06 | 4.18E−06 | 0.003153 | 8.83E−06 | 2.46E−06 | 6.08E−06 |
| 25 | 0.003155 | 9.97E−06 | 3.31E−06 | 1.79E−06 | 0.003155 | 9.72E−06 | 3.11E−06 | 2.93E−06 | 0.003154 | 9.42E−06 | 2.88E−06 | 4.46E−06 | 0.003152 | 9.08E−06 | 2.63E−06 | 6.50E−06 |
| 26 | 0.003155 | 1.03E−05 | 3.55E−06 | 1.89E−06 | 0.003154 | 1.00E−05 | 3.33E−06 | 3.11E−06 | 0.003153 | 9.68E−06 | 3.08E−06 | 4.74E−06 | 0.003151 | 9.33E−06 | 2.81E−06 | 6.92E−06 |
| 27 | 0.003154 | 1.05E−05 | 3.81E−06 | 2.00E−06 | 0.003153 | 1.03E−05 | 3.56E−06 | 3.29E−06 | 0.003152 | 9.94E−06 | 3.28E−06 | 5.02E−06 | 0.00315 | 9.57E−06 | 2.99E−06 | 7.34E−06 |
| 28 | 0.003153 | 1.08E−05 | 4.06E−06 | 2.11E−06 | 0.003153 | 1.05E−05 | 3.80E−06 | 3.47E−06 | 0.003151 | 1.02E−05 | 3.50E−06 | 5.31E−06 | 0.003149 | 9.80E−06 | 3.17E−06 | 7.77E−06 |
| 29 | 0.003153 | 1.11E−05 | 4.33E−06 | 2.22E−06 | 0.003152 | 1.08E−05 | 4.04E−06 | 3.65E−06 | 0.003151 | 1.04E−05 | 3.71E−06 | 5.59E−06 | 0.003148 | 1.00E−05 | 3.36E−06 | 8.20E−06 |
| 30 | 0.003152 | 1.13E−05 | 4.60E−06 | 2.33E−06 | 0.003151 | 1.10E−05 | 4.28E−06 | 3.83E−06 | 0.00315 | 1.07E−05 | 3.93E−06 | 5.88E−06 | 0.003148 | 1.03E−05 | 3.55E−06 | 8.63E−06 |
| 31 | 0.003152 | 1.16E−05 | 4.87E−06 | 2.43E−06 | 0.003151 | 1.13E−05 | 4.53E−06 | 4.01E−06 | 0.003149 | 1.09E−05 | 4.15E−06 | 6.16E−06 | 0.003147 | 1.05E−05 | 3.75E−06 | 9.06E−06 |
| 32 | 0.003151 | 1.18E−05 | 5.16E−06 | 2.54E−06 | 0.00315 | 1.15E−05 | 4.79E−06 | 4.19E−06 | 0.003148 | 1.11E−05 | 4.38E−06 | 6.45E−06 | 0.003146 | 1.07E−05 | 3.95E−06 | 9.49E−06 |
| 33 | 0.00315 | 1.21E−05 | 5.44E−06 | 2.65E−06 | 0.003149 | 1.17E−05 | 5.05E−06 | 4.37E−06 | 0.003148 | 1.13E−05 | 4.62E−06 | 6.73E−06 | 0.003145 | 1.09E−05 | 4.15E−06 | 9.92E−06 |
| 34 | 0.00315 | 1.23E−05 | 5.74E−06 | 2.75E−06 | 0.003149 | 1.20E−05 | 5.32E−06 | 4.55E−06 | 0.003147 | 1.16E−05 | 4.85E−06 | 7.01E−06 | 0.003144 | 1.11E−05 | 4.36E−06 | 1.03E−05 |
| 35 | 0.003149 | 1.25E−05 | 6.04E−06 | 2.86E−06 | 0.003148 | 1.22E−05 | 5.59E−06 | 4.72E−06 | 0.003146 | 1.18E−05 | 5.09E−06 | 7.29E−06 | 0.003143 | 1.13E−05 | 4.57E−06 | 1.08E−05 |
| 36 | 0.003148 | 1.28E−05 | 6.34E−06 | 2.96E−06 | 0.003147 | 1.24E−05 | 5.87E−06 | 4.90E−06 | 0.003145 | 1.20E−05 | 5.34E−06 | 7.57E−06 | 0.003142 | 1.15E−05 | 4.78E−06 | 1.12E−05 |
| 37 | 0.003148 | 1.30E−05 | 6.65E−06 | 3.06E−06 | 0.003147 | 1.26E−05 | 6.15E−06 | 5.07E−06 | 0.003144 | 1.22E−05 | 5.59E−06 | 7.85E−06 | 0.003141 | 1.17E−05 | 4.99E−06 | 1.16E−05 |
| 38 | 0.003147 | 1.32E−05 | 6.97E−06 | 3.17E−06 | 0.003146 | 1.28E−05 | 6.44E−06 | 5.25E−06 | 0.003144 | 1.24E−05 | 5.84E−06 | 8.12E−06 | 0.00314 | 1.19E−05 | 5.21E−06 | 1.20E−05 |
| 39 | 0.003147 | 1.34E−05 | 7.29E−06 | 3.27E−06 | 0.003145 | 1.30E−05 | 6.73E−06 | 5.42E−06 | 0.003143 | 1.26E−05 | 6.10E−06 | 8.39E−06 | 0.00314 | 1.21E−05 | 5.43E−06 | 1.25E−05 |
| 40 | 0.003146 | 1.36E−05 | 7.62E−06 | 3.37E−06 | 0.003145 | 1.32E−05 | 7.02E−06 | 5.59E−06 | 0.003142 | 1.28E−05 | 6.36E−06 | 8.66E−06 | 0.003139 | 1.22E−05 | 5.66E−06 | 1.29E−05 |
| 41 | 0.003145 | 1.38E−05 | 7.95E−06 | 3.46E−06 | 0.003144 | 1.34E−05 | 7.32E−06 | 5.75E−06 | 0.003141 | 1.29E−05 | 6.62E−06 | 8.93E−06 | 0.003138 | 1.24E−05 | 5.88E−06 | 1.33E−05 |
| 42 | 0.003145 | 1.40E−05 | 8.28E−06 | 3.56E−06 | 0.003143 | 1.36E−05 | 7.62E−06 | 5.92E−06 | 0.003141 | 1.31E−05 | 6.89E−06 | 9.19E−06 | 0.003137 | 1.26E−05 | 6.11E−06 | 1.37E−05 |
| 43 | 0.003144 | 1.42E−05 | 8.62E−06 | 3.66E−06 | 0.003143 | 1.38E−05 | 7.93E−06 | 6.08E−06 | 0.00314 | 1.33E−05 | 7.16E−06 | 9.45E−06 | 0.003136 | 1.27E−05 | 6.35E−06 | 1.41E−05 |

**Table S17.** *Cont.*

| TIME (days) | cSAXA08:1 | cSCA08:1 | cESCA08:1 | cSFA08:1 | cSAXA10:1 | cSCA10:1 | cESCA10:1 | cSFA10:1 | cSAXA12:1 | cSCA12:1 | cESCA12:1 | cSFA12:1 | cSAXA14:1 | cSCA14:1 | cESCA14:1 | cSFA14:1 |
|---|---|---|---|---|---|---|---|---|---|---|---|---|---|---|---|---|
| 44 | 0.003143 | 1.44E−05 | 8.97E−06 | 3.75E−06 | 0.003142 | 1.40E−05 | 8.24E−06 | 6.24E−06 | 0.003139 | 1.35E−05 | 7.43E−06 | 9.71E−06 | 0.003135 | 1.29E−05 | 6.58E−06 | 1.45E−05 |
| 45 | 0.003143 | 1.45E−05 | 9.31E−06 | 3.84E−06 | 0.003141 | 1.41E−05 | 8.55E−06 | 6.40E−06 | 0.003139 | 1.36E−05 | 7.71E−06 | 9.96E−06 | 0.003134 | 1.31E−05 | 6.82E−06 | 1.49E−05 |
| 46 | 0.003142 | 1.47E−05 | 9.67E−06 | 3.94E−06 | 0.003141 | 1.43E−05 | 8.87E−06 | 6.56E−06 | 0.003138 | 1.38E−05 | 7.99E−06 | 1.02E−05 | 0.003133 | 1.32E−05 | 7.06E−06 | 1.53E−05 |
| 47 | 0.003142 | 1.49E−05 | 1.00E−05 | 4.03E−06 | 0.00314 | 1.45E−05 | 9.19E−06 | 6.71E−06 | 0.003137 | 1.40E−05 | 8.27E−06 | 1.05E−05 | 0.003132 | 1.34E−05 | 7.30E−06 | 1.56E−05 |
| 48 | 0.003141 | 1.51E−05 | 1.04E−05 | 4.12E−06 | 0.003139 | 1.46E−05 | 9.52E−06 | 6.86E−06 | 0.003136 | 1.41E−05 | 8.56E−06 | 1.07E−05 | 0.003132 | 1.35E−05 | 7.55E−06 | 1.60E−05 |
| 49 | 0.00314 | 1.52E−05 | 1.08E−05 | 4.20E−06 | 0.003139 | 1.48E−05 | 9.85E−06 | 7.01E−06 | 0.003136 | 1.43E−05 | 8.84E−06 | 1.09E−05 | 0.003131 | 1.37E−05 | 7.79E−06 | 1.64E−05 |
| 50 | 0.00314 | 1.54E−05 | 1.11E−05 | 4.29E−06 | 0.003138 | 1.50E−05 | 1.02E−05 | 7.16E−06 | 0.003135 | 1.44E−05 | 9.14E−06 | 1.12E−05 | 0.00313 | 1.38E−05 | 8.04E−06 | 1.67E−05 |
| 51 | 0.003139 | 1.55E−05 | 1.15E−05 | 4.37E−06 | 0.003137 | 1.51E−05 | 1.05E−05 | 7.31E−06 | 0.003134 | 1.46E−05 | 9.43E−06 | 1.14E−05 | 0.003129 | 1.40E−05 | 8.30E−06 | 1.71E−05 |
| 52 | 0.003138 | 1.57E−05 | 1.19E−05 | 4.46E−06 | 0.003137 | 1.53E−05 | 1.09E−05 | 7.45E−06 | 0.003133 | 1.47E−05 | 9.73E−06 | 1.16E−05 | 0.003128 | 1.41E−05 | 8.55E−06 | 1.75E−05 |
| 53 | 0.003138 | 1.58E−05 | 1.23E−05 | 4.54E−06 | 0.003136 | 1.54E−05 | 1.12E−05 | 7.59E−06 | 0.003133 | 1.49E−05 | 1.00E−05 | 1.19E−05 | 0.003127 | 1.43E−05 | 8.81E−06 | 1.78E−05 |
| 54 | 0.003137 | 1.60E−05 | 1.26E−05 | 4.62E−06 | 0.003135 | 1.55E−05 | 1.15E−05 | 7.73E−06 | 0.003132 | 1.50E−05 | 1.03E−05 | 1.21E−05 | 0.003126 | 1.44E−05 | 9.07E−06 | 1.82E−05 |
| 55 | 0.003137 | 1.61E−05 | 1.30E−05 | 4.70E−06 | 0.003135 | 1.57E−05 | 1.19E−05 | 7.86E−06 | 0.003131 | 1.52E−05 | 1.06E−05 | 1.23E−05 | 0.003126 | 1.45E−05 | 9.33E−06 | 1.85E−05 |
| 56 | 0.003136 | 1.63E−05 | 1.34E−05 | 4.78E−06 | 0.003134 | 1.58E−05 | 1.22E−05 | 7.99E−06 | 0.003131 | 1.53E−05 | 1.09E−05 | 1.25E−05 | 0.003125 | 1.47E−05 | 9.59E−06 | 1.88E−05 |
| 57 | 0.003135 | 1.64E−05 | 1.38E−05 | 4.85E−06 | 0.003133 | 1.60E−05 | 1.26E−05 | 8.12E−06 | 0.00313 | 1.54E−05 | 1.13E−05 | 1.27E−05 | 0.003124 | 1.48E−05 | 9.86E−06 | 1.92E−05 |
| 58 | 0.003135 | 1.65E−05 | 1.42E−05 | 4.93E−06 | 0.003133 | 1.61E−05 | 1.29E−05 | 8.25E−06 | 0.003129 | 1.55E−05 | 1.16E−05 | 1.29E−05 | 0.003123 | 1.49E−05 | 1.01E−05 | 1.95E−05 |
| 59 | 0.003134 | 1.66E−05 | 1.46E−05 | 5.00E−06 | 0.003132 | 1.62E−05 | 1.33E−05 | 8.38E−06 | 0.003128 | 1.57E−05 | 1.19E−05 | 1.31E−05 | 0.003122 | 1.50E−05 | 1.04E−05 | 1.98E−05 |
| 60 | 0.003134 | 1.68E−05 | 1.50E−05 | 5.07E−06 | 0.003132 | 1.63E−05 | 1.37E−05 | 8.50E−06 | 0.003128 | 1.58E−05 | 1.22E−05 | 1.33E−05 | 0.003121 | 1.51E−05 | 1.07E−05 | 2.01E−05 |
| 61 | 0.003133 | 1.69E−05 | 1.54E−05 | 5.14E−06 | 0.003131 | 1.65E−05 | 1.40E−05 | 8.62E−06 | 0.003127 | 1.59E−05 | 1.25E−05 | 1.35E−05 | 0.003121 | 1.53E−05 | 1.09E−05 | 2.04E−05 |
| 62 | 0.003132 | 1.70E−05 | 1.58E−05 | 5.21E−06 | 0.00313 | 1.66E−05 | 1.44E−05 | 8.74E−06 | 0.003126 | 1.60E−05 | 1.28E−05 | 1.37E−05 | 0.00312 | 1.54E−05 | 1.12E−05 | 2.07E−05 |
| 63 | 0.003132 | 1.71E−05 | 1.62E−05 | 5.28E−06 | 0.00313 | 1.67E−05 | 1.48E−05 | 8.86E−06 | 0.003126 | 1.61E−05 | 1.32E−05 | 1.39E−05 | 0.003119 | 1.55E−05 | 1.15E−05 | 2.10E−05 |
| 64 | 0.003131 | 1.72E−05 | 1.66E−05 | 5.34E−06 | 0.003129 | 1.68E−05 | 1.51E−05 | 8.97E−06 | 0.003125 | 1.63E−05 | 1.35E−05 | 1.41E−05 | 0.003118 | 1.56E−05 | 1.18E−05 | 2.13E−05 |
| 65 | 0.003131 | 1.73E−05 | 1.71E−05 | 5.41E−06 | 0.003128 | 1.69E−05 | 1.55E−05 | 9.08E−06 | 0.003124 | 1.64E−05 | 1.38E−05 | 1.43E−05 | 0.003117 | 1.57E−05 | 1.21E−05 | 2.16E−05 |

**Table S17.** *Cont.*

| TIME (days) | cSAXA08:1 | cSCA08:1 | cESCA08:1 | cSFA08:1 | cSAXA10:1 | cSCA10:1 | cESCA10:1 | cSFA1 0:1 | cSAXA12:1 | cSCA12:1 | cESCA12:1 | cSFA12:1 | cSAXA14:1 | cSCA14:1 | cESCA14:1 | cSFA14:1 |
|---|---|---|---|---|---|---|---|---|---|---|---|---|---|---|---|---|
| 66 | 0.00313 | 1.74E−05 | 1.75E−05 | 5.47E−06 | 0.003128 | 1.70E−05 | 1.59E−05 | 9.19E−06 | 0.003124 | 1.65E−05 | 1.42E−05 | 1.45E−05 | 0.003117 | 1.58E−05 | 1.23E−05 | 2.18E−05 |
| 67 | 0.003129 | 1.75E−05 | 1.79E−05 | 5.54E−06 | 0.003127 | 1.71E−05 | 1.63E−05 | 9.30E−06 | 0.003123 | 1.66E−05 | 1.45E−05 | 1.46E−05 | 0.003116 | 1.59E−05 | 1.26E−05 | 2.21E−05 |
| 68 | 0.003129 | 1.76E−05 | 1.83E−05 | 5.60E−06 | 0.003127 | 1.72E−05 | 1.67E−05 | 9.41E−06 | 0.003122 | 1.67E−05 | 1.48E−05 | 1.48E−05 | 0.003115 | 1.60E−05 | 1.29E−05 | 2.24E−05 |
| 69 | 0.003128 | 1.77E−05 | 1.88E−05 | 5.66E−06 | 0.003126 | 1.73E−05 | 1.70E−05 | 9.51E−06 | 0.003122 | 1.68E−05 | 1.52E−05 | 1.50E−05 | 0.003114 | 1.61E−05 | 1.32E−05 | 2.26E−05 |
| 70 | 0.003128 | 1.78E−05 | 1.92E−05 | 5.71E−06 | 0.003125 | 1.74E−05 | 1.74E−05 | 9.61E−06 | 0.003121 | 1.69E−05 | 1.55E−05 | 1.51E−05 | 0.003113 | 1.62E−05 | 1.35E−05 | 2.29E−05 |
| 71 | 0.003127 | 1.79E−05 | 1.96E−05 | 5.77E−06 | 0.003125 | 1.75E−05 | 1.78E−05 | 9.71E−06 | 0.00312 | 1.70E−05 | 1.58E−05 | 1.53E−05 | 0.003113 | 1.63E−05 | 1.38E−05 | 2.31E−05 |
| 72 | 0.003127 | 1.80E−05 | 2.00E−05 | 5.83E−06 | 0.003124 | 1.76E−05 | 1.82E−05 | 9.80E−06 | 0.00312 | 1.71E−05 | 1.62E−05 | 1.54E−05 | 0.003112 | 1.64E−05 | 1.41E−05 | 2.34E−05 |
| 73 | 0.003126 | 1.81E−05 | 2.05E−05 | 5.88E−06 | 0.003124 | 1.77E−05 | 1.86E−05 | 9.90E−06 | 0.003119 | 1.72E−05 | 1.65E−05 | 1.56E−05 | 0.003111 | 1.65E−05 | 1.44E−05 | 2.36E−05 |
| 74 | 0.003125 | 1.82E−05 | 2.09E−05 | 5.94E−06 | 0.003123 | 1.78E−05 | 1.90E−05 | 9.99E−06 | 0.003118 | 1.73E−05 | 1.69E−05 | 1.58E−05 | 0.00311 | 1.66E−05 | 1.47E−05 | 2.39E−05 |
| 75 | 0.003125 | 1.83E−05 | 2.14E−05 | 5.99E−06 | 0.003122 | 1.79E−05 | 1.94E−05 | 1.01E−05 | 0.003118 | 1.73E−05 | 1.72E−05 | 1.59E−05 | 0.003109 | 1.67E−05 | 1.50E−05 | 2.41E−05 |
| 76 | 0.003124 | 1.84E−05 | 2.18E−05 | 6.04E−06 | 0.003122 | 1.80E−05 | 1.98E−05 | 1.02E−05 | 0.003117 | 1.74E−05 | 1.76E−05 | 1.60E−05 | 0.003109 | 1.68E−05 | 1.53E−05 | 2.43E−05 |
| 77 | 0.003124 | 1.85E−05 | 2.22E−05 | 6.09E−06 | 0.003121 | 1.80E−05 | 2.02E−05 | 1.03E−05 | 0.003116 | 1.75E−05 | 1.79E−05 | 1.62E−05 | 0.003108 | 1.69E−05 | 1.56E−05 | 2.45E−05 |
| 78 | 0.003123 | 1.85E−05 | 2.27E−05 | 6.14E−06 | 0.003121 | 1.81E−05 | 2.06E−05 | 1.03E−05 | 0.003116 | 1.76E−05 | 1.83E−05 | 1.63E−05 | 0.003107 | 1.69E−05 | 1.59E−05 | 2.47E−05 |
| 79 | 0.003123 | 1.86E−05 | 2.31E−05 | 6.19E−06 | 0.00312 | 1.82E−05 | 2.10E−05 | 1.04E−05 | 0.003115 | 1.77E−05 | 1.86E−05 | 1.65E−05 | 0.003106 | 1.70E−05 | 1.62E−05 | 2.50E−05 |
| 80 | 0.003122 | 1.87E−05 | 2.36E−05 | 6.23E−06 | 0.00312 | 1.83E−05 | 2.14E−05 | 1.05E−05 | 0.003115 | 1.78E−05 | 1.90E−05 | 1.66E−05 | 0.003106 | 1.71E−05 | 1.65E−05 | 2.52E−05 |
| 81 | 0.003121 | 1.88E−05 | 2.40E−05 | 6.28E−06 | 0.003119 | 1.84E−05 | 2.18E−05 | 1.06E−05 | 0.003114 | 1.78E−05 | 1.93E−05 | 1.67E−05 | 0.003105 | 1.72E−05 | 1.68E−05 | 2.54E−05 |
| 82 | 0.003121 | 1.88E−05 | 2.45E−05 | 6.32E−06 | 0.003118 | 1.84E−05 | 2.22E−05 | 1.07E−05 | 0.003113 | 1.79E−05 | 1.97E−05 | 1.68E−05 | 0.003104 | 1.73E−05 | 1.71E−05 | 2.56E−05 |
| 83 | 0.00312 | 1.89E−05 | 2.50E−05 | 6.37E−06 | 0.003118 | 1.85E−05 | 2.26E−05 | 1.07E−05 | 0.003113 | 1.80E−05 | 2.01E−05 | 1.70E−05 | 0.003103 | 1.73E−05 | 1.74E−05 | 2.57E−05 |
| 84 | 0.00312 | 1.90E−05 | 2.54E−05 | 6.41E−06 | 0.003117 | 1.86E−05 | 2.30E−05 | 1.08E−05 | 0.003112 | 1.81E−05 | 2.04E−05 | 1.71E−05 | 0.003103 | 1.74E−05 | 1.77E−05 | 2.59E−05 |
| 85 | 0.003119 | 1.90E−05 | 2.59E−05 | 6.45E−06 | 0.003117 | 1.86E−05 | 2.35E−05 | 1.09E−05 | 0.003111 | 1.81E−05 | 2.08E−05 | 1.72E−05 | 0.003102 | 1.75E−05 | 1.80E−05 | 2.61E−05 |
| 86 | 0.003119 | 1.91E−05 | 2.63E−05 | 6.49E−06 | 0.003116 | 1.87E−05 | 2.39E−05 | 1.09E−05 | 0.003111 | 1.82E−05 | 2.12E−05 | 1.73E−05 | 0.003101 | 1.76E−05 | 1.83E−05 | 2.63E−05 |
| 87 | 0.003118 | 1.92E−05 | 2.68E−05 | 6.53E−06 | 0.003115 | 1.88E−05 | 2.43E−05 | 1.10E−05 | 0.00311 | 1.83E−05 | 2.15E−05 | 1.74E−05 | 0.0031 | 1.76E−05 | 1.86E−05 | 2.65E−05 |

**Table S17.** *Cont.*

| TIME (days) | cSAXA08:1 | cSCA08:1 | cESCA08:1 | cSFA08:1 | cSAXA10:1 | cSCA10:1 | cESCA10:1 | cSFA10:1 | cSAXA12:1 | cSCA12:1 | cESCA12:1 | cSFA12:1 | cSAXA14:1 | cSCA14:1 | cESCA14:1 | cSFA14:1 |
|---|---|---|---|---|---|---|---|---|---|---|---|---|---|---|---|---|
| 88 | 0.003117 | 1.92E−05 | 2.73E−05 | 6.57E−06 | 0.003115 | 1.88E−05 | 2.47E−05 | 1.11E−05 | 0.00311 | 1.83E−05 | 2.19E−05 | 1.75E−05 | 0.0031 | 1.77E−05 | 1.90E−05 | 2.66E−05 |
| 89 | 0.003117 | 1.93E−05 | 2.77E−05 | 6.61E−06 | 0.003114 | 1.89E−05 | 2.51E−05 | 1.12E−05 | 0.003109 | 1.84E−05 | 2.23E−05 | 1.76E−05 | 0.003099 | 1.78E−05 | 1.93E−05 | 2.68E−05 |
| 90 | 0.003116 | 1.93E−05 | 2.82E−05 | 6.64E−06 | 0.003114 | 1.90E−05 | 2.55E−05 | 1.12E−05 | 0.003108 | 1.85E−05 | 2.26E−05 | 1.77E−05 | 0.003098 | 1.78E−05 | 1.96E−05 | 2.70E−05 |
| 91 | 0.003116 | 1.94E−05 | 2.86E−05 | 6.68E−06 | 0.003113 | 1.90E−05 | 2.60E−05 | 1.13E−05 | 0.003108 | 1.85E−05 | 2.30E−05 | 1.78E−05 | 0.003097 | 1.79E−05 | 1.99E−05 | 2.71E−05 |
| 92 | 0.003115 | 1.94E−05 | 2.91E−05 | 6.71E−06 | 0.003113 | 1.91E−05 | 2.64E−05 | 1.13E−05 | 0.003107 | 1.86E−05 | 2.34E−05 | 1.79E−05 | 0.003097 | 1.80E−05 | 2.02E−05 | 2.73E−05 |
| 93 | 0.003115 | 1.95E−05 | 2.96E−05 | 6.75E−06 | 0.003112 | 1.91E−05 | 2.68E−05 | 1.14E−05 | 0.003106 | 1.87E−05 | 2.37E−05 | 1.80E−05 | 0.003096 | 1.80E−05 | 2.05E−05 | 2.74E−05 |
| 94 | 0.003114 | 1.96E−05 | 3.01E−05 | 6.78E−06 | 0.003111 | 1.92E−05 | 2.72E−05 | 1.15E−05 | 0.003106 | 1.87E−05 | 2.41E−05 | 1.81E−05 | 0.003095 | 1.81E−05 | 2.09E−05 | 2.76E−05 |
| 95 | 0.003114 | 1.96E−05 | 3.05E−05 | 6.82E−06 | 0.003111 | 1.92E−05 | 2.77E−05 | 1.15E−05 | 0.003105 | 1.88E−05 | 2.45E−05 | 1.82E−05 | 0.003095 | 1.82E−05 | 2.12E−05 | 2.77E−05 |
| 96 | 0.003113 | 1.97E−05 | 3.10E−05 | 6.85E−06 | 0.00311 | 1.93E−05 | 2.81E−05 | 1.16E−05 | 0.003105 | 1.88E−05 | 2.49E−05 | 1.83E−05 | 0.003094 | 1.82E−05 | 2.15E−05 | 2.79E−05 |
| 97 | 0.003112 | 1.97E−05 | 3.15E−05 | 6.88E−06 | 0.00311 | 1.94E−05 | 2.85E−05 | 1.16E−05 | 0.003104 | 1.89E−05 | 2.53E−05 | 1.84E−05 | 0.003093 | 1.83E−05 | 2.18E−05 | 2.80E−05 |
| 98 | 0.003112 | 1.97E−05 | 3.20E−05 | 6.91E−06 | 0.003109 | 1.94E−05 | 2.90E−05 | 1.17E−05 | 0.003103 | 1.89E−05 | 2.56E−05 | 1.85E−05 | 0.003092 | 1.83E−05 | 2.22E−05 | 2.81E−05 |
| 99 | 0.003111 | 1.98E−05 | 3.24E−05 | 6.94E−06 | 0.003109 | 1.95E−05 | 2.94E−05 | 1.17E−05 | 0.003103 | 1.90E−05 | 2.60E−05 | 1.86E−05 | 0.003092 | 1.84E−05 | 2.25E−05 | 2.83E−05 |
| 100 | 0.003111 | 1.98E−05 | 3.29E−05 | 6.97E−06 | 0.003108 | 1.95E−05 | 2.98E−05 | 1.18E−05 | 0.003102 | 1.90E−05 | 2.64E−05 | 1.87E−05 | 0.003091 | 1.84E−05 | 2.28E−05 | 2.84E−05 |
| 101 | 0.00311 | 1.99E−05 | 3.34E−05 | 6.99E−06 | 0.003108 | 1.95E−05 | 3.02E−05 | 1.18E−05 | 0.003102 | 1.91E−05 | 2.68E−05 | 1.87E−05 | 0.00309 | 1.85E−05 | 2.31E−05 | 2.85E−05 |
| 102 | 0.00311 | 1.99E−05 | 3.39E−05 | 7.02E−06 | 0.003107 | 1.96E−05 | 3.07E−05 | 1.19E−05 | 0.003101 | 1.91E−05 | 2.72E−05 | 1.88E−05 | 0.00309 | 1.86E−05 | 2.35E−05 | 2.86E−05 |
| 103 | 0.003109 | 2.00E−05 | 3.43E−05 | 7.05E−06 | 0.003106 | 1.96E−05 | 3.11E−05 | 1.19E−05 | 0.0031 | 1.92E−05 | 2.75E−05 | 1.89E−05 | 0.003089 | 1.86E−05 | 2.38E−05 | 2.88E−05 |
| 104 | 0.003109 | 2.00E−05 | 3.48E−05 | 7.08E−06 | 0.003106 | 1.97E−05 | 3.16E−05 | 1.20E−05 | 0.0031 | 1.92E−05 | 2.79E−05 | 1.90E−05 | 0.003088 | 1.87E−05 | 2.41E−05 | 2.89E−05 |
| 105 | 0.003108 | 2.00E−05 | 3.53E−05 | 7.10E−06 | 0.003105 | 1.97E−05 | 3.20E−05 | 1.20E−05 | 0.003099 | 1.93E−05 | 2.83E−05 | 1.90E−05 | 0.003088 | 1.87E−05 | 2.45E−05 | 2.90E−05 |
| 106 | 0.003107 | 2.01E−05 | 3.58E−05 | 7.13E−06 | 0.003105 | 1.98E−05 | 3.24E−05 | 1.20E−05 | 0.003099 | 1.93E−05 | 2.87E−05 | 1.91E−05 | 0.003087 | 1.88E−05 | 2.48E−05 | 2.91E−05 |
| 107 | 0.003107 | 2.01E−05 | 3.63E−05 | 7.15E−06 | 0.003104 | 1.98E−05 | 3.29E−05 | 1.21E−05 | 0.003098 | 1.94E−05 | 2.91E−05 | 1.92E−05 | 0.003086 | 1.88E−05 | 2.51E−05 | 2.92E−05 |
| 108 | 0.003106 | 2.02E−05 | 3.68E−05 | 7.17E−06 | 0.003104 | 1.98E−05 | 3.33E−05 | 1.21E−05 | 0.003098 | 1.94E−05 | 2.95E−05 | 1.92E−05 | 0.003086 | 1.89E−05 | 2.55E−05 | 2.93E−05 |
| 109 | 0.003106 | 2.02E−05 | 3.73E−05 | 7.20E−06 | 0.003103 | 1.99E−05 | 3.38E−05 | 1.22E−05 | 0.003097 | 1.95E−05 | 2.99E−05 | 1.93E−05 | 0.003085 | 1.89E−05 | 2.58E−05 | 2.94E−05 |

**Table S17.** *Cont.*

| TIME (days) | cSAXA08:1 | cSCA08:1 | cESCA08:1 | cSFA08:1 | cSAXA10:1 | cSCA10:1 | cESCA10:1 | cSFA1 0:1 | cSAXA12:1 | cSCA12:1 | cESCA12:1 | cSFA12:1 | cSAXA14:1 | cSCA14:1 | cESCA14:1 | cSFA14:1 |
|---|---|---|---|---|---|---|---|---|---|---|---|---|---|---|---|---|
| 110 | 0.003105 | 2.02E−05 | 3.77E−05 | 7.22E−06 | 0.003103 | 1.99E−05 | 3.42E−05 | 1.22E−05 | 0.003096 | 1.95E−05 | 3.03E−05 | 1.94E−05 | 0.003084 | 1.89E−05 | 2.61E−05 | 2.95E−05 |
| 111 | 0.003105 | 2.03E−05 | 3.82E−05 | 7.24E−06 | 0.003102 | 2.00E−05 | 3.46E−05 | 1.22E−05 | 0.003096 | 1.95E−05 | 3.07E−05 | 1.94E−05 | 0.003084 | 1.90E−05 | 2.65E−05 | 2.96E−05 |
| 112 | 0.003104 | 2.03E−05 | 3.87E−05 | 7.26E−06 | 0.003102 | 2.00E−05 | 3.51E−05 | 1.23E−05 | 0.003095 | 1.96E−05 | 3.10E−05 | 1.95E−05 | 0.003083 | 1.90E−05 | 2.68E−05 | 2.97E−05 |
| 113 | 0.003104 | 2.03E−05 | 3.92E−05 | 7.28E−06 | 0.003101 | 2.00E−05 | 3.55E−05 | 1.23E−05 | 0.003095 | 1.96E−05 | 3.14E−05 | 1.95E−05 | 0.003082 | 1.91E−05 | 2.72E−05 | 2.98E−05 |
| 114 | 0.003103 | 2.04E−05 | 3.97E−05 | 7.30E−06 | 0.003101 | 2.01E−05 | 3.60E−05 | 1.24E−05 | 0.003094 | 1.97E−05 | 3.18E−05 | 1.96E−05 | 0.003082 | 1.91E−05 | 2.75E−05 | 2.99E−05 |
| 115 | 0.003103 | 2.04E−05 | 4.02E−05 | 7.32E−06 | 0.0031 | 2.01E−05 | 3.64E−05 | 1.24E−05 | 0.003094 | 1.97E−05 | 3.22E−05 | 1.97E−05 | 0.003081 | 1.92E−05 | 2.78E−05 | 3.00E−05 |
| 116 | 0.003102 | 2.04E−05 | 4.07E−05 | 7.34E−06 | 0.003099 | 2.01E−05 | 3.69E−05 | 1.24E−05 | 0.003093 | 1.97E−05 | 3.26E−05 | 1.97E−05 | 0.00308 | 1.92E−05 | 2.82E−05 | 3.00E−05 |
| 117 | 0.003102 | 2.04E−05 | 4.12E−05 | 7.36E−06 | 0.003099 | 2.02E−05 | 3.73E−05 | 1.25E−05 | 0.003092 | 1.98E−05 | 3.30E−05 | 1.98E−05 | 0.00308 | 1.92E−05 | 2.85E−05 | 3.01E−05 |
| 118 | 0.003101 | 2.05E−05 | 4.17E−05 | 7.38E−06 | 0.003098 | 2.02E−05 | 3.78E−05 | 1.25E−05 | 0.003092 | 1.98E−05 | 3.34E−05 | 1.98E−05 | 0.003079 | 1.93E−05 | 2.89E−05 | 3.02E−05 |
| 119 | 0.0031 | 2.05E−05 | 4.22E−05 | 7.40E−06 | 0.003098 | 2.02E−05 | 3.82E−05 | 1.25E−05 | 0.003091 | 1.98E−05 | 3.38E−05 | 1.99E−05 | 0.003078 | 1.93E−05 | 2.92E−05 | 3.03E−05 |
| 120 | 0.0031 | 2.05E−05 | 4.27E−05 | 7.41E−06 | 0.003097 | 2.03E−05 | 3.87E−05 | 1.25E−05 | 0.003091 | 1.99E−05 | 3.42E−05 | 1.99E−05 | 0.003078 | 1.94E−05 | 2.96E−05 | 3.04E−05 |
| 121 | 0.003099 | 2.05E−05 | 4.32E−05 | 7.43E−06 | 0.003097 | 2.03E−05 | 3.91E−05 | 1.26E−05 | 0.00309 | 1.99E−05 | 3.46E−05 | 2.00E−05 | 0.003077 | 1.94E−05 | 2.99E−05 | 3.04E−05 |
| 122 | 0.003099 | 2.06E−05 | 4.36E−05 | 7.45E−06 | 0.003096 | 2.03E−05 | 3.96E−05 | 1.26E−05 | 0.00309 | 1.99E−05 | 3.50E−05 | 2.00E−05 | 0.003076 | 1.94E−05 | 3.02E−05 | 3.05E−05 |
| 123 | 0.003098 | 2.06E−05 | 4.41E−05 | 7.46E−06 | 0.003096 | 2.03E−05 | 4.00E−05 | 1.26E−05 | 0.003089 | 2.00E−05 | 3.54E−05 | 2.00E−05 | 0.003076 | 1.95E−05 | 3.06E−05 | 3.06E−05 |
| 124 | 0.003098 | 2.06E−05 | 4.46E−05 | 7.48E−06 | 0.003095 | 2.04E−05 | 4.05E−05 | 1.27E−05 | 0.003088 | 2.00E−05 | 3.58E−05 | 2.01E−05 | 0.003075 | 1.95E−05 | 3.09E−05 | 3.06E−05 |
| 125 | 0.003097 | 2.06E−05 | 4.51E−05 | 7.49E−06 | 0.003095 | 2.04E−05 | 4.09E−05 | 1.27E−05 | 0.003088 | 2.00E−05 | 3.62E−05 | 2.01E−05 | 0.003074 | 1.95E−05 | 3.13E−05 | 3.07E−05 |
| 126 | 0.003097 | 2.07E−05 | 4.56E−05 | 7.51E−06 | 0.003094 | 2.04E−05 | 4.14E−05 | 1.27E−05 | 0.003087 | 2.01E−05 | 3.66E−05 | 2.02E−05 | 0.003074 | 1.96E−05 | 3.16E−05 | 3.08E−05 |
| 127 | 0.003096 | 2.07E−05 | 4.61E−05 | 7.52E−06 | 0.003094 | 2.04E−05 | 4.18E−05 | 1.27E−05 | 0.003087 | 2.01E−05 | 3.70E−05 | 2.02E−05 | 0.003073 | 1.96E−05 | 3.20E−05 | 3.08E−05 |
| 128 | 0.003096 | 2.07E−05 | 4.66E−05 | 7.53E−06 | 0.003093 | 2.05E−05 | 4.23E−05 | 1.28E−05 | 0.003086 | 2.01E−05 | 3.74E−05 | 2.03E−05 | 0.003072 | 1.96E−05 | 3.23E−05 | 3.09E−05 |
| 129 | 0.003095 | 2.07E−05 | 4.71E−05 | 7.55E−06 | 0.003093 | 2.05E−05 | 4.27E−05 | 1.28E−05 | 0.003086 | 2.01E−05 | 3.78E−05 | 2.03E−05 | 0.003072 | 1.97E−05 | 3.27E−05 | 3.10E−05 |
| 130 | 0.003095 | 2.07E−05 | 4.76E−05 | 7.56E−06 | 0.003092 | 2.05E−05 | 4.32E−05 | 1.28E−05 | 0.003085 | 2.02E−05 | 3.82E−05 | 2.03E−05 | 0.003071 | 1.97E−05 | 3.30E−05 | 3.10E−05 |
| 131 | 0.003094 | 2.08E−05 | 4.81E−05 | 7.57E−06 | 0.003091 | 2.05E−05 | 4.36E−05 | 1.28E−05 | 0.003085 | 2.02E−05 | 3.86E−05 | 2.04E−05 | 0.00307 | 1.97E−05 | 3.34E−05 | 3.11E−05 |

**Table S17.** *Cont.*

| TIME (days) | cSAXA08:1 | cSCA08:1 | cESCA08:1 | cSFA08:1 | cSAXA10:1 | cSCA10:1 | cESCA10:1 | cSFA10:1 | cSAXA12:1 | cSCA12:1 | cESCA12:1 | cSFA12:1 | cSAXA14:1 | cSCA14:1 | cESCA14:1 | cSFA14:1 |
|---|---|---|---|---|---|---|---|---|---|---|---|---|---|---|---|---|
| 132 | 0.003093 | 2.08E−05 | 4.86E−05 | 7.59E−06 | 0.003091 | 2.06E−05 | 4.41E−05 | 1.28E−05 | 0.003084 | 2.02E−05 | 3.90E−05 | 2.04E−05 | 0.00307 | 1.97E−05 | 3.37E−05 | 3.11E−05 |
| 133 | 0.003093 | 2.08E−05 | 4.91E−05 | 7.60E−06 | 0.00309 | 2.06E−05 | 4.45E−05 | 1.29E−05 | 0.003083 | 2.02E−05 | 3.94E−05 | 2.04E−05 | 0.003069 | 1.98E−05 | 3.41E−05 | 3.12E−05 |
| 134 | 0.003092 | 2.08E−05 | 4.96E−05 | 7.61E−06 | 0.00309 | 2.06E−05 | 4.50E−05 | 1.29E−05 | 0.003083 | 2.03E−05 | 3.99E−05 | 2.05E−05 | 0.003068 | 1.98E−05 | 3.44E−05 | 3.12E−05 |
| 135 | 0.003092 | 2.08E−05 | 5.01E−05 | 7.62E−06 | 0.003089 | 2.06E−05 | 4.55E−05 | 1.29E−05 | 0.003082 | 2.03E−05 | 4.03E−05 | 2.05E−05 | 0.003068 | 1.98E−05 | 3.48E−05 | 3.13E−05 |
| 136 | 0.003091 | 2.09E−05 | 5.06E−05 | 7.63E−06 | 0.003089 | 2.06E−05 | 4.59E−05 | 1.29E−05 | 0.003082 | 2.03E−05 | 4.07E−05 | 2.05E−05 | 0.003067 | 1.99E−05 | 3.51E−05 | 3.13E−05 |
| 137 | 0.003091 | 2.09E−05 | 5.11E−05 | 7.64E−06 | 0.003088 | 2.07E−05 | 4.64E−05 | 1.29E−05 | 0.003081 | 2.03E−05 | 4.11E−05 | 2.06E−05 | 0.003067 | 1.99E−05 | 3.55E−05 | 3.14E−05 |
| 138 | 0.00309 | 2.09E−05 | 5.17E−05 | 7.65E−06 | 0.003088 | 2.07E−05 | 4.68E−05 | 1.30E−05 | 0.003081 | 2.04E−05 | 4.15E−05 | 2.06E−05 | 0.003066 | 1.99E−05 | 3.58E−05 | 3.14E−05 |
| 139 | 0.00309 | 2.09E−05 | 5.22E−05 | 7.66E−06 | 0.003087 | 2.07E−05 | 4.73E−05 | 1.30E−05 | 0.00308 | 2.04E−05 | 4.19E−05 | 2.06E−05 | 0.003065 | 1.99E−05 | 3.62E−05 | 3.15E−05 |
| 140 | 0.003089 | 2.09E−05 | 5.27E−05 | 7.67E−06 | 0.003087 | 2.07E−05 | 4.78E−05 | 1.30E−05 | 0.00308 | 2.04E−05 | 4.23E−05 | 2.07E−05 | 0.003065 | 2.00E−05 | 3.66E−05 | 3.15E−05 |
| 141 | 0.003089 | 2.09E−05 | 5.32E−05 | 7.68E−06 | 0.003086 | 2.07E−05 | 4.82E−05 | 1.30E−05 | 0.003079 | 2.04E−05 | 4.27E−05 | 2.07E−05 | 0.003064 | 2.00E−05 | 3.69E−05 | 3.16E−05 |
| 142 | 0.003088 | 2.09E−05 | 5.37E−05 | 7.69E−06 | 0.003086 | 2.07E−05 | 4.87E−05 | 1.30E−05 | 0.003078 | 2.04E−05 | 4.31E−05 | 2.07E−05 | 0.003063 | 2.00E−05 | 3.73E−05 | 3.16E−05 |
| 143 | 0.003088 | 2.10E−05 | 5.42E−05 | 7.70E−06 | 0.003085 | 2.08E−05 | 4.91E−05 | 1.31E−05 | 0.003078 | 2.05E−05 | 4.35E−05 | 2.07E−05 | 0.003063 | 2.00E−05 | 3.76E−05 | 3.16E−05 |
| 144 | 0.003087 | 2.10E−05 | 5.47E−05 | 7.71E−06 | 0.003085 | 2.08E−05 | 4.96E−05 | 1.31E−05 | 0.003077 | 2.05E−05 | 4.39E−05 | 2.08E−05 | 0.003062 | 2.01E−05 | 3.80E−05 | 3.17E−05 |
| 145 | 0.003087 | 2.10E−05 | 5.52E−05 | 7.72E−06 | 0.003084 | 2.08E−05 | 5.01E−05 | 1.31E−05 | 0.003077 | 2.05E−05 | 4.44E−05 | 2.08E−05 | 0.003061 | 2.01E−05 | 3.83E−05 | 3.17E−05 |
| 146 | 0.003086 | 2.10E−05 | 5.57E−05 | 7.73E−06 | 0.003084 | 2.08E−05 | 5.05E−05 | 1.31E−05 | 0.003076 | 2.05E−05 | 4.48E−05 | 2.08E−05 | 0.003061 | 2.01E−05 | 3.87E−05 | 3.18E−05 |
| 147 | 0.003086 | 2.10E−05 | 5.62E−05 | 7.74E−06 | 0.003083 | 2.08E−05 | 5.10E−05 | 1.31E−05 | 0.003076 | 2.05E−05 | 4.52E−05 | 2.08E−05 | 0.00306 | 2.01E−05 | 3.91E−05 | 3.18E−05 |
| 148 | 0.003085 | 2.10E−05 | 5.67E−05 | 7.74E−06 | 0.003083 | 2.08E−05 | 5.14E−05 | 1.31E−05 | 0.003075 | 2.06E−05 | 4.56E−05 | 2.09E−05 | 0.00306 | 2.01E−05 | 3.94E−05 | 3.18E−05 |
| 149 | 0.003084 | 2.10E−05 | 5.72E−05 | 7.75E−06 | 0.003082 | 2.09E−05 | 5.19E−05 | 1.31E−05 | 0.003075 | 2.06E−05 | 4.60E−05 | 2.09E−05 | 0.003059 | 2.02E−05 | 3.98E−05 | 3.19E−05 |
| 150 | 0.003084 | 2.10E−05 | 5.77E−05 | 7.76E−06 | 0.003082 | 2.09E−05 | 5.24E−05 | 1.32E−05 | 0.003074 | 2.06E−05 | 4.64E−05 | 2.09E−05 | 0.003058 | 2.02E−05 | 4.01E−05 | 3.19E−05 |
| 151 | 0.003083 | 2.10E−05 | 5.82E−05 | 7.77E−06 | 0.003081 | 2.09E−05 | 5.28E−05 | 1.32E−05 | 0.003074 | 2.06E−05 | 4.68E−05 | 2.09E−05 | 0.003058 | 2.02E−05 | 4.05E−05 | 3.19E−05 |
| 152 | 0.003083 | 2.11E−05 | 5.87E−05 | 7.77E−06 | 0.003081 | 2.09E−05 | 5.33E−05 | 1.32E−05 | 0.003073 | 2.06E−05 | 4.72E−05 | 2.09E−05 | 0.003057 | 2.02E−05 | 4.08E−05 | 3.20E−05 |
| 153 | 0.003082 | 2.11E−05 | 5.92E−05 | 7.78E−06 | 0.00308 | 2.09E−05 | 5.38E−05 | 1.32E−05 | 0.003073 | 2.06E−05 | 4.77E−05 | 2.10E−05 | 0.003056 | 2.02E−05 | 4.12E−05 | 3.20E−05 |

**Table S17.** *Cont.*

| TIME (days) | cSAXA08:1 | cSCA08:1 | cESCA08:1 | cSFA08:1 | cSAXA10:1 | cSCA10:1 | cESCA10:1 | cSFA10:1 | cSAXA12:1 | cSCA12:1 | cESCA12:1 | cSFA12:1 | cSAXA14:1 | cSCA14:1 | cESCA14:1 | cSFA14:1 |
|---|---|---|---|---|---|---|---|---|---|---|---|---|---|---|---|---|
| 154 | 0.003082 | 2.11E−05 | 5.98E−05 | 7.79E−06 | 0.00308 | 2.09E−05 | 5.42E−05 | 1.32E−05 | 0.003072 | 2.07E−05 | 4.81E−05 | 2.10E−05 | 0.003056 | 2.03E−05 | 4.16E−05 | 3.20E−05 |
| 155 | 0.003081 | 2.11E−05 | 6.03E−05 | 7.79E−06 | 0.003079 | 2.09E−05 | 5.47E−05 | 1.32E−05 | 0.003071 | 2.07E−05 | 4.85E−05 | 2.10E−05 | 0.003055 | 2.03E−05 | 4.19E−05 | 3.20E−05 |
| 156 | 0.003081 | 2.11E−05 | 6.08E−05 | 7.80E−06 | 0.003078 | 2.09E−05 | 5.52E−05 | 1.32E−05 | 0.003071 | 2.07E−05 | 4.89E−05 | 2.10E−05 | 0.003055 | 2.03E−05 | 4.23E−05 | 3.21E−05 |
| 157 | 0.00308 | 2.11E−05 | 6.13E−05 | 7.81E−06 | 0.003078 | 2.09E−05 | 5.56E−05 | 1.32E−05 | 0.00307 | 2.07E−05 | 4.93E−05 | 2.10E−05 | 0.003054 | 2.03E−05 | 4.27E−05 | 3.21E−05 |
| 158 | 0.00308 | 2.11E−05 | 6.18E−05 | 7.81E−06 | 0.003077 | 2.10E−05 | 5.61E−05 | 1.32E−05 | 0.00307 | 2.07E−05 | 4.97E−05 | 2.10E−05 | 0.003053 | 2.03E−05 | 4.30E−05 | 3.21E−05 |
| 159 | 0.003079 | 2.11E−05 | 6.23E−05 | 7.82E−06 | 0.003077 | 2.10E−05 | 5.66E−05 | 1.33E−05 | 0.003069 | 2.07E−05 | 5.01E−05 | 2.11E−05 | 0.003053 | 2.03E−05 | 4.34E−05 | 3.21E−05 |
| 160 | 0.003079 | 2.11E−05 | 6.28E−05 | 7.82E−06 | 0.003076 | 2.10E−05 | 5.70E−05 | 1.33E−05 | 0.003069 | 2.07E−05 | 5.06E−05 | 2.11E−05 | 0.003052 | 2.04E−05 | 4.37E−05 | 3.22E−05 |
| 161 | 0.003078 | 2.11E−05 | 6.33E−05 | 7.83E−06 | 0.003076 | 2.10E−05 | 5.75E−05 | 1.33E−05 | 0.003068 | 2.07E−05 | 5.10E−05 | 2.11E−05 | 0.003052 | 2.04E−05 | 4.41E−05 | 3.22E−05 |
| 162 | 0.003078 | 2.11E−05 | 6.38E−05 | 7.83E−06 | 0.003075 | 2.10E−05 | 5.79E−05 | 1.33E−05 | 0.003068 | 2.08E−05 | 5.14E−05 | 2.11E−05 | 0.003051 | 2.04E−05 | 4.45E−05 | 3.22E−05 |
| 163 | 0.003077 | 2.11E−05 | 6.43E−05 | 7.84E−06 | 0.003075 | 2.10E−05 | 5.84E−05 | 1.33E−05 | 0.003067 | 2.08E−05 | 5.18E−05 | 2.11E−05 | 0.00305 | 2.04E−05 | 4.48E−05 | 3.22E−05 |
| 164 | 0.003077 | 2.12E−05 | 6.48E−05 | 7.84E−06 | 0.003074 | 2.10E−05 | 5.89E−05 | 1.33E−05 | 0.003067 | 2.08E−05 | 5.22E−05 | 2.11E−05 | 0.00305 | 2.04E−05 | 4.52E−05 | 3.23E−05 |
| 165 | 0.003076 | 2.12E−05 | 6.54E−05 | 7.85E−06 | 0.003074 | 2.10E−05 | 5.93E−05 | 1.33E−05 | 0.003066 | 2.08E−05 | 5.26E−05 | 2.11E−05 | 0.003049 | 2.04E−05 | 4.55E−05 | 3.23E−05 |
| 166 | 0.003076 | 2.12E−05 | 6.59E−05 | 7.85E−06 | 0.003073 | 2.10E−05 | 5.98E−05 | 1.33E−05 | 0.003066 | 2.08E−05 | 5.31E−05 | 2.12E−05 | 0.003048 | 2.05E−05 | 4.59E−05 | 3.23E−05 |
| 167 | 0.003075 | 2.12E−05 | 6.64E−05 | 7.86E−06 | 0.003073 | 2.10E−05 | 6.03E−05 | 1.33E−05 | 0.003065 | 2.08E−05 | 5.35E−05 | 2.12E−05 | 0.003048 | 2.05E−05 | 4.63E−05 | 3.23E−05 |
| 168 | 0.003075 | 2.12E−05 | 6.69E−05 | 7.86E−06 | 0.003072 | 2.10E−05 | 6.07E−05 | 1.33E−05 | 0.003065 | 2.08E−05 | 5.39E−05 | 2.12E−05 | 0.003047 | 2.05E−05 | 4.66E−05 | 3.23E−05 |
| 169 | 0.003074 | 2.12E−05 | 6.74E−05 | 7.86E−06 | 0.003072 | 2.11E−05 | 6.12E−05 | 1.33E−05 | 0.003064 | 2.08E−05 | 5.43E−05 | 2.12E−05 | 0.003047 | 2.05E−05 | 4.70E−05 | 3.23E−05 |
| 170 | 0.003074 | 2.12E−05 | 6.79E−05 | 7.87E−06 | 0.003071 | 2.11E−05 | 6.17E−05 | 1.33E−05 | 0.003063 | 2.08E−05 | 5.47E−05 | 2.12E−05 | 0.003046 | 2.05E−05 | 4.74E−05 | 3.24E−05 |
| 171 | 0.003073 | 2.12E−05 | 6.84E−05 | 7.87E−06 | 0.003071 | 2.11E−05 | 6.22E−05 | 1.33E−05 | 0.003063 | 2.09E−05 | 5.51E−05 | 2.12E−05 | 0.003045 | 2.05E−05 | 4.77E−05 | 3.24E−05 |
| 172 | 0.003072 | 2.12E−05 | 6.89E−05 | 7.88E−06 | 0.00307 | 2.11E−05 | 6.26E−05 | 1.34E−05 | 0.003062 | 2.09E−05 | 5.56E−05 | 2.12E−05 | 0.003045 | 2.05E−05 | 4.81E−05 | 3.24E−05 |
| 173 | 0.003072 | 2.12E−05 | 6.94E−05 | 7.88E−06 | 0.00307 | 2.11E−05 | 6.31E−05 | 1.34E−05 | 0.003062 | 2.09E−05 | 5.60E−05 | 2.12E−05 | 0.003044 | 2.05E−05 | 4.85E−05 | 3.24E−05 |
| 174 | 0.003071 | 2.12E−05 | 7.00E−05 | 7.88E−06 | 0.003069 | 2.11E−05 | 6.36E−05 | 1.34E−05 | 0.003061 | 2.09E−05 | 5.64E−05 | 2.12E−05 | 0.003044 | 2.06E−05 | 4.88E−05 | 3.24E−05 |
| 175 | 0.003071 | 2.12E−05 | 7.05E−05 | 7.89E−06 | 0.003069 | 2.11E−05 | 6.40E−05 | 1.34E−05 | 0.003061 | 2.09E−05 | 5.68E−05 | 2.13E−05 | 0.003043 | 2.06E−05 | 4.92E−05 | 3.24E−05 |

**Table S17.** *Cont.*

| TIME (days) | cSAXA08:1 | cSCA08:1 | cESCA08:1 | cSFA08:1 | cSAXA10:1 | cSCA10:1 | cESCA10:1 | cSFA10:1 | cSAXA12:1 | cSCA12:1 | cESCA12:1 | cSFA12:1 | cSAXA14:1 | cSCA14:1 | cESCA14:1 | cSFA14:1 |
|---|---|---|---|---|---|---|---|---|---|---|---|---|---|---|---|---|
| 176 | 0.00307 | 2.12E−05 | 7.10E−05 | 7.89E−06 | 0.003068 | 2.11E−05 | 6.45E−05 | 1.34E−05 | 0.00306 | 2.09E−05 | 5.72E−05 | 2.13E−05 | 0.003042 | 2.06E−05 | 4.96E−05 | 3.25E−05 |
| 177 | 0.00307 | 2.12E−05 | 7.15E−05 | 7.89E−06 | 0.003068 | 2.11E−05 | 6.50E−05 | 1.34E−05 | 0.00306 | 2.09E−05 | 5.77E−05 | 2.13E−05 | 0.003042 | 2.06E−05 | 4.99E−05 | 3.25E−05 |
| 178 | 0.003069 | 2.12E−05 | 7.20E−05 | 7.90E−06 | 0.003067 | 2.11E−05 | 6.54E−05 | 1.34E−05 | 0.003059 | 2.09E−05 | 5.81E−05 | 2.13E−05 | 0.003041 | 2.06E−05 | 5.03E−05 | 3.25E−05 |
| 179 | 0.003069 | 2.12E−05 | 7.25E−05 | 7.90E−06 | 0.003067 | 2.11E−05 | 6.59E−05 | 1.34E−05 | 0.003059 | 2.09E−05 | 5.85E−05 | 2.13E−05 | 0.003041 | 2.06E−05 | 5.07E−05 | 3.25E−05 |
| 180 | 0.003068 | 2.12E−05 | 7.30E−05 | 7.90E−06 | 0.003066 | 2.11E−05 | 6.64E−05 | 1.34E−05 | 0.003058 | 2.09E−05 | 5.89E−05 | 2.13E−05 | 0.00304 | 2.06E−05 | 5.10E−05 | 3.25E−05 |
| 181 | 0.003068 | 2.12E−05 | 7.35E−05 | 7.90E−06 | 0.003066 | 2.11E−05 | 6.68E−05 | 1.34E−05 | 0.003058 | 2.09E−05 | 5.93E−05 | 2.13E−05 | 0.003039 | 2.06E−05 | 5.14E−05 | 3.25E−05 |
| 182 | 0.003067 | 2.12E−05 | 7.41E−05 | 7.91E−06 | 0.003065 | 2.11E−05 | 6.73E−05 | 1.34E−05 | 0.003057 | 2.09E−05 | 5.98E−05 | 2.13E−05 | 0.003039 | 2.06E−05 | 5.18E−05 | 3.25E−05 |
| 183 | 0.003067 | 2.12E−05 | 7.46E−05 | 7.91E−06 | 0.003065 | 2.11E−05 | 6.78E−05 | 1.34E−05 | 0.003057 | 2.10E−05 | 6.02E−05 | 2.13E−05 | 0.003038 | 2.06E−05 | 5.21E−05 | 3.25E−05 |
| 184 | 0.003066 | 2.12E−05 | 7.51E−05 | 7.91E−06 | 0.003064 | 2.11E−05 | 6.82E−05 | 1.34E−05 | 0.003056 | 2.10E−05 | 6.06E−05 | 2.13E−05 | 0.003038 | 2.07E−05 | 5.25E−05 | 3.25E−05 |
| 185 | 0.003066 | 2.12E−05 | 7.56E−05 | 7.91E−06 | 0.003064 | 2.11E−05 | 6.87E−05 | 1.34E−05 | 0.003056 | 2.10E−05 | 6.10E−05 | 2.13E−05 | 0.003037 | 2.07E−05 | 5.29E−05 | 3.26E−05 |
| 186 | 0.003065 | 2.12E−05 | 7.61E−05 | 7.92E−06 | 0.003063 | 2.12E−05 | 6.92E−05 | 1.34E−05 | 0.003055 | 2.10E−05 | 6.14E−05 | 2.13E−05 | 0.003036 | 2.07E−05 | 5.32E−05 | 3.26E−05 |
| 187 | 0.003065 | 2.13E−05 | 7.66E−05 | 7.92E−06 | 0.003063 | 2.12E−05 | 6.96E−05 | 1.34E−05 | 0.003055 | 2.10E−05 | 6.19E−05 | 2.13E−05 | 0.003036 | 2.07E−05 | 5.36E−05 | 3.26E−05 |
| 188 | 0.003064 | 2.13E−05 | 7.71E−05 | 7.92E−06 | 0.003062 | 2.12E−05 | 7.01E−05 | 1.34E−05 | 0.003054 | 2.10E−05 | 6.23E−05 | 2.13E−05 | 0.003035 | 2.07E−05 | 5.40E−05 | 3.26E−05 |
| 189 | 0.003064 | 2.13E−05 | 7.76E−05 | 7.92E−06 | 0.003062 | 2.12E−05 | 7.06E−05 | 1.34E−05 | 0.003053 | 2.10E−05 | 6.27E−05 | 2.14E−05 | 0.003034 | 2.07E−05 | 5.43E−05 | 3.26E−05 |
| 190 | 0.003063 | 2.13E−05 | 7.82E−05 | 7.92E−06 | 0.003061 | 2.12E−05 | 7.11E−05 | 1.34E−05 | 0.003053 | 2.10E−05 | 6.31E−05 | 2.14E−05 | 0.003034 | 2.07E−05 | 5.47E−05 | 3.26E−05 |
| 191 | 0.003063 | 2.13E−05 | 7.87E−05 | 7.93E−06 | 0.003061 | 2.12E−05 | 7.15E−05 | 1.34E−05 | 0.003052 | 2.10E−05 | 6.35E−05 | 2.14E−05 | 0.003033 | 2.07E−05 | 5.51E−05 | 3.26E−05 |
| 192 | 0.003062 | 2.13E−05 | 7.92E−05 | 7.93E−06 | 0.00306 | 2.12E−05 | 7.20E−05 | 1.34E−05 | 0.003052 | 2.10E−05 | 6.40E−05 | 2.14E−05 | 0.003033 | 2.07E−05 | 5.54E−05 | 3.26E−05 |
| 193 | 0.003062 | 2.13E−05 | 7.97E−05 | 7.93E−06 | 0.00306 | 2.12E−05 | 7.25E−05 | 1.34E−05 | 0.003051 | 2.10E−05 | 6.44E−05 | 2.14E−05 | 0.003032 | 2.07E−05 | 5.58E−05 | 3.26E−05 |
| 194 | 0.003061 | 2.13E−05 | 8.02E−05 | 7.93E−06 | 0.003059 | 2.12E−05 | 7.29E−05 | 1.35E−05 | 0.003051 | 2.10E−05 | 6.48E−05 | 2.14E−05 | 0.003031 | 2.07E−05 | 5.62E−05 | 3.26E−05 |
| 195 | 0.003061 | 2.13E−05 | 8.07E−05 | 7.93E−06 | 0.003059 | 2.12E−05 | 7.34E−05 | 1.35E−05 | 0.00305 | 2.10E−05 | 6.52E−05 | 2.14E−05 | 0.003031 | 2.07E−05 | 5.65E−05 | 3.26E−05 |
| 196 | 0.00306 | 2.13E−05 | 8.12E−05 | 7.93E−06 | 0.003058 | 2.12E−05 | 7.39E−05 | 1.35E−05 | 0.00305 | 2.10E−05 | 6.56E−05 | 2.14E−05 | 0.00303 | 2.07E−05 | 5.69E−05 | 3.26E−05 |
| 197 | 0.00306 | 2.13E−05 | 8.17E−05 | 7.93E−06 | 0.003058 | 2.12E−05 | 7.43E−05 | 1.35E−05 | 0.003049 | 2.10E−05 | 6.61E−05 | 2.14E−05 | 0.00303 | 2.08E−05 | 5.73E−05 | 3.26E−05 |

**Table S17.** *Cont.*

| TIME (days) | cSAXA08:1 | cSCA08:1 | cESCA08:1 | cSFA08:1 | cSAXA10:1 | cSCA10:1 | cESCA10:1 | cSFA1 0:1 | cSAXA12:1 | cSCA12:1 | cESCA12:1 | cSFA12:1 | cSAXA14:1 | cSCA14:1 | cESCA14:1 | cSFA14:1 |
|---|---|---|---|---|---|---|---|---|---|---|---|---|---|---|---|---|
| 198 | 0.003059 | 2.13E−05 | 8.23E−05 | 7.94E−06 | 0.003057 | 2.12E−05 | 7.48E−05 | 1.35E−05 | 0.003049 | 2.10E−05 | 6.65E−05 | 2.14E−05 | 0.003029 | 2.08E−05 | 5.76E−05 | 3.26E−05 |
| 199 | 0.003059 | 2.13E−05 | 8.28E−05 | 7.94E−06 | 0.003057 | 2.12E−05 | 7.53E−05 | 1.35E−05 | 0.003048 | 2.10E−05 | 6.69E−05 | 2.14E−05 | 0.003028 | 2.08E−05 | 5.80E−05 | 3.27E−05 |
| 200 | 0.003058 | 2.13E−05 | 8.33E−05 | 7.94E−06 | 0.003056 | 2.12E−05 | 7.58E−05 | 1.35E−05 | 0.003048 | 2.10E−05 | 6.73E−05 | 2.14E−05 | 0.003028 | 2.08E−05 | 5.84E−05 | 3.27E−05 |

## *4.1. Determined Rate Constants*

| $r_i$ | $k_{opt}$ |
|---|---|
| $r_{1(k1)}$ | 0.0867 $d^{-1}$ |
| $r_{2(k2)}$ | 0.00585 $d^{-1}$ |
| $r_{3(k3)}$ | 0.655 $d^{-1}$ |
| $r_{4(k4)}$ | 0.0555 $d^{-1}$ |
| $r_{5(k5)}$ | 1.68E−04 L $mol^{-1}$ $d^{-1}$ |
| $r_{6(k6)}$ | 0.0241 L $mol^{-1}$ $d^{-1}$ |
| $r_{7(k7)}$ | 0.591 $g_{SAXA}$ $mol^{-2}$ $d^{-}$ |
| $r_{7(k8)}$ | 0.025472 $d^{-1}$ |

## *4.2. Ab Initio Calculations*

### 4.2.1. Structures

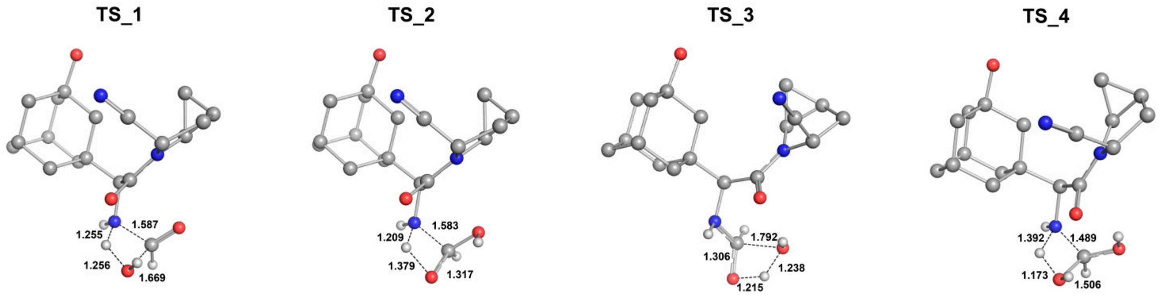


**Figure S1.** Structure of the transition state parallel reaction 1 in gas phase. Some hydrogen atoms are omitted for clarity.

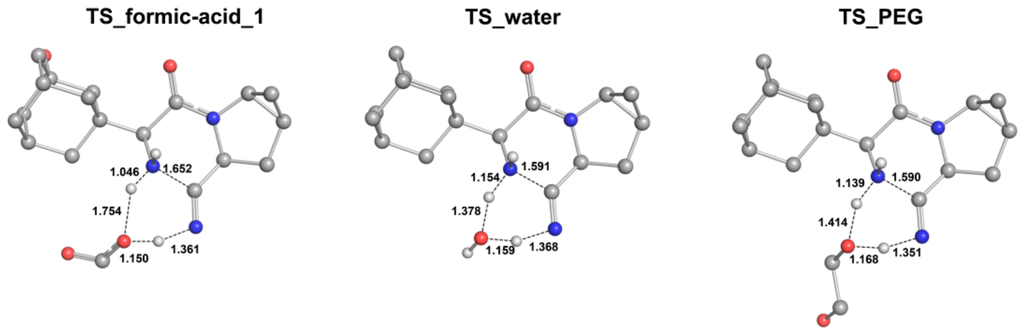


**Figure S2.** Structure of the transition state parallel reaction 2 in gas phase catalyzed by the hydroxyl group.

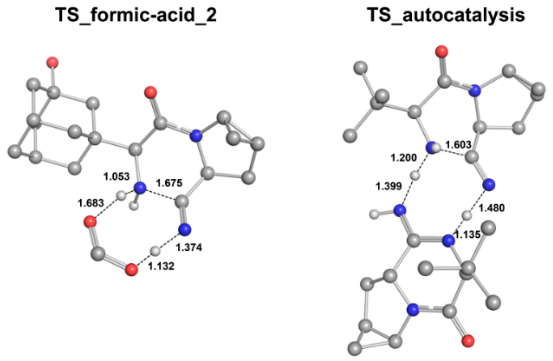


**Figure S3.** Structure of the transition state parallel reaction 2 in gas phase catalyzed by formic acid and ESCA.

## 4.2.2. Calculated Energies

**Table S18.** Calculated energy for the species involved in the reaction 1 in gas phase and water.

| | gas phase | | | water | | |
|---|---|---|---|---|---|---|
| | **Energy (Hartree)** | **$\Delta G_{298}$ (kcal/mol)** | **Imaginary ν ($cm^{-1}$)** | **Energy (Hartree)** | **$\Delta G_{298}$ (kcal/mol)** | **Imaginary ν ($cm^{-1}$)** |
| saxagliptin | −1014.900158 | n.a. | n.a. | −1014.917957 | n.a. | n.a. |
| formic acid | −189.664848 | n.a. | n.a. | −189.676737 | n.a. | n.a. |
| TS_1 | −1204.502403 | 39.28 | −1356 | −1204.525945 | 43.14 | −1357 |
| TS_2 | −1204.504560 | 37.93 | −1415 | −1204.531874 | 39.42 | −1495 |
| Intermediate_1 | −1204.559858 | 3.23 | n.a. | −1204.581799 | 8.09 | n.a. |
| TS_3 | −1204.506670 | 36.61 | −1670 | −1204.530424 | 40.33 | −1670 |
| SFA | −1128.187660 | −1.57 | n.a. | −1128.210647 | −2.06 | n.a. |
| $H_2O$ | −76.379845 | | n.a. | −76.387335 | | n.a. |

**Table S19.** Calculated energy for the species involved in the reaction 1 in gas phase, water and formic acid.

| **Structure** | gas phase | | | water | | | formic acid | | |
|---|---|---|---|---|---|---|---|---|---|
| | **Energy (Hartree)** | **$\Delta G_{298}$ (kcal/mol)** | **Imaginary ν ($cm^{-1}$)** | **Energy (Hartree)** | **$\Delta G_{298}$ (kcal/mol)** | **Imaginary ν ($cm^{-1}$)** | **Energy (Hartree)** | **$\Delta G_{298}$ (kcal/mol)** | **Imaginary ν ($cm^{-1}$)** |
| saxagliptin | −1014.900158 | n.a. | n.a. | −1014.917957 | n.a. | n.a. | −1014.917815 | n.a. | n.a. |
| $HCOOH_2^+$ | −189.955557 | n.a. | n.a. | −190.064963 | n.a. | n.a. | −190.064454 | n.a. | n.a. |
| Intermediate_2 | −1204.924983 | −43.47 | n.a. | −1205.009268 | −16.53 | n.a. | −1205.008647 | −16.55 | n.a. |
| TS_4 | −1204.870810 | −9.47 | −1381 | −1204.953494 | 18.47 | −1413 | −1204.952966 | 18.39 | −1413 |
| $H_2O$ | −76.379845 | −37.99 | n.a. | −76.387335 | −36.22 | n.a. | −76.387284 | −17.74 | n.a. |
| Intermediate_3 | −1128.536412 | | n.a. | −1128.623875 | | n.a. | −1128.623260 | | n.a. |
| $HCOO^-$ | −189.105983 | n.a. | n.a. | −189.211820 | n.a. | n.a. | −189.211336 | n.a. | n.a. |
| HCOOH | −189.664848 | −169.84 | n.a. | −189.676737 | −50.19 | n.a. | −189.676648 | −50.73 | n.a. |
| SFA | −1128.187660 | | n.a. | −1128.210647 | | n.a. | −1128.210516 | | n.a. |

**Table S20.** Calculated energy for the species involved in the parallel reaction 2 catalyzed by the hydroxyl group in gas phase, water and formic acid.

| Structure | gas phase | | | water | | | formic acid | | |
|---|---|---|---|---|---|---|---|---|---|
| | **Energy (Hartree)** | **ΔG298 (kcal/mol)** | Imaginary ν (cm−1) | Energy (Hartree) | ΔG298 (kcal/mol) | Imaginary ν (cm−1) | Energy (Hartree) | ΔG298 (kcal/mol) | Imaginary ν (cm−1) |
| **saxagliptin** | **−1014.900158** | **n.a.** | n.a. | −1014.917957 | n.a. | n.a. | −1014.917815 | n.a. | n.a. |
| formic acid | −189.664848 | n.a. | n.a. | −189.676737 | n.a. | n.a. | −189.676648 | n.a. | n.a. |
| TS_formic_acid_1 | −1204.528428 | 22.95 | −618 | / | / | / | / | / | / |
| TS_formic_acid_2 | −1204.546003 | 11.92 | −309 | −1204.566677 | 17.58 | −182 | −1204.566543 | 17.52 | −182 |
| $H_2O$ | −76.379845 | n.a. | n.a. | −76.387335 | n.a. | n.a. | / | / | / |
| TS_water | −1091.233322 | 29.29 | −1146 | −1091.255101 | 31.5 | −1162 | / | / | / |
| $HOCH_2CH_2OH$[1] | −230.088873 | n.a. | n.a. | −230.097524 | n.a. | n.a. | / | / | / |
| TS_PEG | −1244.942158 | 29.41 | −1086 | −1244.966634 | 30.65 | −1070 | / | / | / |
| SCA | −1014.912519 | −7.76 | n.a. | −1014.929 | −6.76 | n.a. | −1014.928583 | −6.76 | n.a. |
| ESCA | −1014.916325 | −10.14 | n.a. | −1014.934 | −10.02 | n.a. | −1014.933780 | −10.02 | n.a. |

[1]Ethylene glycol was used as simplified model for PEG. /: unavailable.

**Table S21.** Calculated energy for the species involved in the parallel reaction 2 catalyzed by ESCA in gas phase, water and formic acid.

| **Structure** | **gas phase** | | | **water** | | | **formic acid** | | |
|---|---|---|---|---|---|---|---|---|---|
| | **Energy (Hartree)** | **ΔG298 (kcal/mol)** | **Imaginary ν (cm−1)** | **Energy (Hartree)** | **ΔG298 (kcal/mol)** | **Imaginary ν (cm−1)** | **Energy (Hartree)** | **ΔG298 (kcal/mol)** | **Imaginary ν (cm−1)** |
| Saxagliptin_s | −707.620810 | n.a. | n.a. | −707.636070 | n.a. | n.a. | −707.635950 | n.a. | n.a. |
| SCA_s | −707.634373 | −8.51 | n.a. | −707.647002 | −6.86 | n.a. | −707.646895 | −6.87 | n.a. |
| ESCA_s | −707.638442 | −11.06 | n.a. | −707.651746 | −9.84 | n.a. | −707.651631 | −9.84 | n.a. |
| TS_autocatalysis | −1415.225847 | 9.90 | −765 | −1415.249553 | 14.17 | −776 | −1415.249126 | 14.29 | −777 |

### 4.2.3. Calculated Coordinates

**Table S22.** Coordinates of saxagliptin (SAXA).

| in gas phase | | | | in water | | | | in formic acid | | | |
|---|---|---|---|---|---|---|---|---|---|---|---|
| **O** | **1.312629** | **−2.91072** | **−0.20037** | **O** | **1.339106** | **−2.91201** | **−0.11407** | **O** | 1.338949 | −2.91201 | −0.11443 |
| O | −1.36713 | 2.011005 | 1.292291 | O | −1.32635 | 1.862865 | 1.427392 | O | −1.32674 | 1.864144 | 1.426323 |
| N | −2.2633 | 0.649949 | −0.24928 | N | −2.2759 | 0.648358 | −0.20325 | N | −2.27579 | 0.648404 | −0.2037 |
| N | 0.358756 | 3.155111 | −0.57124 | N | 0.312847 | 3.157438 | −0.46179 | N | 0.313303 | 3.157377 | −0.46262 |
| N | −2.27692 | −1.38713 | 2.49493 | N | −2.31168 | −1.50213 | 2.446358 | N | −2.31245 | −1.50118 | 2.446956 |
| C | 1.116611 | 0.813203 | −0.31154 | C | 1.120372 | 0.816491 | −0.28514 | C | 1.120407 | 0.816414 | −0.28533 |
| C | 1.833713 | −1.59703 | −0.07654 | C | 1.856582 | −1.58858 | −0.05169 | C | 1.856473 | −1.58867 | −0.05168 |
| C | 3.449928 | 0.102126 | −0.96744 | C | 3.414931 | 0.094732 | −1.06278 | C | 3.415239 | 0.094668 | −1.06214 |
| C | 2.793279 | 0.145237 | 1.451292 | C | 2.90035 | 0.179863 | 1.388631 | C | 2.899716 | 0.179707 | 1.389084 |
| C | 0.674834 | −0.65634 | −0.40094 | C | 0.676965 | −0.6568 | −0.32961 | C | 0.67697 | −0.65685 | −0.32992 |
| C | 2.277586 | 1.036934 | −1.29889 | C | 2.223697 | 1.02278 | −1.34095 | C | 2.224104 | 1.022707 | −1.34075 |
| C | 1.624358 | 1.086003 | 1.118404 | C | 1.710788 | 1.110907 | 1.107691 | C | 1.710298 | 1.110813 | 1.107729 |
| C | 2.979821 | −1.35649 | −1.06537 | C | 2.941724 | −1.36564 | −1.11019 | C | 2.94203 | −1.36569 | −1.10976 |
| C | 2.3196 | −1.31309 | 1.348723 | C | 2.425552 | −1.28 | 1.337366 | C | 2.424935 | −1.28016 | 1.337589 |
| C | 3.94182 | 0.382655 | 0.45996 | C | 3.988927 | 0.399845 | 0.328499 | C | 3.988696 | 0.399744 | 0.329373 |
| C | −0.05316 | 1.761295 | −0.69513 | C | −0.07157 | 1.757142 | −0.61682 | C | −0.07136 | 1.757133 | −0.6174 |
| C | −2.35822 | −0.12237 | −1.46712 | C | −2.39881 | −0.01791 | −1.48284 | C | −2.39831 | −0.01883 | −1.48281 |
| C | −3.67334 | −0.84385 | −1.43151 | C | −3.71541 | −0.73312 | −1.48297 | C | −3.71493 | −0.73407 | −1.48278 |
| C | −2.38644 | −1.61821 | −1.32346 | C | −2.43318 | −1.52076 | −1.44761 | C | −2.43267 | −1.52164 | −1.44654 |
| C | −4.38591 | −0.43138 | −0.15584 | C | −4.41581 | −0.41624 | −0.17461 | C | −4.41564 | −0.41618 | −0.17484 |
| C | −1.27347 | 1.48506 | 0.190369 | C | −1.27026 | 1.427857 | 0.27784 | C | −1.27026 | 1.428302 | 0.277169 |
| C | −3.29924 | 0.2569 | 0.704941 | C | −3.32464 | 0.217454 | 0.721465 | C | −3.3246 | 0.217909 | 0.72112 |
| C | −2.72933 | −0.65385 | 1.724492 | C | −2.76155 | −0.7437 | 1.698546 | C | −2.76183 | −0.74289 | 1.698724 |
| H | 4.263778 | 0.275231 | −1.68083 | H | 4.185363 | 0.255113 | −1.82517 | H | 4.185969 | 0.255085 | −1.82423 |
| H | 3.136686 | 0.346944 | 2.472052 | H | 3.302774 | 0.398831 | 2.384004 | H | 3.301753 | 0.398639 | 2.384619 |
| H | −0.13735 | −0.8683 | 0.306565 | H | −0.09984 | −0.85622 | 0.421901 | H | −0.09999 | −0.85632 | 0.421407 |
| H | 0.307946 | −0.89046 | −1.40945 | H | 0.25606 | −0.90121 | −1.31398 | H | 0.256352 | −0.90133 | −1.3144 |
| H | 1.925705 | 0.843671 | −2.32264 | H | 1.813285 | 0.814501 | −2.3389 | H | 1.814053 | 0.814352 | −2.33885 |
| H | 2.587651 | 2.085388 | −1.25252 | H | 2.540429 | 2.070642 | −1.32706 | H | 2.540771 | 2.070584 | −1.32684 |
| H | 1.960233 | 2.127652 | 1.19122 | H | 2.044752 | 2.1548 | 1.1462 | H | 2.044342 | 2.154677 | 1.146369 |
| H | 0.811269 | 0.948166 | 1.839992 | H | 0.939788 | 0.979129 | 1.875078 | H | 0.939003 | 0.979094 | 1.874826 |

**Table S22.** *Cont.*

| in gas phase | | | | in water | | | | in formic acid | | | |
|---|---|---|---|---|---|---|---|---|---|---|---|
| H | 2.628318 | −1.59 | −2.0778 | H | 2.531377 | −1.61113 | −2.09754 | H | 2.532059 | −1.61118 | −2.09727 |
| H | 3.808259 | −2.04232 | −0.83674 | H | 3.781793 | −2.04753 | −0.91877 | H | 3.782021 | −2.0476 | −0.91806 |
| H | 3.143543 | −1.99795 | 1.597265 | H | 3.260716 | −1.9618 | 1.548963 | H | 3.260076 | −1.96194 | 1.549432 |
| H | 1.498309 | −1.50995 | 2.048049 | H | 1.646683 | −1.46313 | 2.087994 | H | 1.645816 | −1.46338 | 2.087908 |
| H | 4.790163 | −0.27048 | 0.700963 | H | 4.849442 | −0.24957 | 0.531783 | H | 4.849155 | −0.24966 | 0.532953 |
| H | 4.293717 | 1.4185 | 0.537226 | H | 4.342968 | 1.437218 | 0.367035 | H | 4.342711 | 1.437119 | 0.368079 |
| H | −1.97917 | 0.351152 | −2.36403 | H | −2.03348 | 0.523366 | −2.34546 | H | −2.03277 | 0.521825 | −2.34573 |
| H | −0.29265 | 1.56404 | −1.74672 | H | −0.32903 | 1.590079 | −1.66745 | H | −0.32868 | 1.589854 | −1.66806 |
| H | −4.26523 | −0.94267 | −2.33398 | H | −4.31444 | −0.75962 | −2.38493 | H | −4.31374 | −0.76124 | −2.38487 |
| H | −2.0947 | −2.04959 | −0.37026 | H | −2.13662 | −2.01419 | −0.52601 | H | −2.13621 | −2.01445 | −0.52459 |
| H | −2.08773 | −2.19954 | −2.18865 | H | −2.14937 | −2.04151 | −2.35503 | H | −2.14865 | −2.04303 | −2.35354 |
| H | −4.84887 | −1.26413 | 0.377735 | H | −4.8706 | −1.28615 | 0.303293 | H | −4.87071 | −1.28569 | 0.303529 |
| H | −5.16241 | 0.300027 | −0.39316 | H | −5.1964 | 0.32669 | −0.34837 | H | −5.19607 | 0.326761 | −0.34934 |
| H | −3.66168 | 1.142289 | 1.232395 | H | −3.68937 | 1.073305 | 1.293764 | H | −3.68935 | 1.074063 | 1.292963 |
| H | 2.004899 | −3.52674 | 0.068761 | H | 2.070904 | −3.51752 | 0.063679 | H | 2.070329 | −3.51758 | 0.06477 |
| H | 0.248803 | 3.414632 | 0.407983 | H | 0.237794 | 3.385297 | 0.528402 | H | 0.238323 | 3.385367 | 0.527543 |
| H | −0.28792 | 3.745702 | −1.0861 | H | −0.37594 | 3.742081 | −0.92827 | H | −0.37534 | 3.742131 | −0.92916 |

**Table S23.** Coordinates of SFA.

| In gas phase | | | | In water | | | | In Formic acid | | | |
|---|---|---|---|---|---|---|---|---|---|---|---|
| **C** | **−1.110813** | **0.377372** | **0.324431** | **C** | **1.114217** | **0.373514** | **−0.31621** | **C** | 1.114122 | 0.373535 | −0.31631 |
| C | 0.020863 | 1.396249 | 0.652731 | C | −0.032354 | 1.355641 | −0.69733 | C | −0.03241 | 1.355945 | −0.69684 |
| C | −0.502828 | −1.034649 | 0.263007 | C | 0.56018 | −1.064273 | −0.3185 | C | 0.559771 | −1.06413 | −0.31808 |
| C | −2.158374 | 0.398067 | 1.45441 | C | 2.222784 | 0.464034 | −1.38303 | C | 2.222129 | 0.463619 | −1.38374 |
| C | −1.821357 | 0.675372 | −1.0081 | C | 1.735189 | 0.662052 | 1.062769 | C | 1.735889 | 0.662223 | 1.062275 |
| C | 1.186833 | 1.198021 | −0.31843 | C | −1.230342 | 1.179781 | 0.238192 | C | −1.23004 | 1.179769 | 0.239074 |
| N | −0.398944 | 2.786662 | 0.699716 | N | 0.369422 | 2.754466 | −0.7683 | N | 0.369516 | 2.754727 | −0.76747 |
| C | −1.578007 | −2.091862 | −0.00227 | C | 1.668462 | −2.080113 | −0.01936 | C | 1.667992 | −2.08016 | −0.01928 |
| C | −3.245395 | −0.657567 | 1.193368 | C | 3.337371 | −0.549631 | −1.08282 | C | 3.336666 | −0.55023 | −1.08394 |
| C | −2.905887 | −0.38201 | −1.27237 | C | 2.84786 | −0.355092 | 1.365548 | C | 2.848505 | −0.35509 | 1.364638 |
| N | 2.310408 | 0.565289 | 0.141005 | N | −2.306075 | 0.484231 | −0.21585 | N | −2.30625 | 0.484922 | −0.21513 |
| O | 1.11764 | 1.532242 | −1.49216 | O | −1.219595 | 1.617336 | 1.386162 | O | −1.21863 | 1.61623 | 1.387385 |

**Table S23.** *Cont.*

| In gas phase | | | | In water | | | | In Formic acid | | | |
|---|---|---|---|---|---|---|---|---|---|---|---|
| C | −2.607898 | −2.054196 | 1.12631 | C | 2.755987 | −1.971 | −1.08732 | C | 2.754986 | −1.97149 | −1.08783 |
| C | −2.264806 | −1.776352 | −1.33547 | C | 2.266107 | −1.776189 | 1.358027 | C | 2.266423 | −1.77605 | 1.357717 |
| O | −1.001696 | −3.384949 | −0.00525 | O | 1.155162 | −3.403499 | −0.08079 | O | 1.154358 | −3.40341 | −0.08015 |
| C | −3.941909 | −0.349642 | −0.13977 | C | 3.943644 | −0.250593 | 0.295668 | C | 3.943744 | −0.25104 | 0.294157 |
| C | 2.609959 | 0.039382 | 1.454319 | C | −2.520657 | −0.175268 | −1.48839 | C | −2.52163 | −0.17317 | −1.48824 |
| C | 3.272383 | 0.082084 | −0.85189 | C | −3.347387 | 0.122817 | 0.750211 | C | −3.34682 | 0.122261 | 0.751249 |
| C | 3.967858 | −0.590403 | 1.377394 | C | −3.874638 | −0.81284 | −1.43231 | C | −3.87563 | −0.81071 | −1.43201 |
| C | 2.74907 | −1.454316 | 1.567726 | C | −2.640875 | −1.673821 | −1.44311 | C | −2.64192 | −1.67177 | −1.44465 |
| C | 4.502361 | −0.359224 | −0.02483 | C | −4.504973 | −0.4516 | −0.1002 | C | −4.50509 | −0.45082 | −0.09911 |
| C | 2.678214 | −1.04903 | −1.60521 | C | −2.803198 | −0.865272 | 1.71112 | C | −2.80211 | −0.86739 | 1.710246 |
| N | 2.192998 | −1.953731 | −2.13693 | N | −2.364203 | −1.646729 | 2.441425 | N | −2.36275 | −1.65021 | 2.438869 |
| H | 0.357179 | 1.152391 | 1.66497 | H | −0.329088 | 1.090426 | −1.7143 | H | −0.32953 | 1.091048 | −1.7138 |
| H | 0.004372 | −1.278078 | 1.207083 | H | 0.114393 | −1.302831 | −1.29325 | H | 0.113518 | −1.3028 | −1.2926 |
| H | 0.240219 | −1.098694 | −0.54306 | H | −0.225783 | −1.175344 | 0.443568 | H | −0.22587 | −1.17495 | 0.444345 |
| H | −1.666804 | 0.19546 | 2.417096 | H | 1.794458 | 0.268135 | −2.37561 | H | 1.793232 | 0.267564 | −2.37605 |
| H | −2.6122 | 1.395311 | 1.517588 | H | 2.634645 | 1.480373 | −1.39675 | H | 2.634199 | 1.479874 | −1.3979 |
| H | −1.094794 | 0.688218 | −1.82719 | H | 0.963053 | 0.623004 | 1.839058 | H | 0.964152 | 0.62349 | 1.838961 |
| H | −2.289659 | 1.666194 | −0.96071 | H | 2.16652 | 1.67033 | 1.071117 | H | 2.167407 | 1.670427 | 1.07015 |
| H | −0.653185 | 3.168004 | 1.601132 | H | 0.508227 | 3.147161 | −1.69082 | H | 0.509237 | 3.147481 | −1.68982 |
| H | −3.975951 | −0.627973 | 2.009412 | H | 4.111119 | −0.469537 | −1.85387 | H | 4.11002 | −0.47045 | −1.85543 |
| H | −3.394312 | −0.159037 | −2.22716 | H | 3.270426 | −0.137441 | 2.352335 | H | 3.271642 | −0.13734 | 2.351156 |
| H | −2.10806 | −2.305088 | 2.069875 | H | 2.320435 | −2.21064 | −2.06521 | H | 2.318892 | −2.2113 | −2.06543 |
| H | −3.368757 | −2.821392 | 0.942214 | H | 3.53836 | −2.710627 | −0.87907 | H | 3.537294 | −2.71124 | −0.87982 |
| H | −1.520418 | −1.817309 | −2.14339 | H | 1.482124 | −1.872996 | 2.122139 | H | 1.482808 | −1.87244 | 2.122265 |
| H | −3.018 | −2.547068 | −1.53722 | H | 3.043386 | −2.516806 | 1.581225 | H | 3.043608 | −2.51683 | 1.580682 |
| H | −0.365414 | −3.417184 | −0.7325 | H | 0.474981 | −3.484529 | 0.60154 | H | 0.476273 | −3.48484 | 0.604202 |
| H | −4.419979 | 0.636588 | −0.09588 | H | 4.379324 | 0.755867 | 0.30302 | H | 4.379663 | 0.755323 | 0.30108 |
| H | −4.730558 | −1.088261 | −0.32753 | H | 4.750674 | −0.960949 | 0.511993 | H | 4.750733 | −0.96153 | 0.510171 |
| H | 2.311188 | 0.652177 | 2.294808 | H | −2.167053 | 0.345061 | −2.36845 | H | −2.16862 | 0.348209 | −2.36792 |
| H | 3.49044 | 0.875256 | −1.57006 | H | −3.640339 | 1.001848 | 1.327861 | H | −3.63916 | 1.000508 | 1.33039 |
| H | 4.659358 | −0.486808 | 2.205132 | H | −4.507544 | −0.805705 | −2.31105 | H | −4.50911 | −0.8026 | −2.31035 |
| H | 2.608974 | −1.892194 | 2.549615 | H | −2.422555 | −2.213823 | −2.35717 | H | −2.42436 | −2.21075 | −2.35951 |
| H | 2.389688 | −2.063777 | 0.743941 | H | −2.339206 | −2.178624 | −0.5294 | H | −2.3396 | −2.17769 | −0.53179 |

**Table S23.** *Cont.*

| In gas phase | | | | In water | | | | In Formic acid | | | |
|---|---|---|---|---|---|---|---|---|---|---|---|
| H | 5.22597 | 0.459099 | −0.00566 | H | −5.249513 | 0.332427 | −0.24883 | H | −5.24951 | 0.333575 | −0.24645 |
| H | 4.983867 | −1.235654 | −0.46362 | H | −4.988202 | −1.292832 | 0.40032 | H | −4.98825 | −1.2925 | 0.400734 |
| C | −0.693289 | 3.62101 | −0.34861 | C | 0.620633 | 3.609661 | 0.255084 | C | 0.620934 | 3.609724 | 0.25617 |
| H | −0.454666 | 3.194454 | −1.33269 | H | 0.46475 | 3.171392 | 1.2485 | H | 0.464378 | 3.171441 | 1.249486 |
| O | −1.149893 | 4.732454 | −0.18049 | O | 0.970726 | 4.767669 | 0.078604 | O | 0.971805 | 4.767444 | 0.07981 |

**Table S24.** Coordinates of TS_1.

| In gas phase | | | | In water | | | |
|---|---|---|---|---|---|---|---|
| **O** | **2.35815** | **−2.8031** | **−0.6736** | **O** | **2.27813** | −2.9147 | −0.4122 |
| O | −1.4458 | 0.94446 | 1.63491 | O | −1.4479 | 0.97614 | 1.56621 |
| N | −2.028 | −0.4753 | −0.0083 | N | −2.0657 | −0.4209 | −0.0813 |
| N | −1.0165 | −2.7747 | 2.26887 | N | −1.3488 | −2.6365 | 2.39672 |
| C | −1.8362 | 3.23103 | −0.6506 | C | −1.6714 | 3.31678 | −0.6583 |
| C | 1.11985 | 0.68467 | −0.1875 | C | 1.1429 | 0.64168 | −0.2224 |
| C | 2.49721 | −1.4331 | −0.3405 | C | 2.45733 | −1.5195 | −0.2113 |
| C | 3.53606 | 0.76581 | −0.9444 | C | 3.54347 | 0.58679 | −1.0201 |
| O | −2.6401 | 2.39573 | −1.0605 | O | −2.565 | 2.56369 | −1.0439 |
| C | 2.9893 | 0.25719 | 1.44789 | C | 3.02741 | 0.30183 | 1.4166 |
| O | −2.0009 | 3.69536 | 0.94368 | O | −1.7641 | 3.79057 | 1.00861 |
| C | 1.11027 | −0.821 | −0.5158 | C | 1.08427 | −0.8857 | −0.421 |
| C | 2.13563 | 1.3729 | −1.1247 | C | 2.15875 | 1.21769 | −1.2334 |
| N | −0.4061 | 2.6866 | −0.23 | N | −0.311 | 2.67789 | −0.2781 |
| C | 1.58938 | 0.86716 | 1.26974 | C | 1.64168 | 0.9338 | 1.20718 |
| C | 3.48903 | −0.7321 | −1.2747 | C | 3.44912 | −0.9323 | −1.2202 |
| C | 2.93823 | −1.2433 | 1.11508 | C | 2.93197 | −1.218 | 1.21467 |
| C | 3.98564 | 0.95527 | 0.51162 | C | 4.02461 | 0.88772 | 0.40685 |
| C | −0.3012 | 1.24536 | −0.4519 | C | −0.2646 | 1.23073 | −0.5069 |
| C | −2.2085 | −0.8912 | −1.3768 | C | −2.1303 | −0.9571 | −1.4225 |
| C | −3.2161 | −1.9996 | −1.3644 | C | −3.1953 | −2.0105 | −1.4235 |
| C | −1.7768 | −2.2971 | −1.6921 | C | −1.7506 | −2.4047 | −1.5677 |
| C | −3.7119 | −2.1741 | 0.06016 | C | −3.8337 | −2.0353 | −0.047 |
| C | −1.3275 | 0.58253 | 0.46873 | C | −1.3221 | 0.60173 | 0.40038 |

**Table S24.** *Cont.*

| In gas phase | | | | In water | | | |
|---|---|---|---|---|---|---|---|
| C | −2.7609 | −1.3165 | 0.93436 | C | −2.9057 | −1.1764 | 0.84808 |
| C | −1.7957 | −2.1377 | 1.70026 | C | −2.0414 | −1.9962 | 1.72826 |
| H | −1.6987 | 4.22712 | −1.1019 | H | −1.4897 | 4.31942 | −1.071 |
| H | −2.6037 | 3.03632 | 1.32439 | H | −2.3766 | 3.14345 | 1.3941 |
| H | −0.8888 | 3.12097 | 0.84448 | H | −0.692 | 3.11985 | 0.8096 |
| H | 0.35375 | 3.2048 | −0.6627 | H | 0.47053 | 3.16553 | −0.7115 |
| H | 4.23489 | 1.27241 | −1.6196 | H | 4.24429 | 1.01117 | −1.7467 |
| H | 3.30448 | 0.38996 | 2.48848 | H | 3.36495 | 0.51501 | 2.43655 |
| H | 0.42408 | −1.3588 | 0.14898 | H | 0.38947 | −1.3418 | 0.29588 |
| H | 0.77442 | −0.9906 | −1.548 | H | 0.72603 | −1.1276 | −1.4304 |
| H | 1.80818 | 1.26904 | −2.1684 | H | 1.80854 | 1.02769 | −2.2563 |
| H | 2.20005 | 2.44682 | −0.9021 | H | 2.25451 | 2.30443 | −1.1131 |
| H | 1.6074 | 1.93506 | 1.52323 | H | 1.70061 | 2.01745 | 1.3684 |
| H | 0.88089 | 0.38992 | 1.95371 | H | 0.92957 | 0.53448 | 1.93704 |
| H | 3.17619 | −0.8981 | −2.3126 | H | 3.1092 | −1.1724 | −2.2349 |
| H | 4.48546 | −1.1793 | −1.1543 | H | 4.43399 | −1.3968 | −1.0784 |
| H | 3.92973 | −1.6963 | 1.25959 | H | 3.91124 | −1.6872 | 1.38055 |
| H | 2.2312 | −1.7673 | 1.76925 | H | 2.22311 | −1.6624 | 1.92432 |
| H | 4.99073 | 0.53777 | 0.64897 | H | 5.01913 | 0.45355 | 0.5653 |
| H | 4.04197 | 2.02421 | 0.7523 | H | 4.11282 | 1.97128 | 0.55221 |
| H | −2.2414 | −0.0871 | −2.1017 | H | −2.0237 | −0.2403 | −2.2259 |
| H | −0.5566 | 1.03997 | −1.4972 | H | −0.5083 | 1.03841 | −1.5562 |
| H | −3.9463 | −2.065 | −2.1622 | H | −3.8457 | −2.1126 | −2.2835 |
| H | −1.159 | −2.8083 | −0.9581 | H | −1.2452 | −2.8791 | −0.7305 |
| H | −1.5468 | −2.5366 | −2.7243 | H | −1.424 | −2.7377 | −2.5462 |
| H | −3.7256 | −3.2142 | 0.393 | H | −3.96 | −3.0405 | 0.35963 |
| H | −4.7249 | −1.7746 | 0.14291 | H | −4.8144 | −1.5586 | −0.0912 |
| H | −3.2929 | −0.6863 | 1.65198 | H | −3.459 | −0.4893 | 1.49266 |
| H | 3.19031 | −3.2438 | −0.4633 | H | 3.13053 | −3.3431 | −0.2582 |

**Table S25.** Coordinates of TS_2.

| In gas phase | | | | In water | | | |
|---|---|---|---|---|---|---|---|
| **O** | **2.341488** | **−2.79083** | **−0.70715** | **O** | **2.299019** | −2.88689 | −0.50266 |
| O | −1.38681 | 0.787124 | 1.749763 | O | −1.43712 | 0.875869 | 1.648531 |
| C | −1.75331 | 3.437286 | −0.4714 | C | −1.63812 | 3.463552 | −0.47251 |
| N | −2.07886 | −0.47475 | 0.02389 | N | −2.06951 | −0.44391 | −0.05644 |
| N | −0.9665 | −2.91099 | 2.12009 | N | −1.31724 | −2.73946 | 2.339643 |
| O | −1.99763 | 3.984739 | 0.701775 | O | −1.79751 | 4.020432 | 0.733374 |
| N | −0.43563 | 2.672672 | −0.03994 | N | −0.3426 | 2.671279 | −0.12547 |
| C | 1.111196 | 0.686219 | −0.14571 | C | 1.139185 | 0.6509 | −0.18173 |
| C | 2.489682 | −1.4242 | −0.3651 | C | 2.471407 | −1.4977 | −0.25935 |
| C | 3.498552 | 0.784102 | −0.98448 | C | 3.526761 | 0.642279 | −1.01729 |
| C | 3.037376 | 0.251567 | 1.420441 | C | 3.052386 | 0.275708 | 1.416657 |
| C | 1.095441 | −0.81517 | −0.49017 | C | 1.090009 | −0.86962 | −0.42751 |
| C | 2.089602 | 1.3856 | −1.11372 | C | 2.133308 | 1.266362 | −1.19031 |
| C | 1.629869 | 0.856455 | 1.296362 | C | 1.658171 | 0.901497 | 1.249001 |
| O | −2.68235 | 2.482008 | −0.9062 | O | −2.64275 | 2.573319 | −0.86036 |
| C | 3.44708 | −0.71128 | −1.32579 | C | 3.442319 | −0.87045 | −1.26425 |
| C | 2.979223 | −1.24609 | 1.07624 | C | 2.966643 | −1.23805 | 1.167753 |
| C | 3.997407 | 0.962873 | 0.456715 | C | 4.028046 | 0.902632 | 0.410763 |
| C | −0.32121 | 1.244968 | −0.34681 | C | −0.27875 | 1.236254 | −0.42191 |
| C | −2.21017 | −0.88609 | −1.35336 | C | −2.11299 | −0.95434 | −1.40985 |
| C | −3.20354 | −2.00849 | −1.37882 | C | −3.16775 | −2.01766 | −1.44442 |
| C | −1.75315 | −2.28325 | −1.67412 | C | −1.71813 | −2.39505 | −1.58238 |
| C | −3.72455 | −2.20358 | 0.034093 | C | −3.81668 | −2.0795 | −0.07415 |
| C | −1.31523 | 0.518373 | 0.560842 | C | −1.31404 | 0.551735 | 0.470421 |
| C | −2.76498 | −1.38885 | 0.935791 | C | −2.89646 | −1.24336 | 0.848735 |
| C | −1.76691 | −2.24472 | 1.618384 | C | −2.01939 | −2.08423 | 1.695977 |
| H | −1.51323 | 4.078534 | −1.33568 | H | −1.43458 | 4.119054 | −1.33133 |
| H | −0.94361 | 3.132549 | 0.956338 | H | −0.77037 | 3.168747 | 0.910607 |
| H | 0.392568 | 3.198128 | −0.31397 | H | 0.477873 | 3.166266 | −0.47297 |
| H | −3.2543 | 2.372306 | −0.13116 | H | −3.13347 | 2.393813 | −0.0437 |
| H | 4.171415 | 1.29981 | −1.67873 | H | 4.212199 | 1.095751 | −1.74099 |
| H | 3.387349 | 0.376286 | 2.450711 | H | 3.403926 | 0.459475 | 2.437558 |
| H | 0.431563 | −1.36433 | 0.18854 | H | 0.409588 | −1.3547 | 0.284656 |

**Table S25.** *Cont.*

| In gas phase | | | | In water | | | |
|---|---|---|---|---|---|---|---|
| H | 0.730757 | −0.97295 | −1.51426 | H | 0.720185 | −1.08316 | −1.43887 |
| H | 1.727062 | 1.282669 | −2.14565 | H | 1.76894 | 1.103039 | −2.21284 |
| H | 2.156607 | 2.460257 | −0.8955 | H | 2.221671 | 2.350067 | −1.04127 |
| H | 1.654748 | 1.921387 | 1.56249 | H | 1.710549 | 1.979682 | 1.445846 |
| H | 0.945629 | 0.370654 | 1.998233 | H | 0.960459 | 0.472503 | 1.97594 |
| H | 3.100826 | −0.87065 | −2.35405 | H | 3.088529 | −1.0812 | −2.28074 |
| H | 4.448778 | −1.15501 | −1.24183 | H | 4.433032 | −1.33081 | −1.1525 |
| H | 3.97599 | −1.69786 | 1.182957 | H | 3.952253 | −1.70343 | 1.303476 |
| H | 2.296082 | −1.77804 | 1.749133 | H | 2.273065 | −1.71095 | 1.874153 |
| H | 5.008664 | 0.549627 | 0.555487 | H | 5.028714 | 0.473029 | 0.539925 |
| H | 4.056679 | 2.029782 | 0.705313 | H | 4.10897 | 1.981787 | 0.589027 |
| H | −2.22769 | −0.0836 | −2.08045 | H | −2.00299 | −0.2234 | −2.20001 |
| H | −0.59313 | 1.103855 | −1.39839 | H | −0.52941 | 1.091337 | −1.47739 |
| H | −3.91549 | −2.07718 | −2.19276 | H | −3.80945 | −2.10711 | −2.3123 |
| H | −1.14626 | −2.79911 | −0.93469 | H | −1.21664 | −2.88418 | −0.75143 |
| H | −1.49541 | −2.50269 | −2.70426 | H | −1.37806 | −2.70174 | −2.56486 |
| H | −3.76865 | −3.24955 | 0.344853 | H | −3.94466 | −3.09498 | 0.305486 |
| H | −4.72836 | −1.77959 | 0.112377 | H | −4.79745 | −1.60235 | −0.11308 |
| H | −3.28264 | −0.81787 | 1.710578 | H | −3.45537 | −0.58722 | 1.519922 |
| H | 3.178888 | −3.23457 | −0.52582 | H | 3.15937 | −3.31223 | −0.38942 |

**Table S26.** Coordinates of Intermediate_1.

| | | | In gas phase | | | In water | |
|---|---|---|---|---|---|---|---|
| **O** | **2.286211** | **−2.87873** | **−0.7626** | **O** | **2.268981** | −2.93115 | −0.60456 |
| O | −1.59479 | 0.89553 | 1.6155 | O | −1.507 | 0.808646 | 1.678319 |
| N | −0.26147 | 2.664549 | −0.02258 | N | −0.25596 | 2.650044 | 0.017692 |
| N | −2.06388 | −0.50526 | −0.07182 | N | −2.08533 | −0.47692 | −0.06831 |
| N | −1.36892 | −2.84031 | 2.303914 | N | −1.44519 | −2.82762 | 2.303134 |
| C | 1.094028 | 0.601841 | −0.14522 | C | 1.114473 | 0.592717 | −0.13878 |
| C | 2.441808 | −1.52506 | −0.36715 | C | 2.440007 | −1.55234 | −0.30124 |
| C | 3.517273 | 0.685287 | −0.8579 | C | 3.502342 | 0.617611 | −0.9674 |
| C | 2.932409 | 0.072028 | 1.501449 | C | 3.02813 | 0.145014 | 1.447457 |
| C | 1.064183 | −0.88686 | −0.53157 | C | 1.058688 | −0.91557 | −0.44255 |
| C | 2.12875 | 1.316487 | −1.03561 | C | 2.110336 | 1.248225 | −1.11492 |
| C | 1.545822 | 0.71051 | 1.325997 | C | 1.637056 | 0.781288 | 1.300219 |
| C | 3.457215 | −0.79718 | −1.25417 | C | 3.414564 | −0.88438 | −1.27667 |
| C | 2.867392 | −1.41012 | 1.100451 | C | 2.937886 | −1.35643 | 1.134636 |
| C | 3.949066 | 0.80094 | 0.611532 | C | 4.005584 | 0.813267 | 0.470215 |
| C | −0.29823 | 1.254934 | −0.36972 | C | −0.2836 | 1.239155 | −0.34014 |
| C | −2.04626 | −1.04599 | −1.41048 | C | −2.0858 | −0.97658 | −1.42643 |
| C | −1.36143 | 3.422866 | −0.54454 | C | −1.36384 | 3.395444 | −0.51325 |
| C | −3.11907 | −2.0927 | −1.47871 | C | −3.1658 | −2.01182 | −1.51559 |
| C | −1.67017 | −2.49432 | −1.54688 | C | −1.72176 | −2.42478 | −1.59998 |
| O | −1.26234 | 4.680957 | 0.048537 | O | −1.26302 | 4.667361 | 0.05167 |
| C | −3.82018 | −2.12231 | −0.13253 | C | −3.86391 | −2.07551 | −0.16962 |
| C | −1.37454 | 0.538861 | 0.459533 | C | −1.3449 | 0.509837 | 0.49328 |
| O | −2.63621 | 2.866589 | −0.3144 | O | −2.63313 | 2.837485 | −0.26127 |
| C | −2.90874 | −1.30423 | 0.814891 | C | −2.95421 | −1.27684 | 0.79576 |
| C | −2.05503 | −2.16378 | 1.665526 | C | −2.11793 | −2.14954 | 1.651717 |
| H | −0.26158 | 2.764848 | 0.991996 | H | −0.2762 | 2.73379 | 1.034443 |
| H | −1.28193 | 3.470086 | −1.63991 | H | −1.29174 | 3.417761 | −1.60844 |
| H | −2.08236 | 5.144746 | −0.15655 | H | −2.05135 | 5.157205 | −0.21502 |
| H | −2.68201 | 2.652861 | 0.631073 | H | −2.70654 | 2.708868 | 0.697544 |
| H | 4.236285 | 1.209335 | −1.49777 | H | 4.19262 | 1.096871 | −1.67042 |
| H | 3.232948 | 0.1538 | 2.551824 | H | 3.378801 | 0.284338 | 2.475978 |
| H | 0.365054 | −1.44587 | 0.101945 | H | 0.376667 | −1.43017 | 0.247925 |

**Table S26.** *Cont.*

| | | In gas phase | | | | In water | |
|---|---|---|---|---|---|---|---|
| H | 0.743434 | −1.0125 | −1.57457 | H | 0.690675 | −1.08898 | −1.46225 |
| H | 1.816612 | 1.241047 | −2.08711 | H | 1.748507 | 1.118624 | −2.1442 |
| H | 2.149738 | 2.379664 | −0.77837 | H | 2.153766 | 2.323344 | −0.91375 |
| H | 1.594351 | 1.766696 | 1.619526 | H | 1.702559 | 1.852142 | 1.529684 |
| H | 0.818244 | 0.219658 | 1.982609 | H | 0.937362 | 0.330717 | 2.013688 |
| H | 3.158052 | −0.91292 | −2.30303 | H | 3.06191 | −1.05154 | −2.30184 |
| H | 4.44491 | −1.26582 | −1.13847 | H | 4.403184 | −1.35439 | −1.18294 |
| H | 3.850936 | −1.8831 | 1.235382 | H | 3.922129 | −1.83081 | 1.248764 |
| H | 2.146251 | −1.9545 | 1.722074 | H | 2.243816 | −1.85658 | 1.821805 |
| H | 4.947596 | 0.366006 | 0.745428 | H | 5.005859 | 0.376986 | 0.581641 |
| H | 4.010864 | 1.856813 | 0.901543 | H | 4.086947 | 1.883463 | 0.696083 |
| H | −1.87842 | −0.33332 | −2.20775 | H | −1.91587 | −0.24639 | −2.2067 |
| H | −0.53191 | 1.178232 | −1.43931 | H | −0.52788 | 1.168883 | −1.40631 |
| H | −3.72838 | −2.18727 | −2.36983 | H | −3.777 | −2.07653 | −2.40739 |
| H | −1.21061 | −2.98573 | −0.69406 | H | −1.26682 | −2.93742 | −0.75668 |
| H | −1.29032 | −2.81754 | −2.50953 | H | −1.34895 | −2.72682 | −2.57207 |
| H | −3.98825 | −3.13059 | 0.251749 | H | −4.03116 | −3.0928 | 0.189624 |
| H | −4.78643 | −1.61886 | −0.21388 | H | −4.83007 | −1.57185 | −0.23407 |
| H | −3.46499 | −0.64 | 1.480373 | H | −3.51861 | −0.62147 | 1.462848 |
| H | 3.122846 | −3.33055 | −0.5978 | H | 3.132191 | −3.35719 | −0.51915 |

**Table S27.** Coordinates of TS_3.

| | In gas phase | | | | In water | | |
|---|---|---|---|---|---|---|---|
| **O** | **2.443526** | **−2.7508** | **−0.78418** | **O** | **2.440836** | −2.81222 | −0.69318 |
| O | −1.62625 | 0.836377 | 1.578884 | O | −1.60437 | 0.839518 | 1.566172 |
| N | −0.41052 | 2.639616 | −0.07263 | N | −0.37755 | 2.621461 | −0.1146 |
| N | −2.02217 | −0.59944 | −0.10091 | N | −2.03988 | −0.57805 | −0.12105 |
| N | −1.23238 | −2.84665 | 2.320205 | N | −1.3781 | −2.77308 | 2.380398 |
| C | 1.089594 | 0.662414 | −0.14278 | C | 1.106929 | 0.633798 | −0.15211 |
| C | 2.534326 | −1.39805 | −0.36939 | C | 2.535147 | −1.44285 | −0.32542 |
| C | 3.515622 | 0.865777 | −0.81433 | C | 3.531043 | 0.79823 | −0.84148 |
| C | 2.920486 | 0.195098 | 1.527425 | C | 2.94513 | 0.202026 | 1.521078 |
| C | 1.133186 | −0.8196 | −0.54729 | C | 1.135903 | −0.86071 | −0.51538 |
| C | 2.102884 | 1.43835 | −1.0062 | C | 2.122325 | 1.376497 | −1.04245 |
| C | 1.507636 | 0.771861 | 1.338713 | C | 1.535008 | 0.782556 | 1.323185 |
| C | 3.52783 | −0.61212 | −1.23156 | C | 3.52887 | −0.69115 | −1.21644 |
| C | 2.929422 | −1.28301 | 1.106599 | C | 2.942448 | −1.28693 | 1.143313 |
| C | 3.917104 | 0.980963 | 0.663595 | C | 3.942626 | 0.954646 | 0.629535 |
| C | −0.33311 | 1.229286 | −0.39761 | C | −0.31259 | 1.203863 | −0.42135 |
| C | −1.94323 | −1.17315 | −1.42525 | C | −1.95841 | −1.16147 | −1.44445 |
| C | −1.42324 | 3.378727 | −0.62685 | C | −1.39002 | 3.367411 | −0.64268 |
| C | −2.95722 | −2.27693 | −1.49015 | C | −2.99745 | −2.23957 | −1.51645 |
| C | −1.48842 | −2.60295 | −1.51466 | C | −1.5384 | −2.60332 | −1.51126 |
| O | −1.64691 | 4.581477 | −0.16886 | O | −1.53561 | 4.607921 | −0.21837 |
| C | −3.68785 | −2.30967 | −0.1596 | C | −3.74999 | −2.25132 | −0.19839 |
| C | −1.38387 | 0.474446 | 0.429507 | C | −1.37427 | 0.471699 | 0.411063 |
| O | −3.03935 | 2.870763 | −0.04189 | O | −2.97973 | 2.967988 | 0.063723 |
| C | −2.83979 | −1.42725 | 0.788202 | C | −2.90797 | −1.36975 | 0.755515 |
| C | −1.95168 | −2.22178 | 1.665929 | C | −2.06257 | −2.15967 | 1.679131 |
| H | −0.28974 | 2.846418 | 0.914644 | H | −0.183 | 2.8574 | 0.85397 |
| H | −1.59851 | 3.137161 | −1.68335 | H | −1.63213 | 3.100499 | −1.677 |
| H | −2.69992 | 4.049556 | 0.121973 | H | −2.56811 | 4.12593 | 0.190729 |
| H | −2.90324 | 2.392352 | 0.792522 | H | −2.81388 | 2.462896 | 0.877917 |
| H | 4.219174 | 1.431298 | −1.43527 | H | 4.234921 | 1.338377 | −1.48382 |
| H | 3.199178 | 0.276958 | 2.583534 | H | 3.230126 | 0.313133 | 2.572771 |
| H | 0.449775 | −1.4148 | 0.069503 | H | 0.4456 | −1.42944 | 0.120649 |

**Table S27.** *Cont.*

| In gas phase | | | | In water | | | |
|---|---|---|---|---|---|---|---|
| H | 0.833769 | −0.94609 | −1.59638 | H | 0.828328 | −1.01034 | −1.55854 |
| H | 1.810271 | 1.36838 | −2.06333 | H | 1.822228 | 1.282021 | −2.09475 |
| H | 2.080015 | 2.498736 | −0.73405 | H | 2.114155 | 2.443242 | −0.79432 |
| H | 1.503018 | 1.823664 | 1.653546 | H | 1.541513 | 1.841634 | 1.611719 |
| H | 0.793752 | 0.236849 | 1.975056 | H | 0.82138 | 0.267886 | 1.976638 |
| H | 3.249098 | −0.72638 | −2.2861 | H | 3.240788 | −0.8285 | −2.26578 |
| H | 4.533994 | −1.0365 | −1.10787 | H | 4.531251 | −1.12068 | −1.08631 |
| H | 3.931081 | −1.71174 | 1.253414 | H | 3.94036 | −1.71982 | 1.294905 |
| H | 2.223971 | −1.86772 | 1.709176 | H | 2.236166 | −1.84706 | 1.768834 |
| H | 4.931146 | 0.588073 | 0.808319 | H | 4.953523 | 0.556909 | 0.7804 |
| H | 3.928733 | 2.034478 | 0.968154 | H | 3.962622 | 2.016565 | 0.902754 |
| H | −1.79292 | −0.47417 | −2.23807 | H | −1.77415 | −0.47565 | −2.26074 |
| H | −0.54251 | 1.129904 | −1.46951 | H | −0.51716 | 1.091858 | −1.49162 |
| H | −3.53899 | −2.42653 | −2.39199 | H | −3.56599 | −2.38023 | −2.42744 |
| H | −1.02467 | −3.05042 | −0.64026 | H | −1.1099 | −3.05325 | −0.62003 |
| H | −1.06783 | −2.92696 | −2.46003 | H | −1.10799 | −2.94469 | −2.44579 |
| H | −3.8159 | −3.3158 | 0.244971 | H | −3.89808 | −3.25195 | 0.212097 |
| H | −4.67442 | −1.85515 | −0.27478 | H | −4.72812 | −1.78558 | −0.32944 |
| H | −3.44386 | −0.78287 | 1.430701 | H | −3.52028 | −0.70454 | 1.367851 |
| H | 3.2947 | −3.17039 | −0.6088 | H | 3.31296 | −3.20861 | −0.56548 |

**Table S28.** Coordinates of HCOOH.

| In gas phase | | | | In water | | | | In formic acid | | | |
|---|---|---|---|---|---|---|---|---|---|---|---|
| **C** | **0.129858** | **0.360338** | **0** | **C** | **0.12326** | **0.367486** | **−0.000001** | **C** | 0.123121 | 0.367493 | 0 |
| H | 0.039598 | 1.462359 | 0.000001 | H | 0.035469 | 1.464785 | 0.000001 | H | 0.036408 | 1.464629 | 0.000001 |
| O | 1.172492 | −0.218756 | −0.000005 | O | 1.17076 | −0.220222 | −0.000006 | O | 1.170678 | −0.220278 | −0.000005 |
| O | −1.052962 | −0.279368 | 0.000005 | O | −1.045038 | −0.28181 | 0.000005 | O | −1.044977 | −0.281736 | 0.000005 |
| H | −1.774981 | 0.360601 | 0.000009 | H | −1.780803 | 0.346551 | 0.000009 | H | −1.780743 | 0.346527 | 0.000009 |

**Table S29.** Coordinates of $H_2O$.

| In gas phase | | | | In water | | | | In formic acid | | | |
|---|---|---|---|---|---|---|---|---|---|---|---|
| **O** | **0** | **0** | **0.117793** | **O** | **0** | **0** | **0.119033** | **O** | 0 | 0 | 0.119023 |
| H | 0 | 0.76021 | −0.471173 | H | 0 | 0.757444 | −0.476131 | H | 0 | 0.757468 | −0.476091 |
| H | 0 | −0.76021 | −0.471173 | H | 0 | −0.757444 | −0.476131 | H | 0 | −0.757468 | −0.476091 |

**Table S30.** Coordinates of $HCOO^-$.

| In gas phase | | | | In water | | | |
|---|---|---|---|---|---|---|---|
| **C** | **0** | **0.31016** | **0** | **C** | **0** | 0.323748 | 0 |
| H | 0.000649 | 1.456865 | 0 | H | 0.000305 | 1.454616 | 0 |
| O | 1.135822 | −0.207748 | 0 | O | 1.12966 | −0.212518 | 0 |
| O | −1.135904 | −0.20698 | 0 | O | −1.129698 | −0.21212 | 0 |

**Table S31.** Coordinates of $HCOOH^+$.

| In gas phase | | | | In water | | | |
|---|---|---|---|---|---|---|---|
| **C** | **0.00007** | **0.396905** | **−0.000076** | **C** | **0.000003** | 0.395295 | 0.000283 |
| H | 0.000034 | 1.490371 | 0.000088 | H | −0.000001 | 1.484885 | −0.000042 |
| O | 1.07207 | −0.273526 | 0.000005 | O | 1.072924 | −0.275841 | −0.000066 |
| H | 1.893671 | 0.252167 | 0.000145 | H | 1.87526 | 0.278396 | −0.000295 |
| O | −1.072119 | −0.273495 | 0.000006 | O | −1.072926 | −0.275841 | −0.000067 |
| H | −1.893727 | 0.25219 | 0.000141 | H | −1.875257 | 0.278403 | −0.000298 |

**Table S32.** Coordinates of Intermediate_2.

| In gas phase | | | | In water | | | | In formic acid | | | |
|---|---|---|---|---|---|---|---|---|---|---|---|
| **O** | **2.469636** | **−2.661312** | **−0.793752** | **O** | **2.460693** | **−2.761573** | **−0.719978** | **O** | 2.461147 | −2.76046 | −0.72231 |
| O | −1.698173 | 1.017351 | 1.433986 | O | −1.57868 | 1.010494 | 1.497341 | O | −1.58022 | 1.011394 | 1.496116 |
| N | −1.991846 | −0.620071 | −0.091125 | N | −1.987263 | −0.541876 | −0.086566 | N | −1.98715 | −0.54234 | −0.08687 |
| N | −0.896217 | −2.532594 | 2.419533 | N | −1.314795 | −2.640639 | 2.483095 | N | −1.31374 | −2.64012 | 2.48346 |
| C | −1.872596 | 3.356466 | −0.404485 | C | −1.776429 | 3.350088 | −0.407737 | C | −1.77667 | 3.35042 | −0.4083 |
| C | 1.076469 | 0.728245 | −0.193817 | C | 1.142353 | 0.686824 | −0.18004 | C | 1.141951 | 0.687072 | −0.17992 |
| C | 2.557433 | −1.315291 | −0.376174 | C | 2.563855 | −1.397842 | −0.342416 | C | 2.563963 | −1.39714 | −0.34335 |
| C | 3.519879 | 0.958439 | −0.798118 | C | 3.579781 | 0.838215 | −0.827103 | C | 3.579822 | 0.839556 | −0.82526 |
| C | 2.860623 | 0.286353 | 1.528162 | C | 2.944136 | 0.234407 | 1.521346 | C | 2.942694 | 0.233413 | 1.522213 |

**Table S32.** *Cont.*

| In gas phase | | | | In water | | | | In formic acid | | | |
|---|---|---|---|---|---|---|---|---|---|---|---|
| O | −2.496808 | 3.567484 | 0.775826 | O | −2.352418 | 3.601351 | 0.798162 | O | −2.35309 | 3.601688 | 0.797344 |
| C | 1.15427 | −0.752807 | −0.59467 | C | 1.170932 | −0.807279 | −0.555708 | C | 1.17109 | −0.80664 | −0.55695 |
| C | 2.104644 | 1.518749 | −1.033065 | C | 2.178492 | 1.430177 | −1.051378 | C | 2.178574 | 1.431484 | −1.04983 |
| N | −0.503286 | 2.670363 | −0.154454 | N | −0.394829 | 2.680471 | −0.18323 | N | −0.3953 | 2.680605 | −0.18348 |
| C | 1.441555 | 0.84289 | 1.302493 | C | 1.538482 | 0.822908 | 1.305825 | C | 1.537024 | 0.821793 | 1.306355 |
| C | 3.560643 | −0.518223 | −1.215816 | C | 3.575838 | −0.647449 | −1.212539 | C | 3.576361 | −0.64574 | −1.21213 |
| C | 2.901618 | −1.192422 | 1.11205 | C | 2.940939 | −1.252165 | 1.135323 | C | 2.940029 | −1.25279 | 1.134785 |
| C | 3.869517 | 1.085905 | 0.691688 | C | 3.961262 | 0.986993 | 0.652546 | C | 3.960267 | 0.986997 | 0.654786 |
| C | −0.365934 | 1.212981 | −0.514434 | C | −0.286497 | 1.212963 | −0.489302 | C | −0.28685 | 1.213098 | −0.48971 |
| C | −1.900586 | −1.278234 | −1.380501 | C | −1.937227 | −1.152892 | −1.399578 | C | −1.93602 | −1.15444 | −1.39934 |
| C | −2.841418 | −2.449136 | −1.340464 | C | −2.983576 | −2.226154 | −1.422055 | C | −2.98198 | −2.22814 | −1.42159 |
| C | −1.359977 | −2.681911 | −1.389699 | C | −1.52768 | −2.59847 | −1.435317 | C | −1.52598 | −2.59992 | −1.4338 |
| C | −3.526212 | −2.454713 | 0.015492 | C | −3.714491 | −2.200215 | −0.092125 | C | −3.71353 | −2.20145 | −0.09198 |
| C | −1.427919 | 0.514425 | 0.3358 | C | −1.353691 | 0.544092 | 0.374222 | C | −1.35434 | 0.544213 | 0.373496 |
| C | −2.733662 | −1.4462 | 0.880327 | C | −2.858453 | −1.294246 | 0.826165 | C | −2.85795 | −1.29489 | 0.826125 |
| C | −1.736683 | −2.074843 | 1.771893 | C | −2.007198 | −2.053937 | 1.767367 | C | −2.00635 | −2.05398 | 1.767495 |
| H | −1.648358 | 4.334128 | −0.84133 | H | −1.574376 | 4.308514 | −0.88838 | H | −1.57438 | 4.308916 | −0.88877 |
| H | −2.647412 | 2.708509 | 1.217345 | H | −2.540652 | 2.751191 | 1.236842 | H | −2.54065 | 2.751533 | 1.236389 |
| H | −0.353207 | 2.735845 | 0.862372 | H | −0.179015 | 2.826176 | 0.811113 | H | −0.17986 | 2.825985 | 0.811016 |
| H | 0.242374 | 3.190632 | −0.62387 | H | 0.310373 | 3.18449 | −0.728845 | H | 0.310205 | 3.184582 | −0.72873 |
| H | 4.228193 | 1.535966 | −1.400226 | H | 4.294008 | 1.381568 | −1.45367 | H | 4.29436 | 1.383636 | −1.45084 |
| H | 3.102521 | 0.377026 | 2.591311 | H | 3.207705 | 0.339836 | 2.578456 | H | 3.205531 | 0.3379 | 2.579591 |
| H | 0.471132 | −1.348177 | 0.017927 | H | 0.470078 | −1.37398 | 0.068185 | H | 0.470013 | −1.37402 | 0.066006 |
| H | 0.878747 | −0.892575 | −1.648163 | H | 0.879543 | −0.950394 | −1.604097 | H | 0.880323 | −0.94888 | −1.60564 |
| H | 1.844806 | 1.460097 | −2.098862 | H | 1.897094 | 1.355239 | −2.109389 | H | 1.897884 | 1.357649 | −2.10813 |
| H | 2.121039 | 2.582558 | −0.748036 | H | 2.218867 | 2.496132 | −0.7907 | H | 2.218688 | 2.497183 | −0.78793 |
| H | 1.422572 | 1.891868 | 1.635634 | H | 1.548591 | 1.876883 | 1.614976 | H | 1.546593 | 1.875485 | 1.61657 |
| H | 0.720188 | 0.290723 | 1.915579 | H | 0.813866 | 0.302395 | 1.941503 | H | 0.812139 | 0.30047 | 1.941053 |
| H | 3.312776 | −0.637504 | −2.277066 | H | 3.305836 | −0.777264 | −2.267277 | H | 3.30707 | −0.77463 | −2.26716 |
| H | 4.570069 | −0.921624 | −1.066889 | H | 4.574049 | −1.080132 | −1.067186 | H | 4.574578 | −1.07834 | −1.0666 |
| H | 3.903063 | −1.603217 | 1.295015 | H | 3.933617 | −1.689037 | 1.304966 | H | 3.932695 | −1.6896 | 1.304668 |
| H | 2.186539 | −1.7859 | 1.693987 | H | 2.219882 | −1.812807 | 1.742786 | H | 2.218718 | −1.81419 | 1.741216 |
| H | 4.882287 | 0.707359 | 0.86741 | H | 4.965992 | 0.582167 | 0.820224 | H | 4.964943 | 0.582186 | 0.822739 |

**Table S32.** *Cont.*

| In gas phase | | | | In water | | | | In formic acid | | | |
|---|---|---|---|---|---|---|---|---|---|---|---|
| H | 3.864168 | 2.140467 | 0.994251 | H | 3.983523 | 2.047316 | 0.930971 | H | 3.982221 | 2.047063 | 0.934223 |
| H | −1.820162 | −0.618989 | −2.236663 | H | −1.770641 | −0.481535 | −2.231958 | H | −1.76906 | −0.48372 | −2.23217 |
| H | −0.560121 | 1.130423 | −1.585297 | H | −0.490498 | 1.10903 | −1.555107 | H | −0.49039 | 1.109284 | −1.55565 |
| H | −3.439003 | −2.692606 | −2.210649 | H | −3.569039 | −2.383333 | −2.319358 | H | −3.56712 | −2.38639 | −2.31892 |
| H | −0.848166 | −3.04882 | −0.504542 | H | −1.085162 | −3.026472 | −0.539902 | H | −1.08356 | −3.0274 | −0.5381 |
| H | −0.940079 | −3.033557 | −2.325368 | H | −1.115894 | −2.965794 | −2.368116 | H | −1.11365 | −2.96752 | −2.36625 |
| H | −3.549568 | −3.440825 | 0.483435 | H | −3.854082 | −3.189262 | 0.347748 | H | −3.85318 | −3.19027 | 0.348401 |
| H | −4.553345 | −2.099585 | −0.088857 | H | −4.694639 | −1.738471 | −0.219871 | H | −4.69371 | −1.73994 | −0.22045 |
| H | −3.376157 | −0.808425 | 1.490549 | H | −3.460946 | −0.600138 | 1.415788 | H | −3.46068 | −0.60087 | 1.415597 |
| H | 3.308923 | −3.099231 | −0.604489 | H | 3.323212 | −3.17232 | −0.57354 | H | 3.323464 | −3.17147 | −0.57548 |
| O | −2.510438 | 2.503419 | −1.282006 | O | −2.449387 | 2.459102 | −1.212158 | O | −2.44935 | 2.459591 | −1.21319 |
| H | −3.388092 | 2.861964 | −1.474598 | H | −3.279722 | 2.863213 | −1.501163 | H | −3.28047 | 2.863121 | −1.50078 |

**Table S33.** Coordinates of TS_4.

| In gas phase | | | | In water | | | | In formic acid | | | |
|---|---|---|---|---|---|---|---|---|---|---|---|
| **O** | **2.344187** | **−2.890787** | **−0.529863** | **O** | **2.311411** | **−2.968168** | **−0.364081** | **O** | 2.312971 | −2.96734 | −0.36769 |
| O | −1.807959 | 1.253069 | 1.095345 | O | −1.696562 | 1.178365 | 1.245931 | O | −1.69822 | 1.179402 | 1.243825 |
| N | −2.022422 | −0.569183 | −0.207146 | N | −2.014441 | −0.531016 | −0.183919 | N | −2.01413 | −0.5317 | −0.18438 |
| N | −1.216076 | −2.238859 | 2.579506 | N | −1.637996 | −2.401554 | 2.62163 | N | −1.63868 | −2.39891 | 2.623598 |
| C | −1.504696 | 3.499413 | −0.548331 | C | −1.437079 | 3.479458 | −0.604342 | C | −1.43752 | 3.47943 | −0.60509 |
| C | 1.065502 | 0.592275 | −0.338269 | C | 1.127515 | 0.571961 | −0.290243 | C | 1.127329 | 0.572019 | −0.29028 |
| C | 2.46479 | −1.515116 | −0.231495 | C | 2.465173 | −1.571682 | −0.158603 | C | 2.465909 | −1.57116 | −0.16004 |
| C | 3.552757 | 0.669748 | −0.785879 | C | 3.581003 | 0.540661 | −0.898147 | C | 3.581548 | 0.542751 | −0.89541 |
| C | 2.710681 | 0.243098 | 1.53753 | C | 2.879185 | 0.264852 | 1.494854 | C | 2.877131 | 0.2634 | 1.496364 |
| O | −1.227061 | 3.900948 | 0.876439 | O | −1.25652 | 4.091599 | 0.745658 | O | −1.25688 | 4.091181 | 0.745204 |
| C | 1.104462 | −0.920637 | −0.598879 | C | 1.10294 | −0.956409 | −0.479905 | C | 1.103715 | −0.95609 | −0.48196 |
| C | 2.184512 | 1.261324 | −1.166749 | C | 2.210492 | 1.153469 | −1.226346 | C | 2.211103 | 1.155333 | −1.22432 |
| N | −0.298427 | 2.629028 | −0.622378 | N | −0.242461 | 2.619181 | −0.424134 | N | −0.24284 | 2.619083 | −0.42541 |
| C | 1.336364 | 0.827194 | 1.161503 | C | 1.504575 | 0.875366 | 1.173786 | C | 1.502579 | 0.873706 | 1.174567 |
| C | 3.557727 | −0.839711 | −1.064758 | C | 3.523656 | −0.980288 | −1.09314 | C | 3.525163 | −0.97795 | −1.09254 |
| C | 2.715564 | −1.267844 | 1.2602 | C | 2.822706 | −1.256467 | 1.297503 | C | 2.821583 | −1.2577 | 1.29691 |
| C | 3.80821 | 0.92302 | 0.707022 | C | 3.941487 | 0.86134 | 0.559629 | C | 3.940217 | 0.861659 | 0.563189 |

**Table S33.** *Cont.*

| In gas phase | | | | In water | | | | In formic acid | | | |
|---|---|---|---|---|---|---|---|---|---|---|---|
| C | −0.330492 | 1.153555 | −0.780934 | C | −0.268838 | 1.165822 | −0.670531 | C | −0.26896 | 1.165585 | −0.67139 |
| C | −1.906304 | −1.391706 | −1.398354 | C | −1.907217 | −1.284491 | −1.418633 | C | −1.90607 | −1.28625 | −1.41838 |
| C | −2.939099 | −2.478241 | −1.286927 | C | −3.012208 | −2.297935 | −1.41389 | C | −3.01066 | −2.30016 | −1.41322 |
| C | −1.481412 | −2.820258 | −1.204183 | C | −1.583849 | −2.744271 | −1.276221 | C | −1.5822 | −2.74576 | −1.27455 |
| C | −3.695098 | −2.273202 | 0.014485 | C | −3.829583 | −2.094008 | −0.152007 | C | −3.82861 | −2.09557 | −0.15182 |
| C | −1.447817 | 0.599919 | 0.0969 | C | −1.380336 | 0.588185 | 0.202609 | C | −1.38087 | 0.58817 | 0.201391 |
| C | −2.874064 | −1.22681 | 0.805273 | C | −2.982067 | −1.14906 | 0.733737 | C | −2.98206 | −1.14925 | 0.733394 |
| C | −1.976695 | −1.807832 | 1.823997 | C | −2.240259 | −1.854119 | 1.80106 | C | −2.24058 | −1.85268 | 1.801976 |
| H | −1.363728 | 4.402527 | −1.138864 | H | −1.295616 | 4.255493 | −1.350837 | H | −1.29592 | 4.255876 | −1.35115 |
| H | −1.825865 | 3.417401 | 1.480993 | H | −2.060447 | 4.133114 | 1.297258 | H | −2.06062 | 4.130705 | 1.297222 |
| H | −0.32463 | 3.185772 | 0.652989 | H | −0.4899 | 3.219677 | 0.829546 | H | −0.48987 | 3.219396 | 0.82828 |
| H | 0.379903 | 3.017919 | −1.273542 | H | 0.547211 | 3.039282 | −0.911077 | H | 0.546688 | 3.039133 | −0.91263 |
| H | 4.325195 | 1.160918 | −1.386033 | H | 4.329271 | 0.971448 | −1.571148 | H | 4.330338 | 0.974842 | −1.567 |
| H | 2.885129 | 0.421846 | 2.602862 | H | 3.131339 | 0.491935 | 2.535733 | H | 3.127958 | 0.489194 | 2.537836 |
| H | 0.36003 | −1.432019 | 0.017204 | H | 0.367127 | −1.415983 | 0.189905 | H | 0.367503 | −1.41691 | 0.186515 |
| H | 0.892807 | −1.149156 | −1.651865 | H | 0.830347 | −1.216971 | −1.510275 | H | 0.832315 | −1.21548 | −1.51294 |
| H | 1.993975 | 1.118052 | −2.239874 | H | 1.942235 | 0.952508 | −2.271378 | H | 1.944211 | 0.955731 | −2.26998 |
| H | 2.226479 | 2.341098 | −0.968313 | H | 2.295951 | 2.241058 | −1.107397 | H | 2.29602 | 2.242789 | −1.10363 |
| H | 1.330962 | 1.901057 | 1.392941 | H | 1.54275 | 1.957567 | 1.341609 | H | 1.540042 | 1.955723 | 1.343833 |
| H | 0.555849 | 0.352842 | 1.768963 | H | 0.744286 | 0.465099 | 1.849581 | H | 0.741773 | 0.462173 | 1.849016 |
| H | 3.378252 | −1.047787 | −2.126021 | H | 3.267221 | −1.232714 | −2.128903 | H | 3.270099 | −1.22914 | −2.12894 |
| H | 4.535769 | −1.264985 | −0.806071 | H | 4.500923 | −1.428436 | −0.870914 | H | 4.5024 | −1.42589 | −0.86978 |
| H | 3.68389 | −1.698352 | 1.547369 | H | 3.793112 | −1.70903 | 1.540761 | H | 3.791931 | −1.71007 | 1.540739 |
| H | 1.938932 | −1.77855 | 1.841909 | H | 2.068733 | −1.708597 | 1.953559 | H | 2.067092 | −1.71108 | 1.951488 |
| H | 4.789962 | 0.527082 | 0.988898 | H | 4.927137 | 0.445647 | 0.799673 | H | 4.925789 | 0.446122 | 0.803787 |
| H | 3.825433 | 2.000834 | 0.910497 | H | 3.999878 | 1.947206 | 0.70124 | H | 3.997978 | 1.94736 | 0.706344 |
| H | −1.712803 | −0.85869 | −2.320809 | H | −1.634882 | −0.721192 | −2.301194 | H | −1.63354 | −0.72358 | −2.30129 |
| H | −0.494443 | 0.900557 | −1.834718 | H | −0.458257 | 0.969573 | −1.731133 | H | −0.45789 | 0.969059 | −1.73205 |
| H | −3.504969 | −2.778113 | −2.160699 | H | −3.542464 | −2.517031 | −2.332254 | H | −3.54048 | −2.5203 | −2.33159 |
| H | −1.057666 | −3.114687 | −0.248315 | H | −1.231408 | −3.100355 | −0.312228 | H | −1.23001 | −3.10089 | −0.31012 |
| H | −1.028392 | −3.306478 | −2.06063 | H | −1.126855 | −3.224502 | −2.133671 | H | −1.12457 | −3.22656 | −2.13134 |
| H | −3.817912 | −3.192191 | 0.590879 | H | −4.064289 | −3.02247 | 0.371505 | H | −4.06283 | −3.02373 | 0.372451 |
| H | −4.685304 | −1.862036 | −0.191989 | H | −4.765607 | −1.590138 | −0.397785 | H | −4.76494 | −1.59265 | −0.39839 |

**Table S33.** *Cont.*

| | | | | | | | | | | | |
|---|---|---|---|---|---|---|---|---|---|---|---|
| H | −3.499409 | −0.479456 | 1.297525 | H | −3.579694 | −0.371168 | 1.213352 | H | −3.58034 | −0.37106 | 1.21171 |
| H | 3.154737 | −3.336607 | −0.253928 | H | 3.155687 | −3.388549 | −0.153674 | H | 3.157133 | −3.38774 | −0.1569 |
| O | −2.727086 | 3.006132 | −0.761192 | O | −2.635303 | 2.897051 | −0.764187 | O | −2.63582 | 2.897371 | −0.76522 |
| H | −2.909719 | 2.280262 | −0.139975 | H | −2.81749 | 2.286163 | −0.028477 | H | −2.81774 | 2.285848 | −0.02993 |

**Table S34.** Coordinates of Intermediate_3.

| In gas phase | | | | In water | | | | In formic acid | | | |
|---|---|---|---|---|---|---|---|---|---|---|---|
| O | 1.946267 | −3.046597 | −0.085228 | O | 2.086919 | −3.067086 | −0.042858 | O | 0.785864 | −3.3649 | −0.73969 |
| O | −1.737088 | 1.857775 | 0.470484 | O | −1.724141 | 1.840035 | 0.476068 | O | −1.21881 | 1.616625 | 1.618345 |
| N | −2.13428 | −0.15166 | −0.424965 | N | −2.118308 | −0.175978 | −0.403801 | N | −2.2579 | 0.599096 | −0.1055 |
| N | −2.136336 | −1.13708 | 2.782324 | N | −2.581278 | −1.082357 | 2.845776 | N | −2.60929 | −1.76755 | 2.296918 |
| C | −0.886522 | 3.070799 | 0.238951 | C | −0.836729 | 3.052401 | 0.217299 | C | 1.171508 | 3.596119 | −0.65757 |
| C | 1.143684 | 0.559206 | −0.464044 | C | 1.17423 | 0.524411 | −0.445226 | C | 1.107617 | 0.29765 | −0.12319 |
| C | 2.237161 | −1.667861 | 0.023076 | C | 2.316315 | −1.669084 | 0.047198 | C | 1.473757 | −2.18837 | −0.3437 |
| C | 3.632081 | 0.260711 | −0.762811 | C | 3.666567 | 0.285658 | −0.750261 | C | 3.357475 | −0.60634 | −0.81857 |
| C | 2.644596 | 0.282738 | 1.543138 | C | 2.687795 | 0.295845 | 1.557455 | C | 2.554821 | −0.9265 | 1.533744 |
| C | 0.986667 | −0.966192 | −0.508452 | C | 1.044457 | −1.006186 | −0.486668 | C | 0.47303 | −1.04624 | −0.52363 |
| C | 2.371957 | 0.952893 | −1.310708 | C | 2.390133 | 0.944542 | −1.294794 | C | 2.34837 | 0.538203 | −1.00526 |
| N | −0.187733 | 2.697804 | −0.963057 | N | −0.049933 | 2.665497 | −0.913539 | N | 0.579054 | 2.730906 | 0.093913 |
| C | 1.386449 | 0.982376 | 0.997773 | C | 1.415259 | 0.964038 | 1.011588 | C | 1.544671 | 0.217891 | 1.353214 |
| C | 3.445013 | −1.262648 | −0.827645 | C | 3.513621 | −1.241035 | −0.806955 | C | 2.700736 | −1.93331 | −1.22432 |
| C | 2.456111 | −1.239586 | 1.478555 | C | 2.534232 | −1.230124 | 1.499228 | C | 1.898107 | −2.25396 | 1.127341 |
| C | 3.857595 | 0.689963 | 0.694198 | C | 3.890638 | 0.723503 | 0.704289 | C | 3.786759 | −0.67285 | 0.653795 |
| C | −0.11584 | 1.242897 | −1.08377 | C | −0.080543 | 1.209277 | −1.056441 | C | 0.070376 | 1.432263 | −0.37083 |
| C | −2.015694 | −1.260936 | −1.359339 | C | −1.975792 | −1.290774 | −1.326927 | C | −2.43779 | 0.097196 | −1.45384 |
| C | −3.247389 | −2.105483 | −1.188266 | C | −3.247875 | −2.080984 | −1.25107 | C | −3.84308 | −0.41736 | −1.54372 |
| C | −1.915244 | −2.62635 | −0.74207 | C | −1.977883 | −2.650219 | −0.691462 | C | −2.68883 | −1.37948 | −1.58202 |
| C | −4.138507 | −1.443529 | −0.151467 | C | −4.195358 | −1.374781 | −0.299021 | C | −4.52516 | −0.15489 | −0.21331 |
| C | −1.386671 | 0.908431 | −0.344828 | C | −1.367969 | 0.88159 | −0.321142 | C | −1.20576 | 1.225192 | 0.452333 |
| C | −3.255414 | −0.373097 | 0.531522 | C | −3.33084 | −0.32667 | 0.441647 | C | −3.38424 | 0.228175 | 0.759535 |
| C | −2.659112 | −0.800179 | 1.809118 | C | −2.914663 | −0.74649 | 1.792426 | C | −2.96416 | −0.88948 | 1.634261 |
| H | −0.313377 | 3.177701 | 1.162213 | H | −0.279265 | 3.157135 | 1.14648 | H | 1.310615 | 3.389612 | −1.71534 |
| H | 0.704381 | 3.169575 | −1.068528 | H | −0.370178 | 3.135931 | −1.754937 | H | 0.382134 | 2.915061 | 1.082076 |

**Table S34.** *Cont.*

| In gas phase | | | | In water | | | | In formic acid | | | |
|---|---|---|---|---|---|---|---|---|---|---|---|
| H | 4.489566 | 0.554725 | −1.375718 | H | 4.517073 | 0.594073 | −1.366923 | H | 4.228626 | −0.41498 | −1.45335 |
| H | 2.795135 | 0.592683 | 2.581756 | H | 2.83599 | 0.612482 | 2.594914 | H | 2.852464 | −0.97045 | 2.586318 |
| H | 0.150295 | −1.288486 | 0.120676 | H | 0.20929 | −1.348204 | 0.135378 | H | −0.40253 | −1.26352 | 0.100989 |
| H | 0.80031 | −1.318409 | −1.532168 | H | 0.869617 | −1.358251 | −1.511947 | H | 0.143201 | −1.02544 | −1.5704 |
| H | 2.207902 | 0.665847 | −2.357821 | H | 2.228772 | 0.643911 | −2.338411 | H | 2.048156 | 0.604242 | −2.05964 |
| H | 2.52341 | 2.040813 | −1.288639 | H | 2.484128 | 2.035661 | −1.27347 | H | 2.833268 | 1.484228 | −0.7332 |
| H | 1.540471 | 2.067329 | 1.055941 | H | 1.545656 | 2.05159 | 1.046361 | H | 2.00891 | 1.163208 | 1.662208 |
| H | 0.519418 | 0.727346 | 1.625133 | H | 0.556445 | 0.698369 | 1.645546 | H | 0.673703 | 0.051897 | 1.996845 |
| H | 3.285056 | −1.598766 | −1.85884 | H | 3.355378 | −1.580297 | −1.837697 | H | 2.387137 | −1.90977 | −2.27482 |
| H | 4.344592 | −1.76782 | −0.4532 | H | 4.421925 | −1.729569 | −0.430069 | H | 3.412906 | −2.76055 | −1.10698 |
| H | 3.34338 | −1.744392 | 1.881726 | H | 3.434576 | −1.7166 | 1.896717 | H | 2.60249 | −3.08462 | 1.267174 |
| H | 1.594382 | −1.556967 | 2.078057 | H | 1.680543 | −1.561249 | 2.103025 | H | 1.013458 | −2.45812 | 1.74296 |
| H | 4.763627 | 0.215863 | 1.087034 | H | 4.807096 | 0.267057 | 1.096368 | H | 4.519253 | −1.47646 | 0.792742 |
| H | 4.012038 | 1.77425 | 0.750256 | H | 4.017789 | 1.811389 | 0.753972 | H | 4.270124 | 0.266875 | 0.946572 |
| H | −1.568977 | −1.012458 | −2.313533 | H | −1.44964 | −1.07038 | −2.24601 | H | −1.97649 | 0.672004 | −2.24634 |
| H | −0.190203 | 0.94305 | −2.136443 | H | −0.155633 | 0.932664 | −2.11206 | H | −0.13317 | 1.509939 | −1.44126 |
| H | −3.721915 | −2.540446 | −2.059577 | H | −3.661775 | −2.504179 | −2.157447 | H | −4.41626 | −0.25508 | −2.44806 |
| H | −1.700086 | −2.701281 | 0.320085 | H | −1.856733 | −2.715538 | 0.38625 | H | −2.49447 | −2.00949 | −0.71837 |
| H | −1.434462 | −3.383801 | −1.350552 | H | −1.485173 | −3.434019 | −1.254471 | H | −2.45118 | −1.83169 | −2.538 |
| H | −4.535187 | −2.144697 | 0.584983 | H | −4.678414 | −2.050136 | 0.40872 | H | −5.09559 | −1.00728 | 0.160099 |
| H | −4.976809 | −0.947396 | −0.643983 | H | −4.967333 | −0.850654 | −0.863211 | H | −5.20053 | 0.696752 | −0.30845 |
| H | −3.780979 | 0.571258 | 0.691301 | H | −3.827421 | 0.642399 | 0.524928 | H | −3.64426 | 1.068464 | 1.406783 |
| H | 2.696819 | −3.546989 | 0.258858 | H | 2.878167 | −3.514888 | 0.284597 | H | 1.386507 | −4.11171 | −0.61606 |
| O | −1.660545 | 4.159229 | 0.09748 | O | −1.60847 | 4.143374 | 0.058361 | O | 1.633902 | 4.737941 | −0.2685 |
| H | −1.974951 | 4.199838 | −0.818594 | H | −2.132277 | 4.08108 | −0.755212 | H | 1.521638 | 4.918875 | 0.681353 |

**Table S35.** Coordinates of Ethylene glycol.

| In gas phase | | | | In water | | | |
|---|---|---|---|---|---|---|---|
| C | −0.5736 | 0.49453 | 0.00006 | C | −0.57533 | 0.492981 | −0.00013 |
| C | 0.57357 | −0.4945 | −0.0017 | C | 0.575315 | −0.4929 | −0.00204 |
| H | −0.4858 | 1.13562 | 0.88838 | H | −0.49514 | 1.135195 | 0.887541 |
| H | 0.48683 | −1.1372 | 0.88557 | H | 0.496376 | −1.137 | 0.884405 |
| H | 0.48797 | −1.1335 | −0.8916 | H | 0.497919 | −1.13244 | −0.89179 |
| H | −0.4891 | 1.13537 | −0.8888 | H | −0.49928 | 1.134539 | −0.88863 |
| O | 1.76773 | 0.26036 | 0.0005 | O | 1.77181 | 0.262123 | 0.000823 |
| H | 2.51269 | −0.3478 | 0.00331 | H | 2.513567 | −0.35257 | 0.003236 |
| O | −1.7677 | −0.2604 | 0.00235 | O | −1.77179 | −0.26219 | 0.002848 |
| H | −2.5127 | 0.34767 | −0.0098 | H | −2.51355 | 0.352342 | −0.01112 |

**Table S36.** Coordinates of TS_formic acid1.

| In gas phase | | | |
|---|---|---|---|
| C | 1.115906 | −0.311956 | −0.01811 |
| C | −0.376569 | −0.157842 | −0.359204 |
| N | −2.59242 | −1.109167 | −0.218751 |
| C | −3.596234 | −2.139644 | −0.091604 |
| C | 1.75748 | −1.306064 | −1.009522 |
| C | 1.824274 | 1.051657 | −0.189849 |
| C | −4.92324 | −1.44355 | −0.137632 |
| C | 1.341128 | −0.810735 | 1.423072 |
| C | −4.66319 | 0.052633 | −0.311637 |
| C | −1.26422 | −1.404628 | −0.189729 |
| C | 3.25982 | −1.452522 | −0.73202 |
| C | 3.329349 | 0.908836 | 0.078505 |
| N | −1.04407 | 0.904473 | 0.440028 |
| C | −2.485384 | 1.385527 | −0.207539 |
| C | 2.848728 | −0.946761 | 1.695952 |
| N | −2.773559 | 2.566041 | −0.250404 |
| O | −0.842143 | −2.531393 | 0.001901 |
| O | −0.634763 | 3.53854 | 0.511963 |
| C | 3.944548 | −0.098324 | −0.900944 |

**Table S36.** *Cont.*

| **In gas phase** | | | |
|---|---|---|---|
| C | 3.455716 | −1.951657 | 0.705348 |
| O | 3.854359 | −2.331582 | −1.669172 |
| C | 3.535005 | 0.414941 | 1.51853 |
| H | −1.708154 | 3.296886 | 0.175856 |
| H | −0.50063 | 1.789843 | 0.495069 |
| H | −5.296734 | 0.496645 | −1.079807 |
| H | −3.379236 | −3.079907 | −0.58369 |
| H | −5.755985 | −1.896928 | −0.661244 |
| H | −4.832229 | 0.608205 | 0.615104 |
| H | −0.450581 | 0.172712 | −1.406158 |
| H | 1.626118 | −0.949669 | −2.0404 |
| H | 1.272184 | −2.283505 | −0.922805 |
| H | 1.658006 | 1.436303 | −1.205769 |
| H | −1.199689 | 0.580716 | 1.396844 |
| H | 1.434192 | 1.813231 | 0.496185 |
| H | 0.840502 | −1.7738 | 1.56479 |
| H | 0.914282 | −0.093446 | 2.139572 |
| H | 3.795315 | 1.890785 | −0.049861 |
| H | 2.993819 | −1.306836 | 2.720434 |
| H | 3.820189 | 0.234355 | −1.938029 |
| H | 5.01884 | −0.217361 | −0.718372 |
| H | 2.976783 | −2.934512 | 0.818731 |
| H | 4.528405 | −2.081586 | 0.890407 |
| H | 3.437219 | −3.196144 | −1.566665 |
| H | 3.120546 | 1.142227 | 2.226992 |
| H | 4.606407 | 0.323361 | 1.733673 |
| C | −4.446005 | −2.077287 | 1.142524 |
| H | −4.92564 | −2.994567 | 1.464716 |
| H | −4.117939 | −1.419073 | 1.941153 |
| C | −3.178159 | 0.144114 | −0.721975 |
| H | −3.107149 | 0.160167 | −1.819307 |
| C | 0.077426 | 4.302588 | −0.292177 |
| H | −0.532845 | 4.963275 | −0.938106 |
| O | 1.284556 | 4.30359 | −0.33936 |

**Table S37.** Coordinates of TS_PEG.

| In gas phase | | | | In water | | | |
|---|---|---|---|---|---|---|---|
| C | 1.271231 | −0.389215 | 0.005184 | C | 1.231942 | −0.46122 | 0.015893 |
| C | −0.233612 | −0.422128 | −0.323601 | C | −0.271159 | −0.4392 | −0.31851 |
| N | −2.237262 | −1.779172 | −0.357724 | N | −2.332703 | −1.69669 | −0.384 |
| C | −3.038412 | −2.978248 | −0.362445 | C | −3.196137 | −2.85337 | −0.38302 |
| C | 2.05266 | −1.128188 | −1.103029 | C | 1.979582 | −1.23788 | −1.09012 |
| C | 1.769626 | 1.073947 | 0.033279 | C | 1.781002 | 0.983477 | 0.023948 |
| C | −4.463988 | −2.530153 | −0.243474 | C | −4.591874 | −2.33403 | −0.22085 |
| C | 1.58098 | −1.036168 | 1.370799 | C | 1.52596 | −1.10415 | 1.386038 |
| C | −4.47655 | −1.00271 | −0.185437 | C | −4.522467 | −0.80939 | −0.14364 |
| C | −0.88183 | −1.823452 | −0.341184 | C | −0.987608 | −1.80464 | −0.33175 |
| C | 3.560374 | −1.089468 | −0.824008 | C | 3.488901 | −1.24861 | −0.81889 |
| C | 3.281889 | 1.120132 | 0.302244 | C | 3.295131 | 0.980903 | 0.284558 |
| N | −1.044262 | 0.398726 | 0.611802 | N | −1.056262 | 0.421946 | 0.61285 |
| C | −2.53975 | 0.642075 | 0.129489 | C | −2.495531 | 0.742499 | 0.090641 |
| C | 3.092536 | −0.987173 | 1.644816 | C | 3.040661 | −1.10404 | 1.650123 |
| N | −3.080786 | 1.72478 | 0.332047 | N | −2.965944 | 1.872454 | 0.238954 |
| O | −0.260808 | −2.873935 | −0.30039 | O | −0.415621 | −2.88612 | −0.26755 |
| O | −0.99451 | 2.848477 | 1.043116 | O | −0.870947 | 2.919842 | 1.036271 |
| C | 4.036531 | 0.360707 | −0.795941 | C | 4.015271 | 0.184429 | −0.81095 |
| C | −0.603467 | 3.643129 | −0.053411 | C | −0.397101 | 3.682643 | −0.04466 |
| C | 3.835462 | −1.745984 | 0.534885 | C | 3.751671 | −1.89921 | 0.544547 |
| O | 4.27589 | −1.733843 | −1.862268 | O | 4.173951 | −1.93553 | −1.85686 |
| C | 3.568899 | 0.47227 | 1.6648 | C | 3.567665 | 0.337735 | 1.652024 |
| C | −1.393773 | 4.939538 | −0.068773 | C | −1.050142 | 5.054984 | −0.04892 |
| H | −2.087093 | 2.497166 | 0.823962 | H | −1.985491 | 2.573161 | 0.740449 |
| H | −0.738971 | 1.480375 | 0.795012 | H | −0.675547 | 1.440677 | 0.795076 |
| H | −5.213666 | −0.572454 | −0.863875 | H | −5.260367 | −0.33509 | −0.79108 |
| H | −2.69847 | −3.779776 | −1.007002 | H | −2.925168 | −3.658 | −1.05554 |
| H | −5.241339 | −3.041339 | −0.798338 | H | −5.404572 | −2.79603 | −0.76727 |
| H | −4.69874 | −0.629564 | 0.818177 | H | −4.685525 | −0.44662 | 0.875196 |
| H | −0.374277 | 0.030291 | −1.316895 | H | −0.390232 | 0.013842 | −1.31291 |
| H | 1.865201 | −0.654513 | −2.076546 | H | 1.798861 | −0.76714 | −2.06594 |
| H | 1.71868 | −2.168307 | −1.160917 | H | 1.611161 | −2.26661 | −1.13611 |

**Table S37.** *Cont.*

| In gas phase | | | | In water | | | |
|---|---|---|---|---|---|---|---|
| H | −0.766175 | 3.124505 | −1.01523 | H | −0.602541 | 3.194457 | −1.01535 |
| H | 1.539809 | 1.561197 | −0.925287 | H | 1.560522 | 1.468243 | −0.93764 |
| H | −1.075356 | −0.060352 | 1.524763 | H | −1.136071 | −0.03652 | 1.525119 |
| H | 1.257971 | 1.644627 | 0.818229 | H | 1.297167 | 1.578284 | 0.808417 |
| H | 1.228393 | −2.071784 | 1.377022 | H | 1.139115 | −2.12709 | 1.403793 |
| H | 1.058111 | −0.490565 | 2.169905 | H | 1.024665 | −0.53765 | 2.182907 |
| H | 3.605886 | 2.166479 | 0.315207 | H | 3.655629 | 2.015173 | 0.282935 |
| H | 3.295349 | −1.458335 | 2.612875 | H | 3.233209 | −1.57213 | 2.621326 |
| H | 3.860922 | 0.810909 | −1.779986 | H | 3.844935 | 0.63403 | −1.7967 |
| H | 5.117136 | 0.379404 | −0.612217 | H | 5.097056 | 0.170715 | −0.63142 |
| H | 3.504745 | −2.79357 | 0.504979 | H | 3.388962 | −2.93599 | 0.526888 |
| H | 4.916759 | −1.742965 | 0.715749 | H | 4.833464 | −1.92673 | 0.721953 |
| H | 3.972735 | −2.649502 | −1.902132 | H | 3.845822 | −2.84447 | −1.8704 |
| H | 3.054018 | 1.025035 | 2.460203 | H | 3.075835 | 0.916087 | 2.443552 |
| H | 4.643386 | 0.514029 | 1.880102 | H | 4.64418 | 0.343818 | 1.860819 |
| H | −2.463985 | 4.700847 | −0.158912 | H | −2.140084 | 4.931708 | −0.13496 |
| H | −1.24004 | 5.452688 | 0.891147 | H | −0.844111 | 5.547706 | 0.911562 |
| C | −3.802401 | −3.256271 | 0.898357 | C | −3.941149 | −3.10926 | 0.894655 |
| H | −4.097744 | −4.282117 | 1.087861 | H | −4.286682 | −4.12103 | 1.07419 |
| H | −3.534445 | −2.679447 | 1.778319 | H | −3.620484 | −2.55819 | 1.773673 |
| C | −3.05146 | −0.580172 | −0.602209 | C | −3.092572 | −0.45875 | −0.61046 |
| H | −3.041223 | −0.352306 | −1.678203 | H | −3.104893 | −0.24048 | −1.68709 |
| H | 0.466498 | 3.876012 | 0.015193 | H | 0.692849 | 3.807856 | 0.023756 |
| O | −0.926625 | 5.702229 | −1.16388 | O | −0.520362 | 5.785894 | −1.14015 |
| H | −1.439146 | 6.514767 | −1.210715 | H | −0.925748 | 6.659796 | −1.14142 |

**Table S38.** Coordinates of TS_water.

| In gas phase | | | | In water | | | |
|---|---|---|---|---|---|---|---|
| C | 1.162427 | 0.099205 | −0.085222 | C | 1.144494 | 0.101587 | −0.07823 |
| C | −0.333523 | 0.173616 | −0.450586 | C | −0.344498 | 0.195521 | −0.4712 |
| N | −2.489251 | −0.89897 | −0.211021 | N | −2.506343 | −0.8905 | −0.32241 |
| C | −3.433027 | −1.962188 | 0.031147 | C | −3.447296 | −1.95736 | −0.07741 |
| C | 1.85616 | −0.947016 | −0.984235 | C | 1.851172 | −0.90094 | −1.01768 |
| C | 1.834916 | 1.465593 | −0.348288 | C | 1.824589 | 1.476819 | −0.26801 |
| C | −4.793906 | −1.335833 | −0.027306 | C | −4.784179 | −1.30166 | 0.090633 |
| C | 1.382976 | −0.273422 | 1.395563 | C | 1.341754 | −0.34118 | 1.385782 |
| C | −4.615828 | 0.152132 | −0.330555 | C | −4.592202 | 0.204058 | −0.09108 |
| C | −1.150032 | −1.104037 | −0.158913 | C | −1.17211 | −1.08862 | −0.26013 |
| C | 3.35648 | −1.02541 | −0.673047 | C | 3.345365 | −0.99854 | −0.68539 |
| C | 3.340519 | 1.390971 | −0.047554 | C | 3.32493 | 1.384793 | 0.052026 |
| N | −1.033957 | 1.291929 | 0.225611 | N | −1.060743 | 1.284928 | 0.248809 |
| C | −2.493742 | 1.585841 | −0.336065 | C | −2.459836 | 1.606228 | −0.36376 |
| C | 2.888346 | −0.344315 | 1.700765 | C | 2.842379 | −0.43042 | 1.709182 |
| N | −2.906923 | 2.738721 | −0.405133 | N | −2.818541 | 2.776192 | −0.51866 |
| O | −0.661808 | −2.185827 | 0.129736 | O | −0.687703 | −2.18965 | −0.0228 |
| O | −0.718141 | 3.732019 | 0.193291 | O | −0.64661 | 3.743743 | 0.175334 |
| C | 4.003307 | 0.331683 | −0.936645 | C | 3.99975 | 0.367139 | −0.87576 |
| C | 3.542476 | −1.4039 | 0.801498 | C | 3.509031 | −1.44815 | 0.771336 |
| O | 3.995693 | −1.955229 | −1.528904 | O | 3.994716 | −1.89484 | −1.57669 |
| C | 3.538554 | 1.018928 | 1.42942 | C | 3.49955 | 0.942 | 1.512262 |
| H | −1.825009 | 3.501172 | −0.059897 | H | −1.751021 | 3.493652 | −0.16316 |
| H | −0.603051 | 2.359404 | 0.148181 | H | −0.599378 | 2.322873 | 0.219732 |
| H | −5.284888 | 0.492969 | −1.121174 | H | −5.355293 | 0.640773 | −0.73598 |
| H | −3.18268 | −2.929507 | −0.387543 | H | −3.29222 | −2.87927 | −0.62453 |
| H | −5.617974 | −1.870539 | −0.483992 | H | −5.669943 | −1.77208 | −0.31761 |
| H | −4.80243 | 0.777782 | 0.546724 | H | −4.616047 | 0.73285 | 0.866483 |
| H | −0.408252 | 0.381383 | −1.528908 | H | −0.396726 | 0.459539 | −1.53694 |
| H | 1.733789 | −0.675417 | −2.042022 | H | 1.742604 | −0.57608 | −2.06153 |
| H | 1.398534 | −1.928873 | −0.83085 | H | 1.393004 | −1.88906 | −0.92007 |
| H | 1.671496 | 1.758849 | −1.395707 | H | 1.674746 | 1.822056 | −1.30069 |
| H | −1.116943 | 1.085943 | 1.223448 | H | −1.183347 | 1.028398 | 1.232336 |

**Table S38.** *Cont.*

| In gas phase | | | | In water | | | |
|---|---|---|---|---|---|---|---|
| H | 1.390415 | 2.244942 | 0.281811 | H | 1.376886 | 2.226157 | 0.39419 |
| H | 0.907266 | −1.235476 | 1.607825 | H | 0.863278 | −1.3113 | 1.545626 |
| H | 0.923931 | 0.486731 | 2.044698 | H | 0.872127 | 0.384602 | 2.064249 |
| H | 3.790642 | 2.370169 | −0.24445 | H | 3.779444 | 2.370585 | −0.09322 |
| H | 3.026824 | −0.618605 | 2.752406 | H | 2.964597 | −0.75487 | 2.748221 |
| H | 3.887523 | 0.578153 | −1.998765 | H | 3.898125 | 0.668353 | −1.92543 |
| H | 5.077413 | 0.261921 | −0.728445 | H | 5.070447 | 0.288787 | −0.65129 |
| H | 3.088031 | −2.387915 | 0.98323 | H | 3.053637 | −2.43971 | 0.898987 |
| H | 4.615045 | −1.488069 | 1.012758 | H | 4.578263 | −1.54091 | 0.997092 |
| H | 3.598361 | −2.820354 | −1.368674 | H | 3.589748 | −2.76481 | −1.46148 |
| H | 3.090388 | 1.785238 | 2.073853 | H | 3.041901 | 1.677564 | 2.184887 |
| H | 4.609004 | 0.97892 | 1.664451 | H | 4.566136 | 0.887187 | 1.761726 |
| C | −4.249786 | −1.838937 | 1.283889 | C | −4.105529 | −1.94423 | 1.271692 |
| H | −4.673243 | −2.748781 | 1.694291 | H | −4.508555 | −2.88182 | 1.63775 |
| H | −3.930258 | −1.103413 | 2.015976 | H | −3.676041 | −1.29076 | 2.025267 |
| C | −3.144787 | 0.290874 | −0.773081 | C | −3.196723 | 0.337894 | −0.73924 |
| H | −3.091388 | 0.242005 | −1.870986 | H | −3.305809 | 0.329587 | −1.83203 |
| H | −0.336775 | 4.209193 | −0.552121 | H | −0.178249 | 4.069569 | −0.60367 |

**Table S39.** Coordinates of TS_formic_acid2.

| In gas phase | | | | In water | | | | In formic acid | | | |
|---|---|---|---|---|---|---|---|---|---|---|---|
| C | 1.317545 | −0.185828 | −0.296073 | C | 1.343527 | −0.16644 | −0.297472 | C | 1.34304 | −0.16704 | −0.29748 |
| C | −0.144776 | −0.464654 | −0.698056 | C | −0.123659 | −0.455356 | −0.678118 | C | −0.12403 | −0.45593 | −0.67855 |
| N | −2.221308 | −1.590782 | −0.138399 | N | −2.179661 | −1.599697 | −0.101341 | N | −2.18038 | −1.59983 | −0.10185 |
| C | −3.151867 | −2.288514 | 0.71557 | C | −3.102789 | −2.321273 | 0.742058 | C | −3.10375 | −2.32087 | 0.741763 |
| C | 2.139015 | −1.477196 | −0.490789 | C | 2.163005 | −1.46498 | −0.454435 | C | 2.162621 | −1.46545 | −0.45483 |
| N | −0.945981 | 0.791369 | −0.820107 | N | −0.942124 | 0.78454 | −0.791435 | N | −0.94213 | 0.784267 | −0.79264 |
| C | 1.897829 | 0.898492 | −1.233251 | C | 1.919878 | 0.881252 | −1.277353 | C | 1.919493 | 0.881152 | −1.27678 |
| C | −4.524376 | −1.990329 | 0.204609 | C | −4.471867 | −2.094064 | 0.189783 | C | −4.47288 | −2.0924 | 0.190078 |
| C | −2.618503 | 0.699205 | −0.811562 | C | −2.668299 | 0.653448 | −0.835156 | C | −2.66746 | 0.653929 | −0.83441 |
| C | 1.468599 | 0.301714 | 1.162138 | C | 1.508553 | 0.372931 | 1.139717 | C | 1.507718 | 0.371714 | 1.139994 |
| C | −4.396709 | −1.114368 | −1.033127 | C | −4.345253 | −1.254551 | −1.071792 | C | −4.3461 | −1.25259 | −1.0713 |

**Table S39.** *Cont.*

| In gas phase | | | | In water | | | | In formic acid | | | |
|---|---|---|---|---|---|---|---|---|---|---|---|
| N | −3.276528 | 1.680131 | −0.552164 | N | −3.331319 | 1.635703 | −0.624103 | N | −3.33028 | 1.636098 | −0.62184 |
| C | −0.914749 | −1.450314 | 0.204727 | C | −0.881301 | −1.43166 | 0.242355 | C | −0.88195 | −1.43214 | 0.241854 |
| C | −1.239425 | 3.937409 | 0.636883 | C | −1.413188 | 3.988153 | 0.668693 | C | −1.41019 | 3.988836 | 0.667621 |
| C | 3.618809 | −1.232705 | −0.169552 | C | 3.644704 | −1.21096 | −0.15185 | C | 3.644243 | −1.21147 | −0.15184 |
| C | 3.38268 | 1.138909 | −0.924041 | C | 3.406427 | 1.13225 | −0.985638 | C | 3.405974 | 1.132103 | −0.98468 |
| C | 2.95507 | 0.549163 | 1.471264 | C | 2.997196 | 0.627552 | 1.432387 | C | 2.996289 | 0.626332 | 1.433036 |
| O | −2.449307 | 3.913405 | 0.22267 | O | −2.556746 | 3.945103 | 0.067289 | O | −2.55645 | 3.944629 | 0.07174 |
| O | −0.430308 | 3.002689 | 0.639054 | O | −0.659552 | 3.042248 | 0.878281 | O | −0.65305 | 3.044298 | 0.871046 |
| O | −0.403142 | −2.081856 | 1.113696 | O | −0.37056 | −2.029082 | 1.181883 | O | −0.37117 | −2.02999 | 1.18102 |
| C | −2.902879 | −0.748384 | −1.133743 | C | −2.870271 | −0.810621 | −1.135366 | C | −2.87081 | −0.80984 | −1.1353 |
| C | 4.172665 | −0.162861 | −1.108241 | C | 4.193979 | −0.176396 | −1.131651 | C | 4.193626 | −0.17644 | −1.13109 |
| C | 3.746148 | −0.753532 | 1.280942 | C | 3.784242 | −0.683359 | 1.280565 | C | 3.783461 | −0.68445 | 1.280827 |
| O | 4.379436 | −2.405452 | −0.398144 | O | 4.402623 | −2.398102 | −0.33994 | O | 4.402279 | −2.39843 | −0.34032 |
| C | 3.510671 | 1.624355 | 0.527351 | C | 3.551531 | 1.665422 | 0.447103 | C | 3.550702 | 1.664695 | 0.448319 |
| C | −4.072451 | −1.410755 | 1.517995 | C | −4.080883 | −1.465485 | 1.500757 | C | −4.08084 | −1.46448 | 1.501047 |
| H | −2.794731 | 2.90041 | −0.14483 | H | −2.841759 | 2.938832 | −0.238445 | H | −2.84115 | 2.93825 | −0.2353 |
| H | −0.709584 | 1.257999 | −1.696301 | H | −0.708474 | 1.279979 | −1.652614 | H | −0.70889 | 1.278344 | −1.65473 |
| H | −0.730165 | 1.512766 | −0.083468 | H | −0.776906 | 1.458303 | −0.021785 | H | −0.77545 | 1.459313 | −0.02432 |
| H | −0.923953 | 4.920311 | 1.022581 | H | −1.14421 | 5.001587 | 1.004015 | H | −1.14187 | 5.002023 | 1.004279 |
| H | −4.702935 | −1.6747 | −1.919155 | H | −4.584943 | −1.861745 | −1.946389 | H | −4.58664 | −1.85933 | −1.94599 |
| H | −2.82105 | −3.254167 | 1.074371 | H | −2.747867 | −3.268867 | 1.124768 | H | −2.74936 | −3.26879 | 1.12416 |
| H | −5.287547 | −2.759537 | 0.210221 | H | −5.198393 | −2.897408 | 0.198196 | H | −5.20009 | −2.89513 | 0.198546 |
| H | −4.990344 | −0.198903 | −0.972171 | H | −4.996034 | −0.377273 | −1.06944 | H | −4.99609 | −0.37473 | −1.06837 |
| H | −0.131896 | −0.916665 | −1.700938 | H | −0.121983 | −0.911715 | −1.677343 | H | −0.12208 | −0.91253 | −1.67768 |
| H | 2.063534 | −1.826635 | −1.530173 | H | 2.073973 | −1.846507 | −1.480703 | H | 2.073883 | −1.84657 | −1.48129 |
| H | 1.744805 | −2.263337 | 0.162198 | H | 1.777309 | −2.229948 | 0.227384 | H | 1.776778 | −2.2307 | 0.226592 |
| H | 1.775477 | 0.588703 | −2.282761 | H | 1.788591 | 0.53522 | −2.312242 | H | 1.788472 | 0.535577 | −2.31188 |
| H | −2.505345 | −0.966925 | −2.134892 | H | −2.432502 | −1.020733 | −2.118685 | H | −2.43353 | −1.02 | −2.11885 |
| H | 1.360482 | 1.845168 | −1.090228 | H | 1.382583 | 1.832667 | −1.171138 | H | 1.382106 | 1.832471 | −1.17015 |
| H | 1.06526 | −0.452077 | 1.842149 | H | 1.104521 | −0.351338 | 1.851034 | H | 1.103674 | −0.35293 | 1.850898 |
| H | 0.911804 | 1.234419 | 1.310939 | H | 0.95311 | 1.311797 | 1.25711 | H | 0.952187 | 1.310469 | 1.257719 |
| H | 3.769122 | 1.903257 | −1.607183 | H | 3.789224 | 1.870953 | −1.69774 | H | 3.788863 | 1.871133 | −1.6964 |
| H | 3.047374 | 0.886582 | 2.50928 | H | 3.099355 | 0.999018 | 2.457793 | H | 3.098181 | 0.997379 | 2.458618 |

**Table S39.** *Cont.*

| | | | | | | | | | | | |
|---|---|---|---|---|---|---|---|---|---|---|---|
| H | 4.100832 | −0.526698 | −2.140483 | H | 4.109204 | −0.571001 | −2.151639 | H | 4.109112 | −0.57066 | −2.15124 |
| H | 5.235019 | −0.009735 | −0.885929 | H | 5.258088 | −0.012817 | −0.923099 | H | 5.257684 | −0.01292 | −0.92227 |
| H | 3.362099 | −1.531212 | 1.955892 | H | 3.406818 | −1.438182 | 1.983878 | H | 3.405951 | −1.43957 | 1.983774 |
| H | 4.808084 | −0.606224 | 1.511279 | H | 4.8473 | −0.525297 | 1.499025 | H | 4.846477 | −0.52645 | 1.499519 |
| H | 4.05826 | −3.085431 | 0.207135 | H | 4.07348 | −3.056643 | 0.286301 | H | 4.072412 | −3.05761 | 0.284854 |
| H | 2.952527 | 2.559163 | 0.660321 | H | 3.004421 | 2.610547 | 0.551525 | H | 3.003408 | 2.60968 | 0.553051 |
| H | 4.562632 | 1.827794 | 0.761536 | H | 4.606691 | 1.86844 | 0.666394 | H | 4.605793 | 1.867766 | 0.6679 |
| H | −4.495552 | −1.830402 | 2.423367 | H | −4.512921 | −1.88301 | 2.402856 | H | −4.51282 | −1.8819 | 2.403222 |
| H | −3.850694 | −0.348077 | 1.555459 | H | −3.896257 | −0.395193 | 1.521515 | H | −3.8954 | −0.39434 | 1.521997 |

**Table S40.** Coordinates of SCA.

| In gas phase | | | | In water | | | | In formic acid | | | |
|---|---|---|---|---|---|---|---|---|---|---|---|
| C | 1.117437 | 0.265042 | −0.163127 | C | 1.118075 | 0.259185 | −0.153127 | C | 1.118087 | 0.259269 | −0.153183 |
| C | −0.369886 | 0.258572 | −0.550878 | C | −0.371447 | 0.252438 | −0.533161 | C | −0.37143 | 0.252545 | −0.53323 |
| C | 1.795105 | −0.987564 | −0.757645 | C | 1.794107 | −0.992449 | −0.751696 | C | 1.794056 | −0.992455 | −0.751633 |
| C | 1.800924 | 1.504027 | −0.783523 | C | 1.79356 | 1.499029 | −0.781928 | C | 1.793673 | 1.499015 | −0.782056 |
| C | 1.345041 | 0.278459 | 1.363783 | C | 1.357042 | 0.277209 | 1.371308 | C | 1.357021 | 0.277374 | 1.371262 |
| C | −1.194843 | −0.935594 | −0.031945 | C | −1.202713 | −0.938237 | −0.020263 | C | −1.202664 | −0.938141 | −0.020263 |
| N | −1.049301 | 1.488361 | −0.12398 | N | −1.050132 | 1.47779 | −0.094295 | N | −1.050082 | 1.477979 | −0.094522 |
| C | 3.296603 | −0.996496 | −0.448203 | C | 3.297783 | −0.996529 | −0.452625 | C | 3.297728 | −0.996618 | −0.452544 |
| C | 3.304838 | 1.501713 | −0.474856 | C | 3.299421 | 1.501112 | −0.484054 | C | 3.299529 | 1.501027 | −0.484135 |
| C | 2.851535 | 0.281036 | 1.668544 | C | 2.86601 | 0.283085 | 1.664229 | C | 2.865982 | 0.283164 | 1.664223 |
| N | −2.502434 | −0.817572 | −0.390908 | N | −2.505195 | −0.811265 | −0.375873 | N | −2.505175 | −0.811226 | −0.375945 |
| O | −0.765307 | −1.870956 | 0.623986 | O | −0.781065 | −1.885 | 0.634229 | O | −0.780958 | −1.884821 | 0.634227 |
| C | 3.951992 | 0.239822 | −1.059922 | C | 3.94537 | 0.239926 | −1.072497 | C | 3.94541 | 0.23976 | −1.072473 |
| C | 3.494934 | −0.979739 | 1.072936 | C | 3.509479 | −0.976748 | 1.066127 | C | 3.50937 | −0.976754 | 1.06622 |
| O | 3.922907 | −2.122363 | −1.036736 | O | 3.921248 | −2.128787 | −1.04384 | O | 3.921138 | −2.128897 | −1.043679 |
| C | 3.507179 | 1.523089 | 1.047926 | C | 3.513428 | 1.52595 | 1.03685 | C | 3.51351 | 1.525939 | 1.036773 |
| C | −3.536077 | −1.603456 | 0.235697 | C | −3.5488 | −1.605554 | 0.224787 | C | −3.54872 | −1.605595 | 0.224696 |
| C | −3.026492 | 0.437538 | −0.95957 | C | −3.021761 | 0.445283 | −0.954063 | C | −3.021813 | 0.445326 | −0.954025 |
| C | −4.834395 | −0.912103 | −0.029885 | C | −4.840946 | −0.90536 | −0.043982 | C | −4.840929 | −0.905497 | −0.044055 |
| C | −4.268125 | −0.930265 | 1.364489 | C | −4.288138 | −0.94231 | 1.356089 | C | −4.288148 | −0.942525 | 1.356016 |
| C | −4.558323 | 0.316573 | −0.885331 | C | −4.553562 | 0.32485 | −0.892537 | C | −4.553624 | 0.324916 | −0.892355 |

**Table S40.** *Cont.*

| In gas phase | | | | In water | | | | In formic acid | | | |
|---|---|---|---|---|---|---|---|---|---|---|---|
| C | −2.42032 | 1.595934 | −0.178728 | C | −2.412517 | 1.602912 | −0.17347 | C | −2.41256 | 1.602943 | −0.173434 |
| H | −0.406232 | 0.213296 | −1.651963 | H | −0.413233 | 0.210535 | −1.633684 | H | −0.413197 | 0.210579 | −1.633756 |
| H | 1.663215 | −1.004753 | −1.848112 | H | 1.65063 | −1.010148 | −1.840574 | H | 1.650622 | −1.010245 | −1.840517 |
| H | 1.328715 | −1.888003 | −0.344551 | H | 1.336743 | −1.89504 | −0.333994 | H | 1.336591 | −1.89496 | −0.33386 |
| H | 1.636177 | 1.502547 | −1.86951 | H | 1.620778 | 1.493416 | −1.866501 | H | 1.620939 | 1.493326 | −1.866638 |
| H | 1.354313 | 2.428386 | −0.397072 | H | 1.350425 | 2.422595 | −0.391494 | H | 1.350531 | 2.422646 | −0.391766 |
| H | 0.861211 | −0.595769 | 1.809217 | H | 0.881609 | −0.598747 | 1.822768 | H | 0.881482 | −0.598493 | 1.822778 |
| H | 0.890287 | 1.172996 | 1.813507 | H | 0.902523 | 1.169725 | 1.82227 | H | 0.90258 | 1.169969 | 1.822176 |
| H | −0.571493 | 2.049028 | 0.567398 | H | −0.565315 | 2.062455 | 0.572823 | H | −0.565376 | 2.062328 | 0.572948 |
| H | 3.763964 | 2.390715 | −0.921523 | H | 3.752922 | 2.389596 | −0.93661 | H | 3.753103 | 2.389458 | −0.936732 |
| H | 2.996745 | 0.289525 | 2.75454 | H | 3.019929 | 0.293181 | 2.748759 | H | 3.019869 | 0.293323 | 2.748758 |
| H | 3.828281 | 0.202448 | −2.14877 | H | 3.810875 | 0.205107 | −2.16059 | H | 3.810963 | 0.204839 | −2.160566 |
| H | 5.027343 | 0.216433 | −0.848197 | H | 5.022594 | 0.223539 | −0.867268 | H | 5.022626 | 0.223288 | −0.867231 |
| H | 3.038927 | −1.879211 | 1.509302 | H | 3.063849 | −1.878508 | 1.507882 | H | 3.063626 | −1.878436 | 1.508021 |
| H | 4.569046 | −1.011234 | 1.291022 | H | 4.585608 | −0.999272 | 1.276521 | H | 4.585489 | −0.99938 | 1.276645 |
| H | 3.507873 | −2.912432 | −0.668851 | H | 3.520579 | −2.918047 | −0.655857 | H | 3.520319 | −2.918117 | −0.655792 |
| H | 3.06641 | 2.433478 | 1.473847 | H | 3.071475 | 2.434969 | 1.463046 | H | 3.071661 | 2.435036 | 1.462929 |
| H | 4.578157 | 1.539684 | 1.283683 | H | 4.586106 | 1.545034 | 1.264694 | H | 4.586186 | 1.544954 | 1.264627 |
| H | −3.393989 | −2.675927 | 0.208367 | H | −3.418641 | −2.678929 | 0.176902 | H | −3.418401 | −2.67895 | 0.176812 |
| H | −2.688341 | 0.536259 | −2.000273 | H | −2.675904 | 0.536828 | −1.991892 | H | −2.676055 | 0.536945 | −1.991891 |
| H | −5.72877 | −1.488696 | −0.234992 | H | −5.733002 | −1.480701 | −0.26066 | H | −5.732951 | −1.480849 | −0.260851 |
| H | −4.752611 | −1.568139 | 2.094983 | H | −4.786045 | −1.584431 | 2.073665 | H | −4.785952 | −1.58477 | 2.073551 |
| H | −3.854275 | −0.004727 | 1.754431 | H | −3.863215 | −0.02778 | 1.760745 | H | −3.86342 | −0.027938 | 1.76074 |
| H | −4.9672 | 0.167577 | −1.887202 | H | −4.953913 | 0.179125 | −1.897621 | H | −4.954101 | 0.17948 | −1.897435 |
| H | −4.976783 | 1.234507 | −0.467255 | H | −4.978529 | 1.24073 | −0.477609 | H | −4.978484 | 1.240703 | −0.477106 |
| N | −3.159003 | 2.507575 | 0.32101 | N | −3.140981 | 2.537266 | 0.308662 | N | −3.141066 | 2.537121 | 0.308882 |
| H | −2.599366 | 3.210491 | 0.808531 | H | −2.562516 | 3.22579 | 0.795809 | H | −2.562707 | 3.225721 | 0.796043 |

**Table S41.** Coordinates of ESCA.

| In gas phase | | | | In water | | | | In formic acid | | | |
|---|---|---|---|---|---|---|---|---|---|---|---|
| C | 1.009901 | 0.343566 | −0.471211 | C | 1.002571 | 0.323081 | −0.481283 | C | 1.002602 | 0.323296 | −0.481251 |
| C | −0.299325 | 0.47298 | −1.299682 | C | −0.301557 | 0.428949 | −1.323887 | C | −0.301536 | 0.429387 | −1.323794 |
| C | 1.786851 | −0.918309 | −0.889853 | C | 1.778287 | −0.951438 | −0.861716 | C | 1.778325 | −0.95113 | −0.861985 |
| C | 1.90355 | 1.570679 | −0.74314 | C | 1.896921 | 1.541742 | −0.788066 | C | 1.896986 | 1.542015 | −0.787687 |
| C | 0.723508 | 0.27703 | 1.039665 | C | 0.71526 | 0.297104 | 1.030747 | C | 0.715224 | 0.296973 | 1.030762 |
| C | −1.191937 | −0.760379 | −1.158547 | C | −1.204989 | −0.790548 | −1.156433 | C | −1.204846 | −0.790257 | −1.156587 |
| N | −1.030138 | 1.705907 | −1.028064 | N | −1.037641 | 1.665966 | −1.100665 | N | −1.037618 | 1.666346 | −1.10014 |
| C | 3.102414 | −1.03015 | −0.10929 | C | 3.094275 | −1.041117 | −0.078377 | C | 3.094269 | −1.041033 | −0.078599 |
| C | 3.221933 | 1.462523 | 0.038373 | C | 3.213998 | 1.454324 | −0.002554 | C | 3.214026 | 1.454396 | −0.002129 |
| C | 2.03896 | 0.166233 | 1.8276 | C | 2.031417 | 0.208649 | 1.821587 | C | 2.031329 | 0.208282 | 1.821647 |
| N | −2.237874 | −0.63173 | −0.314375 | N | −2.248024 | −0.635306 | −0.322099 | N | −2.247868 | −0.635295 | −0.322109 |
| O | −0.947493 | −1.788544 | −1.773414 | O | −0.975431 | −1.838347 | −1.757132 | O | −0.975129 | −1.837868 | −1.757434 |
| C | 3.971208 | 0.194564 | −0.389792 | C | 3.963435 | 0.174501 | −0.395371 | C | 3.963458 | 0.174661 | −0.395215 |
| C | 2.791179 | −1.099539 | 1.391699 | C | 2.78426 | −1.068897 | 1.423044 | C | 2.784149 | −1.06919 | 1.422808 |
| O | 3.845071 | −2.158433 | −0.534051 | O | 3.83279 | −2.189582 | −0.472295 | O | 3.832851 | −2.189316 | −0.472763 |
| C | 2.912563 | 1.396351 | 1.541111 | C | 2.905387 | 1.429961 | 1.501475 | C | 2.905333 | 1.429665 | 1.501877 |
| C | −3.195569 | −1.683516 | −0.094158 | C | −3.224924 | −1.667509 | −0.080358 | C | −3.224504 | −1.667734 | −0.080396 |
| C | −2.584602 | 0.567398 | 0.448507 | C | −2.565664 | 0.576367 | 0.438573 | C | −2.565791 | 0.576257 | 0.4386 |
| C | −4.377015 | −1.057399 | 0.58433 | C | −4.388083 | −1.011369 | 0.60063 | C | −4.387841 | −1.011881 | 0.600576 |
| C | −3.486232 | −2.006399 | 1.344707 | C | −3.508521 | −1.966561 | 1.365435 | C | −3.508057 | −1.966872 | 1.365386 |
| C | −4.101231 | 0.43286 | 0.691932 | C | −4.08458 | 0.474697 | 0.682263 | C | −4.084684 | 0.474254 | 0.682262 |
| H | −0.010375 | 0.478624 | −2.357376 | H | −0.002643 | 0.410425 | −2.378056 | H | −0.002677 | 0.411117 | −2.377987 |
| H | 2.007371 | −0.893876 | −1.964899 | H | 1.998867 | −0.955752 | −1.937459 | H | 1.998949 | −0.955198 | −1.93771 |
| H | 1.18034 | −1.814071 | −0.711453 | H | 1.174535 | −1.841707 | −0.650156 | H | 1.1745 | −1.841424 | −0.65075 |
| H | 2.110352 | 1.640576 | −1.820717 | H | 2.101957 | 1.583248 | −1.86671 | H | 2.1021 | 1.583745 | −1.866313 |
| H | 1.37547 | 2.484857 | −0.444112 | H | 1.371056 | 2.464331 | −0.514339 | H | 1.371094 | 2.464544 | −0.513781 |
| H | 0.083346 | −0.589671 | 1.259294 | H | 0.077098 | −0.563565 | 1.277114 | H | 0.077001 | −0.563735 | 1.276855 |
| H | 0.182476 | 1.181216 | 1.349111 | H | 0.176968 | 1.209489 | 1.318967 | H | 0.176921 | 1.209305 | 1.319146 |
| H | −0.742463 | 2.524083 | −1.544519 | H | −0.714765 | 2.482524 | −1.601062 | H | −0.715014 | 2.482982 | −1.600556 |
| H | 3.838376 | 2.343193 | −0.173982 | H | 3.831443 | 2.327231 | −0.240348 | H | 3.831493 | 2.327361 | −0.239669 |
| H | 1.814733 | 0.113366 | 2.898879 | H | 1.80565 | 0.184203 | 2.893071 | H | 1.805523 | 0.183596 | 2.893121 |
| H | 4.208505 | 0.219902 | −1.459796 | H | 4.198443 | 0.175202 | −1.466695 | H | 4.198541 | 0.175574 | −1.466516 |
| H | 4.916736 | 0.094769 | 0.156091 | H | 4.908616 | 0.093915 | 0.155094 | H | 4.90861 | 0.0939 | 0.155265 |

**Table S41.** *Cont.*

| In gas phase | | | | In water | | | | In formic acid | | | |
|---|---|---|---|---|---|---|---|---|---|---|---|
| H | 2.18336 | −1.992439 | 1.595196 | H | 2.178724 | −1.95649 | 1.652335 | H | 2.178541 | −1.956815 | 1.651825 |
| H | 3.731924 | −1.20628 | 1.944468 | H | 3.72509 | −1.153639 | 1.980042 | H | 3.724936 | −1.154161 | 1.979839 |
| H | 3.284098 | −2.936572 | −0.4272 | H | 3.290869 | −2.965567 | −0.276833 | H | 3.290528 | −2.965341 | −0.278637 |
| H | 2.392661 | 2.308696 | 1.859113 | H | 2.385412 | 2.350732 | 1.792641 | H | 2.385356 | 2.350369 | 1.793265 |
| H | 3.845852 | 1.335351 | 2.113763 | H | 3.8385 | 1.383759 | 2.075808 | H | 3.838418 | 1.383319 | 2.076241 |
| H | −3.238714 | −2.429489 | −0.876163 | H | −3.300665 | −2.419558 | −0.85351 | H | −3.299968 | −2.419822 | −0.853539 |
| H | −5.382427 | −1.377617 | 0.337903 | H | −5.399836 | −1.317267 | 0.363224 | H | −5.399529 | −1.31801 | 0.36318 |
| H | −3.879048 | −2.994098 | 1.55633 | H | −3.920473 | −2.943714 | 1.588835 | H | −3.919742 | −2.944134 | 1.5888 |
| H | −2.820298 | −1.610017 | 2.105648 | H | −2.826824 | −1.574665 | 2.11454 | H | −2.826492 | −1.574807 | 2.114524 |
| H | −4.63704 | 0.974672 | −0.09073 | H | −4.614929 | 1.004687 | −0.11262 | H | −4.615089 | 1.004205 | −0.112604 |
| H | −4.38258 | 0.870143 | 1.651593 | H | −4.357473 | 0.930696 | 1.635674 | H | −4.357708 | 0.930211 | 1.635653 |
| C | −2.172081 | 1.829474 | −0.278201 | C | −2.119572 | 1.829659 | −0.284907 | C | −2.120002 | 1.829674 | −0.28483 |
| H | −2.053596 | 0.556705 | 1.410878 | H | −2.038627 | 0.549041 | 1.401418 | H | −2.038732 | 0.549057 | 1.401447 |
| N | −2.878605 | 2.87857 | −0.106206 | N | −2.762129 | 2.915495 | −0.063712 | N | −2.763069 | 2.915222 | −0.063896 |
| H | −2.487807 | 3.676756 | −0.611377 | H | −2.338841 | 3.693221 | −0.575038 | H | −2.340076 | 3.693142 | −0.575173 |

**Table S42.** Coordinates of Saxagliptin_s.

| In gas phase | | | | In water | | | | In formic acid | | | |
|---|---|---|---|---|---|---|---|---|---|---|---|
| O | −0.363941 | −1.787709 | −0.964504 | O | −0.341212 | −1.785124 | −0.939202 | O | −0.34143 | −1.785211 | −0.939549 |
| N | 0.92219 | 0.026764 | −0.663242 | N | 0.92815 | 0.042455 | −0.645718 | N | 0.928042 | 0.04227 | −0.64584 |
| N | −2.481033 | −0.185319 | −1.879843 | N | −2.440941 | −0.170424 | −1.906754 | N | −2.441435 | −0.170713 | −1.906427 |
| N | 2.081849 | −1.92541 | 1.90073 | N | 2.062442 | −2.046242 | 1.809553 | N | 2.063007 | −2.045087 | 1.810494 |
| C | −2.264252 | 0.264869 | 0.54767 | C | −2.274835 | 0.25419 | 0.537458 | C | −2.274732 | 0.254297 | 0.537588 |
| C | −1.262099 | 0.620892 | 1.650639 | C | −1.289975 | 0.603072 | 1.658576 | C | −1.289685 | 0.603378 | 1.658473 |
| C | −3.384175 | 1.308664 | 0.518665 | C | −3.393096 | 1.300079 | 0.506345 | C | −3.393058 | 1.300114 | 0.50642 |
| C | −2.862593 | −1.117657 | 0.830667 | C | −2.8796 | −1.128366 | 0.804413 | C | −2.879372 | −1.128254 | 0.804859 |
| C | −1.565395 | 0.284464 | −0.84586 | C | −1.551436 | 0.288552 | −0.842652 | C | −1.55162 | 0.288418 | −0.842683 |
| C | 1.249928 | 1.413499 | −0.426507 | C | 1.238525 | 1.434849 | −0.398362 | C | 1.238531 | 1.434646 | −0.398647 |
| C | 2.745705 | 1.513318 | −0.387174 | C | 2.730685 | 1.550742 | −0.337821 | C | 2.730727 | 1.550464 | −0.338404 |
| C | 1.943318 | 1.731237 | 0.869119 | C | 1.911512 | 1.739529 | 0.91113 | C | 1.911779 | 1.739532 | 0.910661 |
| C | 3.302147 | 0.128802 | −0.667196 | C | 3.313978 | 0.179988 | −0.626962 | C | 3.313819 | 0.1796 | −0.627446 |
| C | −0.297435 | −0.573596 | −0.824017 | C | −0.279495 | −0.56318 | −0.807941 | C | −0.279693 | −0.563355 | −0.808107 |

**Table S42.** *Cont.*

| In gas phase | | | | In water | | | | In formic acid | | | |
|---|---|---|---|---|---|---|---|---|---|---|---|
| C | 2.096513 | −0.826909 | −0.495614 | C | 2.118643 | −0.796573 | −0.512258 | C | 2.118442 | −0.796834 | −0.512092 |
| C | 2.081751 | −1.464595 | 0.840734 | C | 2.089096 | −1.507738 | 0.786815 | C | 2.089189 | −1.507284 | 0.78738 |
| H | −0.4754 | −0.136066 | 1.753498 | H | −0.500197 | −0.151324 | 1.758959 | H | −0.499975 | −0.151064 | 1.758977 |
| H | −0.785558 | 1.589468 | 1.460856 | H | −0.813617 | 1.573782 | 1.48361 | H | −0.81327 | 1.574017 | 1.483226 |
| H | −2.974837 | 2.319041 | 0.403677 | H | −2.981188 | 2.307953 | 0.384458 | H | −2.981231 | 2.308032 | 0.384553 |
| H | −4.062948 | 1.111776 | −0.31402 | H | −4.077432 | 1.102613 | −0.321829 | H | −4.077295 | 1.102566 | −0.321812 |
| H | −3.643243 | −1.358091 | 0.103214 | H | −3.627381 | −1.380691 | 0.04664 | H | −3.627471 | −1.380595 | 0.04741 |
| H | −2.099708 | −1.900035 | 0.799676 | H | −2.111664 | −1.906269 | 0.809996 | H | −2.111442 | −1.906163 | 0.810204 |
| H | 0.634195 | 2.146865 | −0.930801 | H | 0.621021 | 2.165525 | −0.903051 | H | 0.620983 | 2.165308 | −0.903323 |
| H | −1.312844 | 1.328126 | −1.063652 | H | −1.295828 | 1.332866 | −1.044989 | H | −1.29605 | 1.332713 | −1.04524 |
| H | 3.246909 | 2.363003 | −0.835618 | H | 3.222968 | 2.413263 | −0.770009 | H | 3.223033 | 2.412874 | −0.770799 |
| H | 1.950134 | 0.969753 | 1.643156 | H | 1.917953 | 0.962553 | 1.670434 | H | 1.91829 | 0.962738 | 1.67014 |
| H | 1.869795 | 2.750103 | 1.232787 | H | 1.816955 | 2.750044 | 1.291748 | H | 1.817409 | 2.750139 | 1.291094 |
| H | 4.130937 | −0.153651 | −0.01474 | H | 4.127738 | −0.103251 | 0.043508 | H | 4.127785 | −0.10355 | 0.042813 |
| H | 3.646498 | 0.079649 | −1.703184 | H | 3.688564 | 0.151728 | −1.651863 | H | 3.68806 | 0.15111 | −1.652478 |
| H | 2.069204 | −1.629747 | −1.236099 | H | 2.120043 | −1.562469 | −1.29133 | H | 2.119594 | −1.563128 | −1.290772 |
| H | −2.472399 | −1.203742 | −1.849793 | H | −2.425493 | −1.189036 | −1.896871 | H | −2.42626 | −1.189326 | −1.896135 |
| H | −2.117757 | 0.066763 | −2.794639 | H | −2.047052 | 0.098249 | −2.804966 | H | −2.047756 | 0.097518 | −2.804856 |
| H | −3.951528 | 1.280891 | 1.454434 | H | −3.958274 | 1.27652 | 1.443631 | H | −3.958277 | 1.276537 | 1.443679 |
| H | −3.320928 | −1.119697 | 1.824741 | H | −3.378159 | −1.126857 | 1.779079 | H | −3.377543 | −1.126675 | 1.779722 |
| H | −1.780521 | 0.687093 | 2.612258 | H | −1.82083 | 0.654411 | 2.614444 | H | −1.820409 | 0.65501 | 2.614395 |

**Table S43.** Coordinates of SCA_s.

| In gas phase | | | | In water | | | | In formic acid | | | |
|---|---|---|---|---|---|---|---|---|---|---|---|
| C | 2.629674 | −0.042185 | −0.027861 | C | 2.629674 | −0.042185 | −0.027861 | C | 2.629835 | −0.048784 | −0.027237 |
| C | 1.164385 | 0.165135 | −0.463548 | C | 1.164385 | 0.165135 | −0.463548 | C | 1.161719 | 0.159838 | −0.452454 |
| C | 3.207921 | −1.231779 | −0.806361 | C | 3.207921 | −1.231779 | −0.806361 | C | 3.206409 | −1.226881 | −0.82422 |
| C | 3.437533 | 1.211745 | −0.395772 | C | 3.437533 | 1.211745 | −0.395772 | C | 3.433183 | 1.209873 | −0.39104 |
| C | 2.745411 | −0.307666 | 1.479051 | C | 2.745411 | −0.307666 | 1.479051 | C | 2.758203 | −0.324237 | 1.476494 |
| C | 0.212149 | −1.010126 | −0.164669 | C | 0.212149 | −1.010126 | −0.164669 | C | 0.206498 | −1.01386 | −0.163816 |
| N | 0.588136 | 1.373942 | 0.138645 | N | 0.588136 | 1.373942 | 0.138645 | N | 0.580641 | 1.351389 | 0.174635 |
| N | −1.070418 | −0.706008 | −0.505246 | N | −1.070418 | −0.706008 | −0.505246 | N | −1.072589 | −0.701429 | −0.487735 |

**Table S43.** *Cont.*

| In gas phase | | | | In water | | | | In formic acid | | | |
|---|---|---|---|---|---|---|---|---|---|---|---|
| O | 0.53335 | −2.081389 | 0.322482 | O | 0.53335 | −2.081389 | 0.322482 | O | 0.523698 | −2.099333 | 0.308747 |
| C | −2.185685 | −1.476975 | −0.014304 | C | −2.185685 | −1.476975 | −0.014304 | C | −2.194255 | −1.48011 | −0.022269 |
| C | −1.461073 | 0.667911 | −0.867834 | C | −1.461073 | 0.667911 | −0.867834 | C | −1.460405 | 0.674199 | −0.85494 |
| C | −3.406287 | −0.628457 | −0.167928 | C | −3.406287 | −0.628457 | −0.167928 | C | −3.410428 | −0.625143 | −0.167286 |
| C | −2.866457 | −0.920336 | 1.20648 | C | −2.866457 | −0.920336 | 1.20648 | C | −2.878169 | −0.940012 | 1.20553 |
| C | −2.998964 | 0.685843 | −0.819307 | C | −2.998964 | 0.685843 | −0.819307 | C | −2.997844 | 0.693341 | −0.805578 |
| C | −0.76355 | 1.624948 | 0.08919 | C | −0.76355 | 1.624948 | 0.08919 | C | −0.754018 | 1.637092 | 0.090726 |
| H | 1.173577 | 0.29771 | −1.557956 | H | 1.173577 | 0.29771 | −1.557956 | H | 1.164284 | 0.303661 | −1.545347 |
| H | 3.143862 | −1.056175 | −1.886253 | H | 3.143862 | −1.056175 | −1.886253 | H | 3.133728 | −1.038304 | −1.901038 |
| H | 2.677272 | −2.153096 | −0.565117 | H | 2.677272 | −2.153096 | −0.565117 | H | 2.68032 | −2.153341 | −0.592242 |
| H | 3.34059 | 1.443435 | −1.461712 | H | 3.34059 | 1.443435 | −1.461712 | H | 3.321564 | 1.45255 | −1.453038 |
| H | 3.127741 | 2.096677 | 0.168151 | H | 3.127741 | 2.096677 | 0.168151 | H | 3.135215 | 2.08723 | 0.190404 |
| H | 2.199117 | −1.210083 | 1.757132 | H | 2.199117 | −1.210083 | 1.757132 | H | 2.218438 | −1.231495 | 1.752443 |
| H | 2.365108 | 0.528628 | 2.077205 | H | 2.365108 | 0.528628 | 2.077205 | H | 2.376367 | 0.505276 | 2.081189 |
| H | 1.103089 | 1.78556 | 0.904029 | H | 1.103089 | 1.78556 | 0.904029 | H | 1.126822 | 1.832963 | 0.874988 |
| H | −2.146311 | −2.540446 | −0.210838 | H | −2.146311 | −2.540446 | −0.210838 | H | −2.166769 | −2.539006 | −0.24429 |
| H | −1.095461 | 0.896912 | −1.878365 | H | −1.095461 | 0.896912 | −1.878365 | H | −1.097606 | 0.894389 | −1.867531 |
| H | −4.349127 | −1.075544 | −0.460534 | H | −4.349127 | −1.075544 | −0.460534 | H | −4.351322 | −1.069435 | −0.469063 |
| H | −3.422888 | −1.611861 | 1.828841 | H | −3.422888 | −1.611861 | 1.828841 | H | −3.442893 | −1.636575 | 1.814585 |
| H | −2.368919 | −0.112304 | 1.735061 | H | −2.368919 | −0.112304 | 1.735061 | H | −2.36991 | −0.147132 | 1.747548 |
| H | −3.401205 | 0.734727 | −1.833762 | H | −3.401205 | 0.734727 | −1.833762 | H | −3.400514 | 0.753511 | −1.818499 |
| H | −3.336577 | 1.563342 | −0.263817 | H | −3.336577 | 1.563342 | −0.263817 | H | −3.337122 | 1.565527 | −0.243517 |
| N | −1.420837 | 2.519232 | 0.71797 | N | −1.420837 | 2.519232 | 0.71797 | N | −1.393586 | 2.574047 | 0.68317 |
| H | −0.806408 | 3.077689 | 1.314405 | H | −0.806408 | 3.077689 | 1.314405 | H | −0.760016 | 3.125428 | 1.266574 |
| H | 4.265393 | −1.355762 | −0.552573 | H | 4.265393 | −1.355762 | −0.552573 | H | 4.265279 | −1.350863 | −0.577051 |
| H | 3.799329 | −0.436352 | 1.744838 | H | 3.799329 | −0.436352 | 1.744838 | H | 3.814704 | −0.451694 | 1.73212 |
| H | 4.496506 | 1.03955 | −0.180996 | H | 4.496506 | 1.03955 | −0.180996 | H | 4.494207 | 1.03203 | −0.192156 |

**Table S44.** Coordinates of ESCA_s.

| In gas phase | | | | In water | | | | In formic acid | | | |
|---|---|---|---|---|---|---|---|---|---|---|---|
| C | 2.359304 | −0.241626 | 0.345357 | C | 2.341604 | −0.250447 | 0.352353 | C | 2.341688 | −0.250391 | 0.352313 |
| C | 1.323642 | 0.087156 | −0.773173 | C | 1.320369 | 0.04928 | −0.789153 | C | 1.320407 | 0.049533 | −0.789089 |
| C | 2.960779 | −1.630273 | 0.102503 | C | 2.917686 | −1.659676 | 0.174621 | C | 2.917957 | −1.659487 | 0.174171 |
| C | 3.478809 | 0.80372 | 0.284759 | C | 3.482869 | 0.768297 | 0.25108 | C | 3.48281 | 0.768538 | 0.251375 |
| C | 1.697116 | −0.193554 | 1.724808 | C | 1.677729 | −0.133048 | 1.72707 | C | 1.677742 | −0.133484 | 1.727038 |
| C | 0.188047 | −0.934063 | −0.827668 | C | 0.173327 | −0.956114 | −0.826645 | C | 0.173439 | −0.955975 | −0.826701 |
| N | 0.819506 | 1.453853 | −0.704293 | N | 0.82127 | 1.418094 | −0.77465 | N | 0.821262 | 1.418336 | −0.774241 |
| N | −0.982617 | −0.537935 | −0.281577 | N | −0.98836 | −0.539844 | −0.290014 | N | −0.988299 | −0.539836 | −0.289967 |
| O | 0.352689 | −2.032687 | −1.337679 | O | 0.320417 | −2.069403 | −1.326433 | O | 0.320647 | −2.069155 | −1.326559 |
| C | −2.16622 | −1.35703 | −0.295684 | C | −2.188099 | −1.339035 | −0.288335 | C | −2.187905 | −1.339196 | −0.288302 |
| C | −1.241111 | 0.747946 | 0.365446 | C | −1.223022 | 0.756126 | 0.352284 | C | −1.223161 | 0.756086 | 0.352302 |
| C | −3.316098 | −0.460807 | 0.055546 | C | −3.319548 | −0.420337 | 0.063291 | C | −3.319513 | −0.42065 | 0.063224 |
| C | −2.862068 | −1.521075 | 1.026292 | C | −2.875994 | −1.477745 | 1.041224 | C | −2.875858 | −1.477966 | 1.041217 |
| C | −2.762959 | 0.945117 | 0.216705 | C | −2.741106 | 0.977353 | 0.201644 | C | −2.741272 | 0.977125 | 0.201605 |
| H | 1.83879 | −0.030672 | −1.733661 | H | 1.847547 | −0.096058 | −1.73844 | H | 1.847516 | −0.09564 | −1.738448 |
| H | 3.413947 | −1.693729 | −0.892299 | H | 3.363996 | −1.779532 | −0.818173 | H | 3.364311 | −1.778993 | −0.818642 |
| H | 2.206577 | −2.415948 | 0.169574 | H | 2.1516 | −2.428242 | 0.294153 | H | 2.151949 | −2.428178 | 0.293342 |
| H | 3.949986 | 0.821087 | −0.705103 | H | 3.959271 | 0.728959 | −0.734846 | H | 3.959409 | 0.729391 | −0.734467 |
| H | 3.105236 | 1.806578 | 0.512712 | H | 3.128184 | 1.788156 | 0.426416 | H | 3.127959 | 1.788323 | 0.426826 |
| H | 0.895666 | −0.936399 | 1.810167 | H | 0.858043 | −0.851952 | 1.842288 | H | 0.858178 | −0.852572 | 1.841979 |
| H | 1.283238 | 0.79986 | 1.92796 | H | 1.289107 | 0.8769 | 1.894105 | H | 1.288937 | 0.876355 | 1.894331 |
| H | 1.398139 | 2.169617 | −1.118578 | H | 1.42478 | 2.123413 | −1.174302 | H | 1.424725 | 2.123784 | −1.17371 |
| H | −2.175241 | −2.128249 | −1.05413 | H | −2.226779 | −2.112461 | −1.043128 | H | −2.226346 | −2.112698 | −1.043031 |
| H | −4.275676 | −0.583528 | −0.432663 | H | −4.282798 | −0.533636 | −0.419301 | H | −4.282735 | −0.534077 | −0.419398 |
| H | −3.49588 | −2.392565 | 1.141773 | H | −3.525786 | −2.335899 | 1.166404 | H | −3.525494 | −2.336236 | 1.166412 |
| H | −2.334303 | −1.221136 | 1.926909 | H | −2.334444 | −1.179204 | 1.934056 | H | −2.334446 | −1.179304 | 1.934098 |
| H | −2.961212 | 1.535783 | −0.6807 | H | −2.93582 | 1.550737 | −0.708027 | H | −2.935981 | 1.550526 | −0.708052 |
| H | −3.170573 | 1.489898 | 1.070165 | H | −3.138758 | 1.538817 | 1.049068 | H | −3.139019 | 1.538586 | 1.048984 |
| C | −0.40214 | 1.856524 | −0.231061 | C | −0.359224 | 1.847624 | −0.242557 | C | −0.359499 | 1.847704 | −0.242486 |
| H | −0.982537 | 0.681527 | 1.431559 | H | −0.969841 | 0.684258 | 1.418042 | H | −0.969986 | 0.684297 | 1.418073 |
| N | −0.876905 | 3.041704 | −0.213687 | N | −0.786206 | 3.054269 | −0.183678 | N | −0.786746 | 3.054227 | −0.183779 |
| H | −0.212315 | 3.712985 | −0.604302 | H | −0.095838 | 3.697874 | −0.576846 | H | −0.096531 | 3.698014 | −0.576913 |
| H | 3.741266 | −1.823614 | 0.845379 | H | 3.699604 | −1.827967 | 0.921583 | H | 3.699857 | −1.827929 | 0.921121 |
| H | 2.435689 | −0.410102 | 2.502846 | H | 2.412146 | −0.337688 | 2.512066 | H | 2.412139 | −0.338232 | 2.512025 |
| H | 4.255185 | 0.564536 | 1.017746 | H | 4.245975 | 0.547446 | 1.003476 | H | 4.245855 | 0.547706 | 1.003839 |

**Table S45.** Coordinates of TS_autoatalysis.

| **In gas phase** | | | | **In water** | | | | In formic acid | | | |
|---|---|---|---|---|---|---|---|---|---|---|---|
| C | 3.062579 | −0.801969 | −1.646567 | C | 3.100825 | −0.782176 | −1.633459 | C | 3.099126 | −0.78344 | −1.63341 |
| N | 4.177997 | −0.48215 | −0.741154 | N | 4.202158 | −0.422233 | −0.722362 | N | 4.201886 | −0.42528 | −0.72339 |
| C | 5.055704 | −1.609577 | −0.541954 | C | 5.150841 | −1.497023 | −0.559675 | C | 5.148433 | −1.50198 | −0.56088 |
| C | 4.593449 | −2.692084 | −1.463647 | C | 4.732834 | −2.592186 | −1.48645 | C | 4.727766 | −2.5965 | −1.48723 |
| C | 3.418612 | −2.162001 | −2.27273 | C | 3.521148 | −2.115521 | −2.273911 | C | 3.516574 | −2.11759 | −2.27407 |
| C | 4.401742 | −2.842106 | 0.021923 | C | 4.580115 | −2.772444 | 0.001023 | C | 4.575398 | −2.77613 | 0.000336 |
| C | 4.10959 | 0.527345 | 0.161344 | C | 4.088913 | 0.574586 | 0.183552 | C | 4.091068 | 0.571851 | 0.182525 |
| C | 2.867572 | 1.420504 | −0.044159 | C | 2.812496 | 1.415132 | −0.021397 | C | 2.815768 | 1.414365 | −0.02134 |
| N | 1.677363 | 0.527658 | −0.020171 | N | 1.657121 | 0.475376 | −0.022156 | N | 1.659013 | 0.476313 | −0.01997 |
| C | 1.752928 | −0.829566 | −0.869434 | C | 1.794746 | −0.868516 | −0.846862 | C | 1.793578 | −0.86766 | −0.84571 |
| N | 0.841599 | −1.641865 | −0.83038 | N | 0.918205 | −1.723538 | −0.803457 | N | 0.915768 | −1.72128 | −0.80181 |
| N | −1.498229 | −0.682811 | −0.195089 | N | −1.506549 | −0.752691 | −0.22032 | N | −1.50722 | −0.75005 | −0.2176 |
| C | −1.670034 | 0.592566 | −0.489702 | C | −1.664007 | 0.516889 | −0.573173 | C | −1.66412 | 0.520061 | −0.56836 |
| N | −0.650293 | 1.364963 | −0.79114 | N | −0.638613 | 1.249795 | −0.926168 | N | −0.63816 | 1.25373 | −0.91838 |
| C | −3.071634 | 1.166563 | −0.429373 | C | −3.058874 | 1.100953 | −0.519421 | C | −3.05906 | 1.104167 | −0.51578 |
| N | −4.092872 | 0.134271 | −0.542128 | N | −4.085298 | 0.06342 | −0.601211 | N | −4.08546 | 0.066904 | −0.60103 |
| C | −3.925714 | −1.189155 | −0.284154 | C | −3.932412 | −1.229302 | −0.249694 | C | −3.93304 | −1.2269 | −0.25288 |
| C | −2.55043 | −1.5866 | 0.234932 | C | −2.561481 | −1.60792 | 0.297048 | C | −2.56306 | −1.60691 | 0.295152 |
| C | −2.539078 | −1.803343 | 1.779568 | C | −2.535131 | −1.701976 | 1.854479 | C | −2.53978 | −1.70557 | 1.85233 |
| O | −4.815568 | −2.007586 | −0.453193 | O | −4.828708 | −2.061152 | −0.365792 | O | −4.82914 | −2.05834 | −0.37263 |
| C | −5.279328 | 0.649036 | −1.173767 | C | −5.273804 | 0.566485 | −1.244693 | C | −5.2729 | 0.571289 | −1.24543 |
| C | −4.904783 | 1.957177 | −1.80695 | C | −4.897505 | 1.8604 | −1.902808 | C | −4.89544 | 1.866298 | −1.90071 |
| C | −3.419688 | 2.1601 | −1.56552 | C | −3.411073 | 2.06819 | −1.674179 | C | −3.40933 | 2.073413 | −1.66944 |
| C | −5.821151 | 1.930257 | −0.609838 | C | −5.81037 | 1.861102 | −0.704111 | C | −5.81011 | 1.865004 | −0.70338 |
| C | 2.754842 | 2.614872 | 0.931034 | C | 2.632162 | 2.594569 | 0.962467 | C | 2.638929 | 2.59478 | 0.961971 |
| O | 4.938798 | 0.683216 | 1.045496 | O | 4.919228 | 0.759874 | 1.068262 | O | 4.922327 | 0.755848 | 1.066559 |
| H | −0.470126 | −1.129446 | −0.373791 | H | −0.511241 | −1.185322 | −0.346992 | H | −0.51164 | −1.18293 | −0.34468 |
| H | 0.646849 | 1.004299 | −0.409708 | H | 0.626542 | 0.923866 | −0.471492 | H | 0.627628 | 0.926078 | −0.46653 |
| H | 3.717012 | −2.032267 | −3.315735 | H | 3.797381 | −1.96396 | −3.319425 | H | 3.792528 | −1.96656 | −3.31975 |
| H | 6.091555 | −1.3707 | −0.33956 | H | 6.178377 | −1.205382 | −0.385102 | H | 6.176604 | −1.21236 | −0.38672 |
| H | 5.314442 | −3.331023 | −1.960032 | H | 5.483336 | −3.180473 | −2.000731 | H | 5.476761 | −3.18645 | −2.00182 |
| H | 2.538148 | −2.808561 | −2.232636 | H | 2.678952 | −2.810785 | −2.229584 | H | 2.67303 | −2.81117 | −2.22922 |
| H | 2.984886 | −0.023583 | −2.41787 | H | 2.990199 | 0.00098 | −2.394721 | H | 2.989114 | −0.00021 | −2.39468 |
| H | 2.91877 | 1.831193 | −1.062446 | H | 2.861386 | 1.838676 | −1.033987 | H | 2.863932 | 1.83716 | −1.03428 |
| H | 5.037671 | −3.549642 | 0.541793 | H | 5.264852 | −3.452182 | 0.495358 | H | 5.259036 | −3.45709 | 0.494514 |

**Table S45.** *Cont.*

| **In gas phase** | | | | **In water** | | | | In formic acid | | | |
|---|---|---|---|---|---|---|---|---|---|---|---|
| H | 3.393428 | −2.744106 | 0.413856 | H | 3.579 | −2.72579 | 0.420439 | H | 3.574549 | −2.72753 | 0.420148 |
| H | 1.503864 | 0.22026 | 0.93809 | H | 1.450868 | 0.183629 | 0.934843 | H | 1.454812 | 0.184083 | 0.937323 |
| H | −0.918984 | 2.313916 | −1.02215 | H | −0.88729 | 2.198096 | −1.184642 | H | −0.8865 | 2.202342 | −1.17598 |
| H | −2.344896 | −2.559642 | −0.226473 | H | −2.375552 | −2.617504 | −0.087684 | H | −2.37618 | −2.61532 | −0.0922 |
| H | −2.86297 | 1.891739 | −2.468834 | H | −2.862454 | 1.783356 | −2.576857 | H | −2.85937 | 1.78993 | −2.57173 |
| H | −3.161652 | 3.186815 | −1.29099 | H | −3.151628 | 3.098242 | −1.419091 | H | −3.15009 | 3.103019 | −1.41229 |
| H | −5.914911 | −0.105701 | −1.61654 | H | −5.911421 | −0.190129 | −1.680178 | H | −5.90994 | −0.18454 | −1.68312 |
| H | −5.310487 | 2.243048 | −2.769966 | H | −5.307265 | 2.121828 | −2.870676 | H | −5.30367 | 2.129574 | −2.86873 |
| H | −5.449501 | 2.2721 | 0.351589 | H | −5.434015 | 2.218459 | 0.249829 | H | −5.43514 | 2.220626 | 0.25175 |
| H | −6.869024 | 2.140664 | −0.788654 | H | −6.858171 | 2.067975 | −0.886958 | H | −6.85761 | 2.072376 | −0.88739 |
| H | −3.161822 | 1.677965 | 0.540632 | H | −3.142269 | 1.632195 | 0.438346 | H | −3.144 | 1.63379 | 0.44278 |
| C | 2.679042 | 2.162573 | 2.395622 | C | 2.565834 | 2.131344 | 2.423924 | C | 2.573178 | 2.132601 | 2.423777 |
| H | 3.570256 | 1.604674 | 2.682905 | H | 3.481991 | 1.61986 | 2.71961 | H | 3.488586 | 1.619406 | 2.718794 |
| H | 2.591327 | 3.043306 | 3.039843 | H | 2.425557 | 3.004331 | 3.069285 | H | 2.43539 | 3.006243 | 3.068792 |
| H | 1.791276 | 1.546777 | 2.586541 | H | 1.712469 | 1.466877 | 2.60446 | H | 1.718624 | 1.470002 | 2.605559 |
| C | 3.973764 | 3.525521 | 0.727648 | C | 3.80483 | 3.56712 | 0.771566 | C | 3.813386 | 3.564797 | 0.769269 |
| H | 3.873414 | 4.417296 | 1.354315 | H | 3.661918 | 4.440622 | 1.415192 | H | 3.67259 | 4.439351 | 1.411935 |
| H | 4.897498 | 3.011807 | 0.993421 | H | 4.754815 | 3.095907 | 1.025354 | H | 4.762563 | 3.092 | 1.023073 |
| H | 4.039101 | 3.858908 | −0.314675 | H | 3.849797 | 3.919541 | −0.264996 | H | 3.858466 | 3.915941 | −0.26773 |
| C | 1.488552 | 3.415187 | 0.596421 | C | 1.331664 | 3.3355 | 0.622059 | C | 1.339611 | 3.338147 | 0.622411 |
| H | 1.474848 | 4.33854 | 1.183384 | H | 1.274153 | 4.255484 | 1.211444 | H | 1.284438 | 4.258542 | 1.211374 |
| H | 1.45943 | 3.690763 | −0.464903 | H | 1.295472 | 3.610335 | −0.438615 | H | 1.302974 | 3.612516 | −0.43838 |
| H | 0.578956 | 2.855104 | 0.830529 | H | 0.446658 | 2.735988 | 0.853122 | H | 0.453692 | 2.740463 | 0.854605 |
| C | −1.133917 | −2.265112 | 2.181918 | C | −1.154004 | −2.220753 | 2.270461 | C | −1.15883 | −2.22372 | 2.269663 |
| H | −0.382704 | −1.501494 | 1.958608 | H | −0.356228 | −1.549574 | 1.938105 | H | −0.36125 | −1.55072 | 1.940526 |
| H | −1.102396 | −2.475967 | 3.255484 | H | −1.099859 | −2.304996 | 3.360177 | H | −1.10672 | −2.31075 | 3.359263 |
| H | −0.849186 | −3.174641 | 1.643112 | H | −0.963607 | −3.210634 | 1.841356 | H | −0.96636 | −3.21221 | 1.838329 |
| C | −2.875896 | −0.498028 | 2.506983 | C | −2.77813 | −0.327672 | 2.484757 | C | −2.78606 | −0.33344 | 2.486015 |
| H | −2.139549 | 0.281437 | 2.279657 | H | −1.995813 | 0.384468 | 2.199611 | H | −2.00365 | 0.380297 | 2.205253 |
| H | −3.873656 | −0.133617 | 2.236005 | H | −3.752883 | 0.080842 | 2.193243 | H | −3.76033 | 0.074948 | 2.192718 |
| H | −2.86145 | −0.657592 | 3.589579 | H | −2.76497 | −0.412382 | 3.575586 | H | −2.77606 | −0.42129 | 3.576628 |
| C | −3.552758 | −2.888073 | 2.158068 | C | −3.604126 | −2.68863 | 2.335856 | C | −3.60852 | −2.69503 | 2.32846 |
| H | −3.348288 | −3.817889 | 1.617874 | H | −3.490185 | −3.66144 | 1.846527 | H | −3.49249 | −3.66611 | 1.836216 |
| H | −3.483409 | −3.094836 | 3.230968 | H | −3.501982 | −2.835746 | 3.415608 | H | −3.5083 | −2.84552 | 3.40793 |
| H | −4.576309 | −2.589933 | 1.922797 | H | −4.613169 | −2.324832 | 2.130015 | H | −4.6176 | −2.33181 | 2.121785 |

## References

1. C.; Gerack, L. McElwee−White, Formylation of Amines, *Molecules*, **2014**, *19*, 7689.
2. G.S.; Jones, S.A.; Savage, S.; Ivy, P.L.; Benitez, A.; Ramirez, Correction to Kinetic and Mechanistic Insight into the Thermodynamic Degradation of Saxagliptin, *J. Org. Chem.*, **2013**, *78*, 4627.

© 2019 by the authors. Submitted for possible open access publication under the terms and conditions of the Creative Commons Attribution (CC BY) license (http://creativecommons.org/licenses/by/4.0/).
